# Site-Selective C–H alkylation of Complex Arenes by a Two-Step Aryl Thianthrenation-Reductive Alkylation Sequence

**DOI:** 10.1021/jacs.1c03459

**Published:** 2021-05-24

**Authors:** Beatrice Lansbergen, Paola Granatino, Tobias Ritter

**Affiliations:** Max-Planck-Institut für Kohlenforschung, Kaiser-Wilhelm Platz 1, D-45470 Mülheim an der Ruhr, Germany

## Abstract

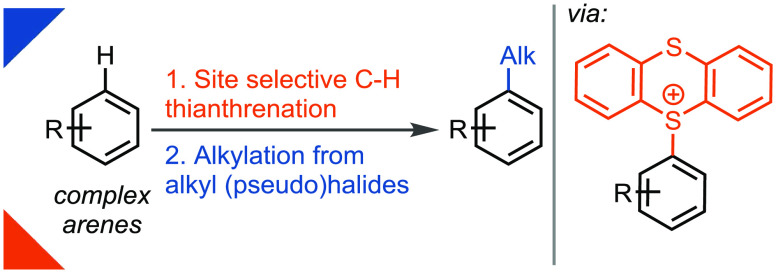

Herein, we present an undirected *para*-selective two-step C–H alkylation of complex arenes useful for late-stage functionalization. The combination of a site-selective C–H thianthrenation with palladium-catalyzed reductive electrophile cross-coupling grants access to a diverse range of synthetically useful alkylated arenes which cannot be accessed otherwise with comparable selectivity, diversity, and practicality. The robustness of this transformation is further demonstrated by thianthrenium-based reductive coupling of two complex fragments.

The Csp^2^–Csp^3^ coupling, particularly direct aryl C–H alkylation, has gained considerable attention as an attractive strategy for alkylation of arenes. While Csp^2^–Csp^2^ cross-coupling reactions have been common, Csp^2^–Csp^3^ cross-coupling reactions are less frequently used due to unresolved shortfalls in available methodologies.^1^ A significant challenge is the regioselective functionalization of structurally complex molecules at a late stage (Scheme 1). Though several methods exist to install alkyl groups via C–H functionalization,^2^ regioselective alkylations in the absence of directing groups remain problematic.^3^ The alkylation of arenes via aryl halides is efficient^4^ but lacks applicability to a wide class of aryl substrates due to the challenging site-selective synthesis of complex aryl halide starting materials.^5^ Simply put, there is currently no reaction chemistry available to introduce, in high positional selectivity, a diverse set of alkyl groups into complex small molecules.^6^ Herein, we present a solution to this problem by a Csp^2^–Csp^3^ reductive cross-coupling between complex aryl thianthrenium salts and readily available alkyl iodides, bromides, and triflates via a two-step undirected regioselective C–H functionalization/reductive alkylation sequence. We show that it is now possible to rapidly access a wide range of alkylated complex arenes, which cannot be accessed by other undirected C–H alkylation methods with the same selectivity, practicality, and diversity of substrates (Scheme 1b). The reactivity of this transformation is robust and can even be applied to two complex fragments. The reaction is thought to proceed via the *in situ* formation of an alkylzinc species. Compared to many other related Negishi-type aryl alkylations, thianthrene-based reductive couplings do not require the organometallic zinc to be preformed prior to the cross-coupling event.^4a,6a,7^ The ability to engage structurally complex arenes at a late stage, a broad selection of alkyl iodides, and excellent functional group tolerance distinguish this protocol to quickly access new value-added chemical entities.

**Scheme 1:**
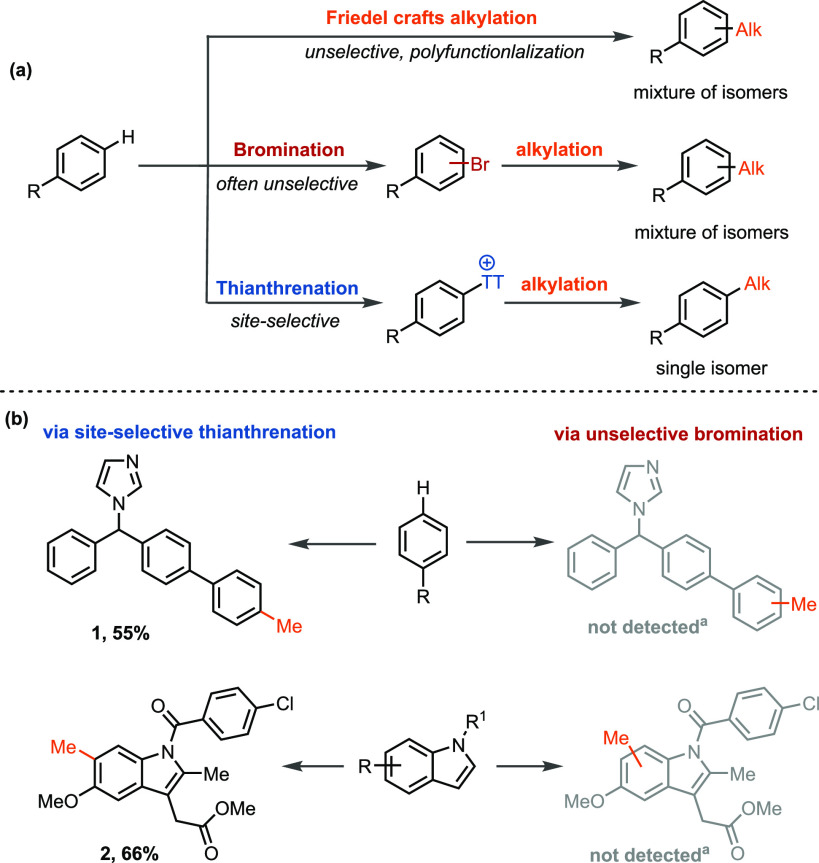
Strategies for Undirected C–H Alkylation of Arenes (a) Conceptual representation of various strategies for undirected C–H alkylation of arenes. (b) Experimental results for palladium-catalyzed aryl C–H alkylation via bromination versus thianthrenation. Two-step yield given for compounds **1** and **2**. ^a^Product not detected by LCMS, GCMS, ^1^H NMR spectroscopy, and ^13^C NMR spectroscopy (see the Supporting Information for details).

Selective direct aryl C–H alkylation reactions are difficult. Classical Friedel–Crafts C–H alkylations are limited by harsh conditions, low regioselectivity, and overalkylation (Scheme 1a).^8^ Synthetically useful regioselective C–H alkylations via transition-metal-catalyzed approaches are restricted to arenes that bear coordinating groups.^2b^ The Negishi reaction is one of the most efficient methods to alkylate aryl (pseudo)halides; however, a long-standing challenge is competitive ß-hydride elimination.^4a,9^ In addition, the required (pseudo)halides are often not available and can generally not be accessed in high selectivity from complex arenes (Scheme 1).^5^ Furthermore, the Negishi reaction and many other traditional transition-metal-catalyzed reactions remain constrained by the availability, stability, and reactivity of the organometallic nucleophiles that must be prepared separately from the electrophile prior to the cross-coupling event and may limit the substrates that can be employed. In view of these limitations, Weix,^10^ Molander,^11^ Gong,^12^ MacMillan,^13^ and others^14^ independently have achieved considerable progress in the field of reductive electrophile cross-coupling reactions and have successfully demonstrated the possibility of directly engaging two readily available halides for Csp^2^–Csp^3^ bond formation in the presence of a sacrificial reductant.^15^ Nonetheless, current reductive electrophile aryl alkylation reactions utilizing alkyl halides often require the use of an aryl (pseudo)halide, and progress toward complex small molecules has not been widely explored. Because general site-selective halogenation is difficult, the substrate scope consists mainly of simple arenes. Thianthrenium salts are promising electrophilic coupling partners for the late-stage site-selective introduction of alkyl motifs on structurally complex arenes to forge products that are currently challenging to access. Owning in part to their positive charge, they can be easier to reduce than aryl halides, which could present a further advantage.^6c^ We rationalized that the use of aryl thianthrenium salts in reductive alkylation reactions with alkyl halides would provide an unrealized opportunity for a two-step undirected *para*-selective C–H alkylation of complex arenes which, to date, has not been reported.

We investigated the reaction of aryl thianthrenium salt **TT-1** with 1-boc-4-iodopiperidine in the presence of a palladium catalyst and a reducing agent (Table 1). Zinc was found to be crucial for the reaction, and other reducing agents such as manganese and tetrakis(dimethylamino)ethylene did not produce any cross-coupled product, which is consistent with the involvement of an intermediate organozinc species. A preference for polar solvents such as DMF was observed as larger amounts of unreacted alkyl iodide remained when the reaction was conducted in less polar solvents, such as toluene, which could be explained by the faster rate of oxidative addition of zinc into alkyl iodides in polar solvents.^16^ While nickel is the preferred transition metal for reductive aryl–alkyl bond formations,^10a,10b,11−13^ we identified palladium to be the metal of choice for the reductive alkylation of aryl thianthrenium salts. A series of bulky phosphine ligands were tested including those that have been successful in previous aryl alkylation reactions to suppress competing ß-hydride elimination (see the Supporting Information, Table S1, entries 8 and 17).^7d,14d,17^ PdCl_2_(amphos)_2_ (Table 1) was found to be pivotal for efficient cross-coupling, and all other catalyst systems resulted in significantly lower yields. Simply replacing the ^t^Bu groups in amphos with cyclohexyl groups (L_2_) decreases the yield to 5%. A general challenge in transition-metal-catalyzed alkylation chemistry is control over regioselectivity due to reversible ß-hydride elimination, which often results in constitutional isomers.^4a,9^ We investigated the selectivity for the branched versus linear product in the cross-coupling of *i*-PrI with pyriproxyphen thianthrenium salt **TT-3** and found superior selectivities when amphos was used as a ligand (>20:1 of *i*-Pr product over *n*-Pr product).^7a,7d,7e,18^ PdCl_2_(tri*o*-tolyphosphine) and PdCl_2_(dppf), for example, only yielded the products in 4.2:1 and 0.84:1 selectivity, respectively (see Supporting Information, Table S2). The high selectivities for the branched product with PdCl_2_(amphos)_2_ are worth mentioning as competitive ß-hydride elimination is a reoccurring problem in palladium-catalyzed reactions.^7e,14k^ The importance of steric effects of the ligand on the extent of competing ß-hydride elimination can also be observed in Negishi-type aryl alkylations, which require specialized catalysts in order to minimize undesired ß-hydride elimination.^7a,7d,7e,18,19^ Noteworthy is also that the preformed PdCl_2_(amphos)_2_ complex was more efficient than the complex generated *in situ* from PdCl_2_ and amphos (L_1_) even when higher quantities of both PdCl_2_ and amphos were used. The presence of pyridine could potentially interfere with complex generation *in situ.* Nonetheless, pyridine was found to improve the overall yield of the reaction, possibly due to ligation to palladium after oxidative addition or to stabilize the organozinc reagent.^14d^

**Table 1:**
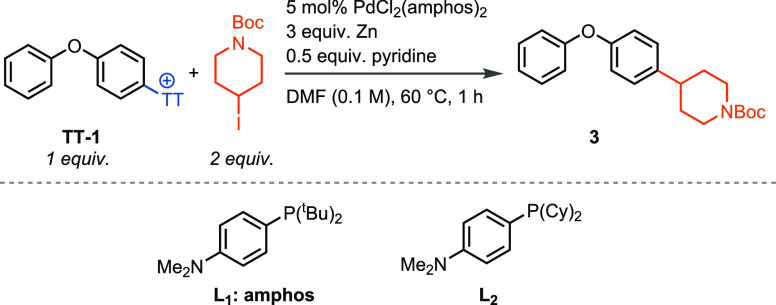
Optimization of the Reaction Conditions^a^

change in reaction conditions	yield^b^
none	75%
Mn instead of Zn	n.o.
TDAE instead of Zn	n.o.
toluene instead of DMF	20%
no catalyst	n.o.
NiBr_2_ + phen instead of PdCl_2_(amphos)_2_	n.o.
PdCl_2_ instead of PdCl_2_(amphos)_2_	<5%
PdCl_2_(dppf) instead of PdCl_2_(amphos)_2_	20%
10 mol % PdCl_2_ + 30 mol % L_2_ instead of PdCl_2_(amphos)_2_	5%
no pyridine	66%

a Reactions were carried out on a 0.1 mmol scale.

b Yields were determined by ^1^H NMR spectroscopy with mesitylene as the internal standard. TDAE = tetrakis(dimethylamino)ethylene. n.o. = not observed.

The alkylation of aryl thianthrenium salts occurred efficiently with primary and both cyclic and acylic secondary alkyl iodides; tertiary alkyl iodides could not be engaged (Scheme 3). Arenes as electron-rich as anisole to electron-poor as chlorobenzene were tolerated (see the Supporting Information, compound **S5**). The ability to employ a wide variety of alkyl substrates in a practical way presents an advantage to our previously published selective aryl C–H alkylations that only engage selected alkylzinc reagents and cannot operate on many complex small molecules.^6a,7g^ We targeted both alkyl and aryl substrates, which contain various functional groups, as high functional group tolerance is relevant for the application of this transformation late stage. Unprotected basic amines, acidic NH groups, strained heterocyclic ring systems (e.g., ß-lactam rings), and a range of basic heterocycles, often considered problematic in transition-metal-catalyzed reactions, did not hamper the reactivity. Despite being under reducing conditions, sulfones could be tolerated and were not reduced. Our catalytic system is also tolerant to sulfonamides, which is noteworthy as organozinc reagents are typically reactive toward such acidic functional groups.^20^ Alcohols, sulfides, and bromides were not compatible. Furthermore, the efficiency of the cross-coupling was not impeded by *ortho*-substituents (**2**, **5**, **15**, **16**, and **20**). Also the presence of a ketone on the alkyl halide was compatible with the cross-coupling, which requires protection as acetals in other, related protocols.^14k^ Other organometallic groups such as silyl and boron groups are not activated for transmetalation and can be held intact for potential further functionalization (**7** and **8**). As exemplified by **1**, **2**, and **15**, undirected selective methylations of aryl thianthrenium salts with methyl iodide instead of methylzinc chloride are now possible, which may have potential for isotopic labeling protocols.^6a,7g^ Alkyl substrates containing ß-σ acceptor substituents, which are difficult to engage by other methods such as S_N_2-type substitutions, could be coupled efficiently (e.g., **5**, **6**, **9**, **10**, **11**, **12**). Because the introduction of saturated heterocyclic motifs is often challenging, they are typically introduced via a more viable sp^2^–sp^2^ coupling followed by hydrogenation.^21^ In this transformation, a variety of saturated heterocyclic motifs could be successfully engaged such as oxetane, azetidine, piperidine, and oxaspiro[3.3]heptane (**5**, **10**, **11**, **12**, **14**, **17**). Radical ring-opening reactions such as observed for compound **13** can give rise to otherwise challenging to access structures. We also show that the thianthrene-based reductive cross-coupling can be successfully employed for the linkage of two complex building blocks (**18**, **19**, **20**, **21**), as exemplified by the coupling of a sulbactam iodide derivative, a privileged motif in drug discovery, which is found in 30% of the approved ß-lactam antibiotics,^22^ with nefiractam, a nootropic drug (**19**). In addition to alkyl iodides, primary alkyl bromides and triflates can be used successfully for the reductive cross-coupling. Secondary alkyl bromides are reactive as well, albeit in lower yields; for example, pyriproxyfen thianthrenium salt **TT-3** with 3-bromooxetane and 3-iodooxetane gave product **10** at 26% and 72% yields, respectively (see the Supporting Information, pp S41–S44 for details).

**Scheme 2:**
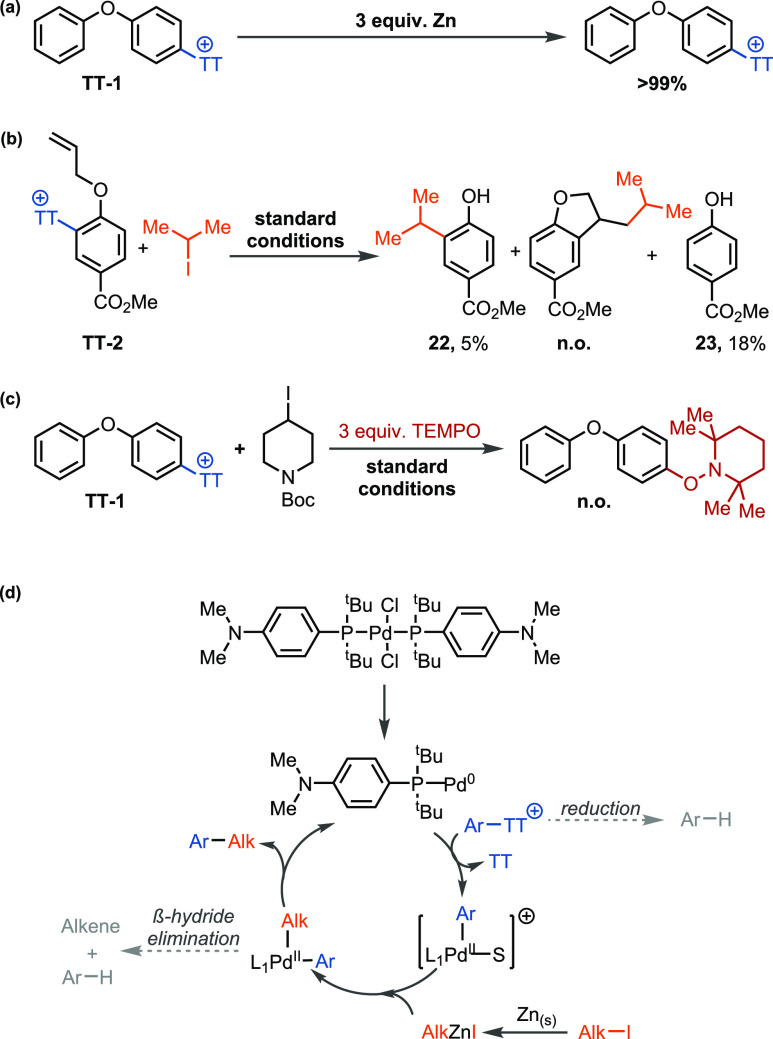
Mechanistic Investigation (a) Zinc insertion experiment. (b) Radical clock cyclization of allyl ether thianthrenium salt **TT-2** under standard conditions. (c) Radical trapping experiment with TEMPO under standard conditions. (d) Mechanistic hypothesis. S = solvent or pyridine. L_1_ = amphos. n.o. = not observed.

**Scheme 3:**
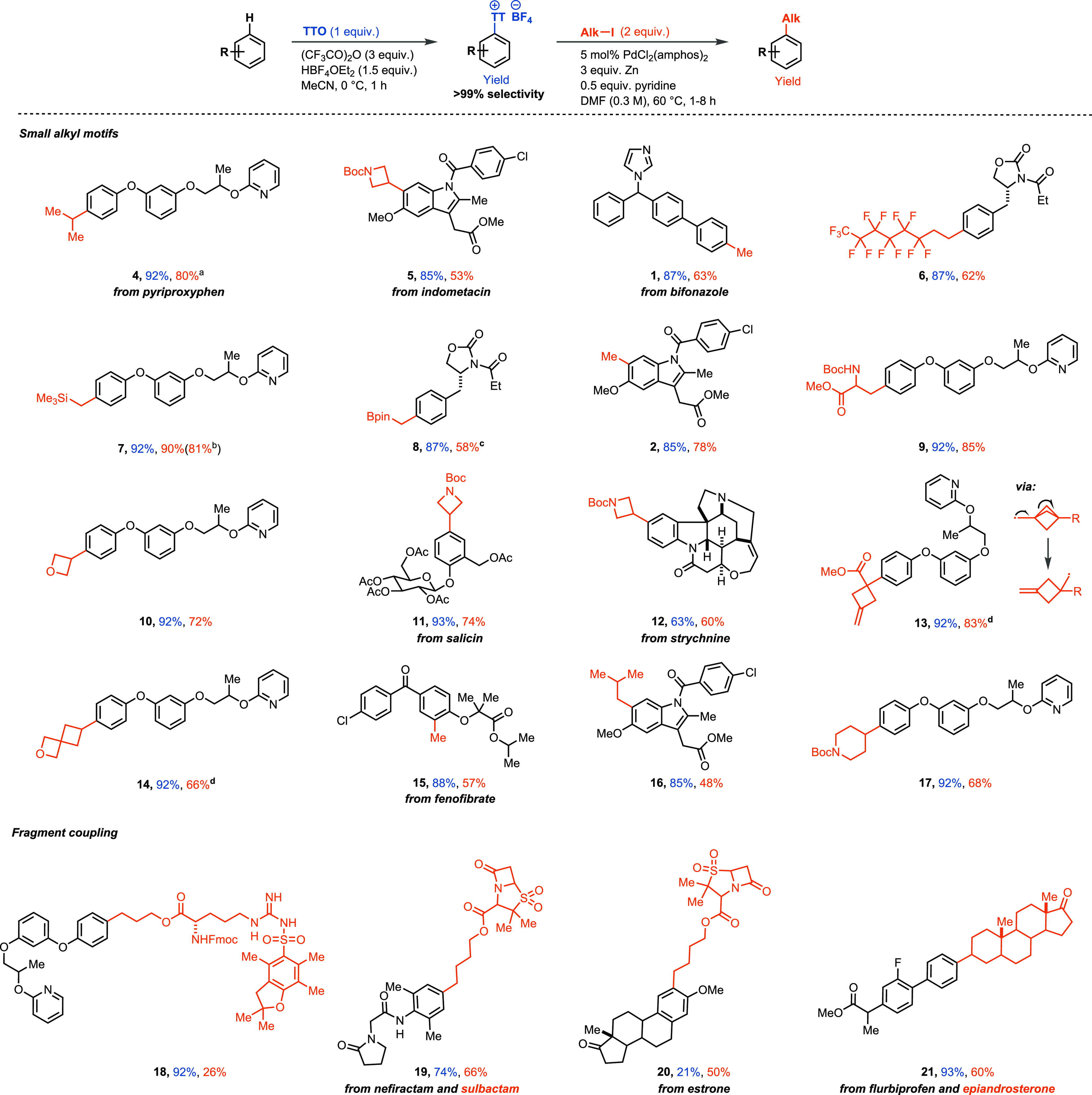
Substrate Scope for the Alkylation of Aryl Thianthrenium Salt General conditions unless otherwise noted: aryl thianthrenium salt (0.3 mmol), alkyl iodide (0.6 mmol), PdCl_2_(amphos)_2_ (15.0 μmol), pyridine (0.15 mmol), DMF (0.3 M). ^a^>20:1 ratio of *i-*PrAr:*n-*PrAr product. ^b^3.0 mmol scale. ^c^Pyridine was omitted. ^d^Reactions carried with aryl thianthrenium salt (0.2 mmol) and MgCl_2_ (3 equiv) as additive. Yields in blue correspond to yield of C–H thianthrenation. Yields in orange correspond to yield of alkylation of aryl thianthrenium salts. Yields of thianthrenation were obtained from refs (5), (6a), (6b), (6c), (6d), and (6e).

A plausible mechanism hypothesis for this transformation is depicted in Scheme 2d. Though most reductive electrophile cross-coupling reactions with alkyl halides proceed via a radical chain process,^4d,10a,15b,23^ we believe an oxidative addition-transmetalation-reductive elimination sequence is operative in this transformation. Control experiments showed that, in the presence of zinc and absence of a palladium-catalyst, no cleavage of aryl thianthrenium salts occurred on a time scale compatible with the reductive cross-coupling (Scheme 2a). Instead, the alkyl iodide was hydrodehalogenated with 90% conversion in <5 min (see the Supporting Information). On the basis of redox potentials, aryl thianthrenium salts (*E*(PhTT^+^/PhTT^•^) = −1.5 V vs SCE)^6c^ cannot be reduced by zinc (*E*(Zn^2+^/Zn_(s)_) = −0.76 V vs SCE). Though unactivated alkyl iodides (*E*(*n*-BuI/BuI^•^) = −2.5 V vs SCE)^24^ are even more difficult to reduce then aryl thianthrenium salts, oxidative addition of zinc is known to proceed via an inner sphere electron transfer involving a bridging ligand.^25^ Because such a process is more feasible on the alkyl iodide than on the aryl thianthrenium salt,^25^ selective radical mediated oxidative addition of zinc into the alkyl iodide could take place. Furthermore, since zinc cannot be replaced by an organic reductant, tetrakis(dimethylamino)ethylene (see Table 1), we postulate the intermediacy of an alkylzinc species under our reaction conditions. A radical clock experiment was conducted with **TT-2** as a mechanistic probe to distinguish between a concerted oxidative addition and a pathway which involves single electron transfer.^6c,26^ The observation of noncyclized product **22** in the aryl alkylation of allyl ether thianthrenium salt **TT-2** under standard conditions is consistent with a concerted oxidative addition mechanism (Scheme 2b). The aryl 2,2,6,6-tetramethylpiperidin-1-oxyl (TEMPO) adduct was not observed for the aryl alkylation upon addition of TEMPO, which is consistent with the absence of aryl radicals (Scheme 2c). By using amphos, the rate of ß-hydride elimination is slower relative to the rate of reductive elimination, which is consistent with the different product distributions of *n-*PrAr:*i-*PrAr when different catalysts are used (see discussion above). The faster reductive elimination from complexes with bulky monodentate ligands instead of bidentate ligands is in agreement with a T-shaped intermediate from which reductive elimination is faster than from a four-coordinate square planar intermediate.^27^ Though the preliminary mechanistic data presented above is consistent with an oxidative addition–transmetalation–reductive elimination sequence, we cannot exclude a single electron transfer mechanism.

In conclusion, we present a method for the site-selective alkylation of aryl thianthrenium salts via a two-step C–H functionalization/reductive alkylation sequence that grants access to alkylated arenes that cannot be obtained with comparable selectivities by other, undirected aryl C–H alkylation methods. By forming the zinc reagent *in situ*, we bypass the need to preform an organometallic reagent prior to the cross-coupling event, which from a synthetic point of view and in terms of practicality provides an advantage to other, related Negishi-type aryl alkylations, including our previously reported selective aryl alkylations that only work with selected alkylzinc reagents.^6a,7g^ The excellent site-selectivity and robust reactivity enable us to engage complex fragments, which could be of value in medicinal chemistry. We believe this work represents a valuable conceptual extension to existing reductive Csp^2^–Csp^3^ cross-coupling reactions with improved efficiency, reactivity, and synthetic utility.

## Supporting Information Available

Experimental procedures and NMR spectra (PDF)

## References

[ref1] aWalters W. P.; Green J.; Weiss J. R.; Murcko M. A. What do medicinal chemists actually make? A 50-year retrospective. J. Med. Chem. 2011, 54, 6405–6416. 10.1021/jm200504p. 21755928

[ref2] aDong Z.; Ren Z.; Thompson S. J.; Xu Y.; Dong G. Transition-metal-catalyzed C–H alkylation using alkenes. Chem. Rev. 2017, 117, 9333–9403. 10.1021/acs.chemrev.6b00574. 28125210

[ref3] Hartwig J. F. Evolution of C–H bond functionalization from methane to methodology. J. Am. Chem. Soc. 2016, 138, 2–24. 10.1021/jacs.5b08707. 26566092 PMC4809212

[ref4] aJana R.; Pathak T. P.; Sigman M. S. Advances in transition metal (Pd, Ni, Fe)-catalyzed cross-coupling reactions using alkyl-organometallics as reaction partners. Chem. Rev. 2011, 111, 1417–1492. 10.1021/cr100327p. 21319862 PMC3075866

[ref5] Tang R.-J.; Milcent T.; Crousse B. Regioselective halogenation of arenes and heterocycles in hexafluoroisopropanol. J. Org. Chem. 2018, 83, 930–938. 10.1021/acs.joc.7b02920. 29256248

[ref6] aBerger F.; Plutschack M. B.; Riegger J.; Yu W.; Speicher S.; Ho M.; Frank N.; Ritter T. Site-selective and versatile aromatic C–H functionalization by thianthrenation. Nature 2019, 567, 223–228. 10.1038/s41586-019-0982-0. 30867606

[ref7] aAtwater B.; Chandrasoma N.; Mitchell D.; Rodriguez M. J.; Organ M. G. Pd-PEPPSI-IHeptCl: A General-Purpose, Highly Reactive Catalyst for the Selective Coupling of Secondary Alkyl Organozincs. Chem. - Eur. J. 2016, 22, 14531–14534. 10.1002/chem.201603603. 27481602

[ref8] Friedel C.; Crafts J. Organic chemistry. J. Chem. Soc. 1877, 32, 725–791. 10.1039/js8773200725.

[ref9] aRudolph A.; Lautens M. Secondary Alkyl Halides in Transition-Metal-Catalyzed Cross-Coupling Reactions. Angew. Chem., Int. Ed. 2009, 48, 2656–2670. 10.1002/anie.200803611. 19173365

[ref10] aEverson D. A.; Jones B. A.; Weix D. J. Replacing conventional carbon nucleophiles with electrophiles: nickel-catalyzed reductive alkylation of aryl bromides and chlorides. J. Am. Chem. Soc. 2012, 134, 6146–6159. 10.1021/ja301769r. 22463689 PMC3324882

[ref11] aMolander G. A.; Traister K. M.; O’Neill B. T. Reductive cross-coupling of nonaromatic, heterocyclic bromides with aryl and heteroaryl bromides. J. Org. Chem. 2014, 79, 5771–5780. 10.1021/jo500905m. 24892751

[ref12] aLiu J.; Gong H. Stereoselective preparation of α-C-vinyl/aryl glycosides via nickel-catalyzed reductive coupling of glycosyl halides with vinyl and aryl halides. Org. Lett. 2018, 20, 7991–7995. 10.1021/acs.orglett.8b03567. 30525666

[ref13] aZhang P.; Le C. C.; MacMillan D. W. Silyl radical activation of alkyl halides in metallaphotoredox catalysis: a unique pathway for cross-electrophile coupling. J. Am. Chem. Soc. 2016, 138, 8084–8087. 10.1021/jacs.6b04818. 27263662 PMC5103281

[ref14] aCzaplik W. M.; Mayer M. Jacobi von Wangelin, A. Domino Iron Catalysis: Direct Aryl–Alkyl Cross-Coupling. Angew. Chem., Int. Ed. 2009, 48, 607–610. 10.1002/anie.200804434. 19067450

[ref15] aWang X.; Dai Y.; Gong H. Nickel-catalyzed reductive couplings. Top. Curr. Chem. 2017, 61–89. 10.1007/978-3-319-49784-6_3. 27573395

[ref16] aMajid T. N.; Knochel P. A new preparation of highly functionaized aromatic and heteroaromatic zinc and copper organometallics. Tetrahedron Lett. 1990, 31, 4413–4416. 10.1016/S0040-4039(00)97635-4.

[ref17] Hayashi T.; Konishi M.; Kobori Y.; Kumada M.; Higuchi T.; Hirotsu K. Dichloro[1,1’-bis (diphenylphosphino)ferrocene]palladium (II): an effective catalyst for cross-coupling of secondary and primary alkyl Grignard and alkylzinc reagents with organic halides. J. Am. Chem. Soc. 1984, 106, 158–163. 10.1021/ja00313a032.

[ref18] Yang Y.; Niedermann K.; Han C.; Buchwald S. L. Highly selective palladium-catalyzed cross-coupling of secondary alkylzinc reagents with heteroaryl halides. Org. Lett. 2014, 16, 4638–4641. 10.1021/ol502230p. 25153332 PMC4156254

[ref19] Dai C.; Fu G. C. The first general method for palladium-catalyzed Negishi cross-coupling of aryl and vinyl chlorides: use of commercially available Pd (P(t-Bu)_3_)_2_ as a catalyst. J. Am. Chem. Soc. 2001, 123, 2719–2724. 10.1021/ja003954y. 11456957

[ref20] aManolikakes G.; Schade M. A.; Hernandez C. M.; Mayr H.; Knochel P. Negishi cross-couplings of unsaturated halides bearing relatively acidic hydrogen atoms with organozinc reagents. Org. Lett. 2008, 10, 2765–2768. 10.1021/ol8009013. 18529011

[ref21] aMarsilje T. H.; Pei W.; Chen B.; Lu W.; Uno T.; Jin Y.; Jiang T.; Kim S.; Li N.; Warmuth M. Synthesis, structure–activity relationships, and in vivo efficacy of the novel potent and selective anaplastic lymphoma kinase (ALK) inhibitor 5-Chloro-N 2-(2-isopropoxy-5-methyl-4-(piperidin-4-yl) phenyl)-N 4-(2-(isopropylsulfonyl) phenyl) pyrimidine-2, 4-diamine (LDK378) currently in phase 1 and phase 2 clinical trials. J. Med. Chem. 2013, 56, 5675–5690. 10.1021/jm400402q. 23742252

[ref22] Vitaku E.; Smith D. T.; Njardarson J. T. Analysis of the structural diversity, substitution patterns, and frequency of nitrogen heterocycles among US FDA approved pharmaceuticals: miniperspective. J. Med. Chem. 2014, 57, 10257–10274. 10.1021/jm501100b. 25255204

[ref23] aEverson D. A.; Weix D. J. Cross-electrophile coupling: principles of reactivity and selectivity. J. Org. Chem. 2014, 79, 4793–4798. 10.1021/jo500507s. 24820397 PMC4049235

[ref24] Shen Y.; Cornella J.; Juliá-Hernández F.; Martin R. Visible-light-promoted atom transfer radical cyclization of unactivated alkyl iodides. ACS Catal. 2017, 7, 409–412. 10.1021/acscatal.6b03205.

[ref25] Guijarro A.; Rosenberg D. M.; Rieke R. D. The reaction of active zinc with organic bromides. J. Am. Chem. Soc. 1999, 121, 4155–4167. 10.1021/ja9844478.

[ref26] Creutz S. E.; Lotito K. J.; Fu G. C. Peters, Photoinduced Ullmann C–N coupling: demonstrating the viability of a radical pathway. Science 2012, 338, 647–651. 10.1126/science.1226458. 23118186

[ref27] Tatsumi K.; Hoffmann R.; Yamamoto A.; Stille J. K. Reductive elimination of d8-organotransition metal complexes. Bull. Chem. Soc. Jpn. 1981, 54, 1857–1867. 10.1246/bcsj.54.1857.

