# Correction to “Site-Selective C–H Alkylation of Complex Arenes by a Two-Step Aryl Thianthrenation-Reductive Alkylation Sequence”

**DOI:** 10.1021/jacs.1c06057

**Published:** 2021-07-01

**Authors:** Beatrice Lansbergen, Paola Granatino, Tobias Ritter

Page 7912. In our previous Communication, we inadvertently drew the substrates derived from the molecule pyriproxyfen with incorrect connectivity of the pyriproxyfen molecule (*meta* instead of *para* was drawn), omitted a methylene group from compound **13**, and drew an epimer of compound **20** in Scheme 3. These drawing errors have been corrected in the corrected Scheme 3 shown here.

**Scheme 3:**
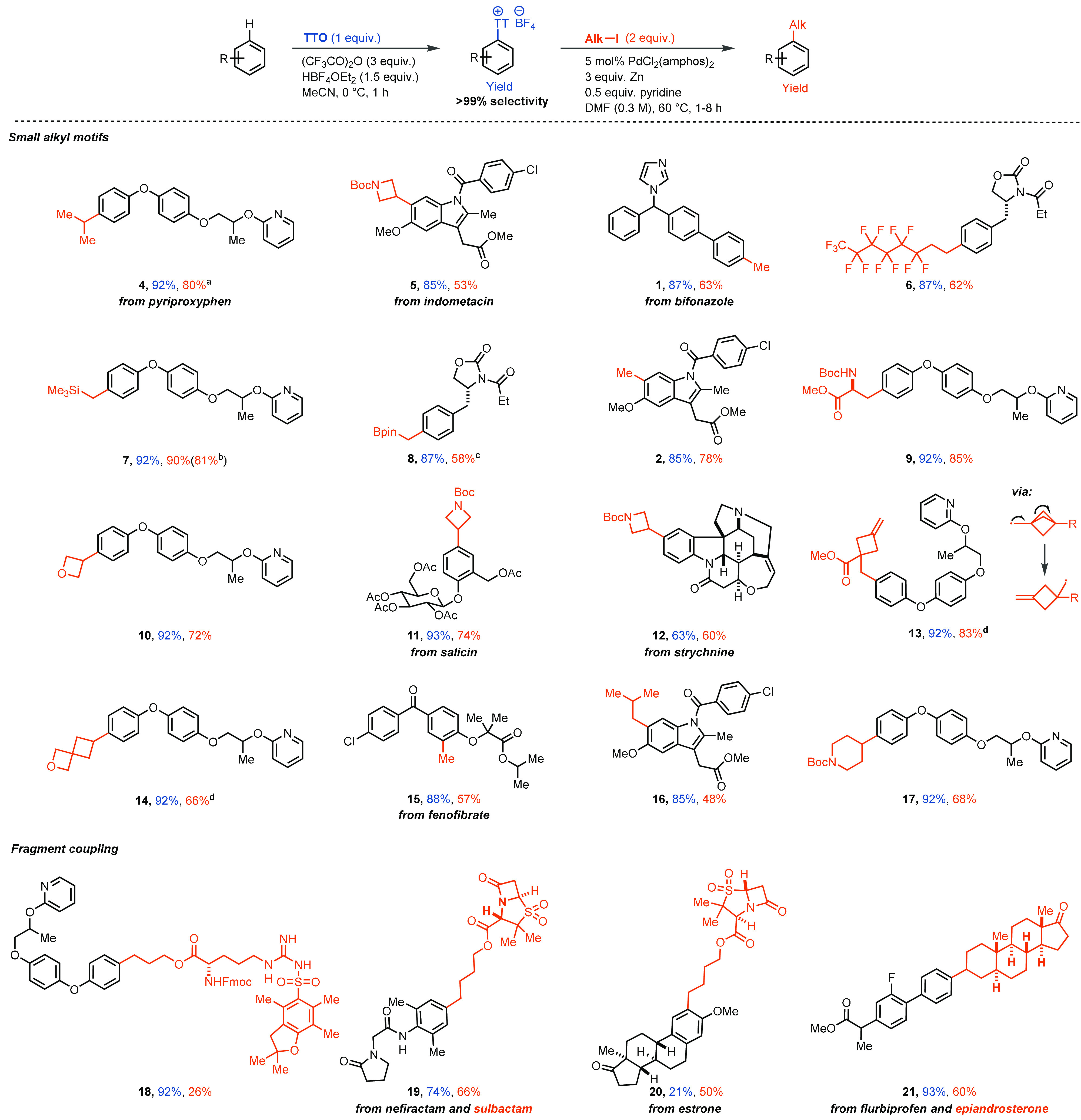
Substrate Scope for the Alkylation of Aryl Thianthrenium Salt General conditions unless otherwise noted: aryl thianthrenium salt (0.3 mmol), alkyl iodide (0.6 mmol), PdCl_2_(amphos)_2_ (15.0 μmol), pyridine (0.15 mmol), DMF (0.3 M). [a]>20:1 ratio of *i-*PrAr:*n-*PrAr product. [b]3.0 mmol scale. [c]Pyridine was omitted. [d]Reactions carried with aryl thianthrenium salt (0.2 mmol) and MgCl_2_ (3 equiv) as additive. Yields in blue correspond to yield of C–H thianthrenation. Yields in orange correspond to yield of alkylation of aryl thianthrenium salts. Yields of thianthrenation were obtained from refs 5b, 6a, 6b, 6c, 6d, and 6e.

As a further clarification, we have commented in the revised Supporting Information about the undefined stereocenter of compound **21**.

The findings and conclusions of the original communication remain unchanged. We apologize for the errors and for any inconvenience that this may have caused the readers of *JACS*.

## Supporting Information Available

Experimental procedures and spectral data (revised) (PDF)

