# Supplementary material for: Site-Selective C–H alkylation of Complex Arenes by a Two-Step Aryl Thianthrenation-Reductive Alkylation Sequence
Source: J Am Chem Soc. 2021 May 24;143(21):7909–14. doi: 10.1021/jacs.1c03459 (PMC8297726; doi:10.1021/jacs.1c03459)
Supplement: Supplementary file 1 — ja1c03459_si_001.pdf [file ja1c03459_si_001.pdf]

SUPPORTING INFORMATION

Site-selective C–H alkylation of complex arenes by a two-step aryl thianthrenation-reductive alkylation sequence

Beatrice Lansbergen, Paola Granatino, Tobias Ritter*

Max-Planck-Institut für Kohlenforschung

Kaiser-Wilhelm-Platz 1, D-45470 Mülheim an der Ruhr, Germany

*E-mail: ritter@mpi-muelheim.mpg.de

TABLE OF CONTENTS

TABLE OF CONTENTS 2

MATERIALS AND METHODS 7

EXPERIMENTAL DATA 8

General procedure for the thianthrenation of arenes 8

General procedure for the thianthrenation of arenes[2] 8

General procedure for the alkylation of aryl thianthrenium salts 8

General procedure for the alkylation of aryl thianthrenium salts using a Schlenk line 8

General procedure for the alkylation of aryl thianthrenium salts using a glovebox 9

General procedure for the activation of zinc dust 10

General procedure for the activation of zinc dust 10

Reaction optimization of the alkylation of aryl thianthrenium salts 10

General Procedure for optimization of reaction conditions 10

Table S1: Optimization of yield as a function of catalyst 10

Table S2: Selectivity for *i*-PrAr versus *n*-PrAr 11

Table S3: Optimization of yield as a function of solvent 15

Table S4: Optimization of yield as a function of reducing agent 15

Table S5: Optimization of yield as a function of an additive 15

General Procedure for the evaluation of a “one-pot” process 16

Table S6: Evaluation of a “one-pot” process 17

Synthesis of aryl thianthrenium salts 18

Estrone methyl ether thianthrenium salt derivative **TT-4** 18

Synthesis of alkyl iodides 19

Sulbactam iodide derivative **S-2** 19

Fmoc-Arg(Pbf)-OH iodide derivative **S-3** 19

3-Iodo epiandrosterone **S-4** 20

Alkylation of aryl thianthrenium salts with alkyl iodides 21

Methyl bifonazole derivative **1** 21

Methyl indomethacin methyl ester derivative **2** 22

Isopropyl pyriproxyfen derivative **4** 23

n-Propyl pyriproxyfen derivative **S-1** 23

Boc-azetidinyl indomethacin methyl ester derivative **5** 24

Tridecafluorooctyl benzyloxazolidinone derivative **6** 25

(Methyl)trimethylsilyl pyriproxyfen derivative **7** 26
Methyl bis(pinacolato)diboron benzyloxazolidinone derivative **8** 27
Analine-*N*-boc methyl ester pyriproxyfen derivative **9** 28
Oxetanyl pyriproxyfen derivative **10** 29
Boc-azetidinyl salicin pentaacetate derivative **11** 30
Boc-azetidinyl strychnine derivative **12** 31
Methylene cyclobutane carboxylate pyriproxyfen derivative **13** 32
Oxaspiro[3.3]heptanyl pyriproxyfen derivative **14** 33
Methyl fenofibrate derivative **15** 34
2-Methylpropanyl indomethacin methyl ester derivative **16** 34
Boc-piperidinyl pyriproxyfen derivative **17** 35
Fmoc-Arg(Pbf)-OH pyriproxyfen derivative **18** 36
Sulbactam nefiractam derivative **19** 37
Sulbactam estrone methyl ether derivative **20** 38
Epiandrosterone flurbiprofen methyl ester derivative **21** 39
Boc-azetidinyl chlorobenzene derivative **S-5** 40

Alkylation of aryl thianthrenium salts with alkyl triflate 41

Methyl pyriproxyfen derivative **S-6** 41

Alkylation of aryl thianthrenium salts with alkyl bromides 42

Fluoro butyl pyriproxyfen derivative **S-7** 42
Oxetanyl pyriproxyfen derivative **10** 43

Mechanistic investigation 43

Radical clock experiment 43
Boc-piperidinyl diphenyl ether derivative 3 in the absence of TEMPO 44
Boc-piperidinyl diphenyl ether derivative 3 in the presence of TEMPO 45
Radical clock starting material allyl ether thianthrenium salt **TT-2** 46
Radical clock cyclization experiment 47
Control experiments to probe for the reduction by zinc 48

Palladium-catalyzed aryl C–H alkylation via bromination versus thianthrenation 49

General procedures for the bromination of arenes using 3 different methods 49
Table S7: Position of thianthrenation and bromination of bifonazole and indomethacin methyl ester 49
Procedures for the bromination of bifonazole using 3 different methods 50
Determination of selectivity of bromination and subsequent alkylation: bifonazole 51
^{1}H NMR analysis for the selective thianthrenation of bifonazole[2, 4] 54
Palladium-catalyzed alkylation of **S-10** 54
Summary of results for the methylation of bifonazole via bromination vs. thianthrenation 55

Procedures for the bromination of indomethacin methyl ester using 3 different methods....55
Determination of selectivity of bromination and subsequent alkylation: indomethacin methyl ester ...56
^{1}H NMR analysis for the selective thianthrenation of indomethacin methyl ester[2, 4]....60
Palladium-catalyzed alkylation of **S-11** 60
Summary of results of the methylation of indomethacin methyl ester via bromination vs. thianthrenation....61

SPECTROSCOPIC DATA....62
^{1}H NMR of estrone methyl ether thianthrenium salt derivative **TT-4**....62
^{13}C NMR of estrone methyl ether thianthrenium salt derivative **TT-4** 63
^{19}F NMR of estrone methyl ether thianthrenium salt derivative **TT-4**....64
^{1}H NMR of sulbactam iodide derivative **S-2** 65
^{13}C NMR of sulbactam iodide derivative **S-2**....66
^{1}H NMR of Fmoc-Arg(Pbf)-OH iodide derivative **S-3** 67
^{13}C NMR of Fmoc-Arg(Pbf)-OH iodide derivative **S-3**....68
^{1}H NMR of 3-iodo epiandrosterone derivative **S-4** 69
^{13}C NMR of 3-iodo epiandrosterone derivative **S-4**....70
^{1}H NMR of methyl bifonazole derivative **1**....71
^{13}C NMR of methyl bifonazole derivative **1** 72
^{1}H NMR of methyl indomethacin methyl ester derivative **2** 73
^{13}C NMR of methyl indomethacin methyl ester derivative **2**....74
^{1}H NMR of isopropyl pyriproxyfen derivative **4**....75
^{13}C NMR of isopropyl pyriproxyfen derivative **4** 76
^{1}H NMR of *n*-propyl pyriproxyfen derivative **S-1** 77
^{13}C NMR of *n*-propyl pyriproxyfen derivative **S-1** 78
^{1}H NMR of boc-azetidinyl indomethacin methyl ester derivative **5** 79
^{13}C NMR of boc-azetidinyl indomethacin methyl ester derivative **5** 80
^{1}H NMR of tridecafluorooctyl benzyloxazolidinone derivative **6**....81
^{13}C NMR of tridecafluorooctyl benzyloxazolidinone derivative **6**....82
^{19}F NMR of tridecafluorooctyl benzyloxazolidinone derivative **6** 83
^{1}H NMR of (methyl)trimethylsilyl pyriproxyfen derivative **7** 84

^{13}C NMR of (methyl)trimethylsilyl pyriproxyfen derivative **7** 85

^{1}H NMR of methyl bis(pinacolato)diboron derivative **8** 86

^{13}C NMR of methyl bis(pinacolato)diboron derivative **8** 87

^{1}H NMR of alanine-*N*-boc-methyl ester pyriproxyfen derivative **9** 88

^{13}C NMR of alanine-*N*-boc-methyl ester pyriproxyfen derivative **9** 89

^{1}H NMR of oxetanyl pyriproxyfen derivative **10** 90

^{13}C NMR of oxetanyl pyriproxyfen derivative **10** 91

^{1}H NMR of boc-azetidinyl salicin pentaacetate derivative **11** 92

^{13}C NMR of boc-azetidinyl salicin pentaacetate derivative **11** 93

^{1}H NMR of boc-azetidinyl strychnine derivative **12** 94

^{13}C NMR of boc-azetidinyl strychnine derivative **12** 95

^{1}H NMR of methylene cyclobutane carboxylate pyriproxyfen derivative **13** 96

^{13}C NMR of methylene cyclobutane carboxylate pyriproxyfen derivative **13** 97

HSQC of methylene cyclobutane carboxylate pyriproxyfen derivative **13** 98

HMBC of methylene cyclobutane carboxylate pyriproxyfen derivative **13** 99

COSY of methylene cyclobutane carboxylate pyriproxyfen derivative **13** 100

^{1}H NMR of oxaspiro[3.3]heptanyl pyriproxyfen derivative **14** 101

^{13}C NMR of oxaspiro[3.3]heptanyl pyriproxyfen derivative **14** 102

^{1}H NMR of methyl fenofibrate derivative **15** 103

^{13}C NMR of methyl fenofibrate derivative **15** 104

^{1}H NMR of 2-methylpropanyl indomethacin methyl ester derivative **16** 105

^{13}C NMR of 2-methylpropanyl indomethacin methyl ester derivative **16** 106

^{1}H NMR of boc-piperidinyl pyriproxyfen derivative **17** 107

^{13}C NMR of boc-piperidinyl pyriproxyfen derivative **17** 108

^{1}H NMR of Fmoc-Arg(Pbf)-OH pyriproxyfen derivative **18** 109

^{13}C NMR of Fmoc-Arg(Pbf)-OH pyriproxyfen derivative **18** 110

^{1}H NMR of nefiractam sulbactam derivative **19** 111

^{13}C NMR of nefiractam sulbactam derivative **19** 112

^{1}H NMR of sulbactam estrone methyl ether derivative **20** 113

^{13}C NMR of sulbactam estrone methyl ether derivative **20** 114

^{1}H NMR epiandrosterone flurbiprofen methyl ester derivative **21** 115

^{13}C NMR epiandrosterone flurbiprofen methyl ester derivative **21** 116

^{19}F NMR epiandrosterone flurbiprofen methyl ester derivative **21** 117

^{1}H NMR methyl 4-hydroxy-3-isopropylbenzoate **22**, radical clock cyclization experiment 118

^{13}C NMR methyl 4-hydroxy-3-isopropylbenzoate **22**, radical clock cyclization experiment 119

^{1}H NMR methyl 4-hydroxybenzoate **23**, radical clock cyclization experiment 120

^{13}C NMR methyl 4-hydroxybenzoate **23**, radical clock cyclization experiment 121

^{1}H NMR of boc-azetidinyl chlorobenzene derivative **S-5** 122

^{13}C NMR of boc-azetidinyl chlorobenzene derivative **S-5** 123

^{1}H NMR of methyl pyriproxyfen derivative **S-6** 124

^{13}C NMR of methyl pyriproxyfen derivative **S-6** 125

^{1}H NMR of fluoro butyl pyriproxyfen derivative **S-7** 126

^{13}C NMR of fluoro butyl pyriproxyfen derivative **S-7** 127

^{19}F NMR of fluoro butyl pyriproxyfen derivative **S-7** 128

^{1}H NMR of butenyl pyriproxyfen derivative **S-8** + **S-9** 129

^{13}C NMR of butenyl pyriproxyfen derivative **S-8** + **S-9** 130

^{1}H NMR of boc-piperidinyl diphenyl ether derivative **3** 131

^{13}C NMR of boc-piperidinyl diphenyl ether derivative **3** 132

^{1}H NMR of radical clock starting material allyl ether thianthrenium salt **TT-2** 133

^{13}C NMR of radical clock starting material allyl ether thianthrenium salt **TT-2** 134

^{19}F NMR of radical clock starting material allyl ether thianthrenium salt **TT-2** 135

REFERENCES 136

MATERIALS AND METHODS

All air and moisture sensitive manipulations were performed using standard Schlenk techniques or glovebox techniques under an atmosphere of argon or nitrogen. High-resolution mass spectra were obtained using *Q Exactive Plus* from *Thermo*. Concentration under reduced pressure was performed by rotary evaporation at 25–40 °C at an appropriate pressure. Yields refer to purified and spectroscopically pure compounds, unless otherwise stated.

Solvents

DMF was purchased from *Sigma-Aldrich* and dried with 4Å molecular sieves. All deuterated solvents were purchased from Eur*iso*-Top.

Chromatography

Thin layer chromatography (TLC) was performed using EMD TLC plates pre-coated with 250 μm thickness silica gel 60 F_{254} plates and visualized by fluorescence quenching under UV light. Flash column chromatography was performed using silica gel (40–63 µm particle size) purchased from Geduran. Preparatory high-performance liquid chromatographic separation was executed on a Shimadzu Prominence Preparative HPLC system with an YMC Pack Pro column or a Triart C18 HPLC column.

Spectroscopy and Instruments

NMR spectra were recorded on a Bruker Ascend™ 500 spectrometer operating at 500 MHz, 471 MHz, and 126 MHz for ^{1}H, ^{19}F, and ^{13}C acquisitions, respectively, a Bruker UltraShield™ 300 spectrometer operating at 300 MHz, 282 MHz, and 75 MHz for ^{1}H, ^{19}F, and ^{13}C acquisitions, respectively, or a Bruker AV600 spectrometer operating at 600 MHz and 150 MHz for ^{1}H and ^{13}C acquisitions. Chemical shifts are reported in ppm with the solvent residual peak as the internal standard. For ^{1}H NMR: $CDCl_3$, δ 7.26; CD_3OD, δ 3.31; $(CD_3)_2SO$, δ 2.50; CD_3CN, δ 1.94, CD_2Cl_2, δ 5.32. For ^{13}C NMR: $CDCl_3$, δ 77.16; CD_3OD, δ 49.00; $(CD_3)_2SO$, δ 39.52; CD_3CN, δ 1.32, CD_2Cl_2, δ 53.84.[1] Data is reported as follows: s = singlet, d = doublet, t = triplet, q = quartet, m = multiplet, br = broad; coupling constants in Hz; integration.

Starting materials

All substrates were used as received from commercial suppliers. Alkyl iodides were purchased from *Sigma-Aldrich*, *Chempur*, *SpiroChem*, or *Alfa Aesar.* Aryl thianthrenium salts **TT-1**, **TT-2’**, **TT-3**, **TT-5**, **TT-6**, **TT-7**, **TT-8. TT-9, TT-10, TT-11, TT-12, TT-13** and thianthrene-*S*-oxide (**TTO**) were prepared according to the literature.[2-7]

EXPERIMENTAL DATA

General procedure for the thianthrenation of arenes

General procedure for the thianthrenation of arenes[2]

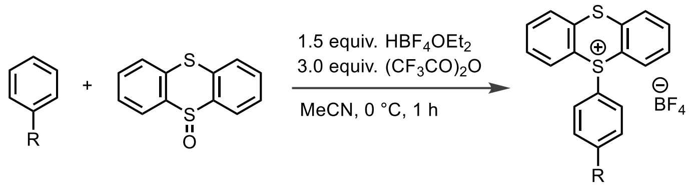

Under an ambient atmosphere, a 20 mL glass vial was charged with arene (0.50 mmol, 1.0 equiv.) and dry MeCN (2.0 – 4.0 mL, c = 0.13 – 0.25 M). After cooling to 0 °C, $HBF_4{\cdot}OEt_2$ (1.2 equiv. + 1.0 equiv. per basic functional group) was added to the vial while stirring the reaction mixture. Other acids may be used instead of $HBF_4{\cdot}OEt_2$ like triflic acid (TfOH). For acid sensitive substrates $BF_3{\cdot}OEt_2$ or trimethylsilyltriflate (TMSOTf) can be used as well. After all solids were dissolved, thianthrenium-*S*-oxide **(TTO)** (0.50 mmol, 1.0 equiv.) was added in one portion to the solution at 0 °C, leading to a suspension. Subsequently, trifluorocetic anhydride (1.5 mmol, 3.0 equiv.) was added in one portion at 0 °C, resulting in a color change to deep purple. The vial was sealed with a screw-cap. The mixture was stirred at 0 °C for 1 h, subsequently the reaction mixture was warmed to 25 °C and stirred until all solids dissolved, and the intensity of the purple color decreased. The solution was diluted with 5 mL dichloromethane and poured onto a mixture of 30 mL dichloromethane, 20 mL saturated aqueous Na_2CO_3 solution, and 10 mL water. After stirring for 5 min at 25 °C, the mixture was poured into a separatory funnel, and the layers were separated. The dichloromethane layer was washed with aqueous $NaBF_4$ solution (2 × ca. 20 mL, 5 % w/w) and with water (2 × ca. 20 mL). Washing with $NaBF_4$ solution is only required if it is of interest that the product contains only one type of counterion, solutions containing other ions, like triflate or hexafluorophosphate may be used as well. The dichloromethane layer was dried over $MgSO_4$, filtered, and the solvent was removed under reduced pressure. In order to obtain analytically pure samples of thianthrenium salts, the residue was purified by chromatography on silica gel eluting with dichloromethane / *i*-PrOH, subsequently, the product was dissolved in 2 mL dichloromethane and precipitated with 20 mL Et_2O. The solid was dried in vacuo to afford the thianthrenium salt.

General procedure for the alkylation of aryl thianthrenium salts

General procedure for the alkylation of aryl thianthrenium salts using a Schlenk line

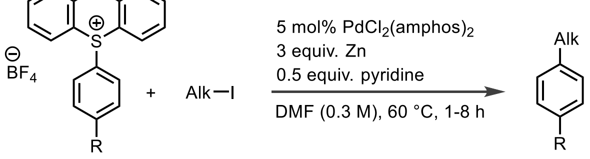

To an oven-dried 5 mL Schlenk finger under argon atmosphere containing a Teflon-coated magnetic stirring bar were added thianthrenium salt (0.3 mmol, 1 equiv.), $PdCl_2(amphos)_2$ (10.6 mg, 15.0 µmol, 5.00 mol%),

and activated zinc powder 100-mesh 99.5% (58.9 mg, 0.900 mmol, 3.00 equiv.). The Schlenk finger was sealed with a septum stopper. The Schlenk finger was evacuated and backfilled with argon. Dry DMF (1 mL, c = 0.3 M) was added to the solids. Subsequently, alkyl iodide (0.6 mmol, 2 equiv.) and pyridine (12 µL, 12 mg, 0.15 mmol, 0.50 equiv.) were added at 25 °C via a syringe. The Schlenk finger was placed in an oil bath preheated at 60 °C where the reaction mixture was stirred rigorously (850 rpm) for 1–8 h. A color change from yellow to dark brown was observed. The mixture was cooled to 25 °C, subsequently diluted with ethyl acetate (40 mL), and thereafter poured into a separatory funnel. The organic layer was washed with water (1 × 40 mL). The aqueous layer was then extracted with ethyl acetate (3 × 40 mL). The organic layers were combined, dried over $MgSO_4$, filtered, and concentrated under reduced pressure. The resulting residue was purified by column chromatography on silica gel to afford the desired alkylation product.

Note: When alkylation of aryl thianthrenium salts was carried out with alkyl bromides or alkyl triflates, ZnI_2 (0.5 equiv.) was added, and the reaction mixture was stirred at 80 °C for 12 h.

General procedure for the alkylation of aryl thianthrenium salts using a glovebox

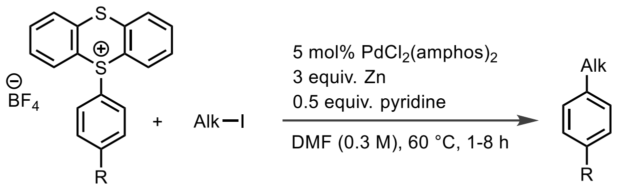

To a 4-mL borosilicate vial containing a Teflon-coated magnetic stirring bar were added thianthrenium salt (0.3 mmol, 1 equiv.), $PdCl_2(amphos)_2$ (10.6 mg, 15.0 µmol, 5.00 mol%), and activated zinc powder 100-mesh 99.5% (58.9 mg, 0.900 mmol, 3.00 equiv.). The vial was transferred into a nitrogen-filled glovebox. Dry DMF (1 mL, c = 0.3 M) was added to the solids. Subsequently, alkyl iodide (0.6 mmol, 2 equiv.) and pyridine (12 µL, 12 mg, 0.15 mmol, 0.50 equiv.) were added at 25 °C. The vial was sealed with a Teflon-lined screw cap, removed from the glovebox, and transferred to a heating block preheated at 60 °C where the reaction mixture was stirred rigorously (850 rpm) for 1–8 h. A color change from yellow to dark brown was observed. The mixture was cooled to 25 °C, subsequently diluted with ethyl acetate (40 mL), and thereafter poured into a separatory funnel. The organic layer was washed with water (1 × 40 mL). The aqueous layer was then extracted with ethyl acetate (3 × 40 mL). The organic layers were combined, dried over $MgSO_4$, filtered, and concentrated under reduced pressure. The resulting residue was purified by column chromatography on silica gel to afford the desired alkylation product.

Note: For convenience and efficiency, the alkylation reactions were conducted in the glovebox unless stated otherwise. No change in yield was found when the alkylation reactions were carried out using a Schlenk line or in the glovebox.

Note: When alkylation of aryl thianthrenium salts was carried out with alkyl bromides or alkyl triflates, ZnI_2 (0.5 equiv.) was added, and the reaction mixture was stirred at 80 °C for 12 h.

General procedure for the activation of zinc dust

General procedure for the activation of zinc dust

To a 20 mL glass vial containing a Teflon-coated magnetic stirring bar was added zinc powder 100-mesh 99.5% (500 mg, 7.64 mmol, 1.00 equiv.) followed by 5 mL saturated aqueous NH_4Cl solution. The mixture was stirred at 500 rpm for 30 minutes. The reaction mixture was decanted, and the activated zinc was washed with water (2 × 2 mL), acetone (1 × 2 mL), ethanol (1 × 2 mL), DMF (1 × 2 mL), and diethyl ether (1 × 2 mL). The washing was carried out quickly to minimize exposure of the activated zinc to air. The solid was then dried under vacuum for 1 h.

Note: In order to obtain highest yields in the subsequent alkylation reaction, the zinc activation was carried out prior to the alkylation reaction. Storage in the glovebox over a period of more than 3 days reduced the activity of the activated zinc.

Reaction optimization of the alkylation of aryl thianthrenium salts

General Procedure for optimization of reaction conditions

To a 4-mL borosilicate vial containing a Teflon-coated magnetic stirring bar were added thianthrenium salt (0.1 mmol, 1 equiv.), $PdCl_2(amphos)_2$ (3.5 mg, 5.0 µmol, 5.0 mol%), and activated zinc powder 100-mesh 99.5% (20 mg, 0.30 mmol, 3.0 equiv.). The vial was transferred into a nitrogen-filled glovebox. Dry DMF (1 mL, c = 0.1 M) was added to the solids. Subsequently, alkyl iodide (0.2 mmol, 2 equiv.) and pyridine (4 µL, 4 mg, 5 µmol, 0.5 equiv.) were added at 25 °C. The vial was sealed with a Teflon-lined screw cap, removed from the glovebox, and transferred to a heating block preheated at 60 °C where the reaction mixture was stirred rigorously (850 rpm) for 1 h. A color change from yellow to dark brown was observed. The mixture was cooled to 25 °C, subsequently diluted with ethyl acetate (10 mL), and thereafter poured into a separatory funnel. The organic layer was washed with water (1 × 10 mL). The aqueous layer was then extracted with ethyl acetate (3 × 10 mL). The organic layers were combined, dried over $MgSO_4$, filtered, and concentrated under reduced pressure. To the residue was added mesitylene (14 µL, 12 mg, 0.10 mmol) as an internal standard. The ^1H NMR resonances of the diphenyl ether protons of the product between 6.8 and 7.4 ppm were integrated relative to the ^1H NMR resonances of the aromatic protons of mesitylene (δ = 6.70 ppm).

Table S1: Optimization of yield as a function of catalyst

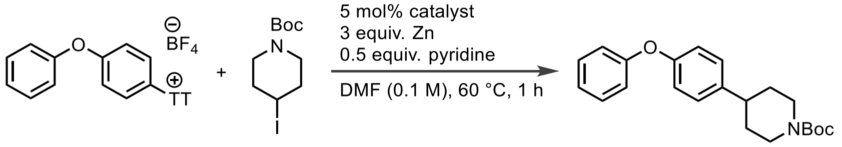

TT-1 **3**

Entry	Catalyst	Yield
1	No catalyst	n.o.
2	$NiBr_2$ + 10 mol% bpy	n.o.

3	$CoBr_2$	n.o.
4	$FeBr_3$	n.o.
5	$Pd(OAc)_2$	20%
6	$PdCl_2$	<5%
7	$PdCl_2(PPh_3)_2$	19%
8	$PdCl_2$(dppf)	20%
**9**	**$PdCl_2$(amphos)$_2$**	**75%**
10	$PdCl_2(PCy_3)_2$	<5%
11	$PdCl_2$(tri-*o*-tolylphosphine)$_2$	40%
12	$PdCl_2$ + 10 mol% dppe	<5%
13	$PdCl_2$ + 10 mol% DPE Phos	44%
14	$PdCl_2$ + 10 mol% XantPhos	<5%
15	10 mol% $PdCl_2$ + 30 mol% APhos	65%
16	10 mol% $PdCl_2$ + 30 mol% *p*- $Me_2N(C_4H_6)PCy_2$	5%
17	10 mol% $PdCl_2$ + 30 mol% Cphos	33%
18	10 mol% $PdCl_2$ + 30 mol% Trixiephos	25%
19	10 mol% $PdCl_2$ + 30 mol% $^tBuMePhos$	25%
20	10 mol% $PdCl_2$ + 30 mol% $^tBuDavePhos$	26%
21	10 mol% $Pd(dba)_2$ + 30 mol% APhos	15%

n.o. = not observed.

Table S2: Selectivity for *i*-PrAr versus *n*-PrAr

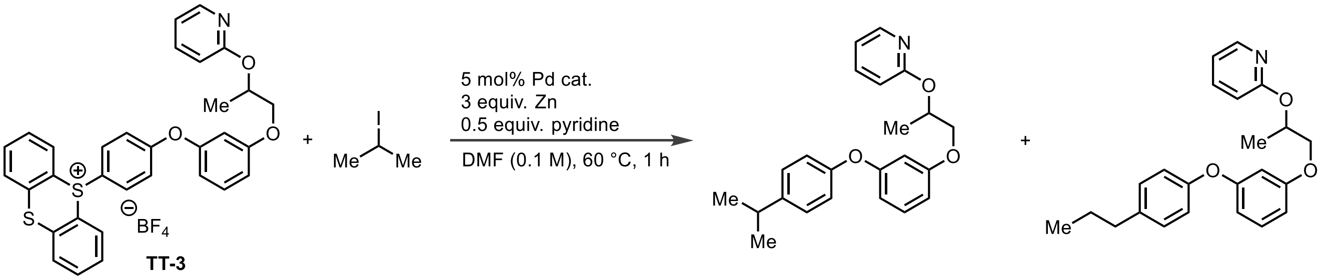

4
i-PrAr

S-1
n-PrAr

Entry	Catalyst	***i*-PrAr : *n*-PrAr**
1	$PdCl_2$(amphos)$_2$	>20 : 1
2	$PdCl_2$(tri-*o*-tolylphosphine)$_2$	4.2 : 1
3	$PdCl_2$(dppf)	0.84 : 1

Determination of selectivity for *i*-PrAr versus *n*-PrAr

The selectivity for the reaction of **TT-3** with 2-iodopropane was determined through independent synthesis of the two isomers (product **4** and product **S-1**) for accurate comparison. The alkylation of **TT-3** was carried out

with 2-iodopropane and 1-iodopropane under the standard reaction conditions (see page S23 for procedures for the synthesis of compound **4** and compound **S-1** and characterization of products). With pure samples of products **4** and **S-1**, the selectivity for the reaction of **TT-3** with 2-iodopropane with three different palladium catalysts could be determined. The ratio of the two isomers present in the reaction mixture (product **4** and product **S-1**) were analyzed by ^{1}H NMR spectroscopy and the ratio of the isomers was used to determine the selectivity. The spectra were recorded with a pre-acquisition delay time of 20 s to ensure accurate integration. The ^{1}H NMR resonances of the protons of the product **4** at 2.82 ppm or 0.95 ppm and the proton of product **S-1** at 2.49 ppm were integrated and compared to determine the selectivity.

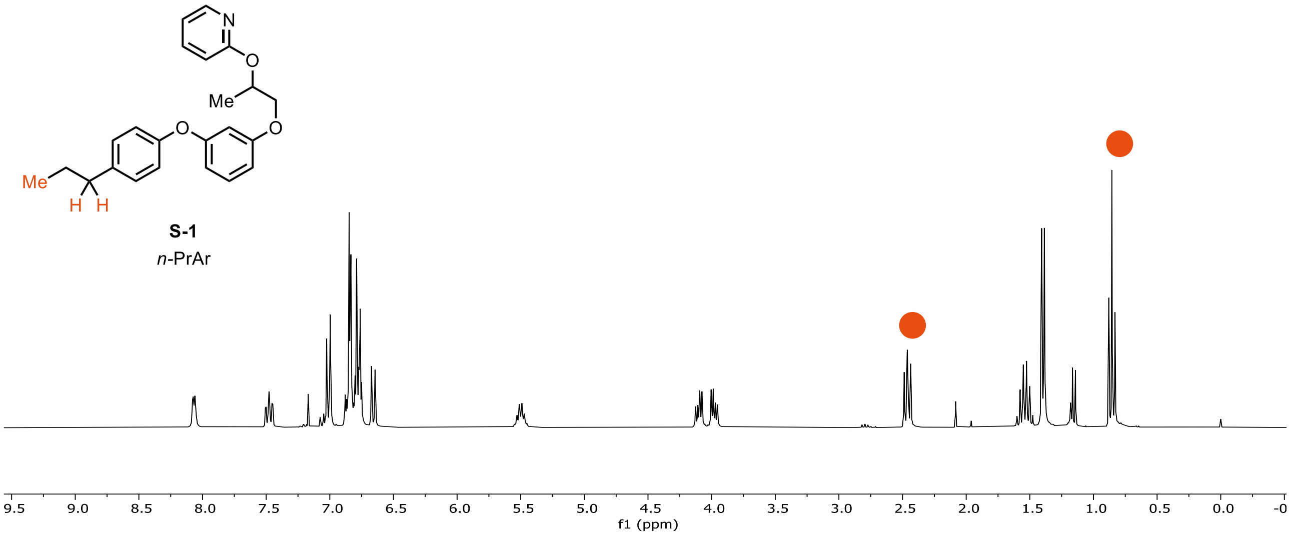

Figure S1: ^{1}H NMR spectrum of compound **S-1** ($CDCl_3$, 500 MHz, 298 K) obtained from the reaction of **TT-3** with 1-iodopropane under standard reaction conditions.

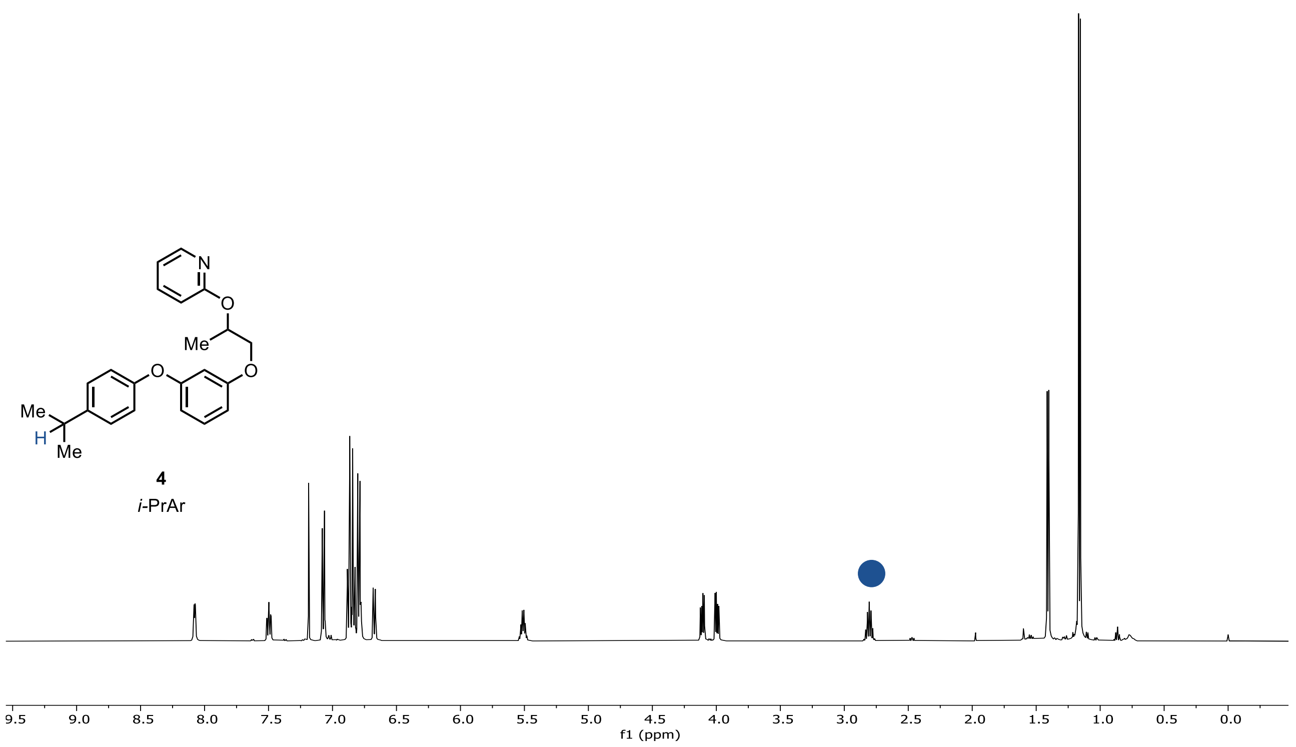

Figure S2: ^{1}H NMR spectrum of compound **4** ($CDCl_3$, 500 MHz, 298 K) obtained from the reaction of **TT-3** with 2-iodopropane under standard reaction conditions.

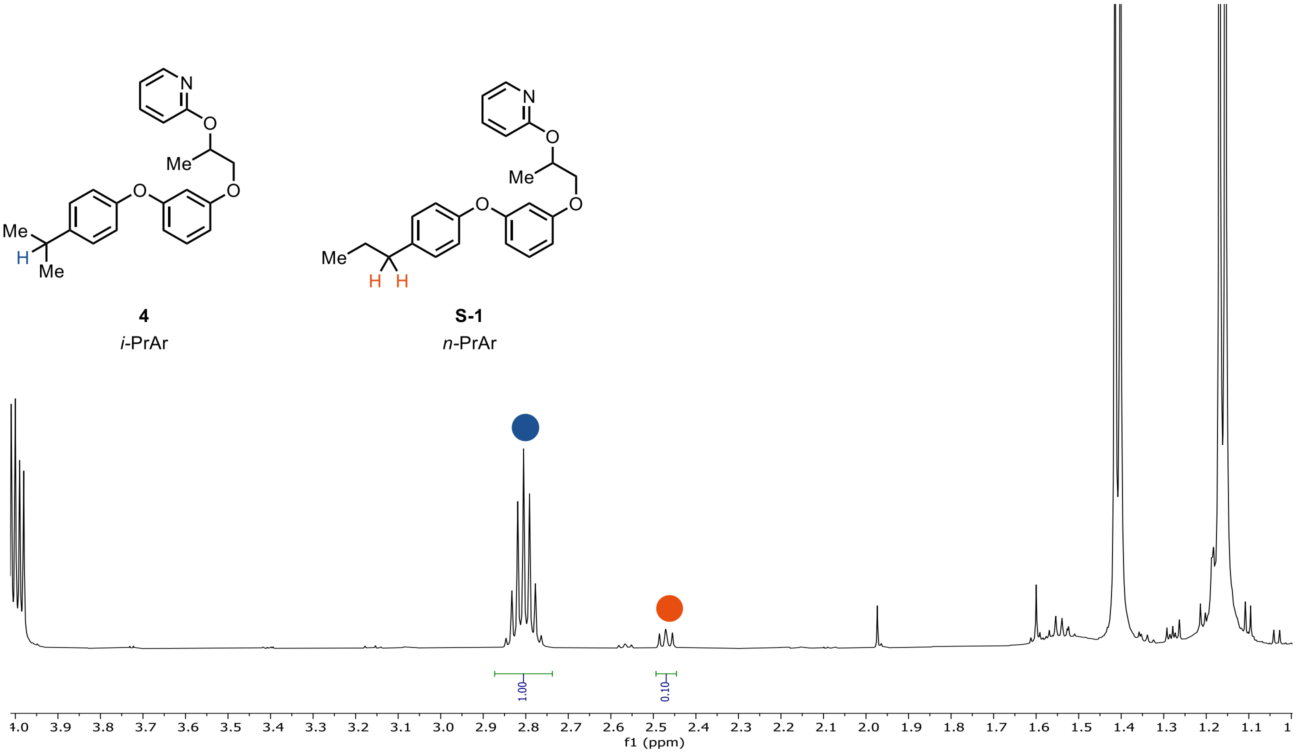

Figure S3: ^{1}H NMR spectrum of compound **4** reaction mixture ($CDCl_3$, 500 MHz, 298 K) obtained from the reaction of **TT-3** with 2-iodopropane with $PdCl_2(amphos)_2$.

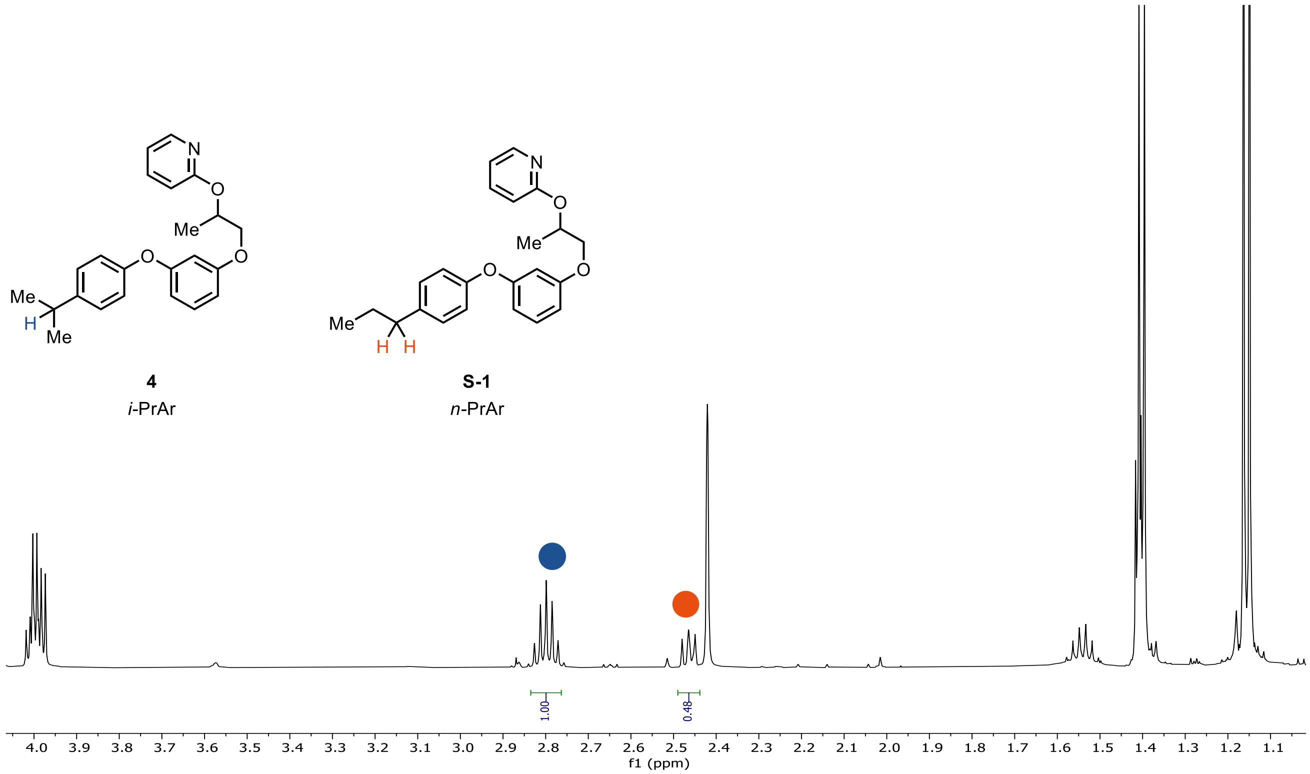

Figure S4: ^{1}H NMR spectrum of compound **4** reaction mixture ($CDCl_3$, 500 MHz, 298 K) obtained from the reaction of **TT-3** with 2-iodopropane with $PdCl_2$(tri-*o*-tolylphosphine)$_2$.

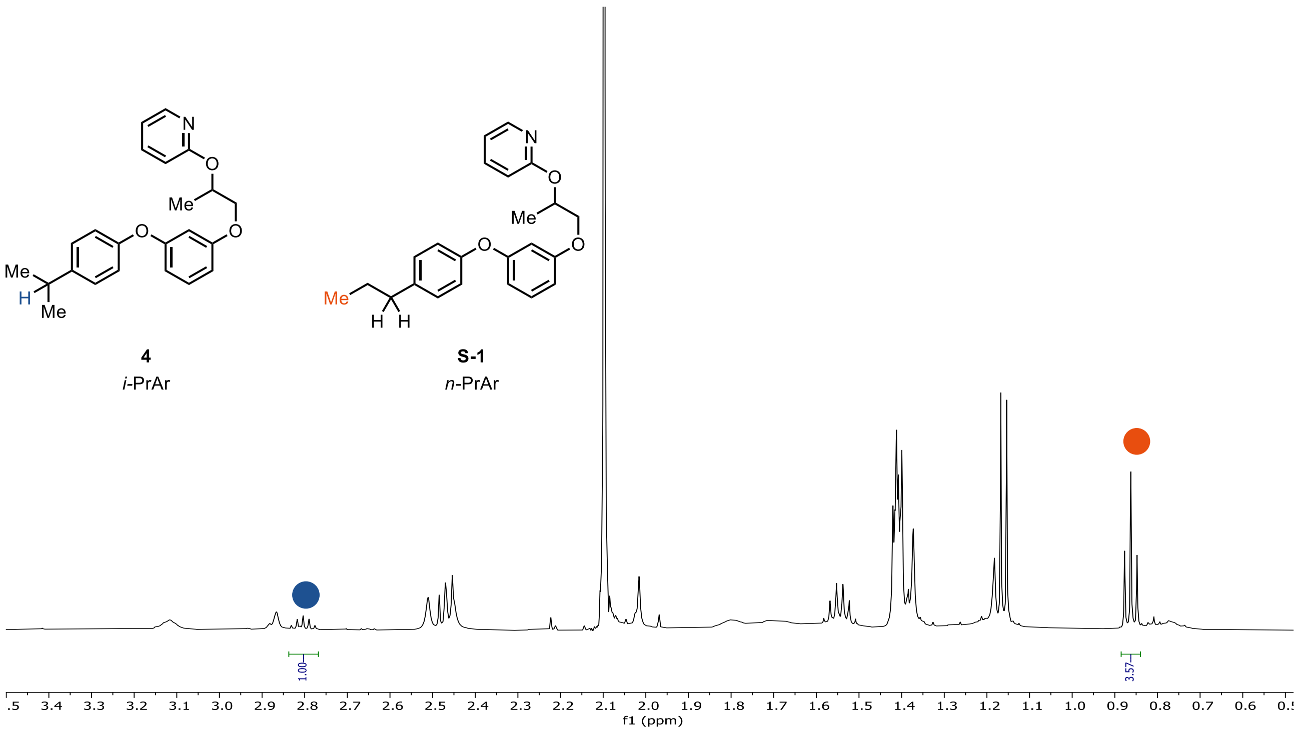

Figure S5: ^{1}H NMR spectrum of compound **3** reaction mixture ($CDCl_3$, 500 MHz, 298 K) obtained from the reaction of **TT-3** with 2-iodopropane with $PdCl_2$(dppf).

Table S3: Optimization of yield as a function of solvent

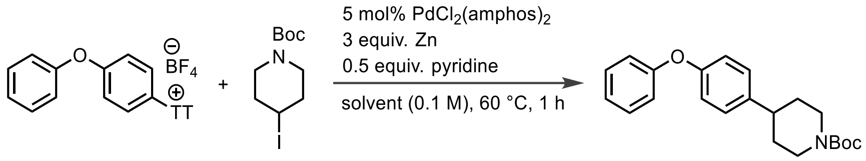

TT-1 **3**

Entry	Solvent	Yield
**1**	**DMF**	**75%**
2	DMA	63%
3	DMPU	59%
4	THF	47%
5	MeCN	51%
6	DCM	32%
7	Toluene	20%
8	DMSO	48%

Table S4: Optimization of yield as a function of reducing agent

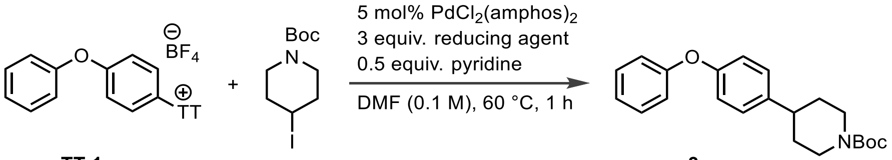

TT-1 **3**

Entry	Reducing agent	Yield
1	No reducing agent	n.o.
2	Mn	n.o.
**3**	**Zn**	**75%**
4	TDAE	n.o.
5	SmI_2	n.o.

n.o. = not observed.

Table S5: Optimization of yield as a function of an additive

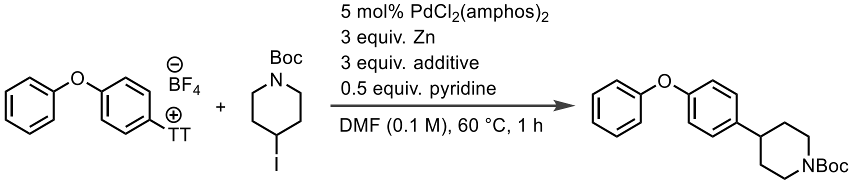

TT-1 **3**

Entry	Additive	Yield

**1**	**No additive**	**75%**
2	No additive, no pyridine	66%
3	$MgCl_2$	47%
4	LiCl	49%
5	KF	38%
6	TBAB	60%
7	TBAI	60%
8	LiOTf	63%
9	$NaBF_4$	65%

General Procedure for the evaluation of a “one-pot” process

To a 4-mL borosilicate vial containing a Teflon-coated magnetic stirring bar were added pyriproxyfen (50 mg, 0.16 mmol, 1.0 equiv.), thianthrene-*S*-oxide (**TTO**) (36 mg, 0.16 mmol, 1.0 equiv.), and dry MeCN (0.66 mL, c = 0.25 M). $HBF_4 \cdot OEt_2$ (47 µL, 55 mg, 0.34 mmol, 2.2 equiv.) was added in one portion at 25 °C. The suspension was cooled to 0 °C, and trifluoroacetic anhydride (65 µL, 98 mg, 0.47 mmol, 3.0 equiv.) was added. The reaction mixture was stirred at 0 °C for 1 h. Subsequently, the mixture was allowed to warm to 25 °C, and the reaction mixture was stirred for an additional 5 h. The work-up was carried out in three different ways as described below. The subsequent cross-coupling reaction is also described below.

For entry 1: Under an argon atmosphere a 4-mL borosilicate vial containing a Teflon-coated magnetic stirring bar was charged with $PdCl_2(amphos)_2$ (5.5 mg, 7.8 µmol, 5.0 mol%), activated zinc powder 100-mesh 99.5% (31 mg, 0.47 mmol, 3.0 equiv.), and 1-boc-4-iodopiperidine (97 mg, 0.31 mmol, 2.0 equiv.). The reaction mixture of the aryl thianthrenation step described above was added to the 4-mL vial via a syringe, followed by dry DMF (0.5 mL), and pyridine (4 µL, 6 mg, 80 µmol, 0.5 equiv.). The vial was transferred to a heating block preheated at 60 °C where the reaction mixture was stirred rigorously (850 rpm) for 12 h. A color change from yellow to dark brown was observed. The mixture was cooled to 25 °C, subsequently diluted with ethyl acetate (10 mL), and thereafter poured into a separatory funnel. The organic layer was washed with water (1 × 10 mL). The aqueous layer was then extracted with ethyl acetate (3 × 10 mL). The organic layers were combined, dried over $MgSO_4$, filtered, and concentrated under reduced pressure. The resulting residue was analyzed by ^1H NMR spectroscopy and LCMS.

For entry 2: The reaction mixture of the aryl thianthrenation step described above was concentrated under reduced pressure. A second 4-mL borosilicate vial containing a Teflon-coated magnetic stirring bar was charged with $PdCl_2(amphos)_2$ (5.5 mg, 7.8 µmol, 5.0 mol%), activated zinc powder 100-mesh 99.5% (31 mg, 0.47 mmol, 3.0 equiv.), and 1-boc-4-iodopiperidine (97 mg, 0.31 mmol, 2.0 equiv.). Both vials were transferred into the glovebox. In the glovebox, to the vial containing the residue of the aryl thianthrenation step was added dry DMF (0.5 mL). The resulting mixture was transferred via a syringe to the second vial containing $PdCl_2(amphos)_2$, activated zinc powder, and 1-boc-4-iodopiperidine followed by the addition of pyridine (4 µL, 6 mg, 80 µmol, 0.5 equiv.). The vial was sealed, removed from the glovebox, and transferred to a heating block preheated at 60 °C where the reaction mixture was stirred rigorously (850 rpm) for 12 h. A

color change from yellow to dark brown was observed. The mixture was cooled to 25 °C, subsequently diluted with ethyl acetate (10 mL), and thereafter poured into a separatory funnel. The organic layer was washed with water (1 × 10 mL). The aqueous layer was then extracted with ethyl acetate (3 × 10 mL). The organic layers were combined, dried over $MgSO_4$, filtered, and concentrated under reduced pressure. The resulting residue was analyzed by ^{1}H NMR spectropscopy and LCMS.

For entry 3: To the reaction mixture of the aryl thianthrenation step described above was added K_2CO_3 (47 mg, 0.34 mmol, 2.2 equiv.). The reaction mixture was stirred for 15 minutes. The resulting mixture was filtered and concentrated under reduced pressure. A second 4-mL borosilicate vial containing a Teflon-coated magnetic stirring bar was charged with $PdCl_2(amphos)_2$ (5.5 mg, 7.8 µmol, 5.0 mol%), activated zinc powder 100-mesh 99.5% (31 mg, 0.47 mmol, 3.0 equiv.), and 1-boc-4-iodopiperidine (97 mg, 0.31 mmol, 2.0 equiv.). Both vials were transferred into the glovebox. In the glovebox, to the vial containing the residue of the aryl thianthrenation step was added dry DMF (0.5 mL). The resulting mixture was transferred via a syringe to the second vial containing $PdCl_2(amphos)_2$, activated zinc powder, and 1-boc-4-iodopiperidine followed by the addition of pyridine (4 µL, 6 mg, 80 µmol, 0.5 equiv.). The vial was sealed, removed from the glovebox, and was transferred to a heating block preheated at 60 °C where the reaction mixture was stirred rigorously (850 rpm) for 12 h. A color change from yellow to dark brown was observed. The mixture was cooled to 25 °C, subsequently diluted with ethyl acetate (10 mL), and thereafter poured into a separatory funnel. The organic layer was washed with water (1 × 10 mL). The aqueous layer was then extracted with ethyl acetate (3 × 10 mL). The organic layers were combined, dried over $MgSO_4$, filtered, and concentrated under reduced pressure. The resulting residue was analyzed by ^{1}H NMR spectroscopy and LCMS.

Table S6: Evaluation of a "one-pot" process

A short optimization was carried out to see whether a "one-pot" process was possible (procedures are described above). No attempt made gave high yields and the main by-products detected were unreacted aryl thianthrenium salt **TT-3** and the reduced aryl thianthrenium salt. Therefore, a two-step sequence must be used to obtain high yields of desired product.

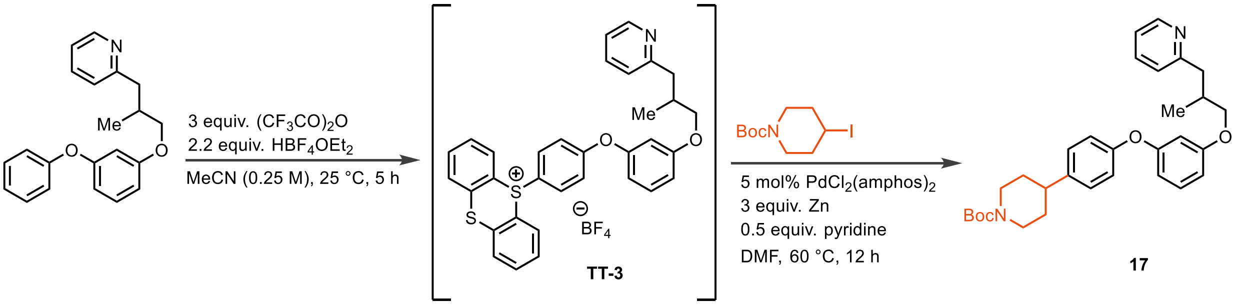

Entry	Yield	Notes
1	n.o.	Direct addition
2	<5%	Solvent swap
3	<5%[a]	Solvent swap

[a]2.2 equiv. K_2CO_3 was added.

Synthesis of aryl thianthrenium salts

Estrone methyl ether thianthrenium salt derivative TT-4

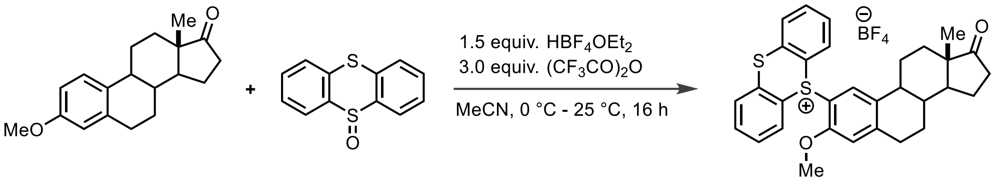

TT-4, 21%

Under an ambient atmosphere, a 25 mL glass-vial was charged with estrone 3-methyl ether (500 mg, 1.76 mmol, 1.00 equiv.), thianthrene-S-oxide (**TTO**) (408 mg, 1.76 mmol, 1.00 equiv.), and dry MeCN (7.0 mL, c = 0.25 M). $HBF_4 \cdot OEt_2$ (0.29 mL, 340 mg, 2.1 mmol, 1.2 equiv.) was added in one portion at 25 °C. The suspension was cooled to 0 °C, and trifluoroacetic anhydride (0.73 mL, 1.1 g, 5.3 mmol, 3.0 equiv.) was added. The reaction mixture was stirred at 0 °C for 1 h. Subsequently, the mixture was allowed to warm to 25 °C, and the reaction mixture was stirred for an additional 15 h. The mixture was concentrated under reduced pressure, diluted with DCM (15 mL), and washed with water (25 mL) and aqueous $NaBF_4$ solution (2 × 25 mL, 10 % (w/w)). The organic layer was dried over $MgSO_4$, filtered, and the solvent was removed under reduced pressure. The residue was purified by chromatography on silica gel eluting with dichloromethane / *i*-PrOH, (1:0 gradient to 9:1 (v/v)) to afford 219 mg (21%) of **TT-4** as a pale yellow powder.

R_f = 0.58 (dichloromethane/*i*-PrOH, 2:3 (v:v))

NMR Spectroscopy:

^{1}H NMR (500 MHz, CD_3CN, 298 K) δ 7.98 (ddd, *J* = 11.1, 8.0, 1.4 Hz, 2H), 7.72 (dd, *J* = 8.0, 1.3 Hz, 1H), 7.67 (dd, *J* = 7.9, 1.4 Hz, 1H), 7.62 (td, *J* = 7.7, 1.4 Hz, 1H), 7.57 (td, *J* = 7.7, 1.5 Hz, 1H), 7.51 (dtd, *J* = 13.1, 7.6, 1.4 Hz, 2H), 6.73 (s, 1H), 6.28 (s, 1H), 3.65 (s, 3H), 2.71 – 2.56 (m, 2H), 2.16 (dd, *J* = 19.2, 8.8 Hz, 1H), 1.88 – 1.69 (m, 4H), 1.51 – 1.43 (m, 2H), 1.38 – 0.92 (m, 6H), 0.89 (s, 3H).

^{13}C{^{1}H} NMR (126 MHz, CD_3CN, 298 K) δ 220.5, 156.5, 147.6, 138.1, 137.5, 135.7, 135.7, 135.6, 135.4, 135.0, 131.2, 131.2, 130.8, 130.6, 127.0, 115.2, 106.4, 57.5, 50.7, 49.4, 48.4, 44.1, 38.3, 36.2, 32.1, 30.3, 27.2, 26.4, 26.2, 22.0, 14.0.

^{19}F{^{1}H} NMR (282 MHz, CD_3CN, 298 K) δ -151.67.

HRMS-ESIpos (m/z) calc'd for $C_{31}H_{31}O_2S_2^+$, 499.1760; found, 499.1734; deviation: –0.7 ppm

Synthesis of alkyl iodides

Sulbactam iodide derivative S-2

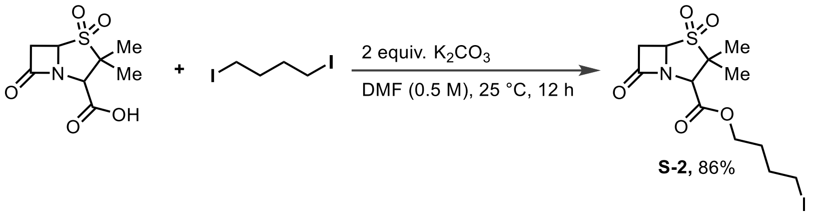

A 20 mL glass vial containing a Teflon-coated magnetic stirring bar was charged with sulbactam (500 mg, 2.14 mmol, 1.00 equiv.) and K_2CO_3 (593 mg, 4.29 mmol, 2.00 equiv.) at 25 °C. DMF was added (4.3 mL, c = 0.50 M) followed by 1,4-diiodo-butane (1.41 mL, 3.32 g, 10.7 mmol, 5.00 equiv.). The vial was sealed with a Teflon-lined screw cap, wrapped in aluminium foil, and the reaction mixture was stirred at 25 °C for 12 h. The resulting reaction mixture was diluted with ethyl acetate (40 mL) and poured into a seperatory funnel containing water (40 mL). The organic layer was seperated, and the aqeuous layer was further extracted with ethyl acetate (2 × 40 mL). The organic layers were combined, dried over $MgSO_4$, filtered, and concentrated under reduced pressure. The resulting residue was purified by column chromatography on silica gel eluting with a solvent mixture of hexanes / ethyl acetate, (1:1 gradient to 1:4 (v/v)) to afford 767.7 mg (86%) of desired product **S-2** as a yellow oil.

R_f = 0.70 (hexanes/EtOAc, 2:3 (v:v))

NMR Spectroscopy:

^{1}H NMR (500 MHz, $CDCl_3$, 298 K) δ 4.66 – 4.56 (m, 1H), 4.38 (s, 1H), 4.27 – 4.10 (m, 2H), 3.54 – 3.40 (m, 2H), 3.22 (t, J = 6.6 Hz, 2H), 1.95 – 1.77 (m, 4H), 1.61 (s, 3H), 1.42 (s, 3H).

^{13}C{^{1}H} NMR (126 MHz, $CDCl_3$, 298 K) δ 170.8, 167.0, 65.4, 63.3, 62.8, 61.2, 38.5, 29.8, 29.4, 20.5, 18.8, 5.5.

HRMS-ESIpos (m/z) calc'd for $C_{12}H_{18}NO_5SNaI$ $[M+Na]^+$, 437.9843; found, 437.9843; deviation: 0.0 ppm

Fmoc-Arg(Pbf)-OH iodide derivative S-3

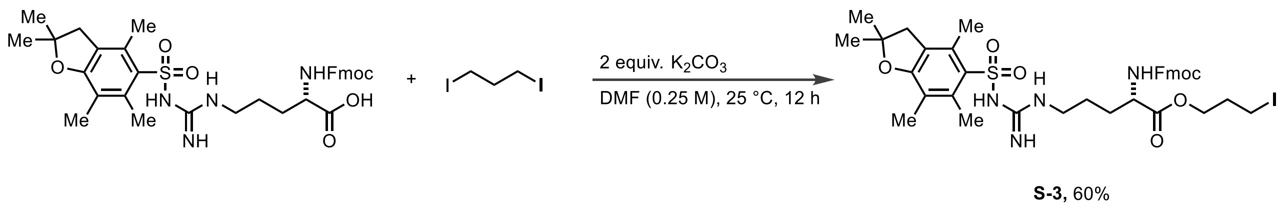

A 20 mL glass vial containing a Teflon-coated magnetic stirring bar was charged with Fmoc-Arg(Pbf)-OH (1.0 g, 1.5 mmol, 1.0 equiv.) (Pbf = 2,2,4,6,7-pentamethyldihydrobenzofuran-5-sulfonyl group) and K_2CO_3 (426 mg, 3.08 mmol, 2.00 equiv.) at 25 °C. DMF was added (6.0 mL, c = 0.25 M) followed by 1,4-diiodo-propane (0.89 mL, 2.3 g, 7.7 mmol, 5.0 equiv.). The vial was sealed with a Teflon-lined screw cap, wrapped in

aluminium foil, and the reaction mixture was stirred at 25 °C for 12 h. The resulting reaction mixture was diluted with ethyl acetate (50 mL) and poured into a seperatory funnel containing water (50 mL). The organic layer was seperated, and the aqeuous layer was further extracted with ethyl acetate (2 × 50 mL). The organic layers were combined, dried over $MgSO_4$, filtered, and concentrated under reduced pressure. The resulting residue was purified by column chromatography on silica gel eluting with a solvent mixture of hexanes / ethyl acetate, (3:7 gradient to 1:9 (v/v)) to afford 756.1 mg (60%) of desired product **S-3** as a yellow foam.

R_f = 0.44 (hexanes/EtOAc, 1:4 (v:v))

NMR Spectroscopy:

^{1}H NMR (300 MHz, $CDCl_3$, 298 K) δ 7.75 (d, *J* = 7.6 Hz, 2H), 7.58 (d, *J* = 7.5 Hz, 2H), 7.39 (t, *J* = 7.5 Hz, 2H), 7.33 – 7.19 (m, 2H), 6.40 – 6.16 (m, 3H), 5.83 (d, *J* = 8.2 Hz, 1H), 4.41 – 4.25 (m, 3H), 4.24 – 4.12 (m, 3H), 3.34 – 3.08 (m, 4H), 2.92 (s, 2H), 2.60 (s, 3H), 2.52 (s, 3H), 2.11 – 2.07 (m, 5H), 1.86 (dt, *J* = 14.3, 7.4 Hz, 1H), 1.73 (dq, *J* = 14.5, 8.1 Hz, 1H), 1.60 (t, *J* = 7.6 Hz, 2H), 1.44 (s, 6H).

^{13}C{^{1}H} NMR (75 MHz, $CDCl_3$, 298 K) δ 172.2, 158.9, 156.5, 156.4, 143.9, 143.7, 141.3, 138.4, 132.8, 132.3, 127.8, 127.2, 125.2, 124.8, 120.1, 117.7, 86.5, 67.3, 65.2, 53.8, 47.1, 43.3, 40.8, 32.0, 29.8, 28.7, 25.5, 19.4, 18.1, 12.6, 1.4.

HRMS-ESIpos (m/z) calc'd for $C_{37}H_{45}IN_4O_7SNa$ $[M+Na]^+$, 839.1946; found, 839.1946; deviation: –0.0 ppm.

3-Iodo epiandrosterone S-4

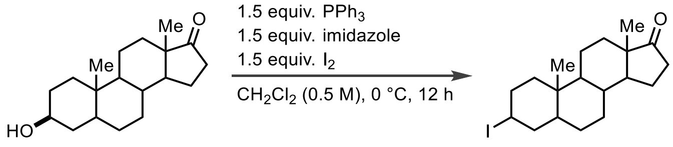

S-4, 77%

To a flame dried Schlenk finger under argon atmosphere wrapped in aluminum foil and equipped with a stirrer bar were added PPh_3 (677 mg, 2.58 mmol, 1.50 equiv.) and imidazole (175 mg, 2.58 mmol, 1.50 equiv.). Dichloromethane (3.4 mL, c = 0.50 M) was added via a syringe followed by epiandrosterone (500 mg, 1.72 mmol, 1.00 equiv.) at 25 °C. The resulting solution was allowed to stir for 15 minutes. The mixture was then cooled to 0 °C and I_2 (655 mg, 2.58 mmol, 1.50 equiv.) was added. The reaction mixture was stirred at 0 °C for 30 minutes and then warmed to 25 °C and stirred at 25 °C for 12 h. The reaction mixture was quenched with an aqueous solution of sodium thiosulfate (20 mL) and poured into a separatory funnel. The aqueous solution was extracted with dichloromethane (3 × 20 mL). The organic layers were combined, dried over $MgSO_4$, filtered, and concentrated under reduced pressure. The resulting residue was purified by column chromatography on silica gel eluting with a solvent mixture of hexanes / ethyl acetate, (10:0 gradient to 1:4 (v/v)) to afford 531.6 mg (77%) of desired product **S-4** as a white solid.

R_f = 0.58 (hexanes/EtOAc, 9:1 (v:v))

NMR Spectroscopy:

^{1}H NMR (500 MHz, $CDCl_3$, 298 K) δ 5.04 – 4.81 (m, 1H), 2.43 (ddd, *J* = 19.2, 8.9, 1.2 Hz, 1H), 2.07 (dt, *J* = 19.2, 9.0 Hz, 1H), 1.98 – 1.87 (m, 2H), 1.84 – 1.62 (m, 7H), 1.58 – 1.43 (m, 5H), 1.36 – 1.24 (m, 5H), 1.08 (qd, *J* = 12.6, 5.6 Hz, 1H), 0.85 (s, 3H), 0.82 (s, 3H).

$^{13}C\{^1H\}$ NMR (126 MHz, $CDCl_3$, 298 K) δ 221.4, 54.1, 51.6, 48.0, 42.2, 38.9, 37.7, 36.9, 36.0, 35.2, 34.5, 32.8, 31.7, 30.8, 27.6, 21.9, 20.2, 14.0, 13.5.

The ^{1}H NMR spectra is in agreement with literature.[8]

HRMS-ESIpos (m/z) calc'd for $C_{19}H_{29}OINa$ $[M+Na]^+$, 423.1155; found, 423.1154; deviation: 0.3 ppm.

Alkylation of aryl thianthrenium salts with alkyl iodides

Methyl bifonazole derivative 1

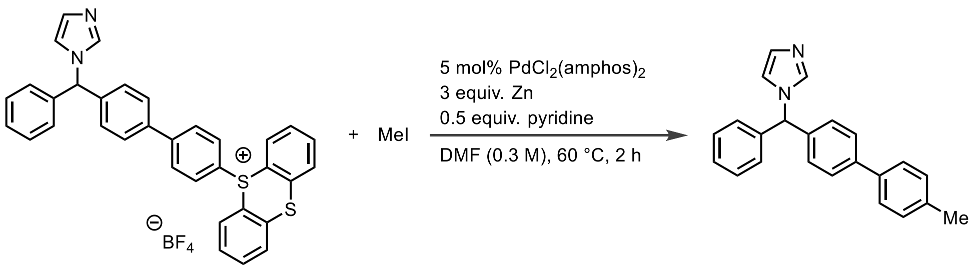

TT-5 **1,** 63%

To a 4-mL borosilicate vial containing a Teflon-coated magnetic stirring bar were added bifonazole thianthrenium salt **TT-5** (184 mg, 0.300 mmol, 1.00 equiv.), $PdCl_2(amphos)_2$ (10.6 mg, 15.0 µmol, 5.00 mol%), and activated zinc dust (58.9 mg, 0.900 mmol, 3.00 equiv.). The vial was transferred into a nitrogen-filled glovebox. Dry DMF (1 mL, c = 0.3 M) was added to the solids. Subsequently, methyl iodide (37 µL, 85 mg, 0.60 mmol, 2.0 equiv.) and pyridine (12 µL, 12 mg, 0.15 mmol, 0.50 equiv.) were added at 25 °C. The vial was sealed with a Teflon-lined screw cap, removed from the glovebox, and transferred to a heating block preheated at 60 °C where the reaction mixture was stirred rigorously (850 rpm) for 2 h. A color change from yellow to dark brown was observed. The mixture was cooled to 25 °C, subsequently diluted with ethyl acetate (40 mL), and thereafter poured into a separatory funnel. The organic layer was washed with water (1 × 40 mL). The aqueous layer was then extracted with ethyl acetate (3 × 40 mL). The organic layers were combined, dried over $MgSO_4$, filtered, and concentrated under reduced pressure. The resulting residue was purified by column chromatography on silica gel eluting with a solvent mixture of hexanes / ethyl acetate, (1:4 gradient to 0:10 (v/v)) to afford 73.3 mg (75%) of a mixture of the desired product and the reduced thianthrenium salt. Further purification by HPLC (Triart C18 (150 mm × 4.6 mm: 5 µm), *i*-PrOH / water = 60:40, flow rate = 1 mL/min, 25 °C) provided 61.2 mg (63%) of desired product **1** as a pale yellow oil.

R_f = 0.71 (dichloromethane/MeOH, 9:1 (v:v))

NMR Spectroscopy:

^{1}H NMR (500 MHz, $CDCl_3$, 298 K) δ 7.56 (d, *J* = 8.2 Hz, 2H), 7.48 (d, *J* = 8.2 Hz, 2H), 7.41 – 7.33 (m, 3H), 7.30 – 7.21 (m, 3H), 7.19 – 7.10 (m, 5H), 6.91 (s, 1H), 6.56 (s, 1H), 2.40 (s, 3H).

$^{13}C\{^1H\}$ NMR (126 MHz, $CDCl_3$, 298 K) 141.4, 139.2, 137.8, 137.6, 137.5, 129.7, 129.1, 128.6, 128.6, 128.2, 127.5, 127.1, 65.1, 21.3.

HRMS-ESIpos (m/z) calc'd for $C_{23}H_{21}N_2$ $[M+H]^+$, 325.1699; found, 325.1698; deviation: 0.5 ppm.

Methyl indomethacin methyl ester derivative 2

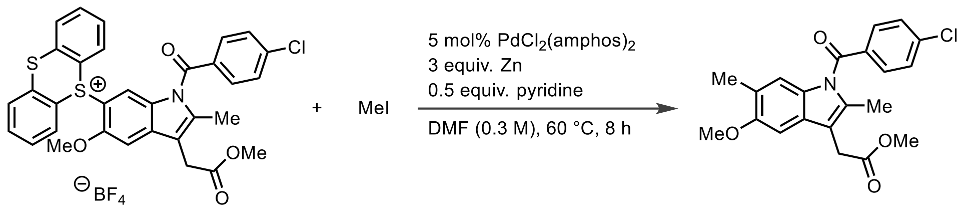

TT-6 **2,** 78%

To a 4-mL borosilicate vial containing a Teflon-coated magnetic stirring bar were added indomethacin methyl ester thianthrenium salt **TT-6** (202 mg, 0.300 mmol, 1.00 equiv.), $PdCl_2(amphos)_2$ (10.6 mg, 15.0 µmol, 5.00 mol%), and activated zinc dust (58.9 mg, 0.900 mmol, 3.00 equiv.). The vial was transferred into nitrogen-filled glovebox. Dry DMF (1 mL, c = 0.3 M) was added to the solids. Subsequently, methyl iodide (37 µL, 85 mg, 0.60 mmol, 2.0 equiv.) and pyridine (12 µL, 12 mg, 0.15 mmol, 0.50 equiv.) were added at 25 °C. The vial was sealed with a Teflon-lined screw cap, removed from the glovebox, and transferred to a heating block preheated at 60 °C where the reaction mixture was stirred rigorously (850 rpm) for 8 h. A color change from yellow to dark brown was observed. The mixture was cooled to 25 °C, subsequently diluted with ethyl acetate (40 mL), and thereafter poured into a separatory funnel. The organic layer was washed with water (1 × 40 mL). The aqueous layer was then extracted with ethyl acetate (3 × 40 mL). The organic layers were combined, dried over $MgSO_4$, filtered, and concentrated under reduced pressure. The resulting residue was purified by column chromatography on silica gel eluting with a solvent mixture of hexanes / ethyl acetate, (4:1 gradient to 3:2 (v/v)) to afford 90.8 mg (78%) of desired product **2** as a yellow oil.

R_f = 0.73 (hexanes/EtOAc, 4:1 (v:v))

NMR Spectroscopy:

^{1}H NMR (500 MHz, $CDCl_3$, 298 K) δ 7.66 (d, *J* = 8.5 Hz, 2H), 7.47 (d, *J* = 8.5 Hz, 2H), 6.89 (s, 1H), 6.87 (s, 1H), 3.88 (s, 3H), 3.70 (s, 3H), 3.67 (s, 2H), 2.32 (s, 3H), 2.16 (s, 3H).

$^{13}C\{^1H\}$ NMR (75 MHz, $CDCl_3$, 298 K) δ 171.5, 168.5, 154.7, 139.2, 134.2, 134.2, 131.3, 130.5, 129.1, 128.5, 123.4, 116.1, 112.6, 98.8, 55.8, 52.2, 30.4, 17.2, 13.5.

HRMS-EI (m/z) calc'd for $C_{21}H_{20}NO_4Cl$, 385.1075; found, 385.1077; deviation: –0.4 ppm.

Isopropyl pyriproxyfen derivative 4

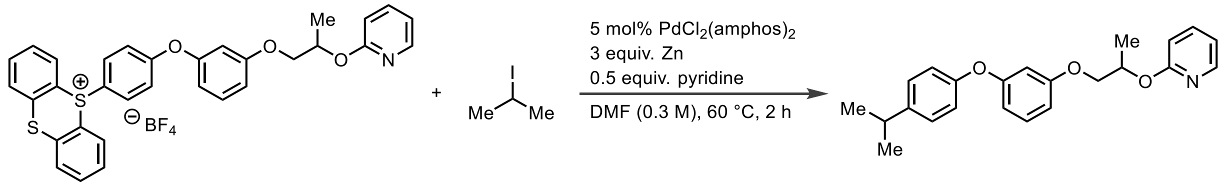

TT-3 **4,** 80%

To a 4-mL borosilicate vial containing a Teflon-coated magnetic stirring bar were added pyriproxyfen thianthrenium salt **TT-3** (187 mg, 0.300 mmol, 1.00 equiv.), $PdCl_2(amphos)_2$ (10.6 mg, 15.0 µmol, 5.00 mol%), and activated zinc powder (58.9 mg, 0.900 mmol, 3.00 equiv.). The vial was transferred into a nitrogen-filled glovebox. Dry DMF (1 mL, c = 0.3 M) was added to the solids. Subsequently, 2-iodopropane (60 µL, 10 mg, 0.6 mmol, 2 equiv.) and pyridine (12 µL, 12 mg, 0.15 mmol, 0.50 equiv.) were added at 25 °C. The vial was sealed with a Teflon-lined screw cap, removed from the glovebox, and transferred to a heating block preheated at 60 °C where the reaction mixture was stirred rigorously (850 rpm) for 2 h. A color change from yellow to dark brown was observed. The mixture was cooled to 25 °C, subsequently diluted with ethyl acetate (40 mL), and thereafter poured into a separatory funnel. The organic layer was washed with water (1 × 40 mL). The aqueous layer was then extracted with ethyl acetate (3 × 40 mL). The organic layers were combined, dried over $MgSO_4$, filtered, and concentrated under reduced pressure. The resulting residue was purified by column chromatography on silica gel eluting with a solvent mixture of hexanes / ethyl acetate, (9:1 gradient to 4:1 (v/v)) to afford 87.6 mg (80%) as a >20:1 mixture of desired product **4** and *n*-propyl product **S-1** as a colorless oil.

R_f = 0.66 (hexanes/EtOAc, 4:1 (v:v))

NMR Spectroscopy:

^{1}H NMR (300 MHz, $CDCl_3$, 298 K) δ 8.16 (dd, *J* = 4.6, 1.6 Hz, 1H), 7.57 (ddd, *J* = 8.3, 7.1, 2.0 Hz, 1H), 7.15 (d, *J* = 8.8 Hz, 2H), 7.01 – 6.80 (m, 7H), 6.80 – 6.67 (m, 1H), 5.59 (dtd, *J* = 11.4, 6.4, 5.1 Hz, 1H), 4.19 (dd, *J* = 9.9, 5.3 Hz, 1H), 4.07 (dd, *J* = 9.9, 4.9 Hz, 1H), 2.89 (hept, *J* = 6.9 Hz, 1H), 1.49 (d, *J* = 6.4 Hz, 3H), 1.24 (d, *J* = 6.9 Hz, 6H).

^{13}C{^{1}H} NMR (126 MHz, $CDCl_3$, 298 K) δ 163.0, 156.2, 154.9, 150.6, 146.6, 142.9, 138.5, 127.3, 120.3, 117.5, 116.6, 115.6, 111.5, 70.9, 69.1, 33.2, 24.0, 16.9.

HRMS-CI (m/z) calc'd for $C_{23}H_{26}NO_3$ $[M+H]^+$, 364.1907; found, 364.1909; deviation: –0.6 ppm.

***n*-Propyl pyriproxyfen derivative S-1**

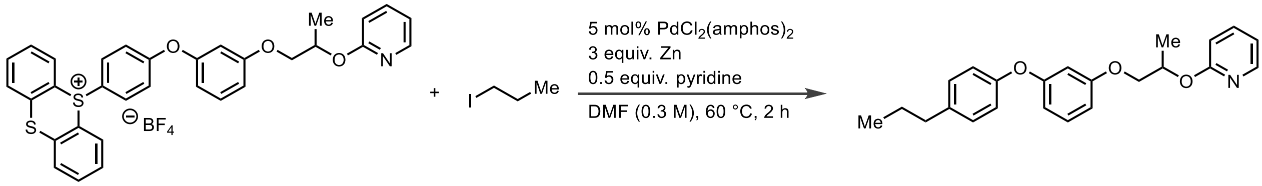

TT-3 **S-1,** 37%

To a 4-mL borosilicate vial containing a Teflon-coated magnetic stirring bar were added pyriproxyfen thianthrenium salt **TT-3** (187 mg, 0.300 mmol, 1.00 equiv.), $PdCl_2(amphos)_2$ (10.6 mg, 15.0 µmol, 5.00 mol%), and activated zinc powder (58.9 mg, 0.900 mmol, 3.00 equiv.). The vial was transferred into a nitrogen-filled glovebox. Dry DMF (1 mL, c = 0.3 M) was added to the solids. Subsequently, 1-iodopropane (59 µL, 100 mg, 0.60 mmol, 2.0 equiv.) and pyridine (12 µL, 12 mg, 0.15 mmol, 0.50 equiv.) were added at 25 °C. The vial was sealed with a Teflon-lined screw cap, removed from the glovebox, and transferred to a heating block preheated at 60 °C where the reaction mixture was stirred rigorously (850 rpm) for 2 h. A color change from yellow to dark brown was observed. The mixture was cooled to 25 °C, subsequently diluted with ethyl acetate (40 mL), and thereafter poured into a separatory funnel. The organic layer was washed with water (1 × 40 mL). The aqueous layer was then extracted with ethyl acetate (3 × 40 mL). The organic layers were combined, dried over $MgSO_4$, filtered, and concentrated under reduced pressure. The resulting residue was purified by column chromatography on silica gel eluting with a solvent mixture of hexanes / ethyl acetate, (9:1 gradient to 4:1 (v/v)) to afford 40.1 mg (37%) of desired product **S-1** as a colorless oil.

$\mathbf{R_f}$ = 0.66 (hexanes/EtOAc, 4:1 (v:v))

NMR Spectroscopy:

^{1}**H NMR** (300 MHz, $CDCl_3$, 298 K) δ 8.16 (dd, J = 5.2, 2.0 Hz, 1H), 7.57 (ddd, J = 8.9, 7.2, 2.0 Hz, 1H), 7.20 – 7.01 (m, 2H), 7.01 – 6.80 (m, 7H), 6.75 (dt, J = 8.3, 0.9 Hz, 1H), 5.59 (h, J = 6.1 Hz, 1H), 4.19 (dd, J = 9.9, 5.3 Hz, 1H), 4.07 (dd, J = 9.9, 4.9 Hz, 1H), 2.55 (dd, J = 8.5, 6.7 Hz, 2H), 1.63 (dq, J = 14.7, 7.4 Hz, 2H), 1.49 (d, J = 6.4 Hz, 3H), 0.95 (t, J = 7.3 Hz, 3H)

^{13}**C{^{1}H} NMR** (75 MHz, $CDCl_3$, 298 K) δ 163.3, 156.4, 155.1, 151.0, 146.9, 138.8, 137.0, 129.6, 120.5, 117.8, 116.9, 115.9, 111.8, 71.2, 69.5, 37.4, 24.8, 17.1, 13.9.

HRMS-ESIpos (m/z) calc'd for $C_{23}H_{26}NO_3$ $[M+H]^+$, 364.1907; found, 364.1905; deviation: 0.7 ppm.

Boc-azetidinyl indomethacin methyl ester derivative 5

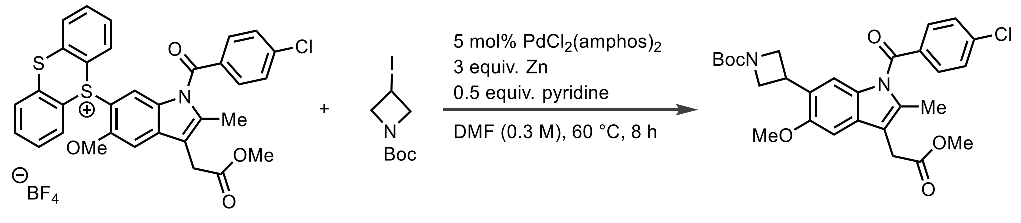

TT-6 **5,** 53%

To a 4-mL borosilicate vial containing a Teflon-coated magnetic stirring bar were added indomethacin methyl ester thianthrenium salt **TT-6** (202 mg, 0.300 mmol, 1.00 equiv.), $PdCl_2(amphos)_2$ (10.6 mg, 15.0 µmol, 5.00 mol%), and activated zinc dust (58.9 mg, 0.900 mmol, 3.00 equiv.). The vial was transferred into a nitrogen-filled glovebox. Dry DMF (1 mL, c = 0.3 M) was added to the solids. Subsequently, 1-boc-3-iodoazetidine (104 µL, 169 mg, 0.600 mmol, 2.00 equiv.) and pyridine (12 µL, 12 mg, 0.15 mmol, 0.50 equiv.) were added at 25 °C. The vial was sealed with a Teflon-lined screw cap, removed from the glovebox, and transferred to a

heating block preheated at 60 °C where the reaction mixture was stirred rigorously (850 rpm) for 8 h. A color change from yellow to dark brown was observed. The mixture was cooled to 25 °C, subsequently diluted with ethyl acetate (40 mL), and thereafter poured into a separatory funnel. The organic layer was washed with water (1 × 40 mL). The aqueous layer was then extracted with ethyl acetate (3 × 40 mL). The organic layers were combined, dried over $MgSO_4$, filtered, and concentrated under reduced pressure. The resulting residue was purified by column chromatography on silica gel eluting with a solvent mixture of hexanes / ethyl acetate, (4:1 gradient to 3:2 (v/v)) to afford 84.1 mg (53%) of desired product **5** as an orange oil.

R_f = 0.57 (hexanes/EtOAc, 1:1 (v:v))

NMR Spectroscopy:

^{1}H NMR (500 MHz, $CDCl_3$, 298 K) δ 7.65 (d, *J* = 8.6 Hz, 2H), 7.49 (d, *J* = 8.5 Hz, 2H), 6.99 (s, 1H), 6.90 (s, 1H), 4.17 (t, *J* = 8.5 Hz, 2H), 3.95 – 3.87 (m, 1H), 3.86 (s, 3H), 3.82 (dd, *J* = 8.3, 6.8 Hz, 2H), 3.70 (s, 3H), 3.67 (s, 2H), 2.31 (s, 3H), 1.44 (s, 9H).

^{13}C{^{1}H} NMR (75 MHz, $CDCl_3$, 298 K) δ 171.4, 168.5, 156.6, 154.5, 139.5, 135.1, 134.1, 131.2, 130.5, 129.1, 126.4, 113.1, 112.6, 99.2, 79.3, 55.8, 52.3, 30.4, 29.8, 28.6, 13.7.

HRMS-ESIpos (m/z) calc'd for $C_{28}H_{31}ClN_2O_6Na$ $[M+Na]^+$, 549.1760; found, 549.1763; deviation: 0.5 ppm.

Tridecafluorooctyl benzyloxazolidinone derivative 6

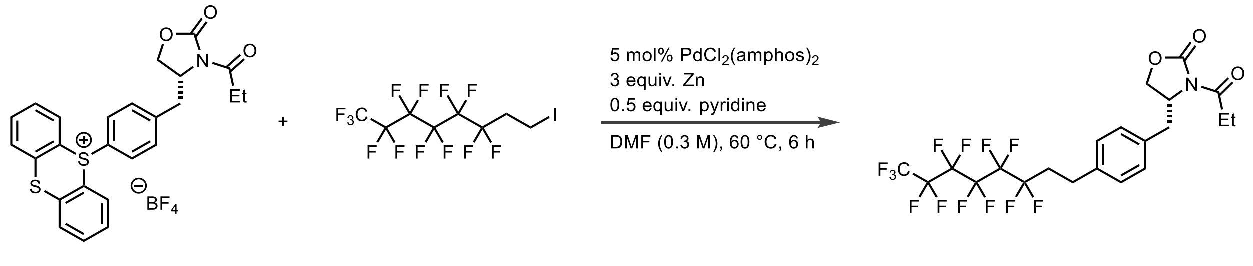

To a 4-mL borosilicate vial containing a Teflon-coated magnetic stirring bar were added benzyloxazolidinone thianthrenium salt **TT-7** (160 mg, 0.300 mmol, 1.00 equiv.), $PdCl_2(amphos)_2$ (10.6 mg, 15.0 µmol, 5.00 mol%), and activated zinc dust (58.9 mg, 0.900 mmol, 3.00 equiv.). The vial was transferred into a nitrogen-filled glovebox. Dry DMF (1 mL, c = 0.3 M) was added to the solids. Subsequently, 1*H*,1*H*,2*H*,2*H*-tridecafluoro-*n*-octyl iodide (147 µL, 284 mg, 0.600 mmol, 2.00 equiv.) and pyridine (12 µL, 12 mg, 0.15 mmol, 0.50 equiv.) were added at 25 °C. The vial was sealed with a Teflon-lined screw cap, removed from the glovebox, and transferred to a heating block preheated at 60 °C where the reaction mixture was stirred rigorously (850 rpm) for 6 h. A color change from yellow to dark brown was observed. The mixture was cooled to 25 °C, subsequently diluted with ethyl acetate (40 mL), and thereafter poured into a separatory funnel. The organic layer was washed with water (1 × 40 mL). The aqueous layer was then extracted with ethyl acetate (3 × 40 mL). The organic layers were combined, dried over $MgSO_4$, filtered, and concentrated under reduced pressure. The resulting residue was purified by column chromatography on silica gel eluting

with a solvent mixture of hexanes / ethyl acetate, (4:1 gradient to 2:3 (v/v)) to afford 107.8 mg (62%) of desired product **6** as a white solid.

R_f = 0.76 (hexanes/EtOAc, 1:1 (v:v))

NMR Spectroscopy:

^{1}H NMR (500 MHz, $CDCl_3$, 298 K) δ 7.21 – 7.14 (m, 4H), 4.66 (ddt, *J* = 9.5, 7.8, 3.1 Hz, 1H), 4.21 (dd, *J* = 9.1, 7.8 Hz, 1H), 4.15 (dd, *J* = 9.1, 2.8 Hz, 1H), 3.26 (dd, *J* = 13.4, 3.3 Hz, 1H), 3.04 – 2.85 (m, 4H), 2.78 (dd, *J* = 13.4, 9.4 Hz, 1H), 2.43 – 2.28 (m, 2H), 1.20 (t, *J* = 7.4 Hz, 3H).

^{13}C{^{1}H} NMR (126 MHz, $CDCl_3$, 298 K) δ 174.3, 153.6, 138.4, 133.9, 130.0, 129.0, 66.3, 55.3, 37.6, 33.0 (t, *J* = 22.1 Hz), 29.3, 26.2, 26.2, 8.4.

Not all carbon signals could be detected due to excessive coupling.

^{19}F NMR (471 MHz, $CDCl_3$, 298 K) δ -80.83 (t, *J* = 9.7 Hz), -114.65 (dqt, *J* = 18.3, 13.9, 4.2 Hz), -120.82 – -122.64 (m), -122.88 (tdd, *J* = 19.0, 9.4, 4.7 Hz), -123.28 – -123.76 (m), -125.91 – -126.45 (m).

HRMS-ESIpos (m/z) calc'd for $C_{21}H_{18}F_{13}NO_3Na$ $[M+Na]^+$, 602.0971; found, 602.0979; deviation: 0.5 ppm.

(Methyl)trimethylsilyl pyriproxyfen derivative 7

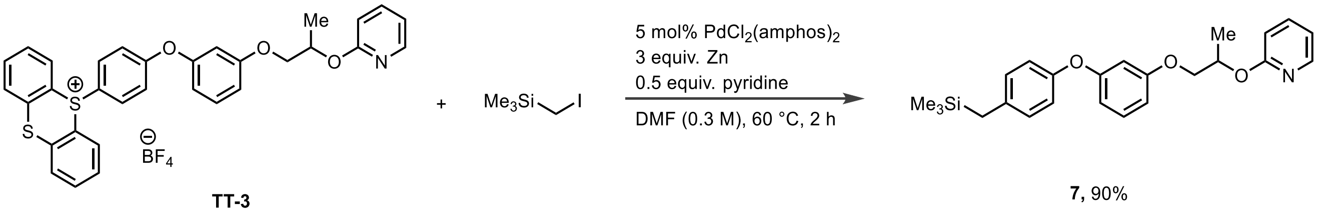

TT-3 **7,** 90%

To a 4-mL borosilicate vial containing a Teflon-coated magnetic stirring bar were added pyriproxyfen thianthrenium salt **TT-3** (187 mg, 0.300 mmol, 1.00 equiv.), $PdCl_2(amphos)_2$ (10.6 mg, 15.0 µmol, 5.00 mol%), and activated zinc dust (58.9 mg, 0.900 mmol, 3.00 equiv.). The vial was transferred into a nitrogen-filled glovebox. Dry DMF (1 mL, c = 0.3 M) was added to the solids. Subsequently, iodo(methyl)trimethylsilane (90 µL, 130 mg, 0.60 mmol, 2.0 equiv.) and pyridine (12 µL, 12 mg, 0.15 mmol, 0.50 equiv.) were added at 25 °C. The vial was sealed with a Teflon-lined screw cap, removed from the glovebox, and transferred to a heating block preheated at 60 °C where the reaction mixture was stirred rigorously (850 rpm) for 2 h. A color change from yellow to dark brown was observed. The mixture was cooled to 25 °C, subsequently diluted with ethyl acetate (40 mL), and thereafter poured into a separatory funnel. The organic layer was washed with water (1 × 40 mL). The aqueous layer was then extracted with ethyl acetate (3 × 40 mL). The organic layers were combined, dried over $MgSO_4$, filtered, and concentrated under reduced pressure. The resulting residue was purified by column chromatography on silica gel eluting with a solvent mixture of hexanes / ethyl acetate, (9:1 gradient to 4:1 (v/v)) to afford 110.4 mg (90%) of desired product **7** as a colorless oil.

Note: When the reaction was carried out on a 3 mmol scale, the concentration of the reaction mixture was 1 M. The reaction was carried out with a Schlenk line instead of the glovebox.

R_f = 0.64 (hexanes/EtOAc, 4:1 (v:v))

NMR Spectroscopy:

^{1}H NMR (500 MHz, $CDCl_3$, 298 K) δ 8.15 (ddd, *J* = 5.0, 2.1, 0.8 Hz, 1H), 7.56 (ddd, *J* = 8.4, 7.1, 2.0 Hz, 1H), 6.98 – 6.88 (m, 6H), 6.86 (ddd, *J* = 7.1, 5.0, 1.0 Hz, 1H), 6.84 – 6.81 (m, 2H), 6.74 (m, 1H), 5.59 (dtd, *J* = 11.4, 6.4, 5.0 Hz, 1H), 4.18 (dd, *J* = 9.9, 5.3 Hz, 1H), 4.07 (dd, *J* = 9.9, 4.9 Hz, 1H), 2.04 (s, 2H), 1.48 (d, *J* = 6.4 Hz, 3H), -0.01 (s, 9H).

^{13}C{^{1}H} NMR (126 MHz, $CDCl_3$, 298 K) δ 163.3, 155.0, 155.0, 151.3, 146.9, 138.8, 134.8, 129.1, 120.2, 118.0, 116.9, 115.8, 111.8, 71.2, 69.4, 26.1, 17.1, –1.8.

HRMS-ESIpos (m/z) calc'd for $C_{24}H_{30}NO_3Si$ $[M+H]^+$, 408.1989; found, 408.1989 ; deviation: 0.0 ppm.

Methyl bis(pinacolato)diboron benzyloxazolidinone derivative 8

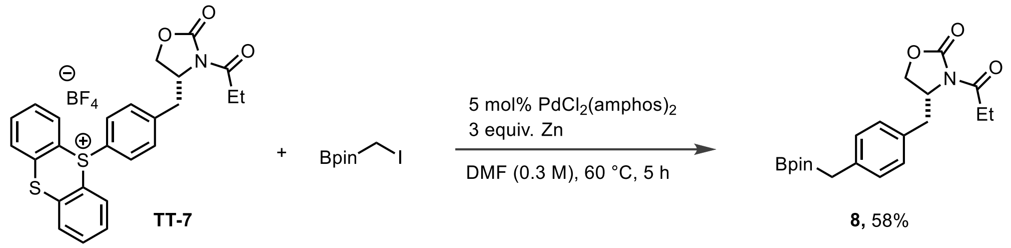

To a 4-mL borosilicate vial containing a Teflon-coated magnetic stirring bar were added benzyloxazolidinone thianthrenium salt **TT-7** (160 mg, 0.300 mmol, 1.00 equiv.), $PdCl_2(amphos)_2$ (10.6 mg, 15.0 µmol, 5.00 mol%), and activated zinc dust (58.9 mg, 0.900 mmol, 3.00 equiv.). The vial was transferred into a nitrogen-filled glovebox. Dry DMF (1 mL, c = 0.3 M) was added to the solids. Subsequently, 2-(iodomethyl)-4,4,5,5-tetramethyl-1,3,2-dioxaborolan (160 mg, 0.600 mmol, 2.00 equiv.) was added at 25 °C. The vial was sealed with a Teflon-lined screw cap, removed from the glovebox, and transferred to a heating block preheated at 60 °C where the reaction mixture was stirred rigorously (850 rpm) for 5 h. A color change from yellow to dark brown was observed. The reaction mixture was cooled to 25 °C and subsequently diluted with ethyl acetate (40 mL), and poured into a separatory funnel. The organic layer was washed with water (1 × 40 mL). The aqueous layer was then extracted with ethyl acetate (3 × 40 mL). The organic layers were combined, dried over $MgSO_4$, filtered, and concentrated under reduced pressure. The resulting residue was purified by column chromatography on silica gel eluting with a solvent mixture of hexanes / ethyl acetate, (9:1 gradient to 3:2 (v/v)) to afford 65.1 mg (58%) of desired product **8** as a colorless oil.

R_f = 0.73 (hexanes/EtOAc, 2:3 (v:v))

NMR Spectroscopy:

^{1}H NMR (500 MHz, $CDCl_3$, 298 K) δ 7.13 (d, *J* = 7.8 Hz, 2H), 7.06 (d, *J* = 7.9 Hz, 2H), 4.63 (ddt, *J* = 10.1, 6.9, 3.6 Hz, 1H), 4.22 – 4.07 (m, 2H), 3.24 (dd, *J* = 13.4, 3.3 Hz, 1H), 3.03 – 2.85 (m, 2H), 2.69 (dd, *J* = 13.4, 9.7 Hz, 1H), 2.26 (s, 2H), 1.22 (s, 12H), 1.19 (t, *J* = 7.3 Hz, 3H).

^{13}C{^{1}H} NMR (126 MHz, $CDCl_3$, 298 K) δ 174.2, 153.7, 137.9, 131.8, 129.7, 129.5, 83.6, 66.4, 55.4, 37.6, 29.3, 24.8, 8.4.

HRMS-ESIpos (m/z) calc'd for $C_{20}H_{28}BNO_5Na$ $[M+Na]^+$, 396.1953; found, 396.1952; deviation: 0.2 ppm.

Analine-*N*-boc methyl ester pyriproxyfen derivative 9

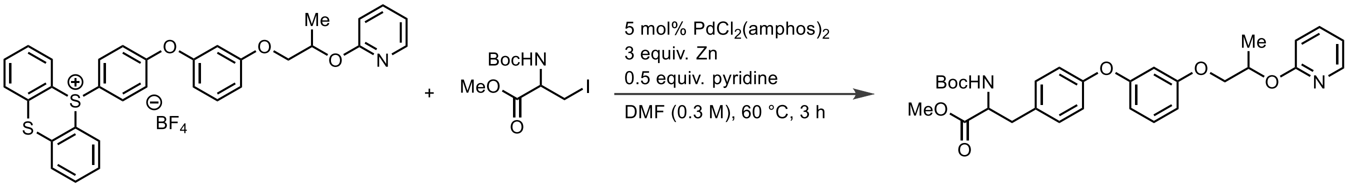

TT-3 **9**, 85%

To a 4-mL borosilicate vial containing a Teflon-coated magnetic stirring bar were added pyriproxyfen thianthrenium salt **TT-3** (187 mg, 0.300 mmol, 1.00 equiv.), $PdCl_2(amphos)_2$ (10.6 mg, 15.0 µmol, 5.00 mol%), and activated zinc dust (58.9 mg, 0.900 mmol, 3.00 equiv.). The vial was transferred into a nitrogen-filled glovebox. Dry DMF (1 mL, c = 0.3 M) was added to the solids. Subsequently, *N*-boc-3-iodo-L-alanine methyl ester (197 mg, 0.600 mmol, 2.00 equiv.) and pyridine (12 µL, 12 mg, 0.15 mmol, 0.50 equiv.) were added at 25 °C. The vial was sealed with a Teflon-lined screw cap, removed from the glovebox, and transferred to a heating block preheated at 60 °C where the reaction mixture was stirred rigorously (850 rpm) for 3 h. A color change from yellow to dark brown was observed. The mixture was cooled to 25 °C, subsequently diluted with ethyl acetate (40 mL), and thereafter poured into a separatory funnel. The organic layer was washed with water (1 × 40 mL). The aqueous layer was then extracted with ethyl acetate (3 × 40 mL). The organic layers were combined, dried over $MgSO_4$, filtered, and concentrated under reduced pressure. The resulting residue was purified by column chromatography on silica gel eluting with a solvent mixture of hexanes / ethyl acetate, (4:1 gradient to 3:2 (v/v)) to afford 133.6 mg (85%) of desired product **9** as a pale orange oil.

R_f = 0.80 (hexanes/EtOAc, 1:1 (v:v))

NMR Spectroscopy:

^{1}H NMR (500 MHz, $CDCl_3$, 298 K) δ 8.15 (dt, *J* = 5.0, 1.3 Hz, 1H), 7.56 (ddd, *J* = 8.9, 7.1, 2.0 Hz, 1H), 7.03 (d, *J* = 8.5 Hz, 2H), 6.97 – 6.90 (m, 4H), 6.86 (td, *J* = 6.0, 1.5 Hz, 3H), 6.74 (d, *J* = 8.4 Hz, 1H), 5.58 (dtd, *J* = 11.5, 6.4, 5.0 Hz, 1H), 4.98 (d, *J* = 8.3 Hz, 1H), 4.56 (q, *J* = 6.5 Hz, 1H), 4.18 (dd, *J* = 9.9, 5.3 Hz, 1H), 4.07 (dd, *J* = 9.9, 4.9 Hz, 1H), 3.71 (s, 3H), 3.13 – 2.94 (m, 2H), 1.48 (d, *J* = 6.4 Hz, 3H), 1.42 (s, 9H).

^{13}C{^{1}H} NMR (126 MHz, $CDCl_3$, 298 K) δ 172.2, 163.0, 157.5, 155.2, 155.0, 150.1, 146.7, 138.6, 130.3, 129.8, 120.7, 117.5, 116.7, 115.7, 111.6, 79.8, 70.9, 69.1, 54.4, 52.1, 37.5, 28.2, 16.9.

HRMS-ESIpos (m/z) calc'd for $C_{29}H_{34}N_2O_7Na$ [M+Na]$^+$, 545.2258; found, 545.2258; deviation: –0.0 ppm.

Oxetanyl pyriproxyfen derivative 10

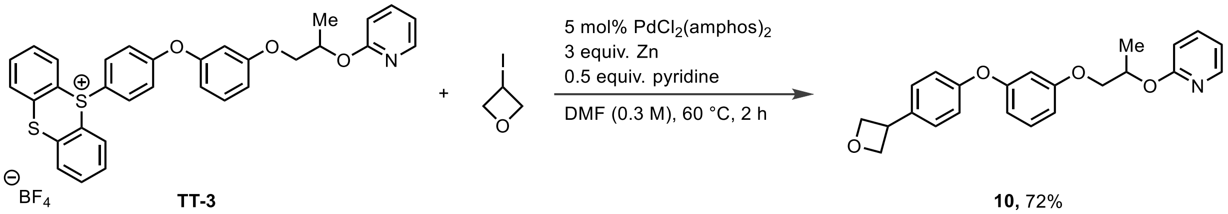

To a 4-mL borosilicate vial containing a Teflon-coated magnetic stirring bar were added pyriproxyfen thianthrenium salt **TT-3** (187 mg, 0.300 mmol, 1.00 equiv.), $PdCl_2(amphos)_2$ (10.6 mg, 15.0 µmol, 5.00 mol%), and activated zinc dust (58.9 mg, 0.900 mmol, 3.00 equiv.). The vial was transferred into a nitrogen-filled glovebox. Dry DMF (1 mL, c = 0.3 M) was added to the solids. Subsequently, 3-iodooxetane (53 µL, 110 mg, 0.60 mmol, 2.0 equiv.) and pyridine (12 µL, 12 mg, 0.15 mmol, 0.50 equiv.) were added at 25 °C. The vial was sealed with a Teflon-lined screw cap, removed from the glovebox, and transferred to a heating block preheated at 60 °C where the reaction mixture was stirred rigorously (850 rpm) for 2 h. A color change from yellow to dark brown was observed. The mixture was cooled to 25 °C, subsequently diluted with ethyl acetate (40 mL), and thereafter poured into a separatory funnel. The organic layer was washed with water (1 × 40 mL). The aqueous layer was then extracted with ethyl acetate (3 × 40 mL). The organic layers were combined, dried over $MgSO_4$, filtered, and concentrated under reduced pressure. The resulting residue was purified by column chromatography on silica gel eluting with a solvent mixture of hexanes / ethyl acetate, (4:1 gradient to 3:2 (v/v)) to afford 81.7 mg (72%) of desired product **10** as a colorless oil.

$\mathbf{R_f}$ = 0.51 (hexanes/EtOAc, 1:1 (v:v))

NMR Spectroscopy:

^{1}H NMR (500 MHz, $CDCl_3$, 298 K) δ 8.15 (ddd, *J* = 5.1, 2.0, 0.8 Hz, 1H), 7.57 (ddd, *J* = 8.4, 7.1, 2.0 Hz, 1H), 7.32 (d, *J* = 8.6 Hz, 2H), 6.99 – 6.89 (m, 6H), 6.89 – 6.82 (m, 1H), 6.74 (dt, *J* = 8.4, 0.9 Hz, 1H), 5.59 (dtd, *J* = 11.5, 6.4, 5.1 Hz, 1H), 5.06 (dd, *J* = 8.4, 6.0 Hz, 2H), 4.75 (dd, *J* = 6.8, 6.0 Hz, 2H), 4.25 – 4.15 (m, 2H), 4.08 (dd, *J* = 9.8, 4.9 Hz, 1H), 1.48 (d, *J* = 6.4 Hz, 3H).

^{13}C{^{1}H} NMR (126 MHz, $CDCl_3$, 298 K) δ 163.3, 157.7, 155.4, 150.4, 146.9, 138.8, 135.7, 128.2, 120.8, 118.0, 116.9, 116.0, 111.8, 79.3, 71.2, 69.4, 39.9, 17.1.

HRMS-ESIpos (m/z) calc'd for $C_{23}H_{24}NO_4$ [M+H]$^+$, 378.1700; found, 378.1702; deviation: –0.4 ppm.

Boc-azetidinyl salicin pentaacetate derivative 11

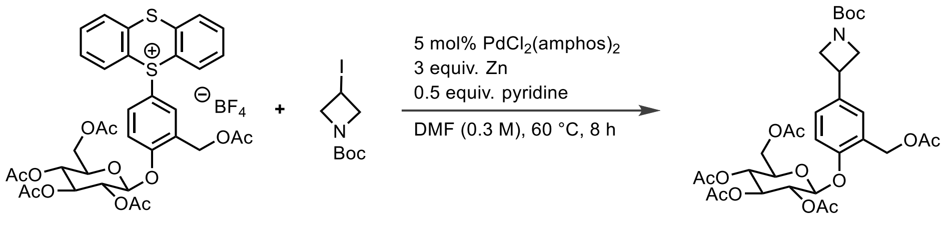

TT-8 **11,** 74%

To a 4-mL borosilicate vial containing a Teflon-coated magnetic stirring bar were added salicin pentaacetate thianthrenium salt **TT-8** (240 mg, 0.300 mmol, 1.00 equiv.), $PdCl_2(amphos)_2$ (10.6 mg, 15.0 µmol, 5.00 mol%), and activated zinc dust (58.9 mg, 0.900 mmol, 3.00 equiv.). The vial was transferred into a nitrogen-filled glovebox. Dry DMF (1 mL, c = 0.3 M) was added to the solids. Subsequently, 1-boc-3-iodoazetidine (104 µL, 169 mg, 0.600 mmol, 2.00 equiv.) and pyridine (12 µL, 12 mg, 0.15 mmol, 0.50 equiv.) were added at 25 °C. The vial was sealed with a Teflon-lined screw cap, removed from the glovebox, and transferred to a heating block preheated at 60 °C where the reaction mixture was stirred rigorously (850 rpm) for 8 h. A color change from yellow to dark brown was observed. The mixture was cooled to 25 °C, subsequently diluted with ethyl acetate (40 mL), and thereafter poured into a separatory funnel. The organic layer was washed with water (1 × 40 mL). The aqueous layer was then extracted with ethyl acetate (3 × 40 mL). The organic layers were combined, dried over $MgSO_4$, filtered, and concentrated under reduced pressure. The resulting residue was purified by column chromatography on silica gel eluting with a solvent mixture of hexanes / ethyl acetate, (3:2 gradient to 2:3 (v/v)) to afford 144 mg (74%) of desired product **11** as an pale yellow oil.

$\mathbf{R_f}$ = 0.63 (hexanes/EtOAc, 1:4 (v:v))

NMR Spectroscopy:

^{1}H NMR (500 MHz, $CDCl_3$, 298 K) δ 7.29 – 7.22 (m, 2H), 7.08 (d, *J* = 8.4 Hz, 1H), 5.33 – 5.28 (m, 2H), 5.24 – 5.15 (m, 1H), 5.11 (m, 1H), 5.09 – 5.01 (m, 2H), 4.35 – 4.23 (m, 3H), 4.20 (dd, *J* = 12.3, 2.5 Hz, 1H), 3.92 (dd, *J* = 8.6, 5.9 Hz, 2H), 3.86 (ddd, *J* = 10.0, 5.2, 2.5 Hz, 1H), 3.70 (tt, *J* = 8.8, 6.0 Hz, 1H), 2.12 – 2.07 (m, 9H), 2.07 (m, 6H), 1.47 (s, 9H).

^{13}C{^{1}H} NMR (126 MHz, $CDCl_3$, 298 K) δ 170.9, 170.5, 170.3, 170.0, 169.2, 169.1, 156.2, 153.3, 137.4, 128.0, 127.4, 126.3, 116.3, 99.3, 79.4, 72.4, 71.9, 70.8, 68.1, 61.7, 60.8, 60.2, 32.7, 28.2, 20.8, 20.8, 20.5, 20.4, 14.0.

HRMS-ESIpos (m/z) calc'd for $C_{31}H_{41}NO_{14}Na$ $[M+Na]^+$, 674.2420; found, 674.2417; deviation: 0.4 ppm.

Boc-azetidinyl strychnine derivative 12

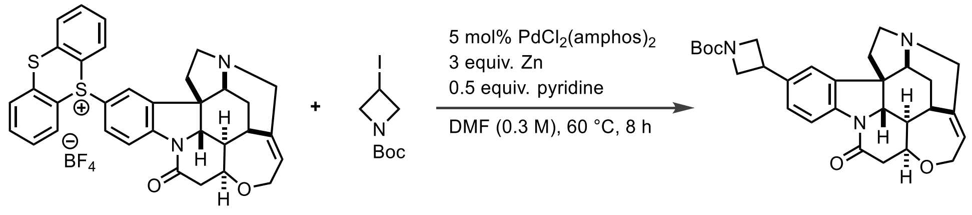

TT-9 **12,** 60%

To a 4-mL borosilicate vial containing a Teflon-coated magnetic stirring bar were added strychnine thianthrenium salt **TT-9** (191 mg, 0.300 mmol, 1.00 equiv.), $PdCl_2(amphos)_2$ (10.6 mg, 15 µmol, 5.00 mol%), and activated zinc dust (58.9 mg, 0.900 mmol, 3.00 equiv.). The vial was transferred into a nitrogen-filled glovebox. Dry DMF (1 mL, c = 0.3 M) was added to the solids. Subsequently, 1-boc-3-iodoazetidine (104 µL, 169 mg, 0.600 mmol, 2.00 equiv.) and pyridine (12 µL, 12 mg, 0.15 mmol, 0.50 equiv.) were added at 25 °C. The vial was sealed with a Teflon-lined screw cap, removed from the glovebox, and transferred to a heating block preheated at 60 °C where the reaction mixture was stirred rigorously (850 rpm) for 8 h. A color change from yellow to dark brown was observed. The mixture was cooled to 25 °C, subsequently diluted with ethyl acetate (40 mL), and thereafter poured into a separatory funnel. The organic layer was washed with water (1 × 40 mL). The aqueous layer was then extracted with ethyl acetate (3 × 40 mL). The organic layers were combined, dried over $MgSO_4$, filtered, and concentrated under reduced pressure. The resulting residue was purified by column chromatography on silica gel eluting with a solvent mixture of dichloromethane / Et_3N, (9:1 (v/v)) to afford 88.5 mg (60%) of desired product **12** as an off-white foam.

$\mathbf{R_f}$ = 0.70 (dichloromethane/Et_3N, 9:1 (v:v))

NMR Spectroscopy:

^{1}**H NMR** (500 MHz, $CDCl_3$, 298 K) δ 8.04 (d, *J* = 8.2 Hz, 1H), 7.19 (dd, *J* = 8.3, 1.9 Hz, 1H), 7.06 (m, 1H), 5.94 – 5.88 (m, 1H), 4.34 – 4.24 (m, 3H), 4.18 – 4.00 (m, 3H), 3.97 – 3.84 (m, 3H), 3.86 (d, *J* = 10.5 Hz, 1H), 3.76 – 3.57 (m, 3H), 3.21 (m, 1H), 3.16 – 3.06 (m, 2H), 2.91 – 2.81 (m, 1H), 2.72 (d, *J* = 14.8 Hz, 1H), 2.65 (dd, *J* = 17.4, 3.3 Hz, 1H), 2.36 (dt, *J* = 14.5, 4.4 Hz, 1H), 1.91 – 1.85 (m, 2H), 1.46 (s, 9H).

^{13}**C{^{1}H} NMR** (75 MHz, $CDCl_3$, 298 K) δ 169.1, 156.5, 141.2, 138.7, 138.3, 132.1, 129.8, 127.5, 120.8, 116.6, 79.7, 77.6, 70.7, 64.6, 60.5, 60.2, 56.7, 52.6, 52.1, 50.4, 48.0, 42.5, 42.5, 33.4, 31.4, 29.7, 28.5, 27.2, 26.7.

HRMS-ESIpos (m/z) calc'd for $C_{29}H_{36}N_3O_4$ $[M+H]^+$, 490.2700; found, 490.2700; deviation: –0.0 ppm.

Methylene cyclobutane carboxylate pyriproxyfen derivative 13

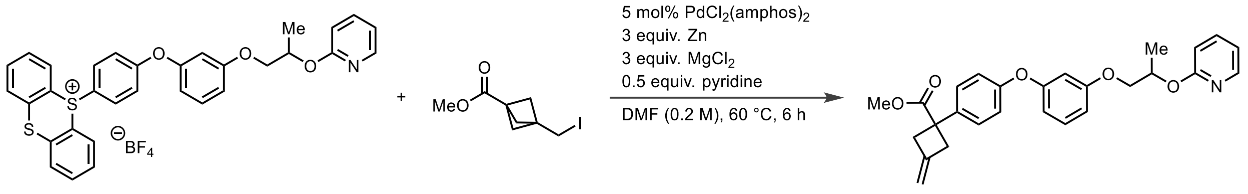

TT-3 **13**, 83%

To a 4-mL borosilicate vial containing a Teflon-coated magnetic stirring bar were added pyriproxyfen thianthrenium salt **TT-3** (124 mg, 0.200 mmol, 1.00 equiv.), $PdCl_2(amphos)_2$ (7.1 mg, 10 µmol, 5.0 mol%), activated zinc dust (39.2 mg, 0.600 mmol, 3.00 equiv.), and $MgCl_2$ (57.1 mg, 0.600 mmol, 3.00 equiv.). The vial was transferred into a nitrogen-filled glovebox. Dry DMF (1 mL, c = 0.2 M) was added to the solids. Subsequently, methyl 4-(iodomethyl)bicyclo[1.1.1]pentane-2-carboxylate (106 mg, 0.400 mmol, 2.00 equiv.) and pyridine (8 µL, 8 mg, 0.1 mmol, 0.5 equiv.) were added at 25 °C. The vial was sealed with a Teflon-lined screw cap, removed from the glovebox, and transferred to a heating block preheated at 60 °C where the reaction mixture was stirred rigorously (850 rpm) for 6 h. A color change from yellow to dark brown was observed. The mixture was cooled to 25 °C, subsequently diluted with ethyl acetate (40 mL), and thereafter poured into a separatory funnel. The organic layer was washed with water (1 × 40 mL). The aqueous layer was then extracted with ethyl acetate (3 × 40 mL). The organic layers were combined, dried over $MgSO_4$, filtered, and concentrated under reduced pressure. The resulting residue was purified by column chromatography on silica gel eluting with a solvent mixture of hexanes / ethyl acetate, (1:0 gradient to 4:1 (v/v)) to afford 76.3 mg (83%) of desired product **13** as a colorless oil.

$\mathbf{R_f}$ = 0.53 (hexanes/EtOAc, 4:1 (v:v))

NMR Spectroscopy:

^{1}H NMR (500 MHz, $CDCl_3$, 298 K) δ 8.15 (dd, *J* = 5.1, 2.0 Hz, 1H), 7.61 – 7.53 (m, 1H), 7.02 (d, *J* = 8.6 Hz, 2H), 6.95 – 6.89 (m, 4H), 6.90 – 6.84 (m, 1H), 6.82 (d, *J* = 8.7 Hz, 2H), 6.74 (d, *J* = 8.2 Hz, 1H), 5.58 (dtd, *J* = 11.5, 6.4, 5.0 Hz, 1H), 4.91 – 4.74 (m, 2H), 4.18 (dd, *J* = 9.9, 5.3 Hz, 1H), 4.07 (dd, *J* = 9.9, 4.8 Hz, 1H), 3.67 (s, 3H), 3.14 – 3.03 (m, 4H), 2.72 – 2.64 (m, 2H), 1.48 (d, *J* = 6.4 Hz, 3H).

^{13}C{^{1}H} NMR (126 MHz, $CDCl_3$, 298 K) δ 176.4, 163.3, 157.4, 155.3, 150.4, 146.9, 142.6, 138.9, 132.1, 130.4, 120.8, 117.5, 116.9, 115.9, 111.8, 108.2, 71.2, 69.4, 52.0, 44.6, 42.1, 40.4, 17.1.

HRMS-ESIpos (m/z) calc'd for $C_{28}H_{29}NO_5Na$ $[M+Na]^+$, 482.1938; found, 482.1936; deviation: 0.4 ppm.

Oxaspiro[3.3]heptanyl pyriproxyfen derivative 14

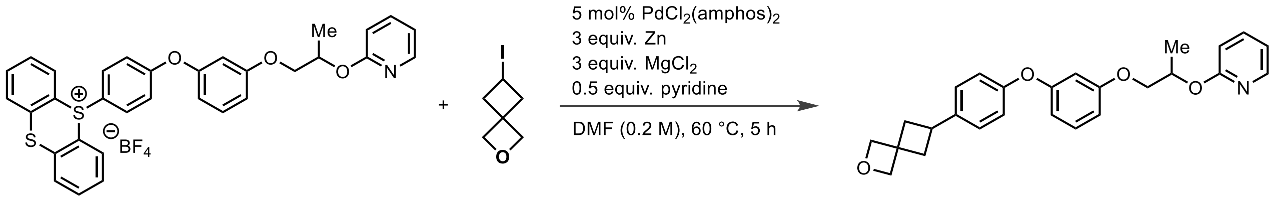

TT-3 **14,** 66%

To a 4-mL borosilicate vial containing a Teflon-coated magnetic stirring bar were added pyriproxyfen thianthrenium salt **TT-3** (125 mg, 0.200 mmol, 1.00 equiv.), $PdCl_2(amphos)_2$ (7.1 mg, 10 µmol, 5.0 mol%), activated zinc dust (39.2 mg, 0.600 mmol, 3.00 equiv.), and $MgCl_2$ (57.1 mg, 0.600 mmol, 3.00 equiv.). The vial was transferred into a nitrogen-filled glovebox. Dry DMF (1 mL, c = 0.2 M) was added to the solids. Subsequently, 6-iodo-2-oxaspiro[3.3]heptane (89.6 mg, 0.400 mmol, 2.00 equiv.) and pyridine (8 µL, 8 mg, 0.1 mmol, 0.5 equiv.) were added at 25 °C. The vial was sealed with a Teflon-lined screw cap, removed from the glovebox, and transferred to a heating block preheated at 60 °C where the reaction mixture was stirred rigorously (850 rpm) for 5 h. A color change from yellow to dark brown was observed. The mixture was cooled to 25 °C, subsequently diluted with ethyl acetate (40 mL), and thereafter poured into a separatory funnel. The organic layer was washed with water (1 × 40 mL). The aqueous layer was then extracted with ethyl acetate (3 × 40 mL). The organic layers were combined, dried over $MgSO_4$, filtered, and concentrated under reduced pressure. The resulting residue was purified by column chromatography on silica gel eluting with a solvent mixture of hexanes / ethyl acetate, (4:1 gradient to 3:2 (v/v)) to afford 55.1 mg (66%) of desired product **14** as a colorless oil.

$\mathbf{R_f}$ = 0.51 (hexanes/EtOAc, 1:1 (v:v))

NMR Spectroscopy:

^{1}H NMR (500 MHz, $CDCl_3$, 298 K) δ 8.15 (ddd, *J* = 5.0, 2.0, 0.8 Hz, 1H), 7.56 (ddd, *J* = 8.4, 7.1, 2.0 Hz, 1H), 7.11 – 7.04 (m, 2H), 6.96 – 6.89 (m, 4H), 6.89 – 6.84 (m, 3H), 6.74 (d, *J* = 8.3 Hz, 1H), 5.58 (dtd, *J* = 11.5, 6.4, 5.0 Hz, 1H), 4.83 (s, 2H), 4.62 (s, 2H), 4.18 (dd, *J* = 9.9, 5.3 Hz, 1H), 4.06 (dd, *J* = 9.9, 4.9 Hz, 1H), 3.47 – 3.13 (m, 1H), 2.71 – 2.62 (m, 2H), 2.31 – 2.22 (m, 2H), 1.48 (d, *J* = 6.4 Hz, 3H).

^{13}C{^{1}H} NMR (126 MHz, $CDCl_3$, 298 K) δ 163.0, 156.5, 155.0, 150.4, 146.6, 138.8, 138.6, 127.2, 120.3, 117.5, 116.6, 115.6, 111.6, 84.6, 82.5, 70.9, 69.2, 39.7, 39.3, 33.2, 16.9.

HRMS-ESIpos (m/z) calc'd for $C_{26}H_{28}NO_4$ $[M+H]^+$, 418.2013; found, 418.2017; deviation: –1.0 ppm.

Methyl fenofibrate derivative 15

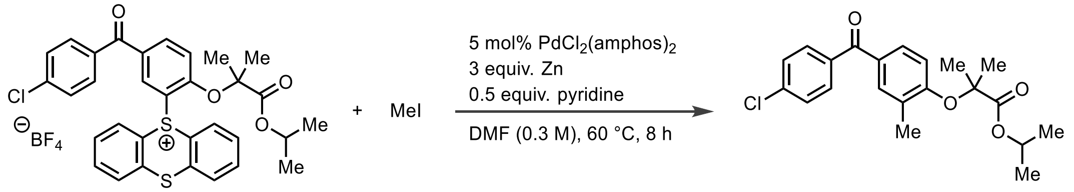

TT-10 **15,** 57%

To a 4-mL borosilicate vial containing a Teflon-coated magnetic stirring bar were added fenofibrate thianthrenium salt **TT-10** (199 mg, 0.300 mmol, 1.00 equiv.), $PdCl_2(amphos)_2$ (10.6 mg, 15.0 µmol, 5.00 mol%.), and activated zinc dust (58.9 mg, 0.900 mmol, 3.00 equiv.). The vial was transferred into a nitrogen-filled glovebox. Dry DMF (1 mL, c = 0.3 M) was added to the solids. Subsequently, methyl iodide (37 µL, 85 mg, 0.60 mmol, 2.0 equiv.) and pyridine (12 µL, 12 mg, 0.15 mmol, 0.50 equiv.) were added at 25 °C. The vial was sealed with a Teflon-lined screw cap, removed from the glovebox, and transferred to a heating block preheated at 60 °C where the reaction mixture was stirred rigorously (850 rpm) for 8 h. A color change from yellow to dark brown was observed. The mixture was cooled to 25 °C, subsequently diluted with ethyl acetate (40 mL), and thereafter poured into a separatory funnel. The organic layer was washed with water (1 × 40 mL). The aqueous layer was then extracted with ethyl acetate (3 × 40 mL). The organic layers were combined, dried over $MgSO_4$, filtered, and concentrated under reduced pressure. The resulting residue was purified by column chromatography on silica gel eluting with a solvent mixture of hexanes / ethyl acetate, (9:1 gradient to 7:3 (v/v)) to afford 64.2 mg (57%) of desired product **15** as a yellow oil.

R_f = 0.65 (hexanes/EtOAc, 4:1 (v:v))

NMR Spectroscopy:

^{1}H NMR (500 MHz, $CDCl_3$, 298 K) δ 7.69 (d, J = 8.5 Hz, 2H), 7.65 (dd, J = 2.2, 0.9 Hz, 1H), 7.51 (dd, J = 8.5, 2.3 Hz, 1H), 7.44 (d, J = 8.5 Hz, 2H), 6.65 (d, J = 8.5 Hz, 1H), 5.08 (p, J = 6.3 Hz, 1H), 2.27 (s, 3H), 1.66 (s, 6H), 1.20 (d, J = 6.3 Hz, 6H).

^{13}C{^{1}H} NMR (126 MHz, $CDCl_3$, 298 K) δ 194.7, 173.4, 158.3, 138.3, 136.7, 133.1, 131.3, 129.9, 129.5, 129.1, 128.6, 114.3, 79.6, 69.4, 25.6, 21.6, 16.9.

HRMS-ESIpos (m/z) calc'd for $C_{21}H_{23}ClO_4Na$ $[M+Na]^+$, 397.1177; found, 397.1178; deviation: – 0.1 ppm.

2-Methylpropanyl indomethacin methyl ester derivative 16

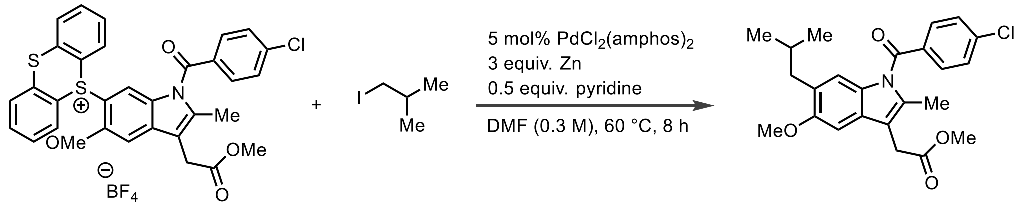

TT-6 **16,** 48%

To a 4-mL borosilicate vial containing a Teflon-coated magnetic stirring bar were added indomethacin methyl ester thianthrenium salt **TT-6** (202 mg, 0.300 mmol, 1.00 equiv.), $PdCl_2(amphos)_2$ (10.6 mg, 15.0 µmol, 5.00 mol%), and activated zinc dust (58.9 mg, 0.900 mmol, 3.00 equiv.). The vial was transferred into a nitrogen-filled glovebox. Dry DMF (1 mL, c = 0.3 M) was added to the solids. Subsequently, 1-iodo-2-methylpropane (69 µL, 110 mg, 0.60 mmol, 2.0 equiv.) and pyridine (12 µL, 12 mg, 0.15 mmol, 0.50 equiv.) were added at 25 °C. The vial was sealed with a Teflon-lined screw cap, removed from the glovebox, and transferred to a heating block preheated at 60 °C where the reaction mixture was stirred rigorously (850 rpm) for 8 h. A color change from yellow to dark brown was observed. The mixture was cooled to 25 °C, subsequently diluted with ethyl acetate (40 mL), and thereafter poured into a separatory funnel. The organic layer was washed with water (1 × 40 mL). The aqueous layer was then extracted with ethyl acetate (3 × 40 mL). The organic layers were combined, dried over $MgSO_4$, filtered, and concentrated under reduced pressure. The resulting residue was purified by column chromatography on silica gel eluting with a solvent mixture of hexanes / ethyl acetate, (1:0 gradient to 4:1 (v/v)) to afford 62.0 mg (48%) of desired product **16** as a yellow solid.

R_f = 0.48 (hexanes/EtOAc, 4:1 (v:v))

NMR Spectroscopy:

^{1}H NMR (500 MHz, $CDCl_3$, 298 K) δ 7.66 (d, *J* = 8.5 Hz, 2H), 7.46 (d, *J* = 8.5 Hz, 2H), 6.88 (s, 1H), 6.65 (s, 1H), 3.85 (s, 3H), 3.71 (s, 3H), 3.67 (s, 2H), 2.38 (s, 3H), 2.36 (d, *J* = 7.1 Hz, 2H), 1.76 (hept, *J* = 6.8 Hz, 1H), 0.79 (d, *J* = 6.7 Hz, 6H).

^{13}C{^{1}H} NMR (126 MHz, $CDCl_3$, 298 K) δ 171.6, 168.5, 154.7, 139.2, 134.7, 134.2, 131.3, 130.4, 129.2, 128.5, 126.8, 116.5, 112.6, 99.1, 55.8, 52.3, 40.2, 30.4, 28.8, 22.6, 13.4.

HRMS-ESIpos (m/z) calc'd for $C_{24}H_{26}ClNO_4Na$ $[M+Na]^+$, 450.1442; found, 450.1443; deviation: 0.1 ppm.

Boc-piperidinyl pyriproxyfen derivative 17

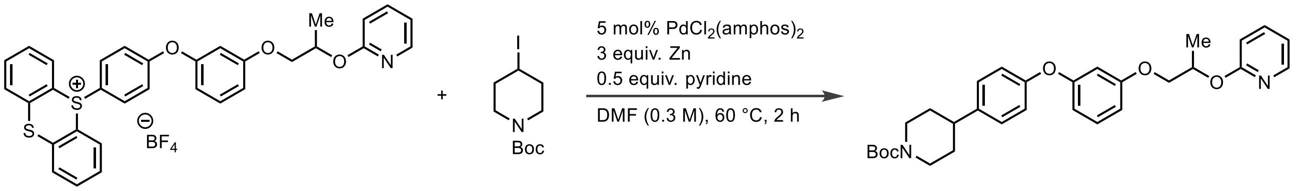

TT-3 **17,** 68%

To a 4-mL borosilicate vial containing a Teflon-coated magnetic stirring bar were added pyriproxyfen thianthrenium salt **TT-3** (187 mg, 0.300 mmol, 1.00 equiv.), $PdCl_2(amphos)_2$ (10.6 mg, 15.0 µmol, 5.00 mol%), and activated zinc dust (58.9 mg, 0.900 mmol, 3.00 equiv.). The vial was transferred into a nitrogen-filled glovebox. Dry DMF (1 mL, c = 0.3 M) was added to the solids. Subsequently, 1-boc-4-iodopiperidine (186 mg, 0.600 mmol, 2.00 equiv.) and pyridine (12 µL, 12 mg, 0.15 mmol, 0.50 equiv.) were added at 25 °C. The vial was sealed with a Teflon-lined screw cap, removed from the glovebox, and transferred to a heating block heated at 60 °C where the reaction mixture was stirred rigorously (850 rpm) for 2 h. A color change from yellow to dark brown was observed. The mixture was cooled to 25 °C, subsequently diluted with ethyl

acetate (40 mL), and thereafter poured into a separatory funnel. The organic layer was washed with water (1 × 40 mL). The aqueous layer was then extracted with ethyl acetate (3 × 40 mL). The organic layers were combined, dried over $MgSO_4$, filtered, and concentrated under reduced pressure. The resulting residue was purified by column chromatography on silica gel eluting with a solvent mixture of hexane / ethyl acetate, (9:1 gradient to 4:1 (v/v)) to afford 103.5 mg (68%) of desired product **17** as a colorless oil.

R_f = 0.57 (hexanes/EtOAc, 4:1 (v:v))

NMR Spectroscopy:

^{1}H NMR (500 MHz, $CDCl_3$, 298 K) δ 8.14 (ddd, *J* = 5.1, 2.1, 0.9 Hz, 1H), 7.55 (ddd, *J* = 8.3, 7.1, 2.0 Hz, 1H), 7.11 (d, *J* = 8.7 Hz, 2H), 6.98 – 6.80 (m, 7H), 6.73 (d, *J* = 8.3 Hz, 1H), 5.58 (dtd, *J* = 11.5, 6.4, 5.0 Hz, 1H), 4.18 (m, 3H), 4.06 (dd, *J* = 9.9, 4.9 Hz, 1H), 2.80 (d, *J* = 13.5 Hz, 2H), 2.60 (tt, *J* = 12.2, 3.6 Hz, 1H), 1.83 – 1.76 (m, 2H), 1.68 – 1.50 (m, 2H), 1.47 (m, 12H).

^{13}C{^{1}H} NMR (126 MHz, $CDCl_3$, 298 K) δ 163.3, 157.0, 155.3, 155.0, 150.6, 146.8, 140.1, 138.9, 127.9, 120.8, 117.8, 116.9, 115.9, 111.9, 79.6, 71.2, 69.5, 42.1, 33.5, 28.6, 17.1.

HRMS-ESIpos (m/z) calc'd for $C_{30}H_{37}N_2O_5$ $[M+H]^+$, 505.2698; found, 505.2697; deviation: –0.1 ppm.

Fmoc-Arg(Pbf)-OH pyriproxyfen derivative 18

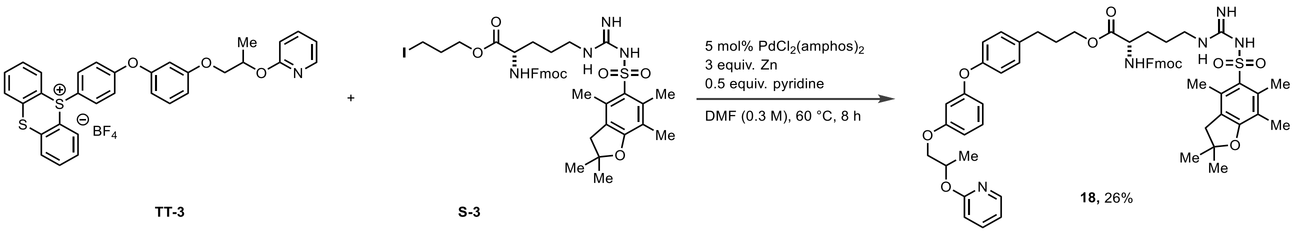

TT-3 S-3 **18**, 26%

To a 4-mL borosilicate vial containing a Teflon-coated magnetic stirring bar were added pyriproxyfen thianthrenium salt **TT-3** (187 mg, 0.300 mmol, 1.00 equiv.), $PdCl_2(amphos)_2$ (10.6 mg, 15.0 µmol, 5.00 mol%), and activated zinc dust (58.9 mg, 0.900 mmol, 3.00 equiv.). The vial was transferred into a nitrogen-filled glovebox. Dry DMF (1 mL, c = 0.3 M) was added to the solids. Subsequently, Fmoc-Arg(Pbf)-OH iodide derivative **S-3** (490 mg, 0.600 mmol, 2.00 equiv.) and pyridine (12 µL, 12 mg, 0.15 mmol, 0.50 equiv.) were added at 25 °C. The vial was sealed with a Teflon-lined screw cap, removed from the glovebox, and transferred to a heating block preheated at 60 °C where the reaction mixture was stirred rigorously (850 rpm) for 8 h. A color change from yellow to dark brown was observed. The mixture was cooled to 25 °C, subsequently diluted with ethyl acetate (40 mL), and thereafter poured into a separatory funnel. The organic layer was washed with water (1 × 40 mL). The aqueous layer was then extracted with ethyl acetate (3 × 40 mL). The organic layers were combined, dried over $MgSO_4$, filtered, and concentrated under reduced pressure. The resulting residue was purified by column chromatography on silica gel eluting with a solvent mixture of hexanes / ethyl acetate, (5:5 gradient to 1:4 (v/v)) to afford 112 mg (37%) of product **18** and remaining starting material **S-3** as a pale brown oil. Further purification by HPLC (YMC Pack Pro C18 (150

mm × 4.6 mm: 5 μm), MeCN / water = 75:25, flow rate = 1 mL/min, 25 °C) provided 78.1 mg (26%) of desired product **18** as an off-white foam.

R_f = 0.59 (hexanes/EtOAc, 1:4 (v:v))

NMR Spectroscopy:

^{1}H NMR (500 MHz, $CDCl_3$, 298 K) δ 8.14 (dd, *J* = 5.3, 2.0 Hz, 1H), 7.73 (d, *J* = 7.6 Hz, 2H), 7.60 – 7.49 (m, 3H), 7.36 (td, *J* = 7.4, 3.1 Hz, 2H), 7.30 – 7.21 (m, 2H), 7.05 (d, *J* = 8.1 Hz, 2H), 6.94 – 6.88 (m, 4H), 6.87 – 6.81 (m, 3H), 6.74 (dd, *J* = 8.4, 1.0 Hz, 1H), 6.13 (s, 2H), 5.66 – 5.53 (m, 2H), 4.33 (dd, *J* = 40.6, 6.3 Hz, 3H), 4.22 – 4.10 (m, 4H), 4.06 (dd, *J* = 9.8, 4.8 Hz, 1H), 3.67 – 3.62 (m, 2H), 3.32 – 3.10 (m, 2H), 2.89 (s, 2H), 2.58 (d, *J* = 15.5 Hz, 4H), 2.50 (s, 3H), 2.06 (s, 3H), 1.92 (p, *J* = 6.9 Hz, 2H), 1.84 (d, *J* = 7.8 Hz, 1H), 1.63 (dp, *J* = 37.0, 7.4 Hz, 3H), 1.48 (d, *J* = 6.4 Hz, 3H), 1.41 (s, 6H).

^{13}C{^{1}H} NMR (75 MHz, $CDCl_3$, 298 K) δ 172.2, 163.3, 159.0, 156.9, 156.2, 155.3, 150.7, 146.9, 143.8, 143.7, 141.4, 138.8, 138.6, 135.0, 132.5, 129.6, 127.9, 127.3, 125.2, 124.8, 120.6, 120.1, 117.9, 117.7, 116.9, 115.9, 111.8, 86.5, 71.2, 69.5, 67.3, 65.2, 47.2, 43.3, 40.9, 31.4, 30.3, 29.8, 28.7, 25.3, 19.4, 18.0, 17.1, 12.6.

HRMS-ESIpos (m/z) calc'd for $C_{57}H_{64}O_{10}N_5S$ $[M+H]^+$, 1010.4368; found, 1010.4367; deviation: 0.2 ppm.

Sulbactam nefiractam derivative 19

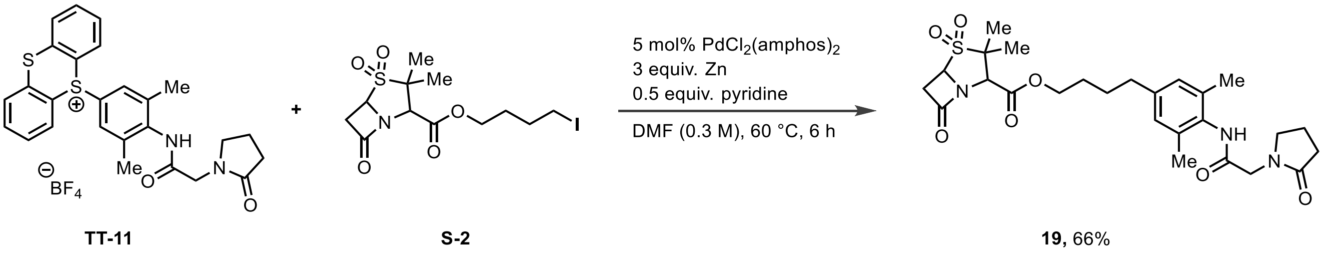

To a 4-mL borosilicate vial containing a Teflon-coated magnetic stirring bar were added nefiractam thianthrenium salt **TT-11** (165 mg, 0.300 mmol, 1.00 equiv.), $PdCl_2(amphos)_2$ (10.6 mg, 15 µmol, 5.00 mol%), and activated zinc dust (58.9 mg, 0.900 mmol, 3.00 equiv.). The vial was transferred into a nitrogen-filled glovebox. Dry DMF (1 mL, c = 0.3 M) was added to the solids. Subsequently, sulbactam iodide derivative **S-2** (249 mg, 0.600 mmol, 2.00 equiv.) and pyridine (12 µL, 12 mg, 0.15 mmol, 0.50 equiv.) were added at 25 °C. The vial was sealed with a Teflon-lined screw cap, removed from the glovebox, and transferred to a heating block preheated at 60 °C where the reaction mixture was stirred rigorously (850 rpm) for 6 h. A color change from yellow to dark brown was observed. The mixture was cooled to 25 °C, subsequently diluted with ethyl acetate (40 mL), and thereafter poured into a separatory funnel. The organic layer was washed with water (1 × 40 mL). The aqueous layer was then extracted with ethyl acetate (3 × 40 mL). The organic layers were combined, dried over $MgSO_4$, filtered, and concentrated under reduced pressure. The resulting residue was purified by column chromatography on silica gel eluting with a solvent mixture of dichloromethane / *i*-PrOH, (1:0 gradient to 4:1 (v/v)) to afford 104.9 mg (66%) of desired product **19** as a white solid.

R$_f$ = 0.40 (dichloromethane/*i*-PrOH, 9:1 (v:v))

NMR Spectroscopy:

^{1}H NMR (600 MHz, $CDCl_3$, 298 K) δ 7.62 (s, 1H), 6.87 (s, 2H), 4.60 (dd, *J* = 4.3, 2.1 Hz, 1H), 4.36 (s, 1H), 4.26 – 4.17 (m, 2H), 4.10 (s, 2H), 3.61 (t, *J* = 7.1 Hz, 2H), 3.55 – 3.31 (m, 2H), 2.56 (t, *J* = 7.1 Hz, 2H), 2.47 (t, *J* = 8.1 Hz, 2H), 2.17 (s, 6H), 2.16 – 2.11 (m, 2H), 1.74 – 1.62 (m, 4H), 1.60 (s, 3H), 1.39 (s, 3H).

^{13}C{^{1}H} NMR (151 MHz, $CDCl_3$, 298 K) δ 176.3, 170.7, 167.1, 166.9, 140.7, 135.1, 131.1, 128.2, 66.3, 63.3, 62.6, 61.0, 48.7, 47.9, 38.2, 34.7, 30.3, 27.9, 27.4, 20.3, 18.6, 18.3, 18.2.

HRMS-ESIpos (m/z) calc'd for $C_{26}H_{35}N_3O_7SNa$ [M+Na]$^+$, 556.2088; found, 556.2093; deviation: –1.0 ppm.

Sulbactam estrone methyl ether derivative 20

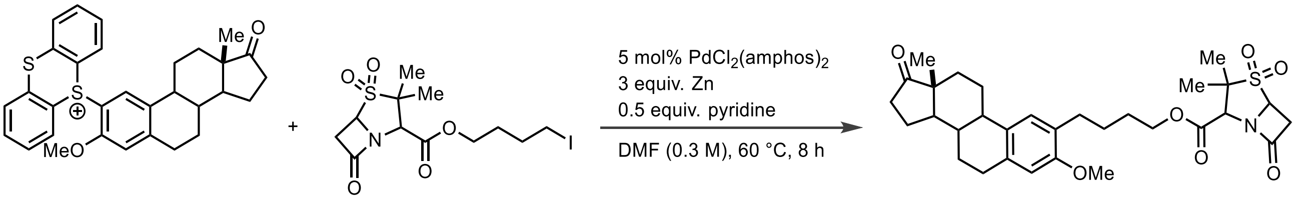

TT-4 **S-2** **20,** 50%

To a 4-mL borosilicate vial containing a Teflon-coated magnetic stirring bar were added estrone methyl ether derived thianthrenium salt **TT-4** (172 mg, 0.300 mmol, 1.00 equiv.), $PdCl_2$(amphos)$_2$ (10.6 mg, 15.0 µmol, 5.00 mol%.), and activated zinc dust (58.9 mg, 0.900 mmol, 3.00 equiv.). The vial was transferred into a nitrogen-filled glovebox. Dry DMF (1 mL, c = 0.3 M) was added to the solids. Subsequently, sulbactam iodide derivative **S-2** (249 mg, 0.600 mmol, 2.00 equiv.) and pyridine (12 µL, 12 mg, 0.15 mmol, 0.50 equiv.) were added at 25 °C. The vial was sealed with a Teflon-lined screw cap, removed from the glovebox, and transferred to a heating block preheated at 60 °C where the reaction mixture was stirred rigorously (850 rpm) for 8 h. A color change from yellow to dark brown was observed. The mixture was cooled to 25 °C, subsequently diluted with ethyl acetate (40 mL), and thereafter poured into a separatory funnel. The organic layer was washed with water (1 × 40 mL). The aqueous layer was then extracted with ethyl acetate (3 × 40 mL). The organic layers were combined, dried over $MgSO_4$, filtered, and concentrated under reduced pressure. The resulting residue was purified by column chromatography on silica gel eluting with a solvent mixture of dichloromethane / *i*-PrOH, (1:0 gradient to 9:1 (v/v)) to afford 130.2 mg (76%) as a mixture of desired product **20** and remaining starting material **S-2** as a yellow oil. Further purification by HPLC (YMC Pack Pro C18 (150 mm × 4.6 mm: 5 µm), MeCN / water = 80:20, flow rate = 1 mL/min, 25 °C) provided 85.6 mg (50%) of desired product **20** as an off-white foam.

R$_f$ = 0.67 (hexanes/EtOAc, 2:3 (v:v))

NMR Spectroscopy:

^{1}H NMR (300 MHz, $CDCl_3$, 298 K) δ 7.03 (s, 1H), 6.58 (s, 1H), 4.60 (dd, *J* = 3.9, 2.5 Hz, 1H), 4.37 (s, 1H), 4.23 (t, *J* = 6.3 Hz, 2H), 3.78 (s, 3H), 3.46 (t, *J* = 3.1 Hz, 2H), 2.90 (dd, *J* = 10.6, 5.2 Hz, 2H), 2.72 – 2.35 (m, 4H), 2.30 – 1.92 (m, 5H), 1.79 – 1.45 (m, 13H), 1.40 (s, 3H), 0.91 (s, 3H).

^{13}C{^{1}H} NMR (126 MHz, $CDCl_3$, 298 K) δ 221.1, 170.8, 167.1, 155.6, 135.5, 131.6, 127.6, 127.1, 110.9, 66.7, 63.4, 62.8, 61.2, 55.4, 50.5, 48.2, 44.1, 38.6, 38.5, 36.0, 31.8, 29.9, 29.7, 28.5, 26.8, 26.6, 26.2, 21.7, 20.5, 18.7, 14.0.

HRMS-ESIpos (m/z) calc'd for $C_{31}H_{41}NO_7SNa$ $[M+Na]^+$, 594.2496; found, 594.2493; deviation: 0.5 ppm.

Epiandrosterone flurbiprofen methyl ester derivative 21

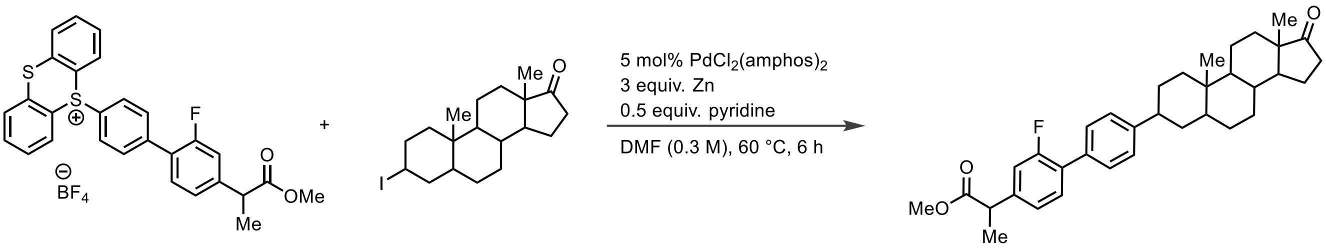

TT-12 **S-4** **21,** 60%

To a 4-mL borosilicate vial containing a Teflon-coated magnetic stirring bar were added flurbiprofen methyl ester thianthrenium salt **TT-12** (168 mg, 0.300 mmol, 1.00 equiv.), $PdCl_2(amphos)_2$ (10.6 mg, 15.0 µmol, 5.00 mol%.), and activated zinc dust (58.9 mg, 0.900 mmol, 3.00 equiv.). The vial was transferred into a nitrogen-filled glovebox. Dry DMF (1 mL, c = 0.3 M) was added to the solids. Subsequently, 3-iodo epiandrosterone derivative **S-4** (240 mg, 0.600 mmol, 2.00 equiv.) and pyridine (12 µL, 12 mg, 0.15 mmol, 0.50 equiv.) were added at 25 °C. The vial was sealed with a Teflon-lined screw cap, removed from the glovebox, and transferred to a heating block heated at 60 °C where the reaction mixture was stirred rigorously (850 rpm) for 6 h. A color change from yellow to dark brown was observed. The mixture was cooled to 25 °C, subsequently diluted with ethyl acetate (40 mL), and thereafter poured into a separatory funnel. The organic layer was washed with water (1 × 40 mL). The aqueous layer was then extracted with ethyl acetate (3 × 40 mL). The organic layers were combined, dried over $MgSO_4$, filtered, and concentrated under reduced pressure. The resulting residue was purified by column chromatography on silica gel eluting with a solvent mixture of hexanes / ethyl acetate, (1:0 gradient to 9:1 (v/v)) to afford 96.1 mg (60%) of desired product **21** as a white powder.

R_f = 0.58 (hexanes/EtOAc, 4:1 (v:v))

NMR Spectroscopy:

^{1}H NMR (600 MHz, $CDCl_3$, 298 K) δ 7.46 (dd, *J* = 8.3, 1.6 Hz, 2H), 7.38 (t, *J* = 8.0 Hz, 1H), 7.29 (d, *J* = 8.2 Hz, 2H), 7.16 – 7.03 (m, 2H), 3.75 (q, *J* = 7.2 Hz, 1H), 3.70 (s, 3H), 2.75 – 2.55 (m, 1H), 2.45 (ddd, *J* = 19.3, 8.9, 1.1 Hz, 1H), 2.14 – 2.02 (m, 1H), 1.99 – 1.91 (m, 1H), 1.89 – 1.74 (m, 5H), 1.75 – 1.55 (m, 4H), 1.53 (d, *J* = 7.2 Hz, 3H), 1.42 – 1.26 (m, 10H), 0.92 (s, 3H), 0.88 (s, 3H).

$^{13}C\{^1H\}$ NMR (151 MHz, $CDCl_3$, 298 K) δ 221.8, 174.8, 160.1 (d, *J* = 248.0 Hz), 147.4, 141.8 (d, *J* = 7.6 Hz), 133.4 (d, *J* = 1.3 Hz), 131.1 (d, *J* = 4.0 Hz), 129.2 (d, *J* = 2.9 Hz), 128.2 (d, *J* = 13.6 Hz), 127.3, 123.8 (d, *J* = 3.2 Hz), 115.6 (d, *J* = 23.7 Hz), 55.1, 52.6, 51.9, 48.2, 47.4, 45.3, 44.8, 39.2, 36.8, 36.3, 35.5, 32.0, 31.3, 30.1, 28.9, 22.2, 20.7, 18.8, 14.2, 12.8.

$^{19}F\{^1H\}$ NMR (282 MHz, $CDCl_3$) δ -117.58.

HRMS-ESIpos (m/z) calc'd for $C_{35}H_{44}O_3F$ $[M+H]^+$, 531.3269; found, 531.3269; deviation: 0.0 ppm.

Boc-azetidinyl chlorobenzene derivative S-5

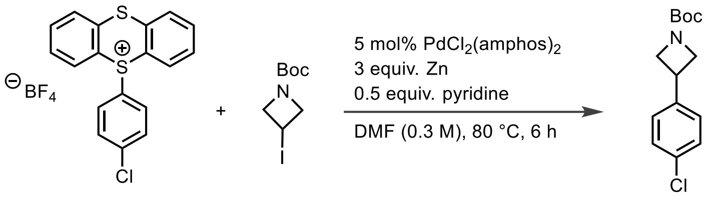

TT-13 **S-5**, 53%

To a 4-mL borosilicate vial containing a Teflon-coated magnetic stirring bar were chlorobenzene thianthrenium salt **TT-13** (124 mg, 0.300 mmol, 1.00 equiv.), $PdCl_2(amphos)_2$ (10.6 mg, 15.0 µmol, 5.00 mol%), and activated zinc dust (58.9 mg, 0.900 mmol, 3.00 equiv.). The vial was transferred into a nitrogen-filled glovebox. Dry DMF (1 mL, c = 0.3 M) was added to the solids. Subsequently, 1-boc-3-iodoazetidine (104 µL, 169 mg, 0.600 mmol, 2.00 equiv.) and pyridine (12 µL, 12 mg, 0.15 mmol, 0.50 equiv.) were added at 25 °C. The vial was sealed with a Teflon-lined screw cap, removed from the glovebox, and transferred to a heating block preheated at 80 °C where the reaction mixture was stirred rigorously (850 rpm) for 6 h. A color change from yellow to dark brown was observed. The mixture was cooled to 25 °C, subsequently diluted with ethyl acetate (40 mL), and thereafter poured into a separatory funnel. The organic layer was washed with water (1 × 40 mL). The aqueous layer was then extracted with ethyl acetate (3 × 40 mL). The organic layers were combined, dried over $MgSO_4$, filtered, and concentrated under reduced pressure. The resulting residue was purified by column chromatography on silica gel eluting with a solvent mixture of hexanes / ethyl acetate, (4:1 gradient to 3:2 (v/v)) to afford 42.6 mg (53%) of desired product **S-5** as a pale yellow oil.

R_f = 0.66 (hexanes/EtOAc, 3:2 (v:v))

NMR Spectroscopy:

^1H NMR (500 MHz, $CDCl_3$, 298 K) δ 7.32 (d, *J* = 8.5 Hz, 2H), 7.24 (d, *J* = 8.4 Hz, 2H), 4.32 (t, *J* = 8.7 Hz, 2H), 3.92 (dd, *J* = 8.7, 5.9 Hz, 2H), 3.74 – 3.65 (m, 1H), 1.46 (s, 9H).

$^{13}C\{^1H\}$ NMR (126 MHz, $CDCl_3$) δ 156.5, 140.9, 132.9, 129.0, 128.3, 79.8, 33.1, 28.6.

HRMS-ESIpos (m/z) calc'd for $C_{14}H_{18}NO_2ClNa$ $[M+Na]^+$, 290.0918; found, 290.0919; deviation: –0.3 ppm.

Alkylation of aryl thianthrenium salts with alkyl triflate

Methyl pyriproxyfen derivative S-6

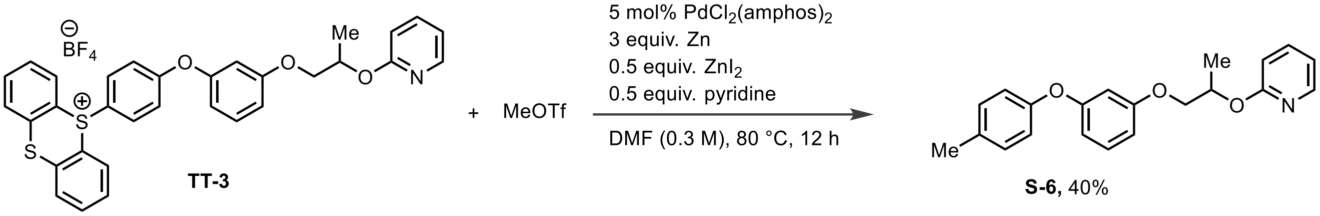

To a 4-mL borosilicate vial containing a Teflon-coated magnetic stirring bar were added pyriproxyfen thianthrenium salt **TT-3** (187 mg, 0.300 mmol, 1.00 equiv.), $PdCl_2(amphos)_2$ (10.6 mg, 15.0 µmol, 5.00 mol%), activated zinc dust (58.9 mg, 0.900 mmol, 3.00 equiv.), and ZnI_2 (47.9 mg, 0.150 mmol, 0.500 equiv.). The vial was transferred into a nitrogen-filled glovebox. Dry DMF (1 mL, c = 0.3 M) was added to the solids. Subsequently, methyl triflate (68 µL, 99 mg, 0.60 mmol, 2.0 equiv.) and pyridine (12 µL, 12 mg, 0.15 mmol, 0.50 equiv.) were added at 25 °C. The vial was sealed with a Teflon-lined screw cap, removed from the glovebox, and transferred to a heating block preheated at 80 °C where the reaction mixture was stirred rigorously (850 rpm) for 12 h. A color change from yellow to dark brown was observed. The mixture was cooled to 25 °C, subsequently diluted with ethyl acetate (40 mL), and thereafter poured into a separatory funnel. The organic layer was washed with water (1 × 40 mL). The aqueous layer was then extracted with ethyl acetate (3 × 40 mL). The organic layers were combined, dried over $MgSO_4$, filtered, and concentrated under reduced pressure. The resulting residue was purified by column chromatography on silica gel eluting with a solvent mixture of hexane / ethyl acetate, (1:0 gradient to 9:1 (v/v)) to afford 40.1 mg (40%) of desired product **S-6** as a colorless oil.

R_f = 0.44 (hexanes/EtOAc, 9:1 (v:v))

NMR Spectroscopy:

^{1}H NMR (300 MHz, $CDCl_3$, 298 K) δ 8.15 (ddd, *J* = 5.0, 2.0, 0.8 Hz, 1H), 7.56 (ddd, *J* = 8.3, 7.1, 2.0 Hz, 1H), 7.09 (d, *J* = 7.8 Hz, 2H), 6.96 – 6.87 (m, 4H), 6.89 – 6.82 (m, 3H), 6.74 (d, *J* = 8.3 Hz, 1H), 5.58 (dtd, *J* = 11.5, 6.4, 5.0 Hz, 1H), 4.18 (dd, *J* = 9.9, 5.3 Hz, 1H), 4.06 (dd, *J* = 9.9, 4.8 Hz, 1H), 2.31 (s, 3H), 1.48 (d, *J* = 6.4 Hz, 3H).

^{13}C{^{1}H} NMR (151 MHz, $CDCl_3$, 298 K) δ 163.3, 156.2, 155.1, 151.0, 146.9, 138.8, 132.2, 130.2, 120.4, 118.0, 116.9, 115.9, 111.8, 71.2, 69.4, 20.8, 17.2.

HRMS-ESIpos (m/z) calc'd for $C_{21}H_{22}NO_3$ $[M+H]^+$, 336.1594; found, 336.1597; deviation: –0.7 ppm.

Alkylation of aryl thianthrenium salts with alkyl bromides

Fluoro butyl pyriproxyfen derivative S-7

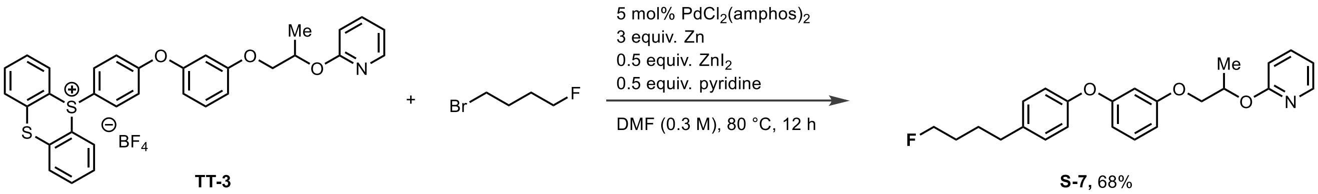

TT-3 **S-7,** 68%

To a 4-mL borosilicate vial containing a Teflon-coated magnetic stirring bar were added pyriproxyfen thianthrenium salt **TT-3** (187 mg, 0.300 mmol, 1.00 equiv.), $PdCl_2(amphos)_2$ (10.6 mg, 15.0 µmol, 5.00 mol%), activated zinc dust (58.9 mg, 0.900 mmol, 3.00 equiv.), and ZnI_2 (47.9 mg, 0.150 mmol, 0.500 equiv.). The vial was transferred into a nitrogen-filled glovebox. Dry DMF (1 mL, c = 0.3 M) was added to the solids. Subsequently, 1-bromo-4-fluorobutane (64 µL, 93 mg, 0.60 mmol, 2.0 equiv.) and pyridine (12 µL, 12 mg, 0.15 mmol, 0.50 equiv.) were added at 25 °C. The vial was sealed with a Teflon-lined screw cap, removed from the glovebox, and transferred to a heating block preheated at 80 °C where the reaction mixture was stirred rigorously (850 rpm) for 12 h. A color change from yellow to dark brown was observed. The mixture was cooled to 25 °C, subsequently diluted with ethyl acetate (40 mL), and thereafter poured into a separatory funnel. The organic layer was washed with water (1 × 40 mL). The aqueous layer was then extracted with ethyl acetate (3 × 40 mL). The organic layers were combined, dried over $MgSO_4$, filtered, and concentrated under reduced pressure. The resulting residue was purified by column chromatography on silica gel eluting with a solvent mixture of hexane / ethyl acetate, (1:0 gradient to 4:1 (v/v)) to afford 81.0 mg (68%) of desired product **S-7** as a colorless oil.

$\mathbf{R_f}$ = 0.27 (hexanes/EtOAc, 9:1 (v:v))

NMR Spectroscopy:

^{1}H NMR (300 MHz, $CDCl_3$, 298 K) δ 8.16 (ddd, *J* = 5.0, 2.0, 0.8 Hz, 1H), 7.57 (ddd, *J* = 8.4, 7.1, 2.0 Hz, 1H), 7.11 (d, *J* = 8.5 Hz, 2H), 7.04 – 6.81 (m, 7H), 6.75 (dt, *J* = 8.3, 0.9 Hz, 1H), 5.60 (dtd, *J* = 11.4, 6.4, 5.1 Hz, 1H), 4.54 (t, *J* = 5.6 Hz, 1H), 4.38 (t, *J* = 5.7 Hz, 1H), 4.19 (dd, *J* = 9.9, 5.3 Hz, 1H), 4.07 (dd, *J* = 9.9, 4.9 Hz, 1H), 2.63 (t, *J* = 7.1 Hz, 2H), 1.77 – 1.75 (m, 4H), 1.49 (d, *J* = 6.4 Hz, 3H).

^{13}C{^{1}H} NMR (75 MHz, $CDCl_3$, 298 K) δ 163.3, 156.6, 155.2, 150.8, 146.9, 138.8, 136.3, 129.6, 120.6, 117.9, 116.9, 115.9, 111.8, 84.1 (d, *J* = 164.6 Hz), 71.2, 69.5, 34.7, 30.1 (d, *J* = 19.6 Hz), 27.3 (d, *J* = 5.1 Hz), 17.14.

^{19}F{^{1}H} NMR (282 MHz, $CDCl_3$, 298 K) δ -218.24.

HRMS-ESIpos (m/z) calc'd for $C_{24}H_{27}NO_3F$ $[M+H]^+$, 396.1969; found, 396.1973; deviation: –0.8 ppm.

Oxetanyl pyriproxyfen derivative 10

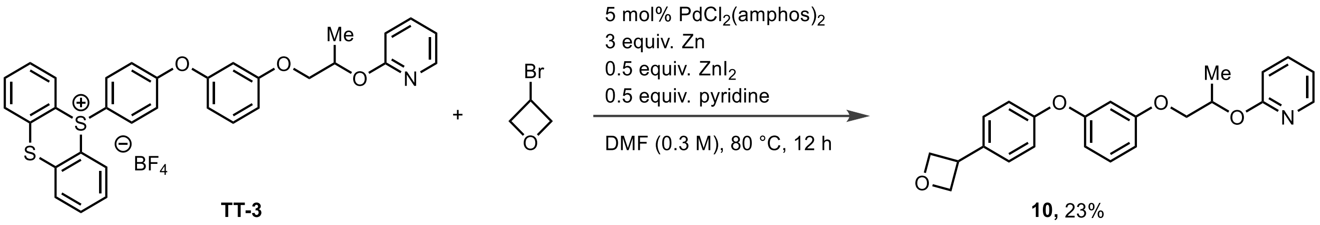

To a 4-mL borosilicate vial containing a Teflon-coated magnetic stirring bar were added pyriproxyfen thianthrenium salt **TT-3** (187 mg, 0.300 mmol, 1.00 equiv.), $PdCl_2(amphos)_2$ (10.6 mg, 15.0 µmol, 5.00 mol%), activated zinc dust (58.9 mg, 0.900 mmol, 3.00 equiv.), and ZnI_2 (47.9 mg, 0.150 mmol, 0.500 equiv.). The vial was transferred into a nitrogen-filled glovebox. Dry DMF (1 mL, c = 0.3 M) was added to the solids. Subsequently, 3-bromooxetane (50 µL, 80 mg, 0.6 mmol, 2 equiv.) and pyridine (12 µL, 12 mg, 0.15 mmol, 0.50 equiv.) were added at 25 °C. The vial was sealed with a Teflon-lined screw cap, removed from the glovebox, and transferred to a heating block preheated at 80 °C where the reaction mixture was stirred rigorously (850 rpm) for 12 h. A color change from yellow to dark brown was observed. The mixture was cooled to 25 °C, subsequently diluted with ethyl acetate (40 mL), and thereafter poured into a separatory funnel. The organic layer was washed with water (1 × 40 mL). The aqueous layer was then extracted with ethyl acetate (3 × 40 mL). The organic layers were combined, dried over $MgSO_4$, filtered, and concentrated under reduced pressure. The resulting residue was purified by column chromatography on silica gel eluting with a solvent mixture of hexane / ethyl acetate, (4:1 gradient to 3:2 (v/v)) to afford 26.0 mg (23%) of desired product **10** as a colorless oil.

*Data matches data obtained for product **10** from the reaction of **TT-3** with 3-iodooxetane (see page 80-81).*

Mechanistic investigation

Radical clock experiment

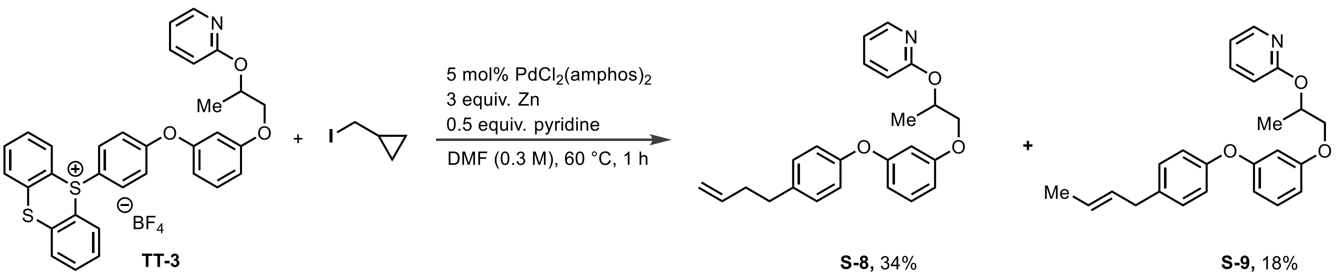

To a 4-mL borosilicate vial containing a Teflon-coated magnetic stirring bar were added pyriproxyfen thianthrenium salt **TT-3** (187 mg, 0.300 mmol, 1.00 equiv.), $PdCl_2(amphos)_2$ (10.6 mg, 15.0 µmol, 5.00 mol%.), and activated zinc dust (58.9 mg, 0.900 mmol, 3.00 equiv.). The vial was transferred into a nitrogen-filled glovebox. Dry DMF (1 mL, c = 0.3 M) was added to the solids. Subsequently, (iodomethyl)cyclopropane (68 µL, 110 mg, 0.60 mmol, 2.0 equiv.) and pyridine (12 µL, 12 mg, 0.15 mmol, 0.50 equiv.) were added at 25 °C. The vial was sealed with a Teflon-lined screw cap, removed from the glovebox, and transferred to a

heating block preheated at 60 °C where the reaction mixture was stirred rigorously (850 rpm) for 1 h. A color change from yellow to dark brown was observed. The mixture was cooled to 25 °C, subsequently diluted with ethyl acetate (40 mL), and thereafter poured into a separatory funnel. The organic layer was washed with water (1 × 40 mL). The aqueous layer was then extracted with ethyl acetate (3 × 40 mL). The organic layers were combined, dried over $MgSO_4$, filtered, and concentrated under reduced pressure. The resulting residue was purified by column chromatography on silica gel eluting with a solvent mixture of hexane / ethyl acetate, (1:0 gradient to 4:1 (v/v)) to afford 58.4 mg (52 %) of a mixture of **S-8** and **S-9** as a colorless oil. From NMR analysis the ratio of the two components in the product mixture were found to be 6.5 : 3.5 (**S-8** : **S-9**). From this ratio the yields of the respective products were determined to be 34% (**S-8**) and 18% (**S-9**).

R_f = 0.63 (hexanes/EtOAc, 4:1 (v:v))

NMR Spectroscopy (S-8):

^{1}H NMR (500 MHz, $CDCl_3$, 298 K) δ 8.18 (dd, *J* = 5.3, 2.0 Hz, 1H), 7.64 (ddd, *J* = 8.8, 7.0, 2.0 Hz, 1H), 7.18 – 7.05 (m, 2H), 7.02 – 6.84 (m, 7H), 6.81 (d, *J* = 8.4 Hz, 1H), 5.85 (ddt, *J* = 16.9, 10.2, 6.6 Hz, 1H), 5.63 – 5.43 (m, 1H), 5.15 – 4.84 (m, 2H), 4.19 (ddd, *J* = 10.0, 5.6, 1.1 Hz, 1H), 4.08 (ddd, *J* = 9.9, 4.6, 1.1 Hz, 1H), 2.67 (dd, *J* = 8.2, 6.8 Hz, 2H), 2.44 – 2.25 (m, 2H), 1.49 (d, *J* = 6.4 Hz, 3H).

^{13}C{^{1}H} NMR (75 MHz, $CDCl_3$, 298 K) δ 163.2, 156.6, 155.1, 150.9, 146.6, 139.2, 138.2, 136.2, 129.6, 120.9, 117.8, 117.0, 115.9, 115.1, 111.9, 71.3, 69.9, 35.8, 34.7, 17.1.

NMR Spectroscopy (S-9):

^{1}H NMR (500 MHz, $CDCl_3$, 298 K) δ 8.18 (dd, *J* = 5.3, 2.0 Hz, 1H), 7.64 (ddd, *J* = 8.8, 7.0, 2.0 Hz, 1H), 7.18 – 7.05 (m, 2H), 7.02 – 6.84 (m, 7H), 6.81 (d, *J* = 8.4 Hz, 1H), 5.63 – 5.43 (m, 3H), 4.19 (ddd, *J* = 10.0, 5.6, 1.1 Hz, 1H), 4.08 (ddd, *J* = 9.9, 4.6, 1.1 Hz, 1H), 3.27 (d, *J* = 6.3 Hz, 2H), 1.69 (dd, *J* = 6.2, 1.4 Hz, 3H), 1.49 (d, *J* = 6.4 Hz, 3H).

^{13}C{^{1}H} NMR (126 MHz, $CDCl_3$, 298 K) δ 163.2, 156.6, 155.3, 150.9, 146.6, 139.2, 135.4, 130.3, 129.7, 126.4, 120.6, 117.9, 117.0, 115.9, 111.9, 71.3, 69.9, 38.4, 18.0, 17.1.

HRMS-ESIpos (m/z) calc'd for $C_{24}H_{26}NO_3$ $[M+H]^+$, 376.1907; found, 376.1906; deviation: 0.4 ppm.

Boc-piperidinyl diphenyl ether derivative 3 in the absence of TEMPO

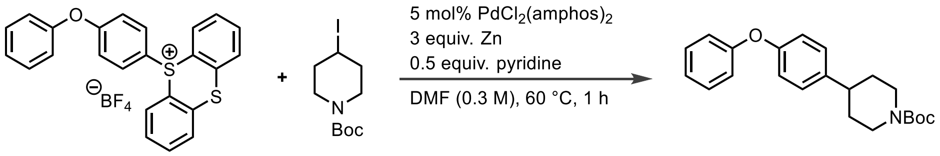

TT-1 **3,** 62%

To a 4-mL borosilicate vial containing a Teflon-coated magnetic stirring bar were added diphenyl ether thianthrenium salt **TT-1** (141 mg, 0.300 mmol, 1.00 equiv.), $PdCl_2(amphos)_2$ (10.6 mg, 15.0 µmol, 5.00 mol%), and activated zinc dust (58.9 mg, 0.900 mmol, 3.00 equiv.). The vial was transferred into a nitrogen-filled glovebox. Dry DMF (1 mL, c = 0.3 M) was added to the solids. Subsequently, 1-boc-4-iodopiperidine

(186 mg, 0.600 mmol, 2.00 equiv.) and pyridine (12 µL, 12 mg, 0.15 mmol, 0.50 equiv.) were added at 25 °C. The vial was sealed with a Teflon-lined screw cap, removed from the glovebox, and transferred to a heating block preheated at 60 °C where the reaction mixture was stirred rigorously (850 rpm) for 1 h. A color change from yellow to dark brown was observed. The mixture was cooled to 25 °C, subsequently diluted with ethyl acetate (40 mL), and thereafter poured into a separatory funnel. The organic layer was washed with water (1 × 40 mL). The aqueous layer was then extracted with ethyl acetate (3 × 40 mL). The organic layers were combined, dried over $MgSO_4$, filtered, and concentrated under reduced pressure. The resulting residue was purified by column chromatography on silica gel eluting with a solvent mixture of hexanes / ethyl acetate, (1:0 gradient to 7:3 (v/v)) to afford 65.3 mg (62%) of desired product **3** as a white solid.

$\mathbf{R_f}$ = 0.78 (hexanes/EtOAc, 1:0 (v:v))

NMR Spectroscopy:

^{1}H NMR (500 MHz, $CDCl_3$, 298 K) δ 7.36 – 7.28 (m, 2H), 7.19 – 7.12 (m, 2H), 7.09 (tt, *J* = 7.4, 1.1 Hz, 1H), 7.00 (dd, *J* = 8.7, 1.1 Hz, 2H), 6.98 – 6.91 (m, 2H), 4.24 (d, *J* = 13.3 Hz, 2H), 2.80 (td, *J* = 13.0, 2.6 Hz, 2H), 2.63 (tt, *J* = 12.2, 3.6 Hz, 1H), 1.82 (ddd, *J* = 12.4, 4.3, 2.2 Hz, 2H), 1.66 – 1.54 (m, 2H), 1.48 (s, 9H).

^{13}C{^{1}H} NMR (126 MHz, $CDCl_3$, 298 K) δ 157.8, 156.0, 155.3, 141.1, 130.1, 128.3, 123.5, 119.3, 119.1, 79.8, 44.8, 42.4, 33.8, 28.9.

HRMS-ESIpos (m/z) calc'd for $C_{22}H_{27}NO_3Na$ $[M+Na]^+$, 376.1883; found, 376.1881; deviation: 0.5 ppm.

Boc-piperidinyl diphenyl ether derivative 3 in the presence of TEMPO

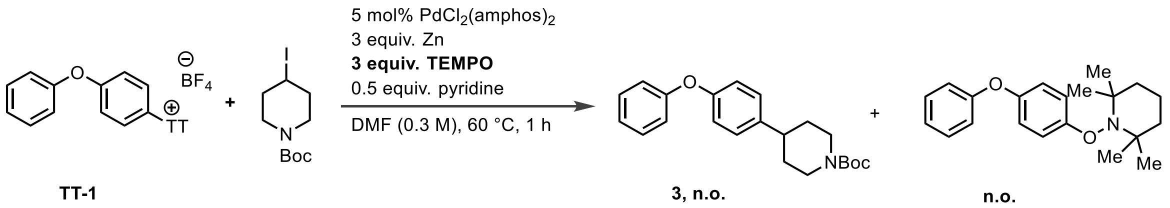

To a 4-mL borosilicate vial containing a Teflon-coated magnetic stirring bar were added diphenyl ether thianthrenium salt **TT-1** (141 mg, 0.0300 mmol, 1.00 equiv.), $PdCl_2(amphos)_2$ (10.6 mg, 15.0 µmol, 5.00 mol%), activated Zn dust (58.9 mg, 0.900 mmol, 3.00 equiv.) and (2,2,6,6-tetramethylpiperidin-1-yl)oxyl (TEMPO) (141 mg, 0.9 mmol, 3 equiv.) The vial was transferred into a nitrogen-filled glovebox. Dry DMF (1 mL, c = 0.3 M) was added to the solids. Subsequently, 1-boc-4-iodopiperidine (186 mg, 0.600 mmol, 2.00 equiv.) and pyridine (12 µL, 12 mg, 0.15 mmol, 0.50 equiv.) were added at 25 °C. The vial was sealed with a Teflon-lined screw cap, removed from the glovebox, and transferred to a heating block heated at 60 °C where the reaction mixture was stirred rigorously (850 rpm) for 12 h. The mixture was cooled to 25 °C, subsequently diluted with ethyl acetate (40 mL), and thereafter poured into a separatory funnel. The organic layer was washed with water (1 × 40 mL). The aqueous layer was then extracted with ethyl acetate (3 × 40 mL). The organic layers were combined, dried over $MgSO_4$, filtered, and concentrated under reduced

pressure. The resulting reaction mixture was analyzed by ^{1}H NMR spectroscopy and LCMS. ^{1}H NMR and LCMS showed no product formation.

Radical clock starting material allyl ether thianthrenium salt TT-2

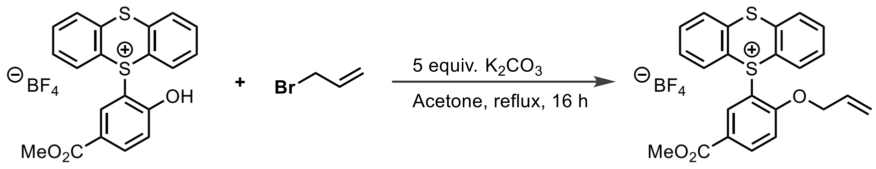

TT-2' **TT-2,** 64%

Under ambient atmosphere, a 10 mL round bottom flask was charged with TT-2' (395 mg, 0.870 mmol, 1.00 equiv.), and K_2CO_3 (601 mg, 4.35 mmol, 5.00 equiv.). Acetone was added (6.0 mL, c = 0.15 M) at 25 °C. Allyl bromide (0.38 mL, 530 mg, 4.3 mmol, 5.0 equiv.) was subsequently added into the reaction mixture at 25 °C. The reaction mixture was stirred at reflux for 16 h until a white precipitate was formed. The reaction mixture was concentrated under reduced pressure, and the residue was dissolved in 20 mL dichloromethane. The resulting mixture was poured into a separatory funnel, which was pre-charged with 20 mL water. The dichloromethane layer was collected, and the aqueous layer was further extracted with dichloromethane (2 × 20 mL). The combined dichloromethane solution was washed with aqueous $NaBF_4$ solution (2 × 20 mL, 5% w/w). The organic layer was dried over $MgSO_4$, filtered, and the solvent was removed under reduced pressure. The residue was purified by chromatography on silica gel eluting with dichloromethane / *i*-PrOH (1:0 to 9:1). The product was collected and dried in vacuo to afford **TT-2** (276 mg, 64%) as a white solid.

$\mathbf{R_f}$ = 0.61 (dichloromethane/*i*-PrOH, 9:1 (v:v))

NMR Spectroscopy:

^{1}H NMR (500 MHz, CD_2Cl_2, 298 K) δ 8.31 (dd, *J* = 8.0, 1.3 Hz, 2H), 8.20 (dd, *J* = 8.8, 2.0 Hz, 1H), 7.92 (dd, *J* = 8.0, 1.4 Hz, 2H), 7.84 (td, *J* = 7.7, 1.4 Hz, 2H), 7.80 – 7.70 (m, 2H), 7.24 (d, *J* = 1.9 Hz, 1H), 7.16 (d, *J* = 8.8 Hz, 1H), 6.06 (ddt, *J* = 17.2, 10.4, 5.9 Hz, 1H), 5.44 (dq, *J* = 10.4, 1.1 Hz, 1H), 5.35 (dq, *J* = 17.2, 1.4 Hz, 1H), 4.79 (d, *J* = 5.9 Hz, 2H), 3.77 (s, 3H).

^{13}C{^{1}H} NMR (126 MHz, CD_2Cl_2, 298 K) δ 164.7, 160.6, 138.1, 137.4, 135.6, 135.5, 131.0, 130.9, 130.5, 124.4, 121.5, 116.9, 114.9, 109.4, 72.1, 52.9.

^{19}F{^{1}H} NMR (282 MHz, CD_2Cl_2, 298 K) δ -150.25, -150.31.

HRMS-ESIpos (m/z) calc'd for $C_{23}H_{19}O_3S_2^+$[M–BF_4]$^+$, 407.0770; found, 407.0774; deviation: –1.0 ppm

Radical clock cyclization experiment

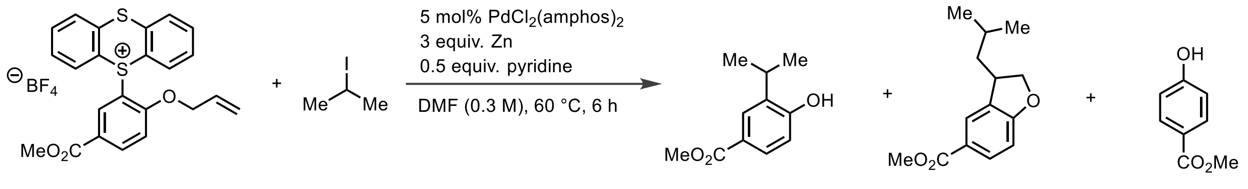

TT-2 **22,** 5% **not detected** **23,** 18%

To a 4-mL borosilicate vial containing a Teflon-coated magnetic stirring bar were allyl ether thianthrenium salt **TT-2** (148 mg, 0.300 mmol, 1.00 equiv.), $PdCl_2(amphos)_2$ (10.6 mg, 15.0 µmol, 5.00 mol%), and activated zinc dust (58.9 mg, 0.900 mmol, 3.00 equiv.). The vial was transferred into a nitrogen-filled glovebox. Dry DMF (1 mL, c = 0.3 M) was added to the solids. Subsequently, 2-iodopropane (60 µL, 100 mg, 0.6 mmol, 2 equiv.) and pyridine (12 µL, 12 mg, 0.15 mmol, 0.50 equiv.) were added at 25 °C. The vial was sealed with a Teflon-lined screw cap, removed from the glovebox, and transferred to a heating block preheated at 80 °C where the reaction mixture was stirred rigorously (850 rpm) for 6 h. A color change from yellow to dark brown was observed. The mixture was cooled to 25 °C, subsequently diluted with ethyl acetate (40 mL), and thereafter poured into a separatory funnel. The organic layer was washed with water (1 × 40 mL). The aqueous layer was then extracted with ethyl acetate (3 × 40 mL). The organic layers were combined, dried over $MgSO_4$, filtered, and concentrated under reduced pressure. The resulting residue was purified by column chromatography on silica gel eluting with a solvent mixture of hexanes / ethyl acetate, (4:1 gradient to 3:2 (v/v)) to afford 4.3 mg (7%) of product **22** as a 1 : 0.37 ratio branched : linear product as a colorless oil and 8.3 mg (18%) of by-product **23**. From NMR ratios, 5% yield of the desired branched product **22** was determined to have be obtained. No cyclized product was observed.

Compound 22 R_f = 0.39 (hexanes/EtOAc, 4:1 (v:v))

Compound 23 R_f = 0.32 (hexanes/EtOAc, 4:1 (v:v))

NMR Spectroscopy for compound 22:

^{1}H NMR (500 MHz, $CDCl_3$, 298 K) δ 7.92 (d, *J* = 2.2 Hz, 1H), 7.79 (dd, *J* = 8.4, 2.1 Hz, 1H), 6.76 (d, *J* = 8.3 Hz, 1H), 3.88 (s, 3H), 3.21 (hept, *J* = 6.9 Hz, 1H), 1.28 (d, *J* = 6.9 Hz, 6H).

^{13}C{^{1}H} NMR (151 MHz, $CDCl_3$) δ 167.3, 157.1, 134.5, 129.1, 128.7, 123.1, 115.2, 52.0, 27.2, 22.5.

HRMS-ESIpos (m/z) calc'd for $C_{11}H_{14}O_3Na$ $[M+Na]^+$, 217.0835; found, 217.0833; deviation: 0.9 ppm.

NMR Spectroscopy for compound 23:

^{1}H NMR (500 MHz, $CDCl_3$, 298 K) δ δ 7.96 (d, *J* = 8.9 Hz, 2H), 6.86 (d, *J* = 8.9 Hz, 2H), 3.89 (s, 3H).

^{13}C{^{1}H} NMR (151 MHz, $CDCl_3$) δ 167.0, 159.8, 132.1, 123.0, 115.3, 52.1.

HRMS-ESIpos (m/z) calc'd for $C_8H_8O_3Na$ $[M+Na]^+$, 175.0366; found, 175.0364; deviation: 0.7 ppm.

Control experiments to probe for the reduction by zinc

To a 4-mL borosilicate vial containing a Teflon-coated magnetic stirring bar were added reagent (0.1 mmol, 1.0 equiv.), and/or $PdCl_2(amphos)_2$ (3.5 mg, 5.0 µmol, 5.0 mol%), and/or activated zinc powder 100-mesh 99.5% (20 mg, 0.30 mmol, 3.0 equiv.). The vial was transferred into a nitrogen-filled glovebox. Dry solvent (1 mL, c = 0.1 M) was added to the solids followed by mesitylene (14 µL, 12 mg, 0.1 mmol) as an internal standard at 25 °C. The vial was sealed with a Teflon-lined screw cap, removed from the glovebox, and transferred to a heating block preheated at 60 °C where the reaction mixture was stirred rigorously (850 rpm) for 1 h. Experiments A-D were analyzed by ^{1}H-NMR spectroscopy. The ^{1}H NMR resonances of the diphenyl ether protons of the product between 6.8 and 7.4 ppm was integrated relative to the ^{1}H NMR resonances of the aromatic protons of mesitylene (δ = 6.70 ppm). Experiment E was analyzed by ^{2}D NMR spectroscopy. The reactions were carried out three times and an average of the yields were taken.

A)

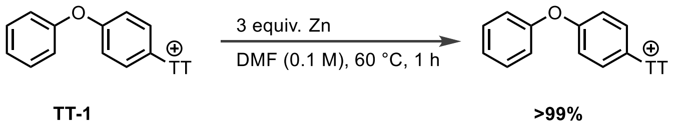

B)

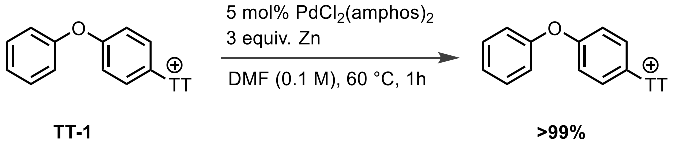

C)

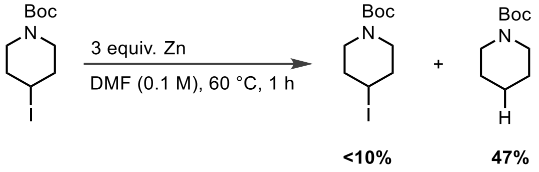

D)

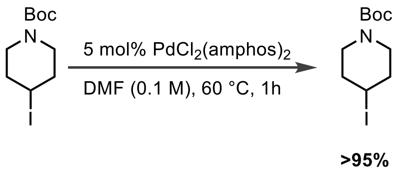

E)

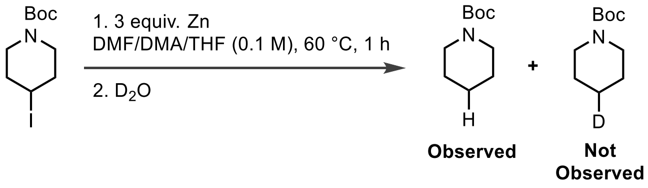

Palladium-catalyzed aryl C–H alkylation via bromination versus thianthrenation

General procedures for the bromination of arenes using 3 different methods

Bromination of arenes with condition A

In a 4-mL borosilicate vial containing a Teflon-coated magnetic stirring bar, arene (1.00 equiv.) was dissolved or suspended in AcOH (c = 0.30 M) at 25 °C under ambient atmosphere. Subsequently, a suspension of bromine and iron(III)-chloride in acetic acid (c_{Br2} = 0.50 M, c_{FeCl3} = 0.50 M, 1.0 equiv. Br_2, 1.0 equiv. $FeCl_3$) was added, and the mixture was stirred at 25 °C for 24 h. The reaction mixture was poured onto a mixture of water and ethyl acetate. The mixture was poured into a separatory funnel, and the layers were separated. The aqueous layer was extracted with ethyl acetate. The combined organic layers were dried over $MgSO_4$. The solvent was removed under reduced pressure, and the residue was dried in vacuo. The obtained residue was analyzed by ^1H NMR spectroscopy and LCMS. The residue was then was purified by column chromatography on silica gel eluting with a solvent mixture of hexanes / ethyl acetate. The results are summarized in table S7.

Bromination of arenes with condition B[9]

To a 4-mL borosilicate vial containing a Teflon-coated magnetic stirring bar was added arene (1.00 equiv.) and *N*-bromosuccinimide (1.00 equiv.). The vial was sealed with a septum-cap, and evacuated and filled with argon three times. In a nitrogen-filled glovebox, a solution of gold trichloride (1.0 mol%) in 1,2-dichloroethane (1 mL) was prepared. The vial was sealed with a septum-cap, removed from the glovebox, and the gold trichloride solution was added via the septum using a syringe to the vial containing the stirring bar, arene, and *N*-bromosuccinimide. The reaction mixture was stirred at 80 °C for 24 h. At this point, the reaction mixture was analyzed by ^1H NMR spectroscopy and LCMS. The results are summarized in table S7.

Bromination of arenes with condition C[10]

In a 4-mL borosilicate vial containing a Teflon-coated magnetic stirring bar, arene (1.00 equiv.) was dissolved in HFIP (c = 0.3 M). Then *N*-bromosuccinimide (1.00 equiv) was added to the reaction mixture and stirred at 25 °C for 24 h. At this point, the reaction mixture was analyzed by ^1H NMR spectroscopy and LCMS. The results are summarized in table S7.

Table S7: Position of thianthrenation and bromination of bifonazole and indomethacin methyl ester

	**Bromination** *condition A*	**Bromination** *condition B*	**Bromination** *condition C*	**Thianthrenation**
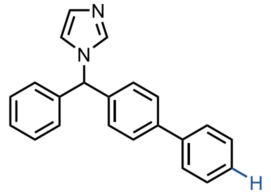	Complex mixture[a] (see Figure S6, S7, S10)	Complex mixture[a] (see Figure S6, S8)	Complex mixture[a] (see Figure S6, S9)	Single isomer (see Figure S11)

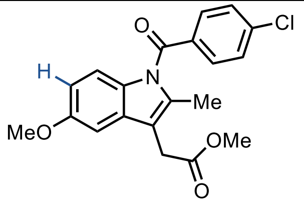	Complex mixture[a] (see Figure S12, S13, S16)	Complex mixture[a] (see Figure S12, S14)	Complex mixture[a] (see Figure S12, S15)	Single isomer (see Figure S17)

[a]Due to the complex make up of these reaction mixtures, bromination selectivity could not be quantified. The blue hydrogen denotes the position of thianthrenation.

Procedures for the bromination of bifonazole using 3 different methods

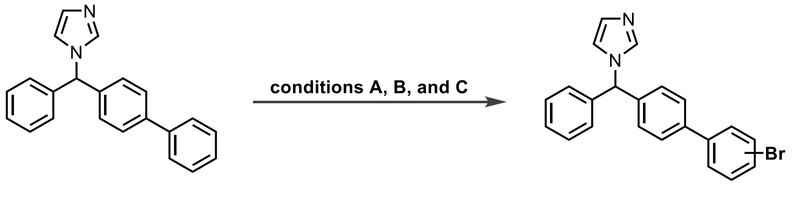

S-10

Bromination of bifonazole with condition A

In a 4-mL borosilicate vial containing a Teflon-coated magnetic stirring bar, bifonazole (93.1 mg, 0.300 mmol, 1.00 equiv.) was dissolved or suspended in AcOH (1 mL, c = 0.3 M) at 25 °C under ambient atmosphere. Subsequently, a suspension of bromine and iron(III)-chloride in acetic acid (c_{Br2} = 0.50 M, c_{FeCl3} = 0.50 M, 1.0 equiv. Br_2, 1.0 equiv. $FeCl_3$) was added, and the mixture was stirred at 25 °C for 24 h. The reaction mixture was poured onto a mixture of water (ca. 30 mL) and ethyl acetate (ca. 10 mL). The mixture was poured into a separatory funnel, and the layers were separated. The aqueous layer was extracted with ethyl acetate (ca. 10 mL). The combined organic layers were dried over $MgSO_4$.The solvent was removed under reduced pressure, and the residue was dried in vacuo. The obtained material was analyzed by ^{1}H NMR spectroscopy (Figure S6) and LCMS (Figure S7). The resulting residue was purified by column chromatography on silica gel eluting with a solvent mixture of hexanes / ethyl acetate, (1:0 gradient to 0:1 (v/v)) to afford 44.2 mg (38%) of a complex mixture of **S-10** and other unknown by-products as a yellow oil. ^{1}H NMR (Figure S10) of the complex mixture of **S-10** after purification by column chromatography are shown below.

Bromination of bifonazole with condition B[9]

To a 4-mL borosilicate vial containing a Teflon-coated magnetic stirring bar was added bifonazole (93.1 mg, 0.300 mmol, 1.00 equiv.) and *N*-bromosuccinimide (53.4 mg, .300 mmol, 1.00 equiv.). The vial was sealed with a septum-cap, and evacuated and filled with argon three times. In a nitrogen-filled glovebox, a solution of gold trichloride (0.91 mg, 3.0 µmol, 1.0 mol%) in 1,2-dichloroethane (1 mL) was prepared. The vial was sealed with a septum-cap, removed from the glovebox, and the gold trichloride solution was added via the septum using a syringe to the vial containing the stirring bar, bifonazole, and *N*-bromosuccinimide. The reaction mixture was stirred at 80 °C for 24 h. At this point, the reaction mixture was analyzed by ^{1}H NMR spectroscopy (Figure S6) and LCMS (Figure S8). The results are summarized in table S7.

Bromination of bifonazole with condition C[10]

In a 4-mL borosilicate vial containing a Teflon-coated magnetic stirring bar, bifonazole (93.1 mg, 0.300 mmol, 1.00 equiv.) was dissolved in HFIP (1 mL, c = 0.3 M). Then *N*-bromosuccinimide (53.4 mg, 0.300 mmol, 1.00 equiv) was added to the reaction mixture and stirred at 25 °C for 24 h. At this point, the reaction mixture was analyzed by ^{1}H NMR spectroscopy (Figure S6) and LCMS (Figure S9). The results are summarized in table S7.

Determination of selectivity of bromination and subsequent alkylation: bifonazole

The reaction mixtures for the bromination of bifonazole were analyzed by ^{1}H NMR spectroscopy and LCMS. The crude ^{1}H NMR was too messy to glean any quantitative information from them. The LCMS traces showed complex mixtures of mono-bromination isomers and non-brominated isomers. Due to the complex make up of these reactions, bromination selectivity could not be quantified. Reaction mixture for the bromination of bifonazole using condition A gave the highest conversion of starting material, hence the reaction mixture was purified. The resulting residue was then used for the subsequent palladium-catalyzed alkylation. The alkylation reaction was analyzed by ^{1}H NMR spectroscopy and LCMS. No alkylated product formation was detected.

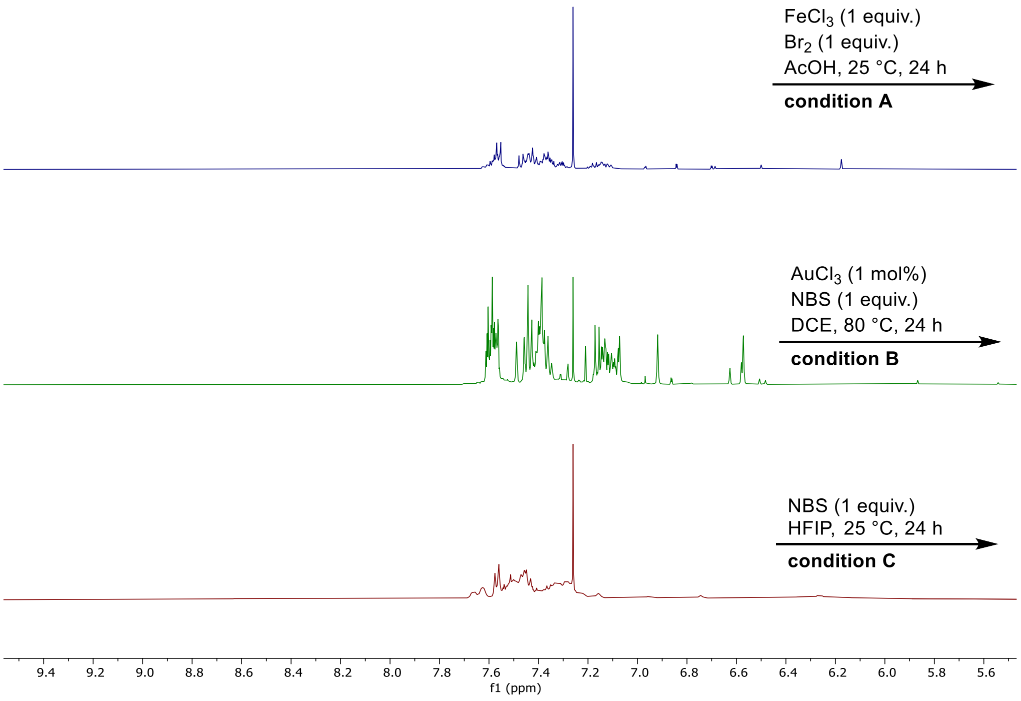

Figure S6: ^{1}H NMR analysis of the bromination of bifonazole using various conditions prior to purification (conditions A, B, and C), $CDCl_3$, 500 MHz, 298 K.

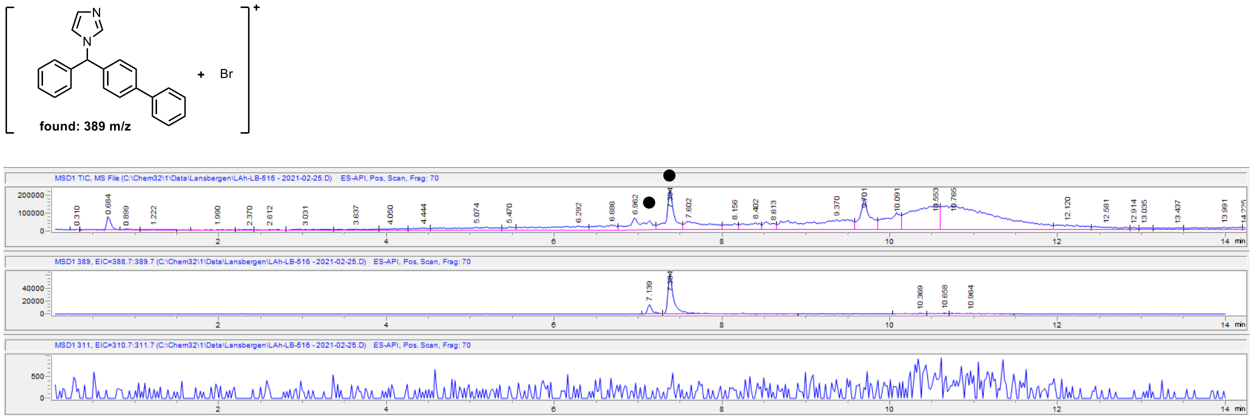

Figure S7: LCMS analysis of the bromination of bifonazole with condition A

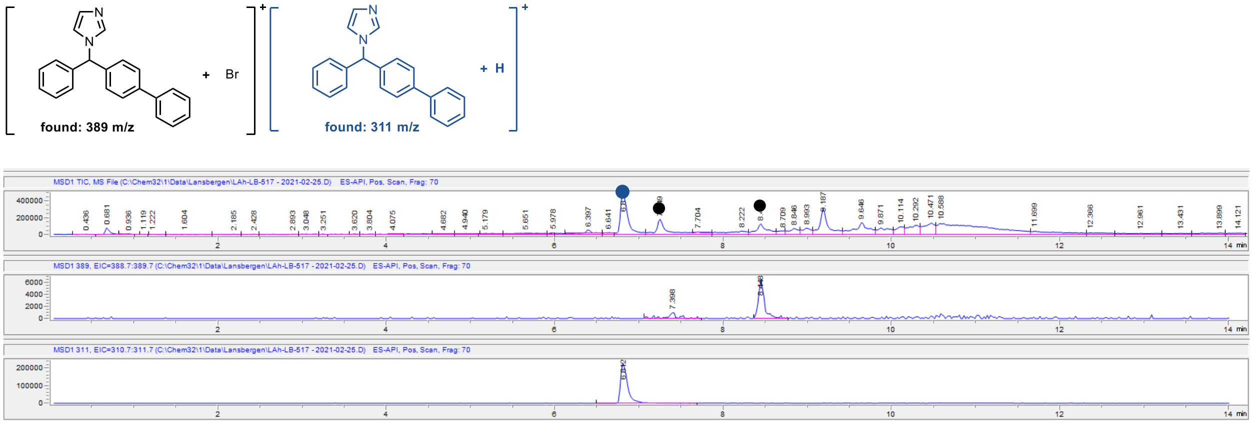

Figure S8: LCMS analysis of the bromination of bifonazole with condition B

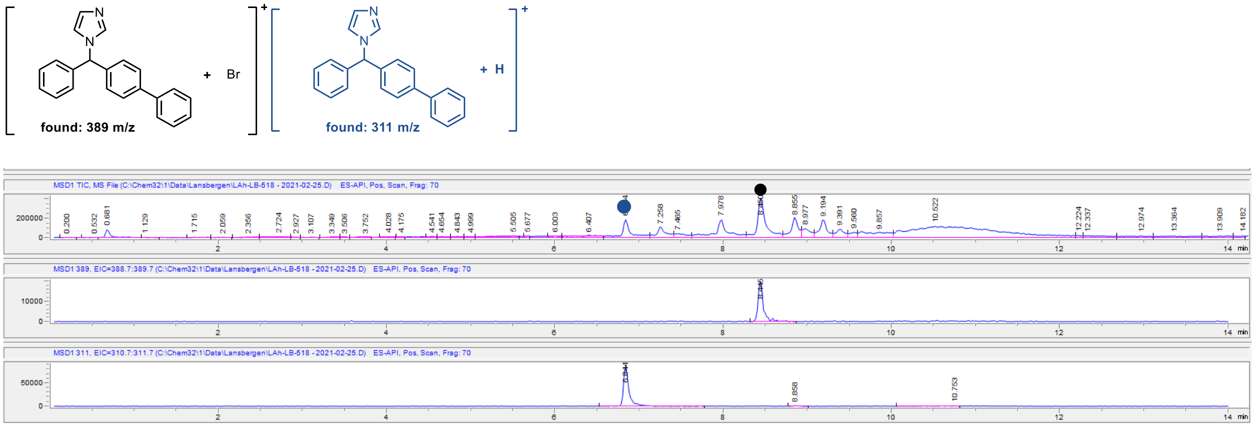

Figure S9: LCMS analysis of the bromination of bifonazole with condition C

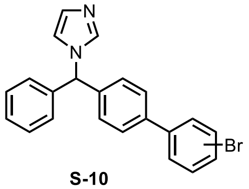

S-10

Under these reaction conditions, selective bromination of bifonazole could not be achieved

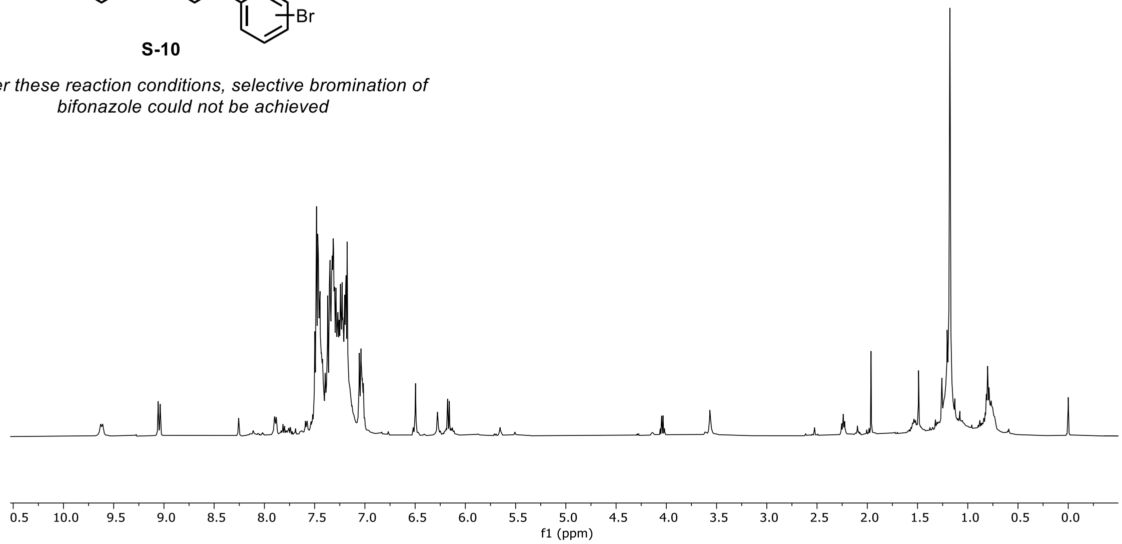

Figure S10: ^{1}H NMR of reaction mixture of compound **S-10** using condition A after purification by column chromatography, $CDCl_3$, 500 MHz, 298 K. ^{1}H NMR shows that bifonazole cannot undergo selective bromination and a complex mixture must be used for the subsequent alkylation step.

^{1}H NMR analysis for the selective thianthrenation of bifonazole[2, 4]

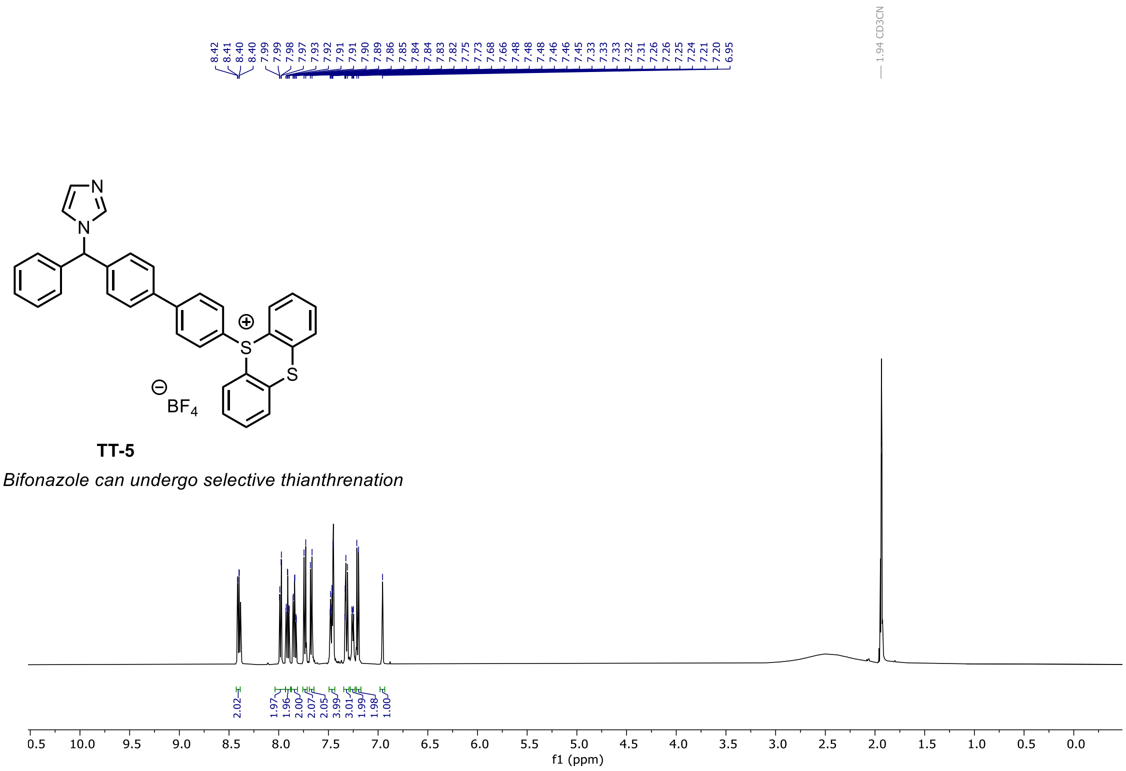

Figure S11: ^{1}H NMR of compound **TT-5** after purification by column chromatography, CD_3CN, 500 MHz, 298 K. ^{1}H NMR shows that bifonazole can undergo selective thianthrenation and a pure compound can be used for the subsequent alkylation step (see page S21, compound **1** for the selective alkylation of **TT-5**).

NMR Spectroscopy:

^{1}H NMR (500 MHz, CD_3CN, 298 K) δ 8.41 (dd, *J* = 7.9, 1.4 Hz, 2H), 7.98 (dd, *J* = 7.9, 1.4 Hz, 2H), 7.91 (td, *J* = 7.7, 1.4 Hz, 2H), 7.84 (td, *J* = 7.7, 1.4 Hz, 2H), 7.74 (d, *J* = 8.9 Hz, 2H), 7.67 (d, *J* = 8.5 Hz, 2H), 7.52 – 7.39 (m, 4H), 7.35 – 7.26 (m, 3H), 7.28 – 7.23 (m, 2H), 7.21 (d, *J* = 8.8 Hz, 2H), 6.95 (s, 1H).

Palladium-catalyzed alkylation of S-10

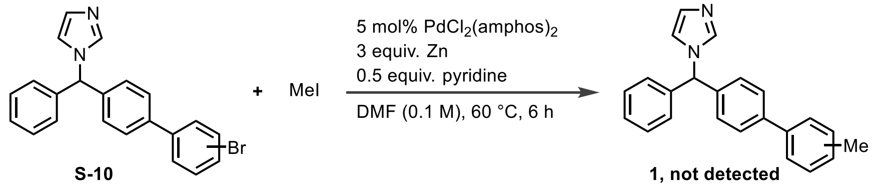

To a 4-mL borosilicate vial containing a Teflon-coated magnetic stirring bar were added bromo bifonazole reaction mixture **S-10** (38.9 mg, 0.100 mmol, 1.00 equiv.), $PdCl_2(amphos)_2$ (3.5 mg, 5.0 µmol, 5.0 mol%) and activated zinc dust (19.6 mg, 0.300 mmol, 3.00 equiv.). The vial was transferred into a nitrogen-filled glovebox. Dry DMF (1 mL, c = 0.1 M) was added to the solids. Subsequently, methyl iodide (13 µL, 28 mg, 0.20 mmol, 2.0 equiv.) and pyridine (4 µL, 4 mg, 5 µmol, 0.5 equiv.) were added at 25 °C. The vial was

sealed with a Teflon-lined screw cap, removed from the glovebox, and transferred to a heating block preheated at 60 °C where the reaction mixture was stirred rigorously (850 rpm) for 6 h. A color change from yellow to dark brown was observed. The mixture was cooled to 25 °C, subsequently diluted with ethyl acetate (10 mL), and thereafter poured into a separatory funnel. The organic layers were combined, dried over $MgSO_4$, filtered, and concentrated under reduced pressure. The resulting residue was purified by column chromatography on silica gel eluting with a solvent mixture of hexanes / ethyl acetate, (1:0 gradient to 0:1 (v/v)). The resulting fractions collected were analyzed by LCMS, GCMS, ^{1}H NMR spectroscopy, and ^{13}C NMR spectroscopy. No formation of product **1** could be accurately confirmed.

Summary of results for the methylation of bifonazole via bromination vs. thianthrenation

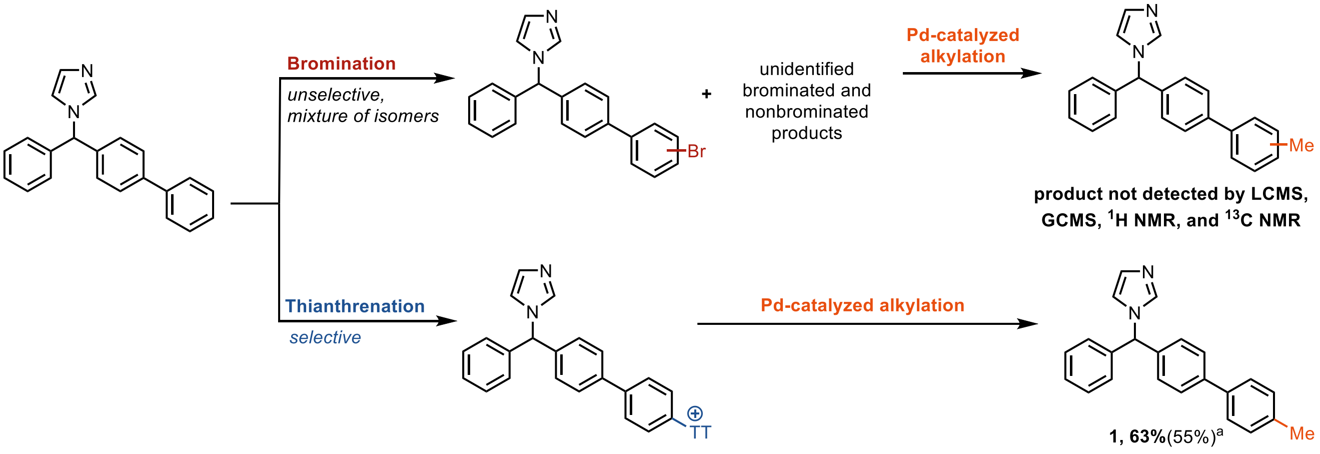

[a] Two-step yield.

Procedures for the bromination of indomethacin methyl ester using 3 different methods

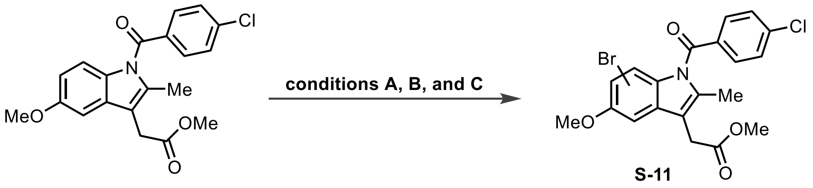

Bromination of indomethacin methyl ester with condition A

In a 25 mL round-bottom flask containing a Teflon-coated magnetic stirring bar, indomethacin methyl ester (357 mg, 1.00 mmol, 1.00 equiv.) was dissolved or suspended in AcOH (3 mL, c = 0.3 M) at 25 °C under ambient atmosphere. Subsequently, a suspension of bromine and iron(III)-chloride in acetic acid (c_{Br2} = 0.50 M, c_{FeCl3} = 0.50 M, 1.0 equiv. Br_2, 1.0 equiv. $FeCl_3$) was added, and the mixture was stirred at 25 °C for 24 h. The reaction mixture was poured onto a mixture of water (ca. 60 mL) and ethyl acetate (ca. 20 mL). The mixture was poured into a separatory funnel, and the layers were separated. The aqueous layer was extracted with ethyl acetate (ca. 10 mL). The combined organic layers were dried over $MgSO_4$.The solvent was removed under reduced pressure, and the residue was dried in vacuo. The obtained material was analyzed by ^{1}H NMR spectroscopy (Figure S12) and LCMS (Figure S13). The resulting residue was purified

by column chromatography on silica gel eluting with a solvent mixture of hexanes / ethyl acetate, (7:3 gradient to 2:3 (v/v)) to afford 240 mg (45%) of **S-11** as a mixture of mono-, di-, and tri-brominated products as a yellow oil. ^{1}H NMR (Figure S16) of **S-11** after purification by column chromatography are shown below.

Bromination of indomethacin methyl ester with condition B[9]

To a 4-mL borosilicate vial containing a Teflon-coated magnetic stirring bar was added indomethacin methyl ester (112 mg, 0.300 mmol, 1.00 equiv.) and *N*-bromosuccinimide (53.4 mg, 0.300 mmol, 1.00 equiv.). The vial was sealed with a septum-cap, and evacuated and filled with argon three times. In a nitrogen-filled glove box, a solution of gold trichloride (0.91 mg, 3.0 µmol, 1.0 mol%) in 1,2-dichloroethane (1 mL) was prepared. The vial was sealed with a septum-cap, removed from the glovebox, and the gold trichloride solution was added via the septum using a syringe to the vial containing the stirring bar, indomethacin methyl ester, and *N*-bromosuccinimide. The reaction mixture was stirred at 80 °C for 24 h. At this point, the reaction mixture was analyzed by ^{1}H NMR spectroscopy (Figure S12) and LCMS (Figure S14). The results are summarized in table S7.

Bromination of indomethacin methyl ester with condition C[10]

In a 4-mL borosilicate vial containing a Teflon-coated magnetic stirring bar, indomethacin methyl ester (112 mg, 0.300 mmol, 1.00 equiv.) was dissolved in HFIP (1 mL, c = 0.3 M). Then *N*-bromosuccinimide (53.4 mg, 0.300 mmol, 1.00 equiv) was added to the reaction mixture and stirred at 25 °C for 24 h. At this point, the reaction mixture was analyzed by ^{1}H NMR spectroscopy (Figure S12) and LCMS (Figure S15). The results are summarized in table S7.

Determination of selectivity of bromination and subsequent alkylation: indomethacin methyl ester

The reaction mixtures for the bromination of indomethacin methyl ester were analyzed by ^{1}H NMR spectroscopy and LCMS. LCMS traces and ^{1}H NMR analysis of the reaction mixture for the bromination of indomethacin methyl ester with condition A showed a mixture of mono-brominated products, di-brominated products, and tri-brominated products. The reaction mixture for the bromination of indomethacin methyl ester with condition B and condition C resulted in more complex mixtures. In all cases bromination selectivity could not be quantified. The reaction mixture for the bromination of indomethacin methyl ester with condition A was purified by column chromatography and used for the subsequent palladium-catalyzed alkylation. The alkylation reaction was analyzed by ^{1}H NMR spectroscopy and LCMS. No alkylated product formation was detected.

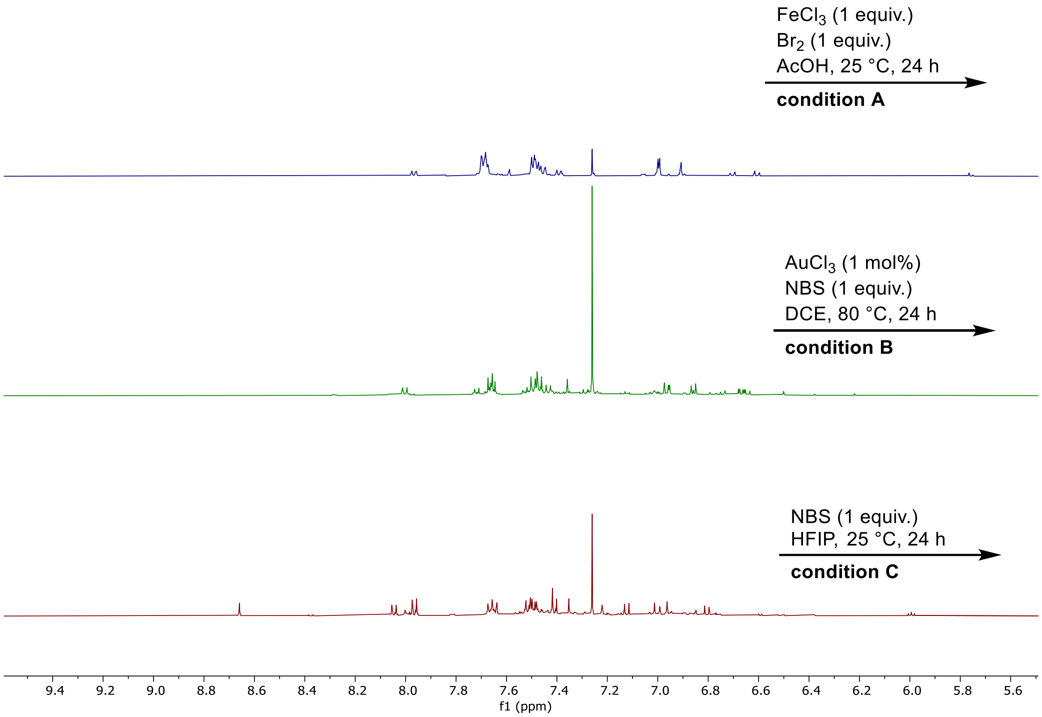

Figure S12: ^{1}H NMR analysis of the bromination of indomethacin methyl ester using various conditions prior to purification (conditions A, B, and C), $CDCl_3$, 500 MHz, 298 K.

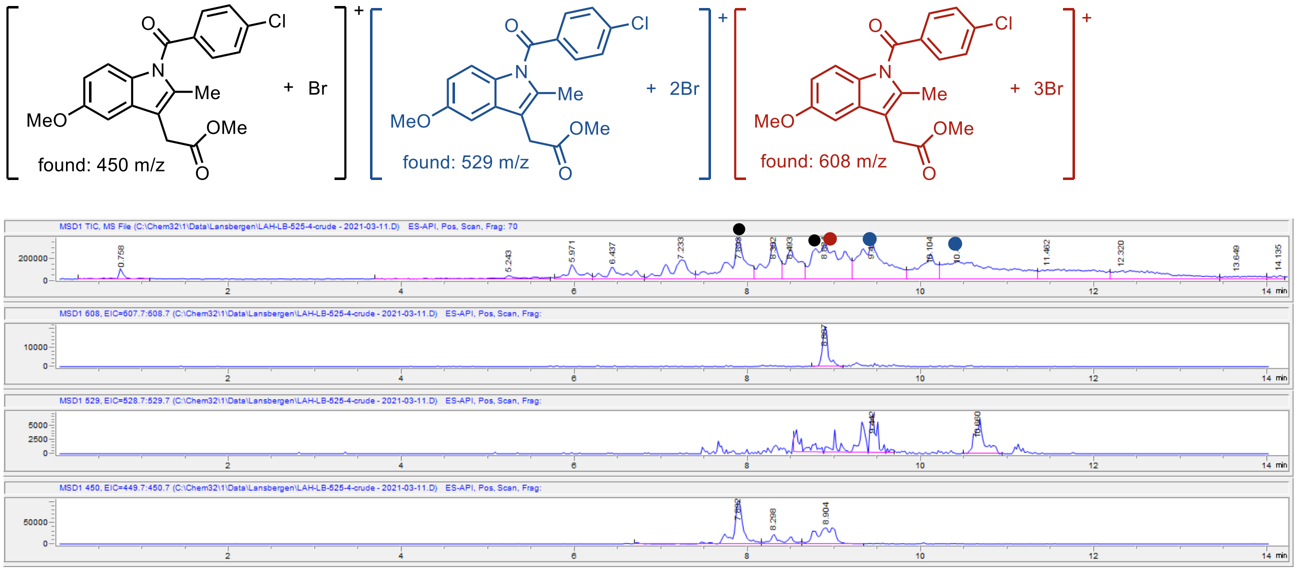

Figure S13: LCMS analysis of the bromination of indomethacin methyl ester with condition A

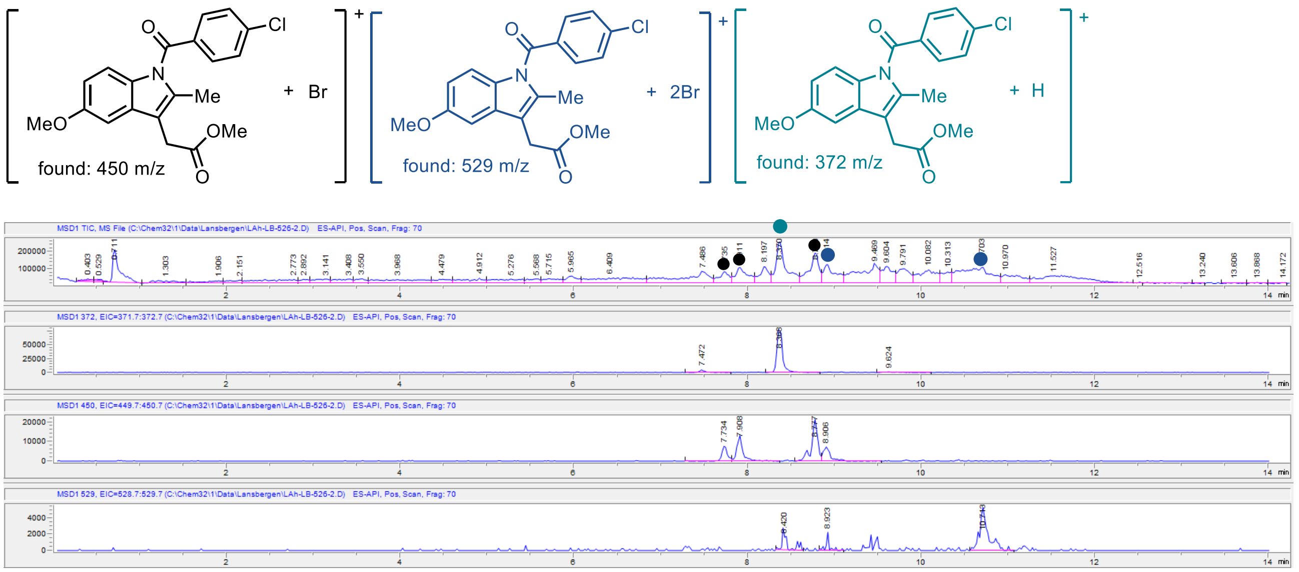

Figure S14: LCMS analysis of the bromination of indomethacin methyl ester with condition B

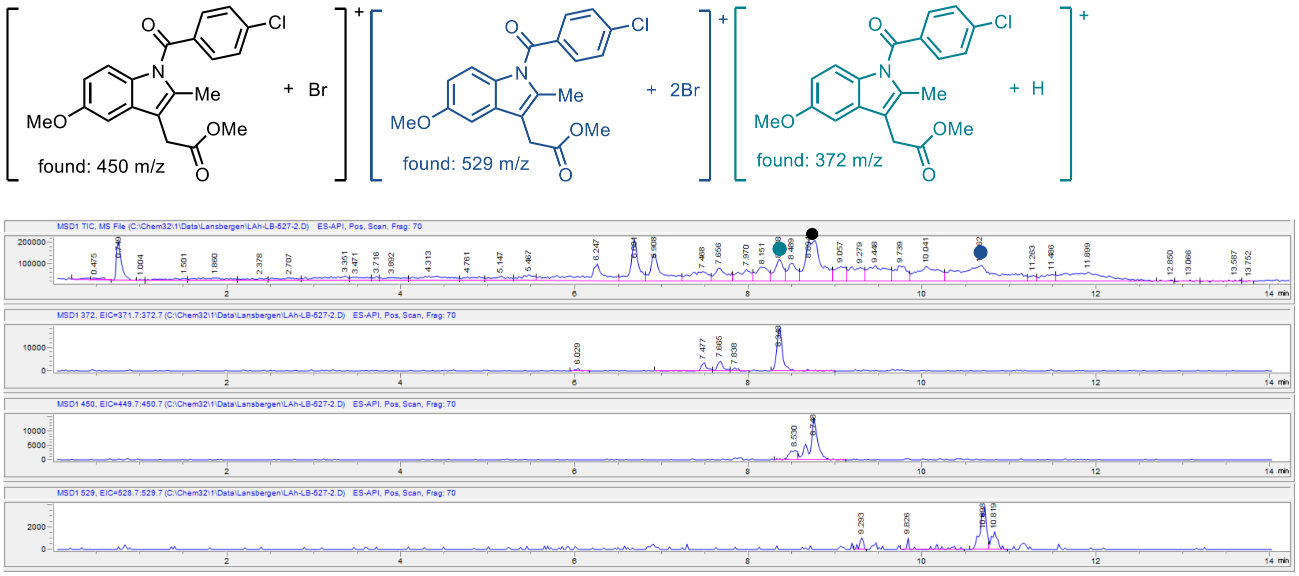

Figure S15: LCMS analysis of the bromination of indomethacin methyl ester with condition C

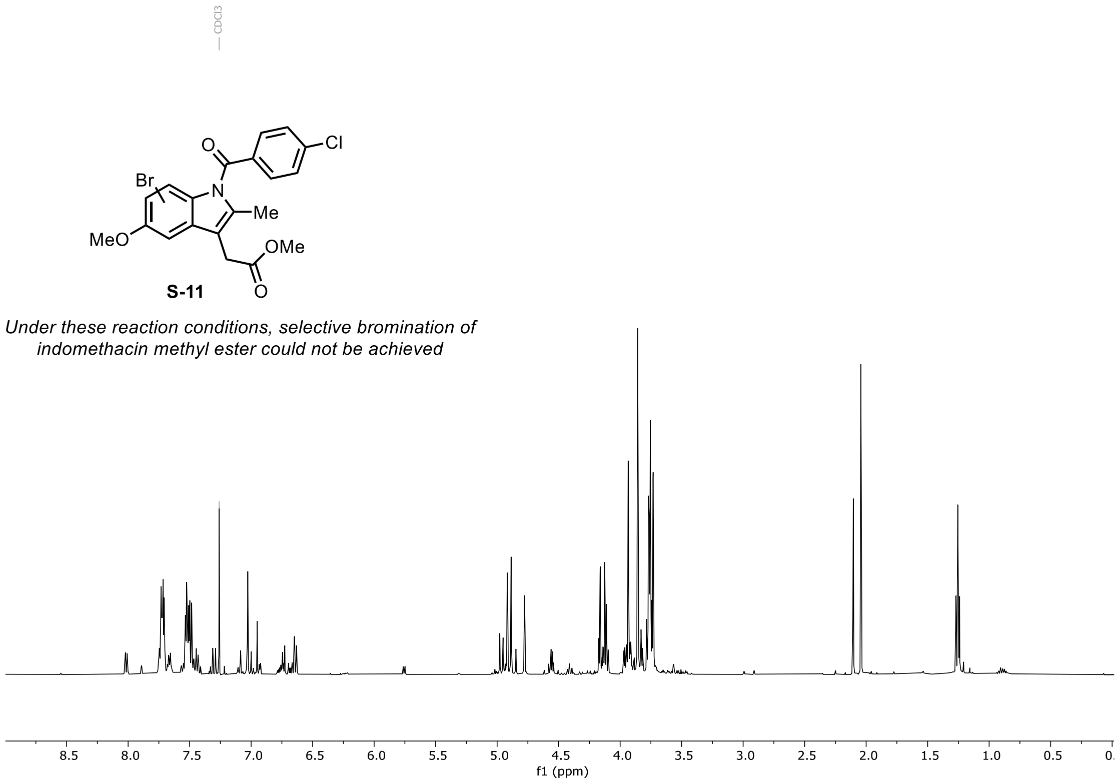

Figure S16: ^{1}H NMR of compound **S-11** as a mixture of brominated compounds using condition A after purification by column chromatography, $CDCl_3$, 500 MHz, 298 K. ^{1}H NMR shows that indomethacin methyl ester cannot undergo selective bromination and a mixture of brominated products must be used for the subsequent alkylation step.

^{1}H NMR analysis for the selective thianthrenation of indomethacin methyl ester[2, 4]

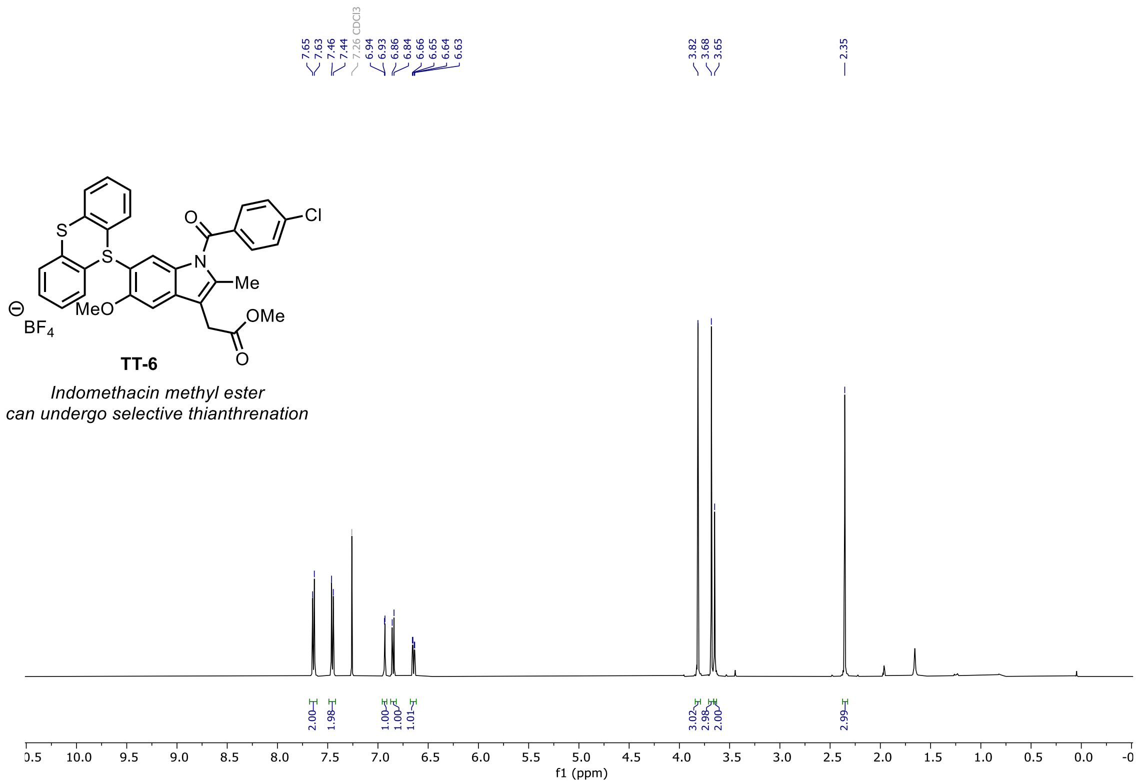

Figure S17: ^{1}H NMR of compound **TT-6** after purification by column chromatography, $CDCl_3$, 500 MHz, 298 K. ^{1}H NMR shows that indomethacin methyl ester can undergo selective thianthrenation and a pure compound can be used for the subsequent alkylation step (see page S22, compound **2** for the selective alkylation of **TT-6**).

NMR Spectroscopy:

^{1}H NMR (500 MHz, $CDCl_3$, 298 K) δ 7.64 (d, *J* = 8.5 Hz, 2H), 7.45 (d, *J* = 8.6 Hz, 2H), 6.93 (d, *J* = 2.5 Hz, 1H), 6.85 (d, *J* = 9.0 Hz, 1H), 6.65 (dd, *J* = 9.0, 2.5 Hz, 3H), 3.82 (s, 3H), 3.68 (s, 2H), 2.35 (s, 3H).

Palladium-catalyzed alkylation of S-11

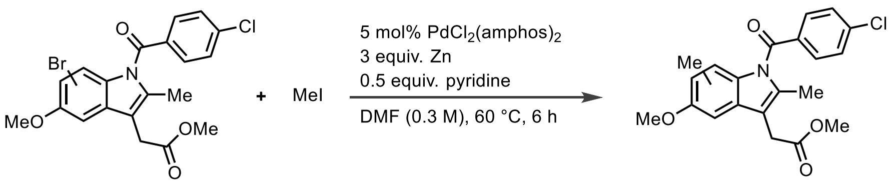

S-11 **2, not detected**

To a 4-mL borosilicate vial containing a Teflon-coated magnetic stirring bar were added bromo indomethacin methyl ester reaction mixture **S-11** (158 mg, 0.300 mmol, 1.00 equiv.), $PdCl_2(amphos)_2$ (10.6 mg, 15.0 µmol, 5.00 mol%) and activated zinc dust (58.9 mg, 0.900 mmol, 3.00 equiv.). The vial was transferred into a nitrogen-filled glovebox. Dry DMF (1 mL, c = 0.3 M) was added to the solids. Subsequently, methyl iodide

(37 µL, 85 mg, 0.60 mmol, 2.0 equiv.) and pyridine (12 µL, 12 mg, 0.15 mmol, 0.50 equiv.) were added at 25 °C. The vial was sealed with a Teflon-lined screw cap, removed from the glovebox, and transferred to a heating block preheated at 60 °C where the reaction mixture was stirred rigorously (850 rpm) for 6 h. A color change from yellow to dark brown was observed. The mixture was cooled to 25 °C, subsequently diluted with ethyl acetate (40 mL), and thereafter poured into a separatory funnel. The organic layer was washed with water (1 × 40 mL). The aqueous layer was then extracted with ethyl acetate (3 × 40 mL). The organic layers were combined, dried over $MgSO_4$, filtered, and concentrated under reduced pressure. The resulting residue was purified by column chromatography on silica gel eluting with a solvent mixture of hexanes / ethyl acetate, (1:0 gradient to 0:1 (v/v)). The resulting fractions collected were analyzed by LCMS, GCMS, ^{1}H NMR spectroscopy, and ^{13}C NMR spectroscopy. No formation of product **2** could be accurately confirmed.

Summary of results of the methylation of indomethacin methyl ester via bromination vs. thianthrenation

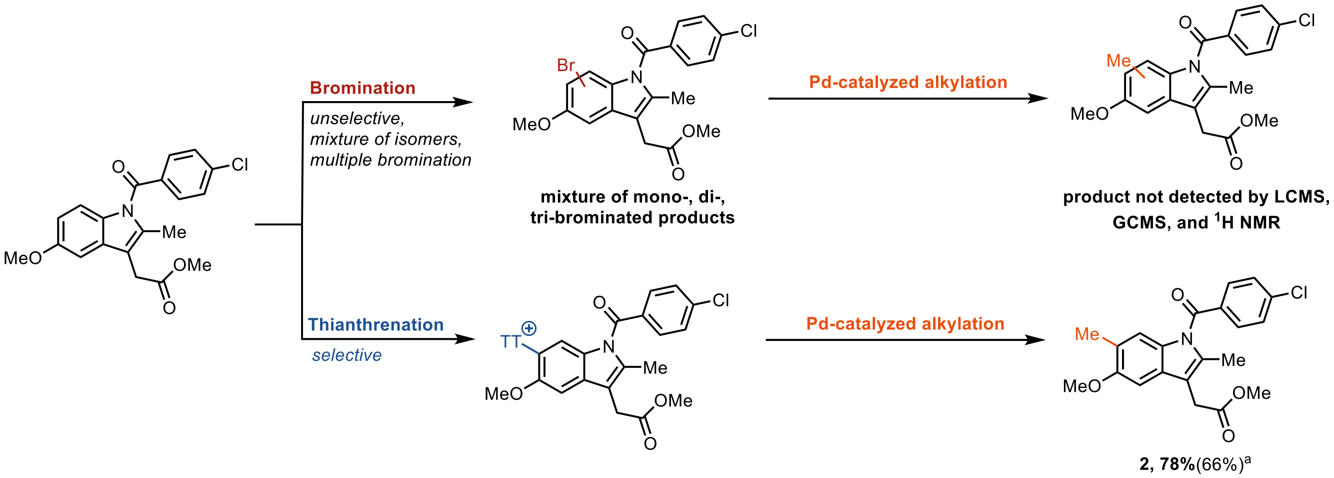

[a] Two-step yield.

SPECTROSCOPIC DATA

^{1}H NMR of estrone methyl ether thianthrenium salt derivative TT-4

CD_3CN, 23 °C

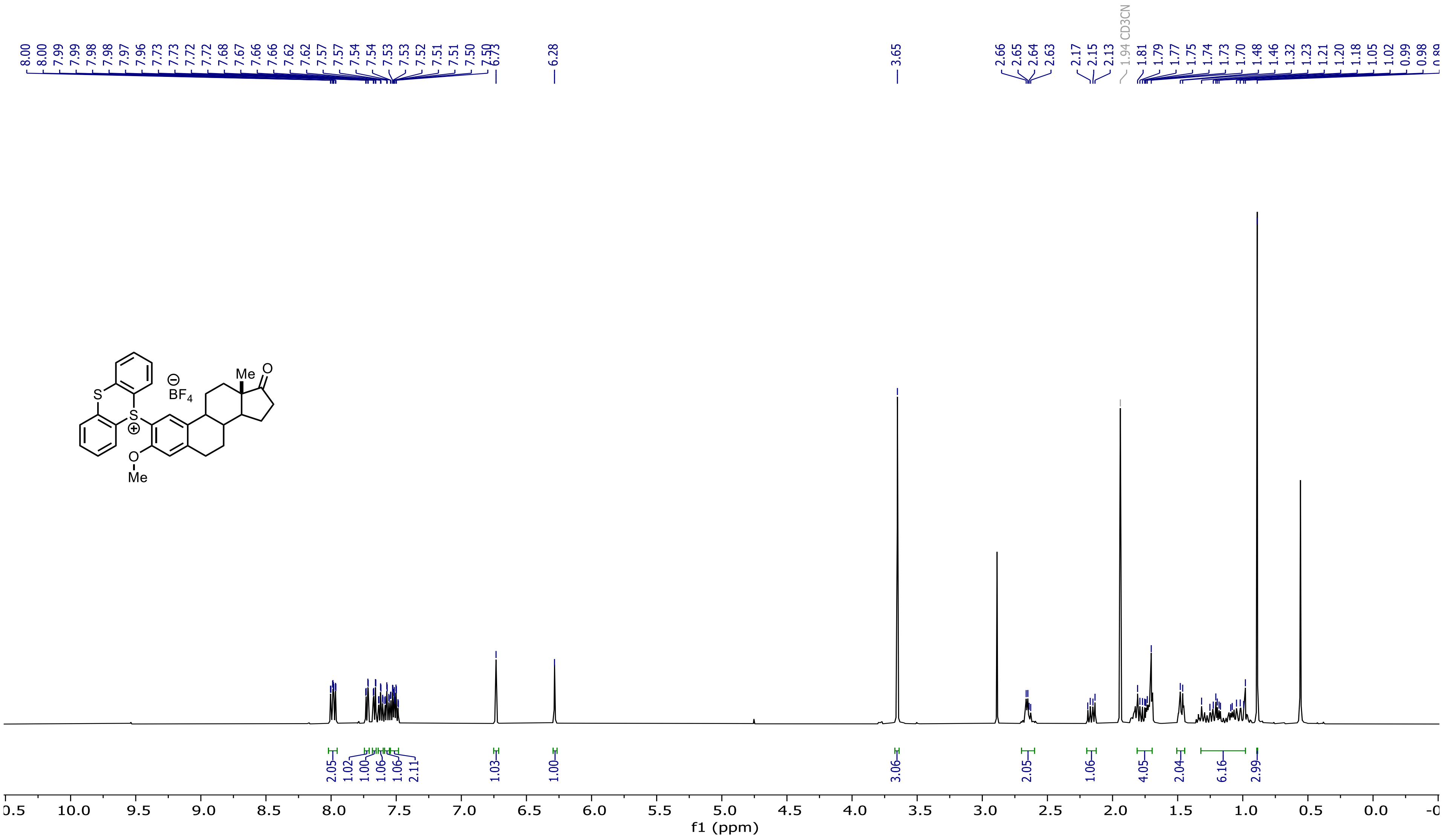

^{13}C NMR of estrone methyl ether thianthrenium salt derivative TT-4

CD_3CN, 23 °C

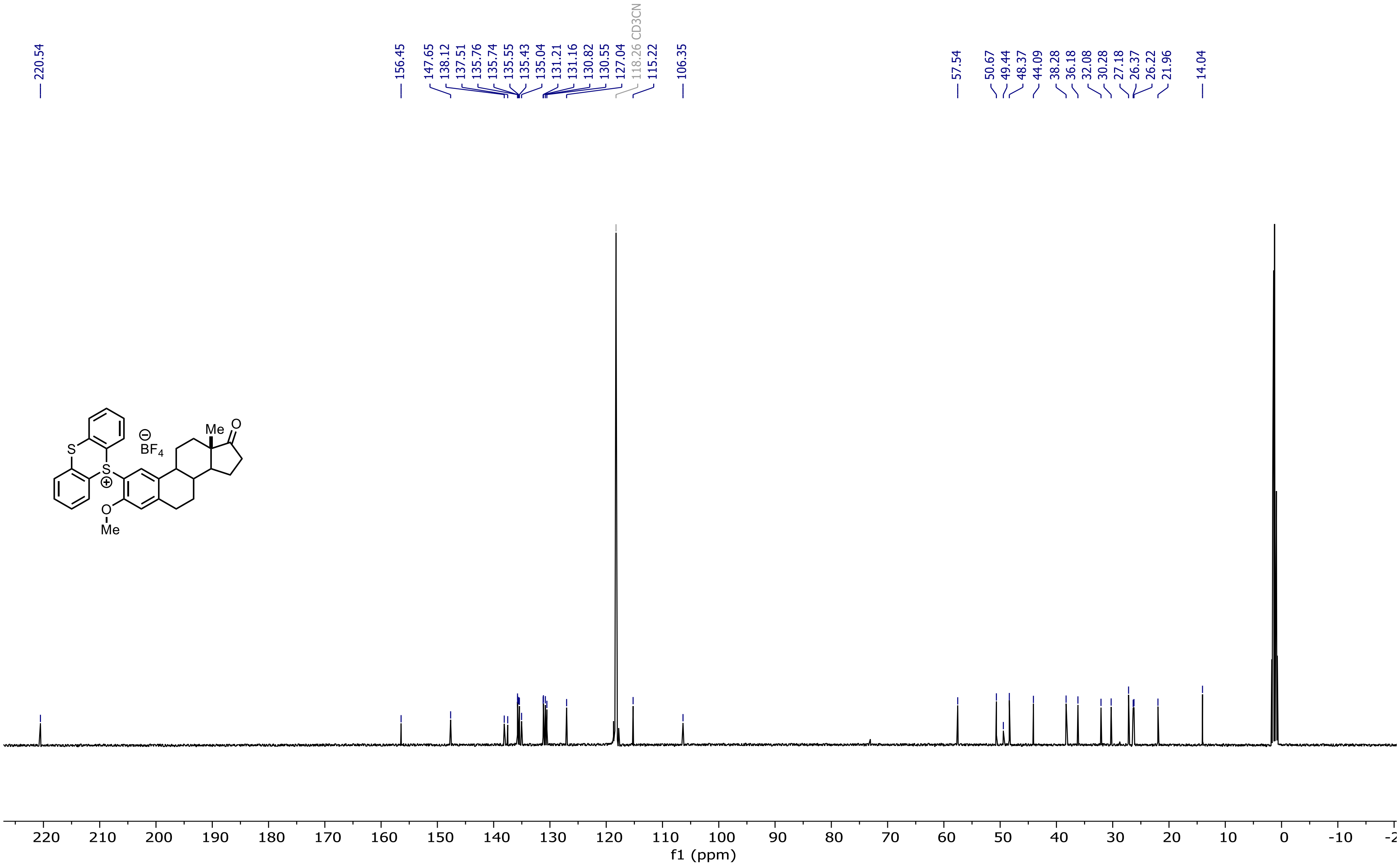

^{19}F NMR of estrone methyl ether thianthrenium salt derivative TT-4

CD_3CN, 23 °C

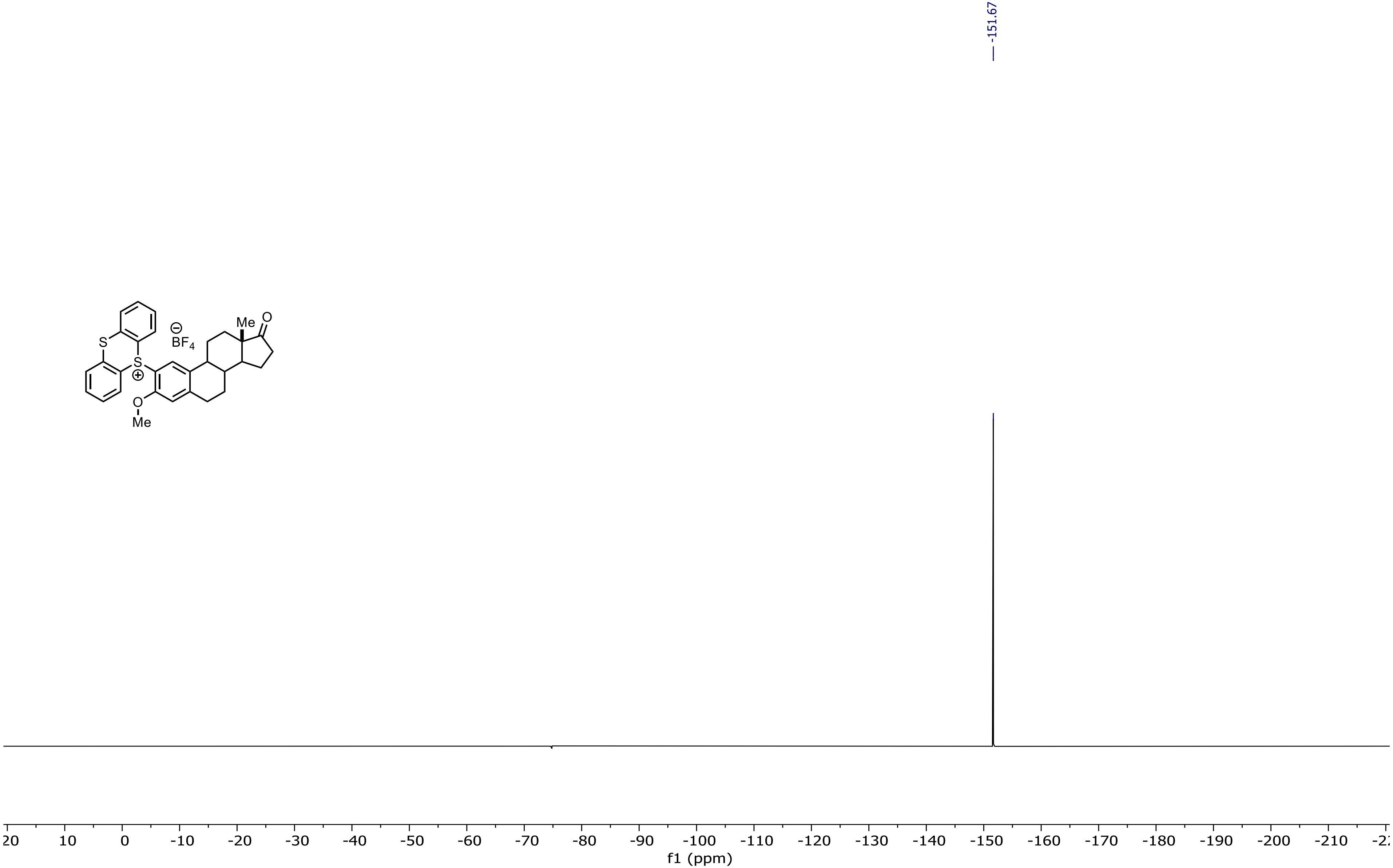

^{1}H NMR of sulbactam iodide derivative S-2

CDCl$_3$, 23 °C

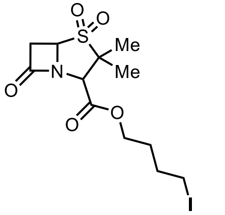

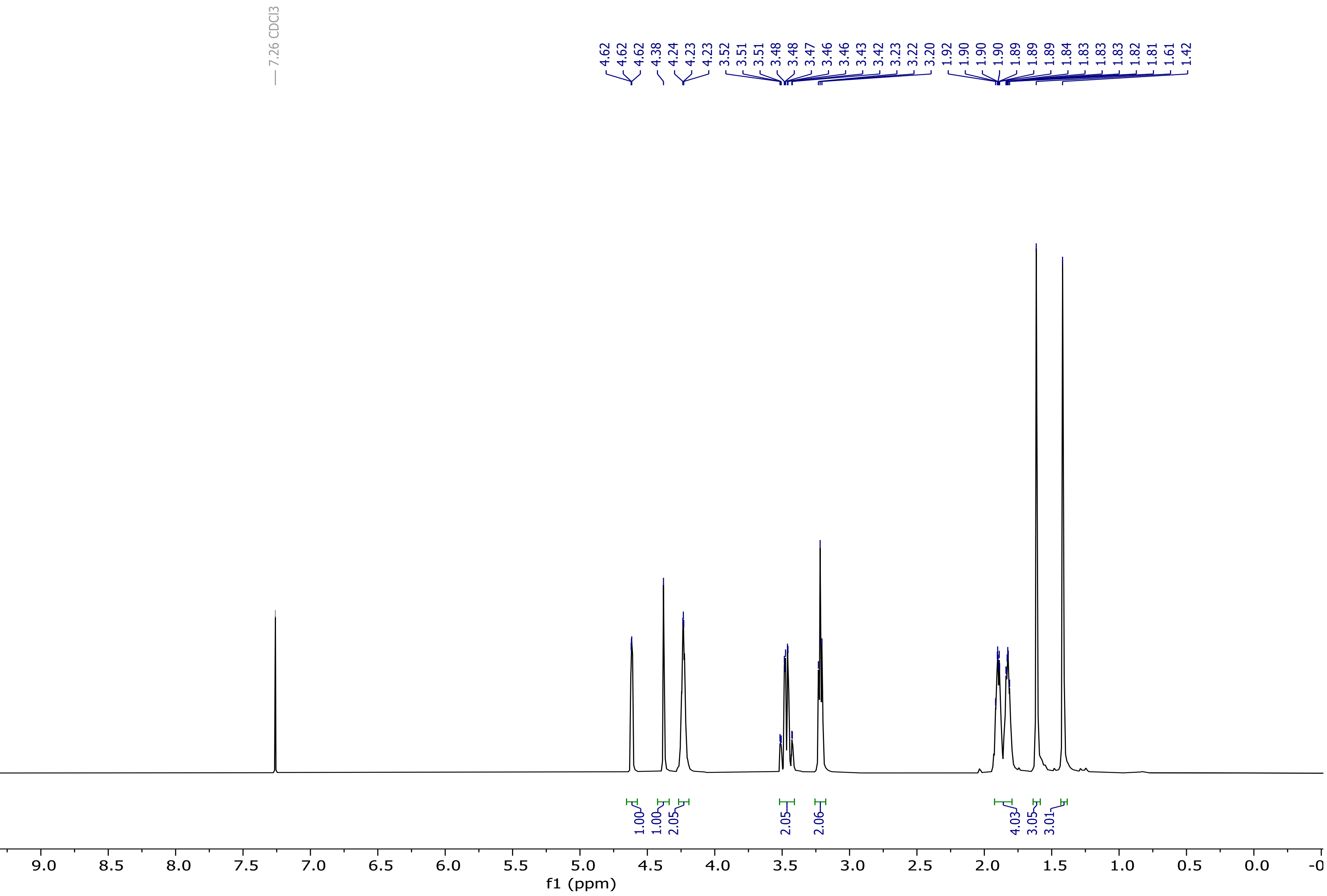

^{13}C NMR of sulbactam iodide derivative S-2

$CDCl_3$, 23 °C

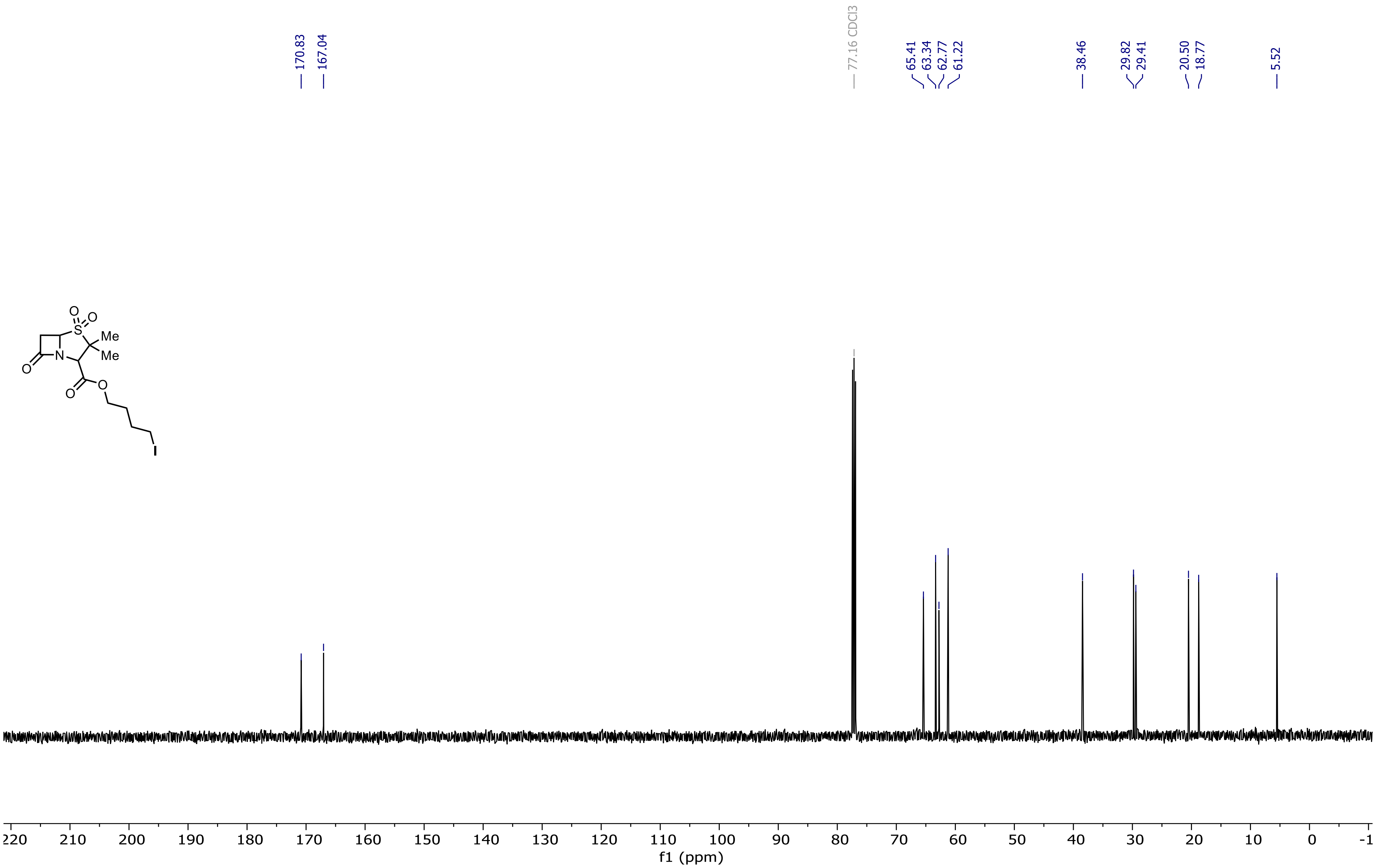

^{1}H NMR of Fmoc-Arg(Pbf)-OH iodide derivative S-3

$CDCl_3$, 23 °C

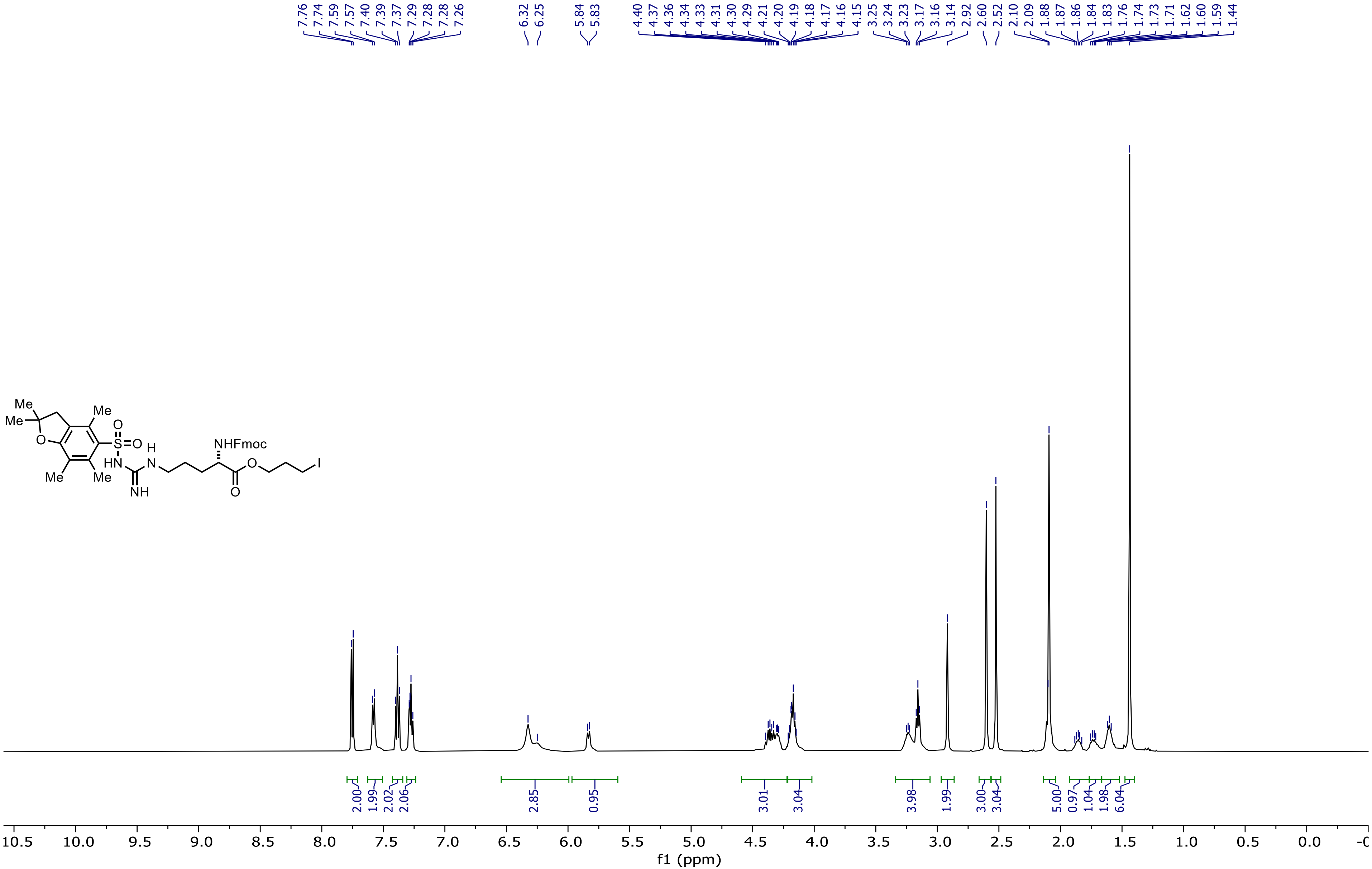

^{13}C NMR of Fmoc-Arg(Pbf)-OH iodide derivative S-3

$CDCl_3$, 23 °C

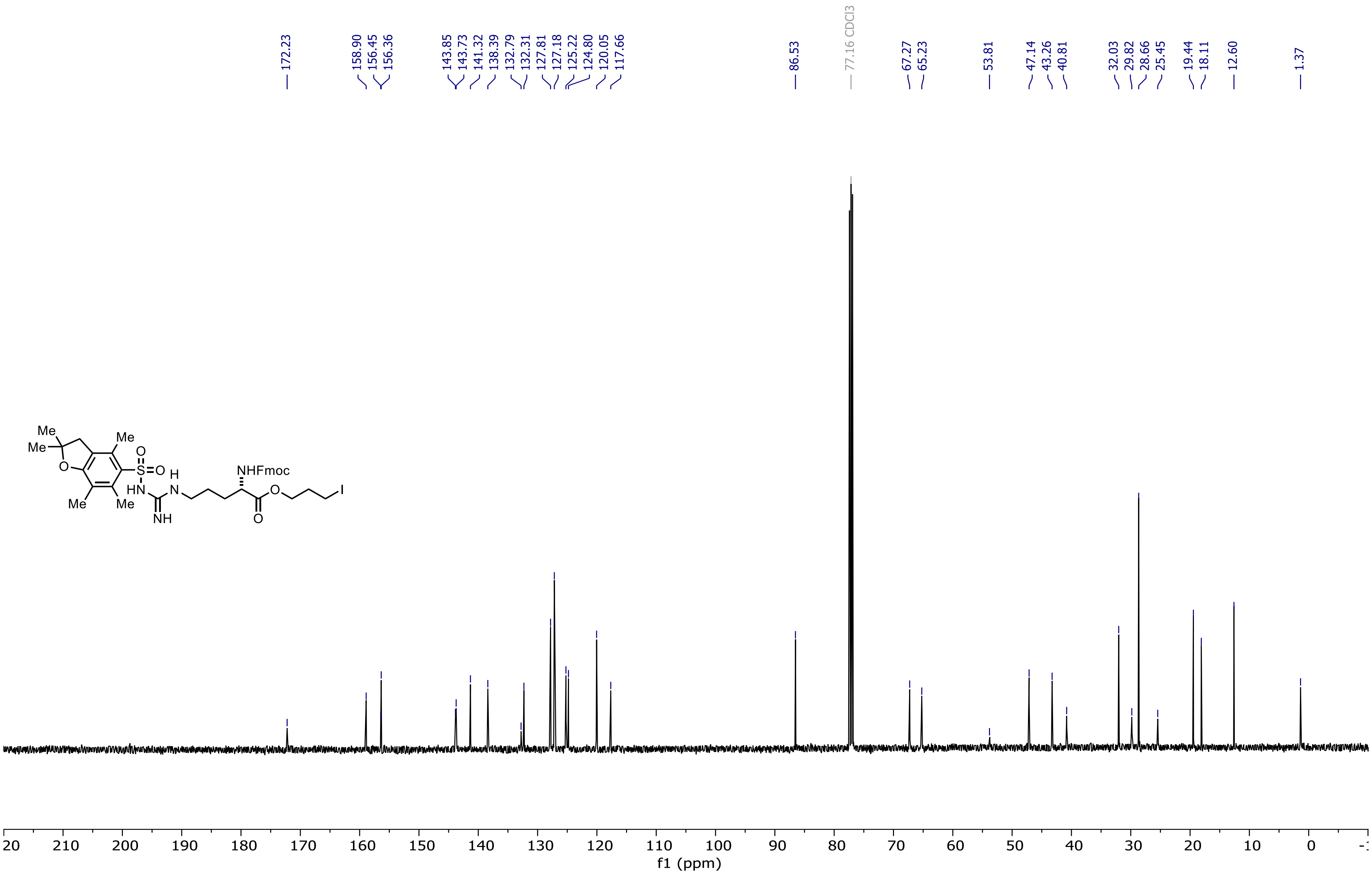

^{1}H NMR of 3-iodo epiandrosterone derivative S-4

$CDCl_3$, 23 °C

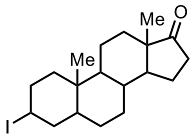

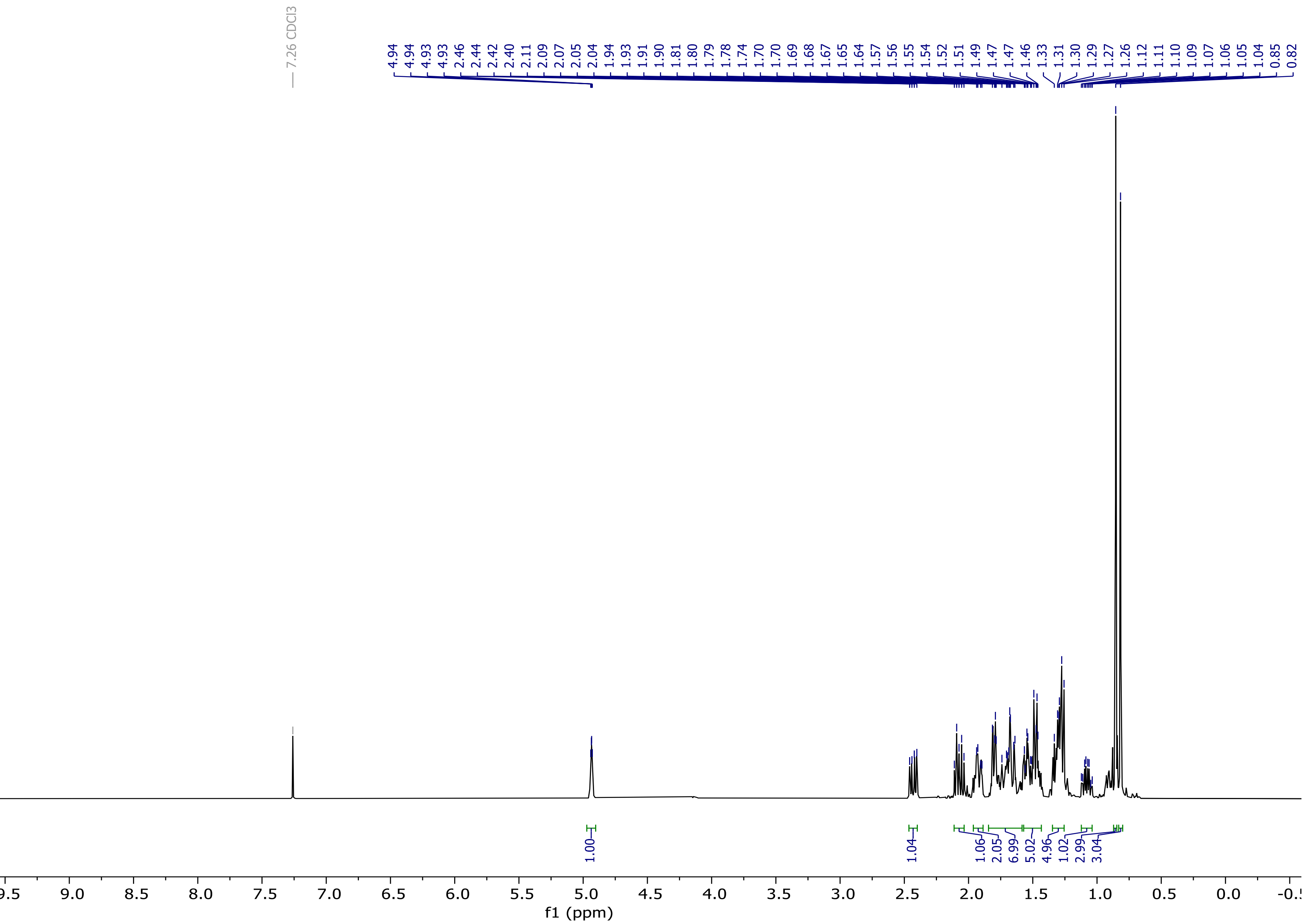

^{13}C NMR of 3-iodo epiandrosterone derivative S-4

$CDCl_3$, 23 °C

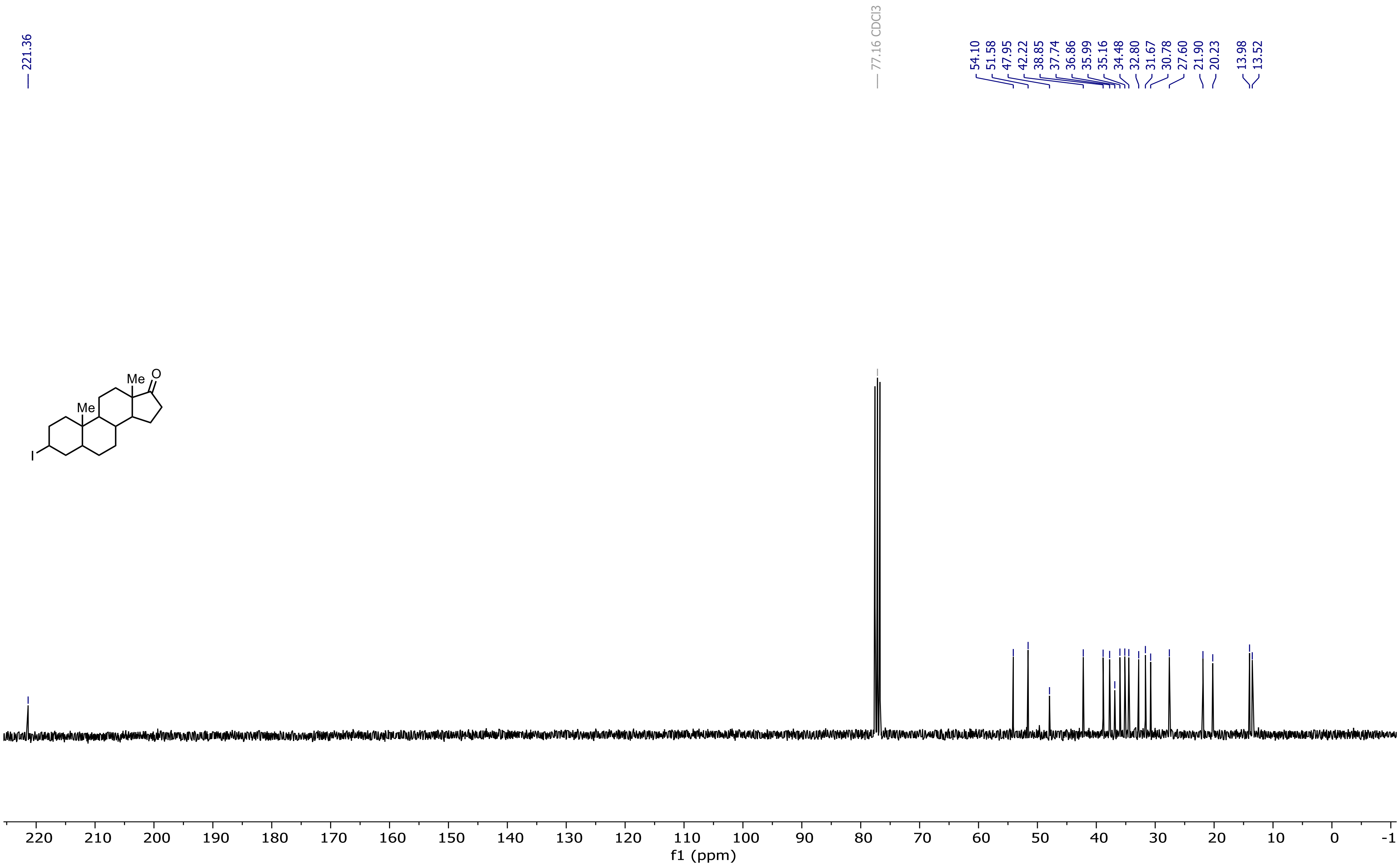

^{1}H NMR of methyl bifonazole derivative 1

$CDCl_3$, 23 °C

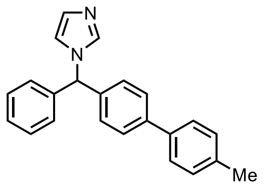

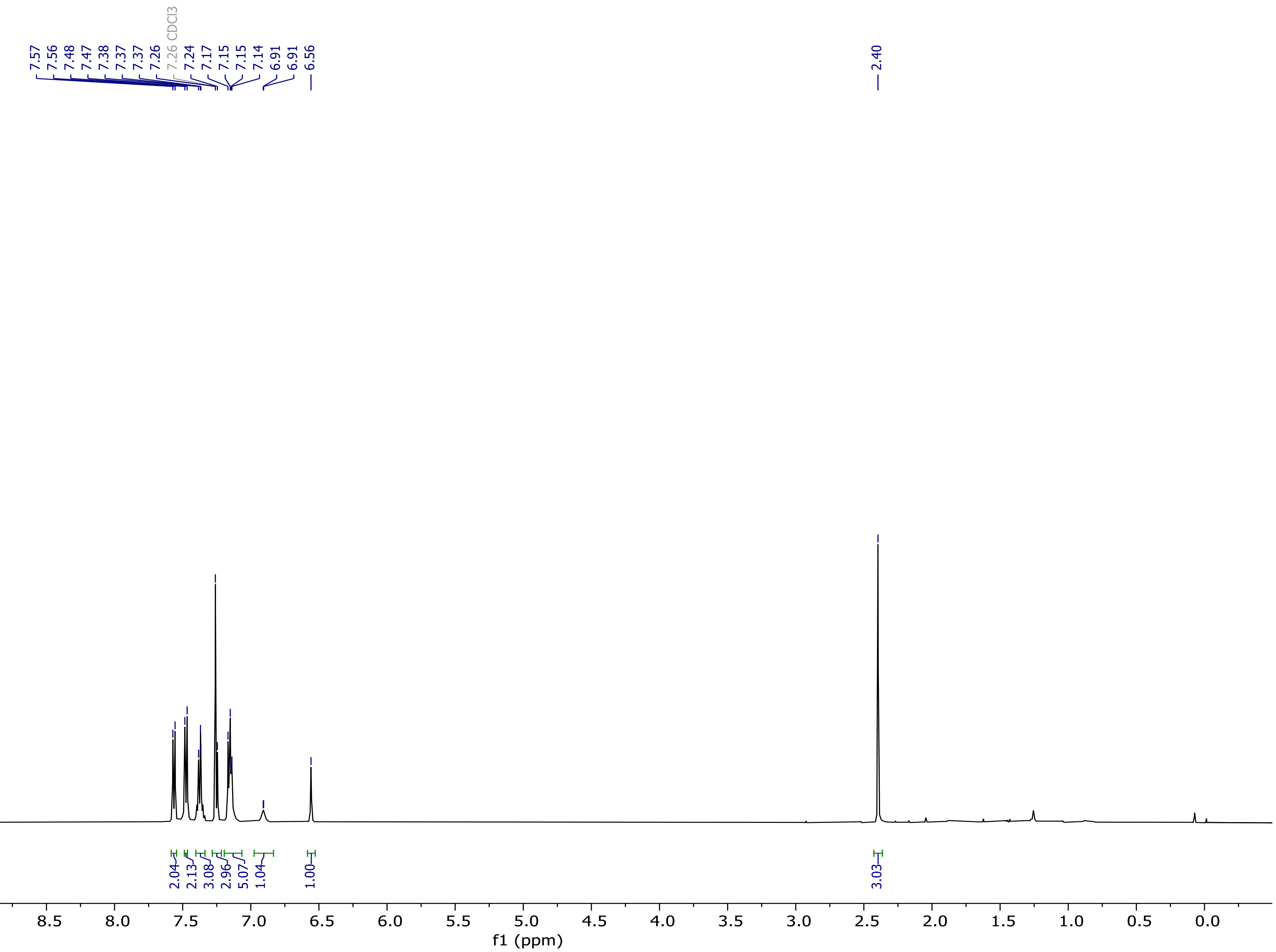

^{13}C NMR of methyl bifonazole derivative 1

$CDCl_3$, 23 °C

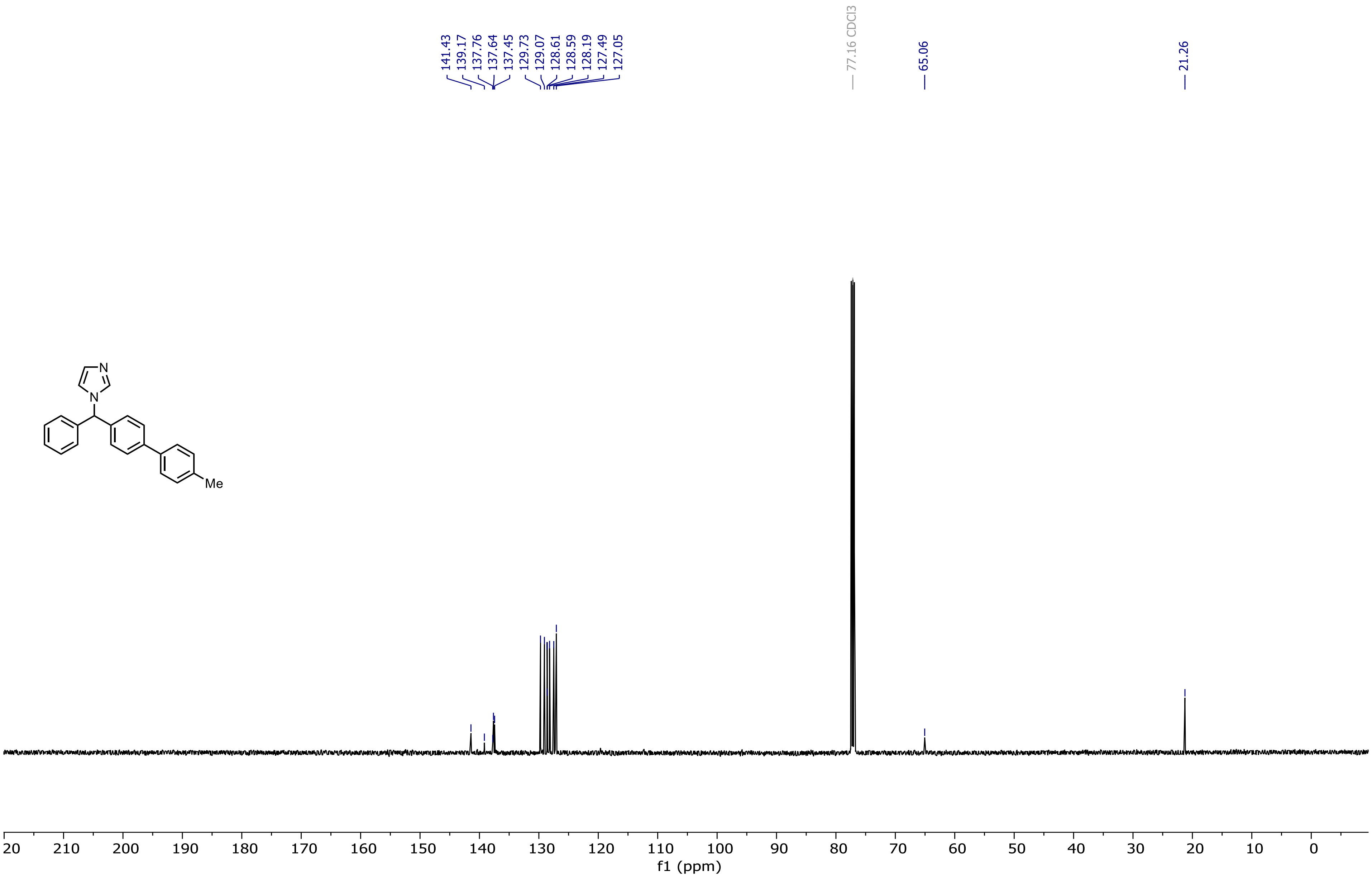

^{1}H NMR of methyl indomethacin methyl ester derivative 2

$CDCl_3$, 23 °C

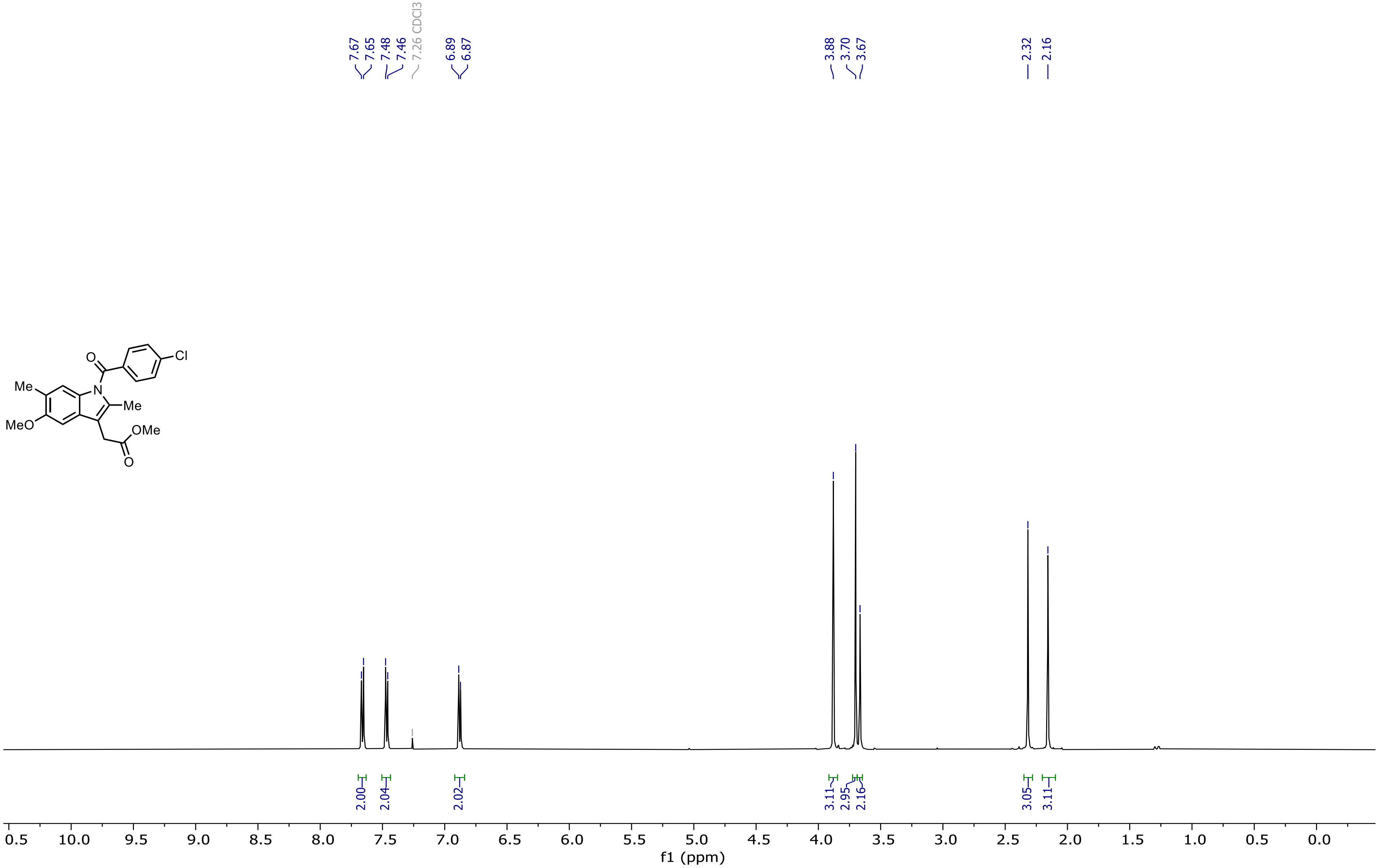

^{13}C NMR of methyl indomethacin methyl ester derivative 2

$CDCl_3$, 23 °C

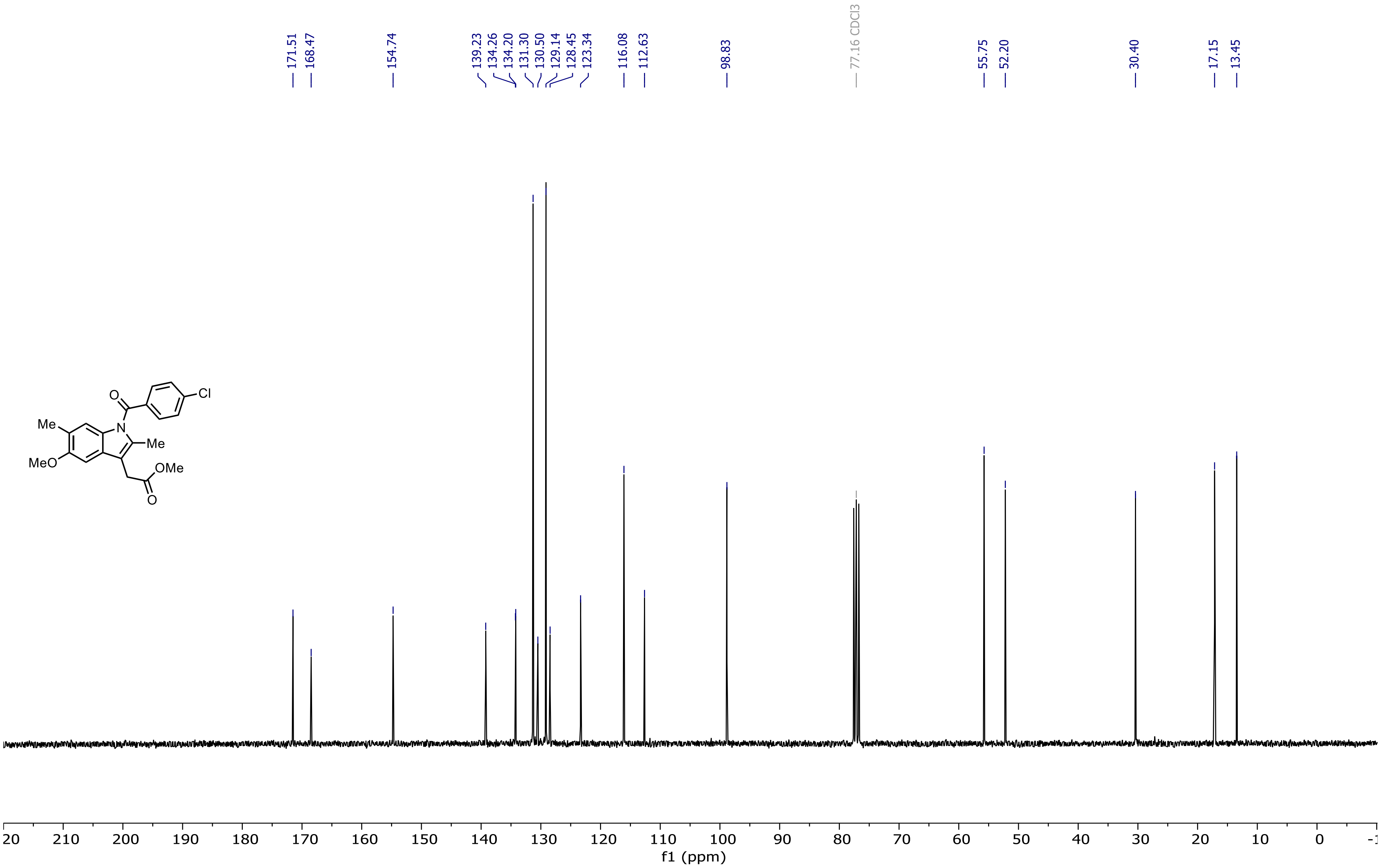

^{1}H NMR of isopropyl pyriproxyfen derivative 4

CDCl3, 23 °C

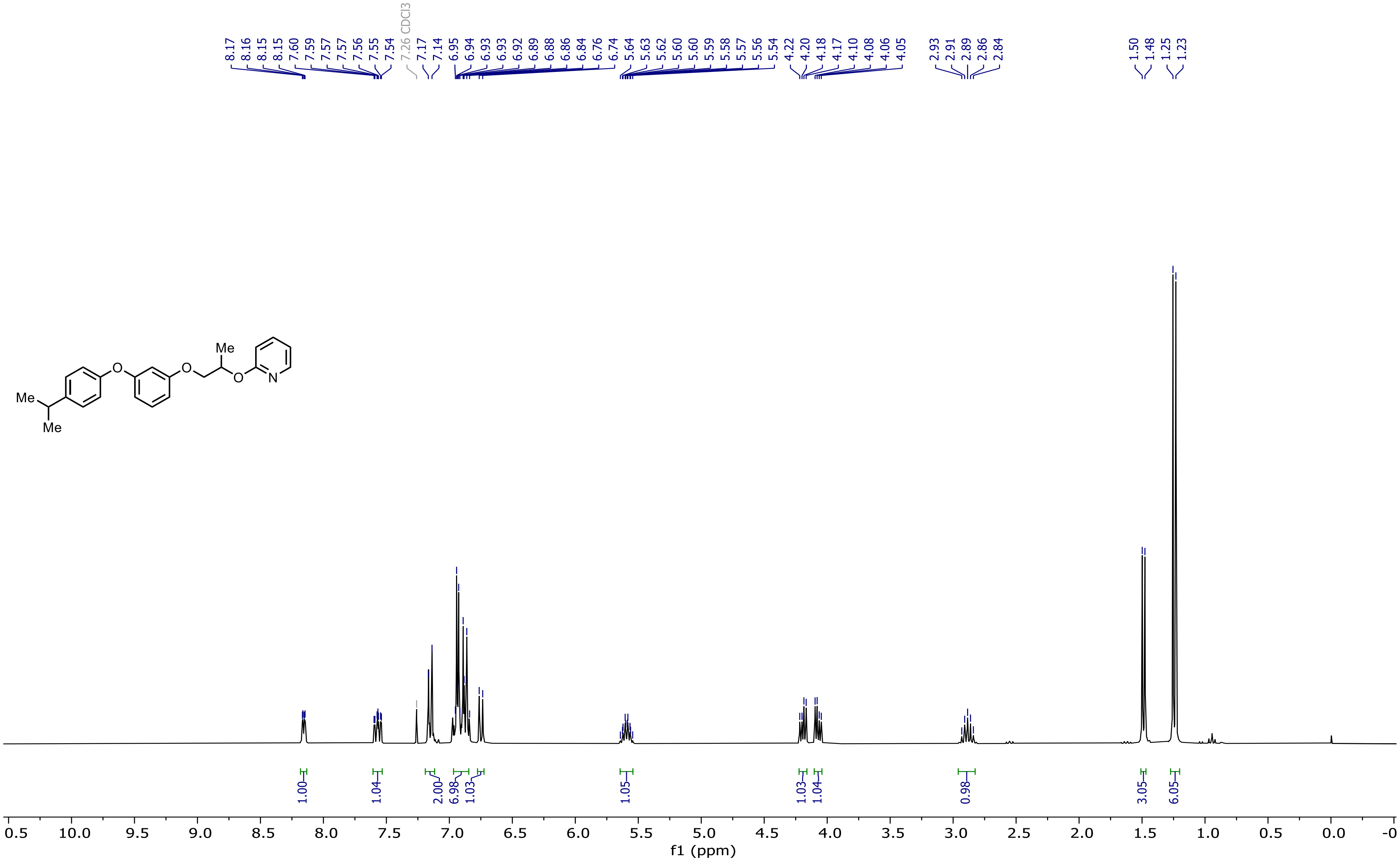

^{13}C NMR of isopropyl pyriproxyfen derivative 4

$CDCl_3$, 23 °C

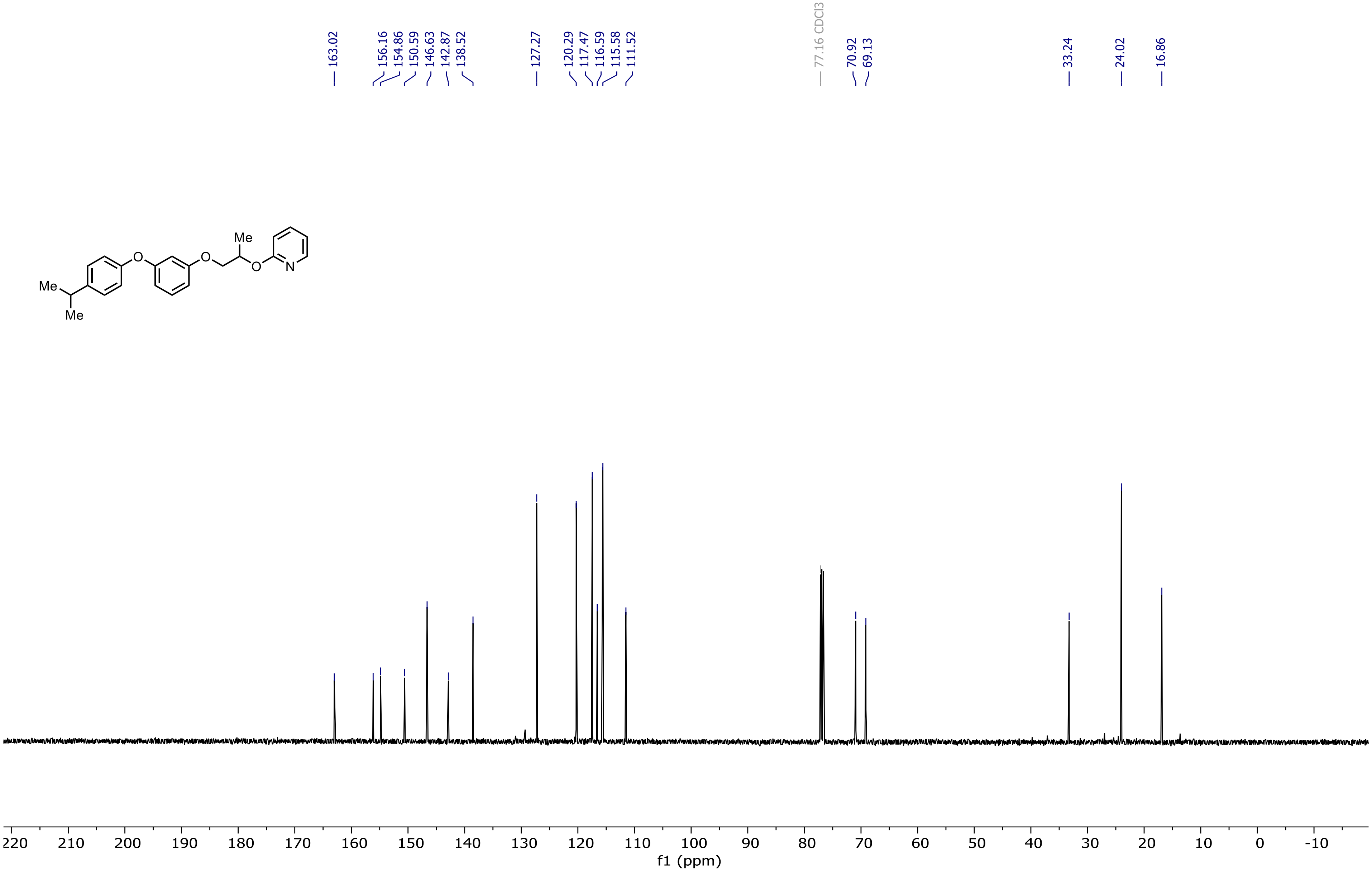

^{1}H NMR of *n*-propyl pyriproxyfen derivative S-1

$CDCl_3$, 23 °C

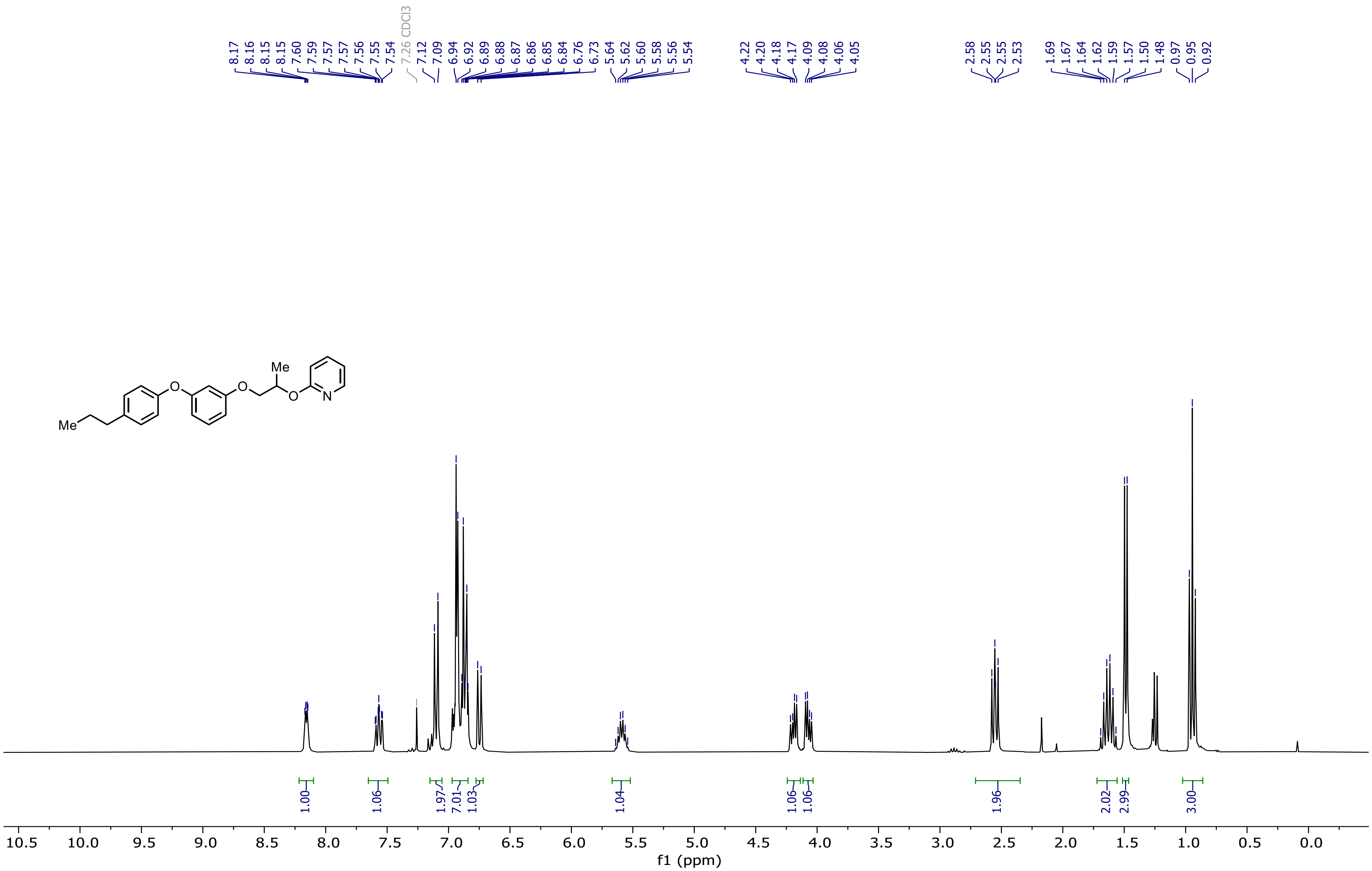

^{13}C NMR of *n*-propyl pyriproxyfen derivative S-1

$CDCl_3$, 23 °C

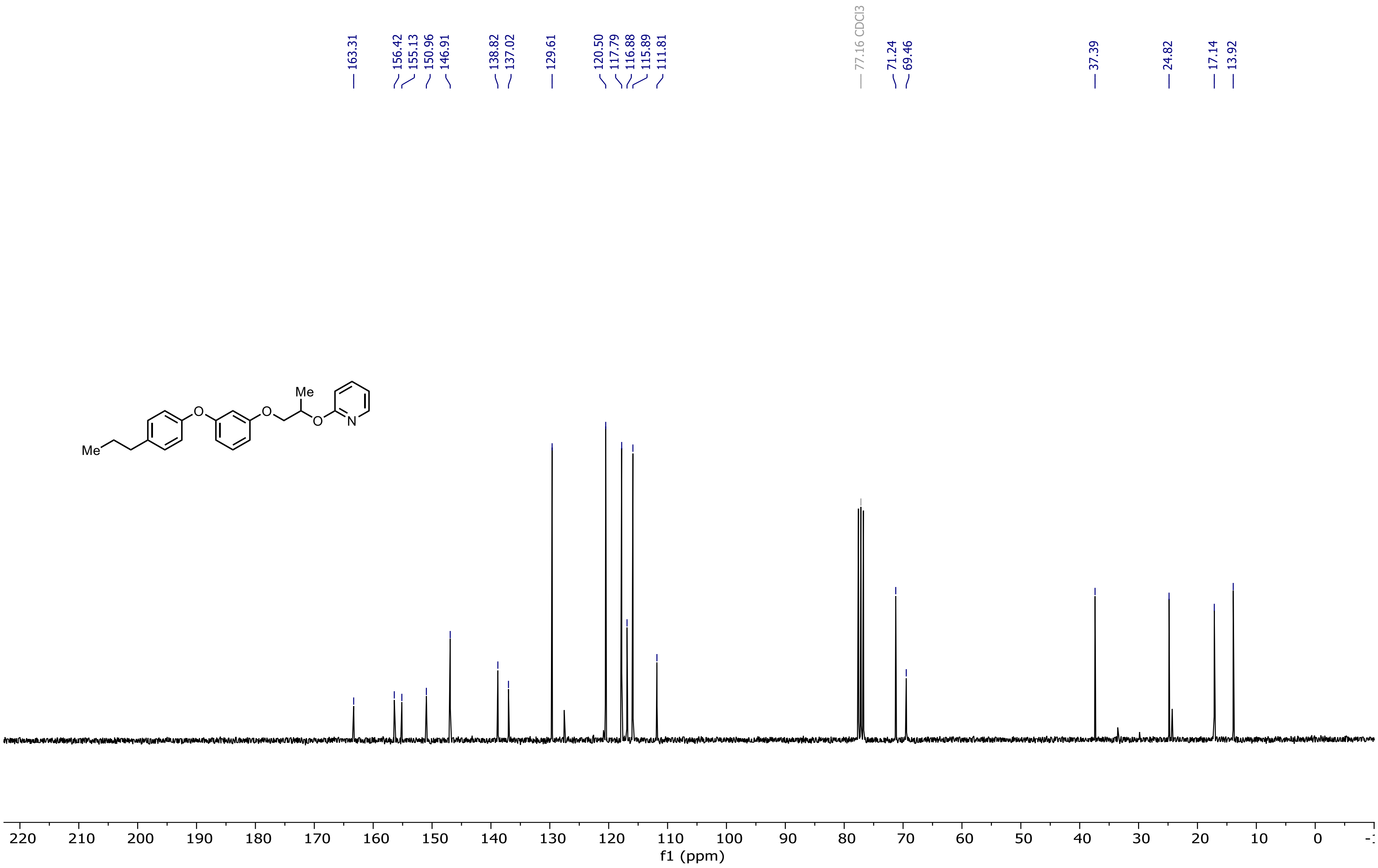

^{1}H NMR of boc-azetidinyl indomethacin methyl ester derivative 5

CDCl₃, 23 °C

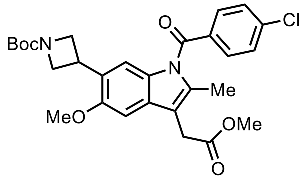

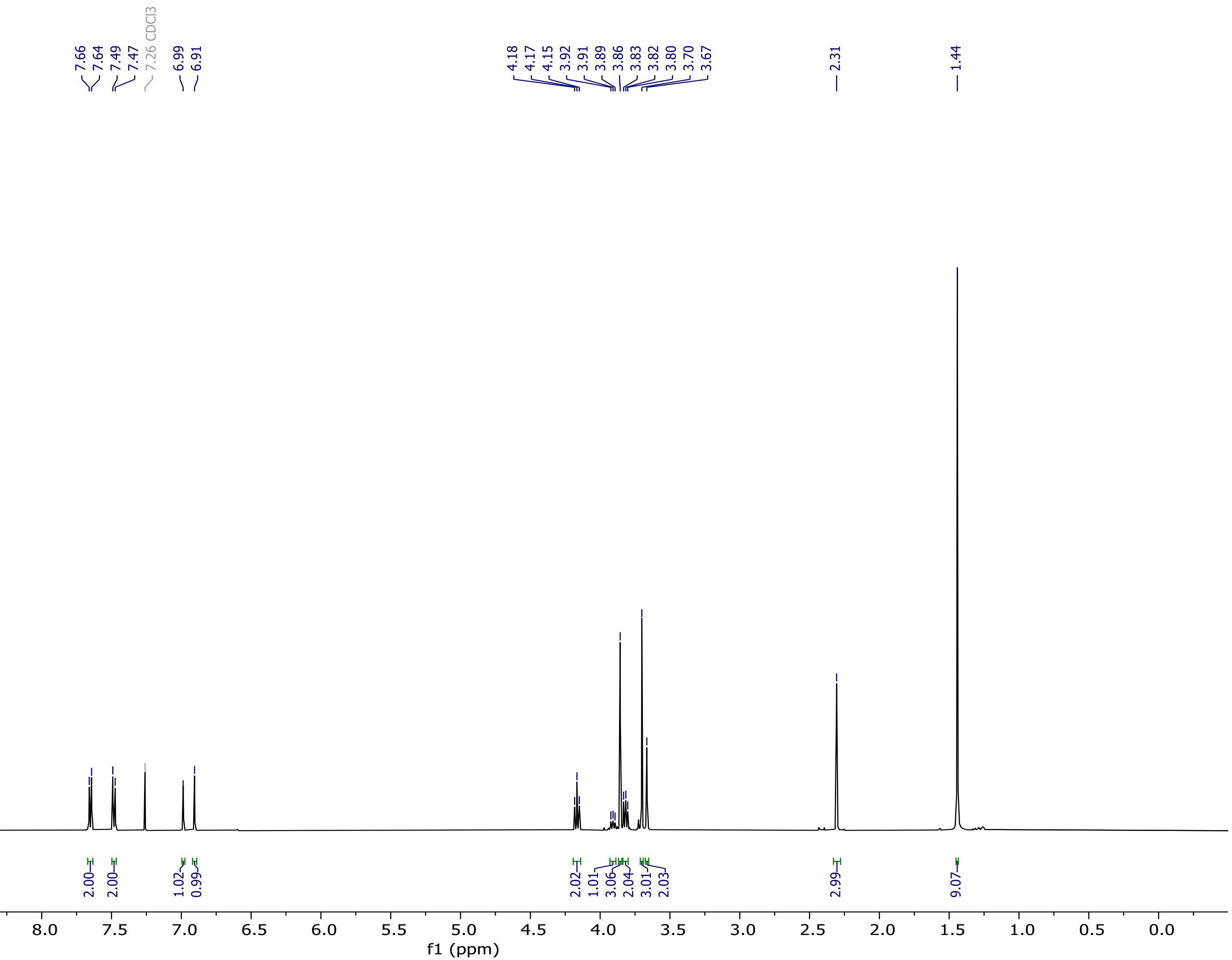

^{13}C NMR of boc-azetidinyl indomethacin methyl ester derivative 5

$CDCl_3$, 23 °C

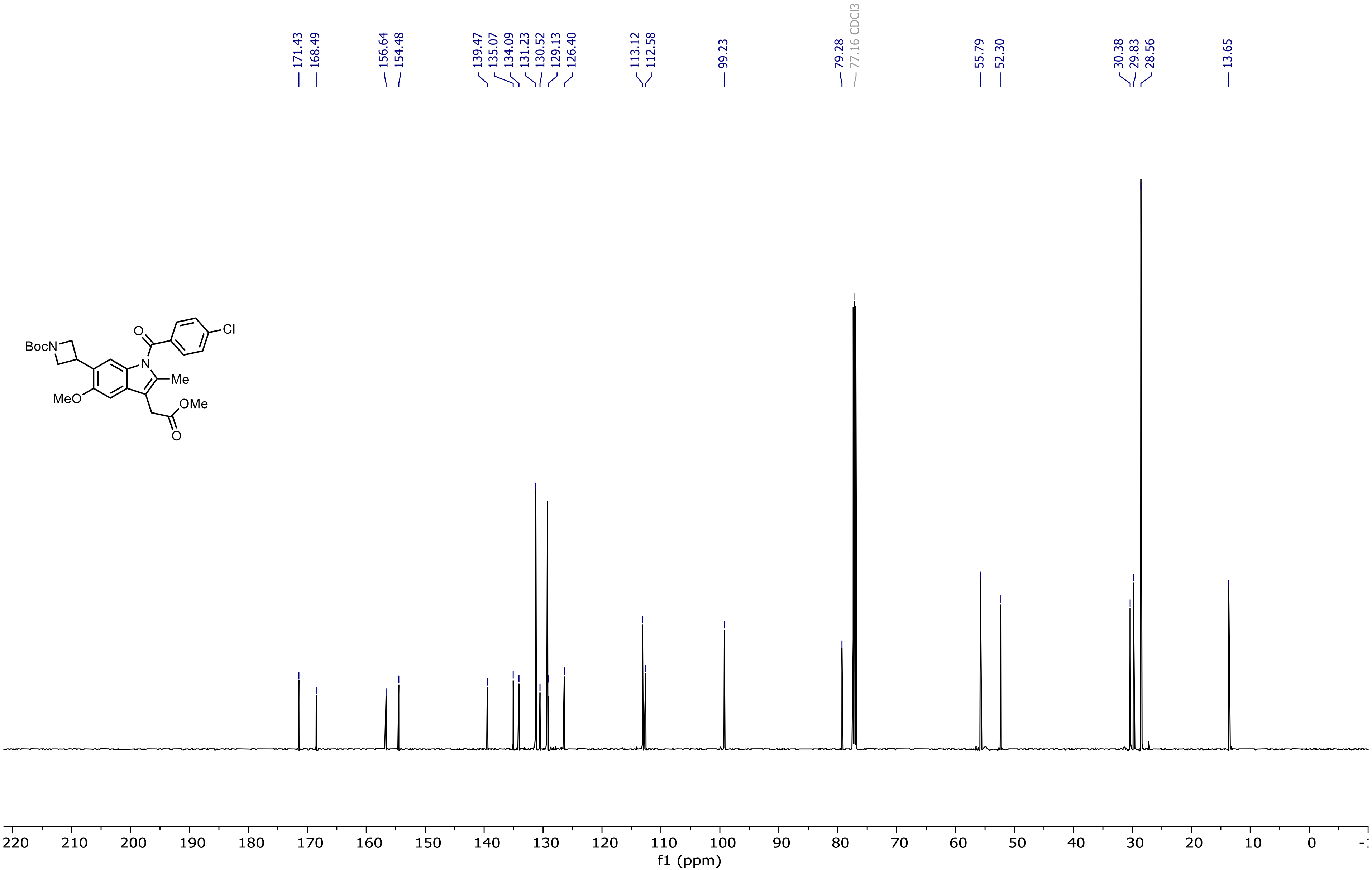

^{1}H NMR of tridecafluorooctyl benzyloxazolidinone derivative 6

CDCl$_3$, 23 °C

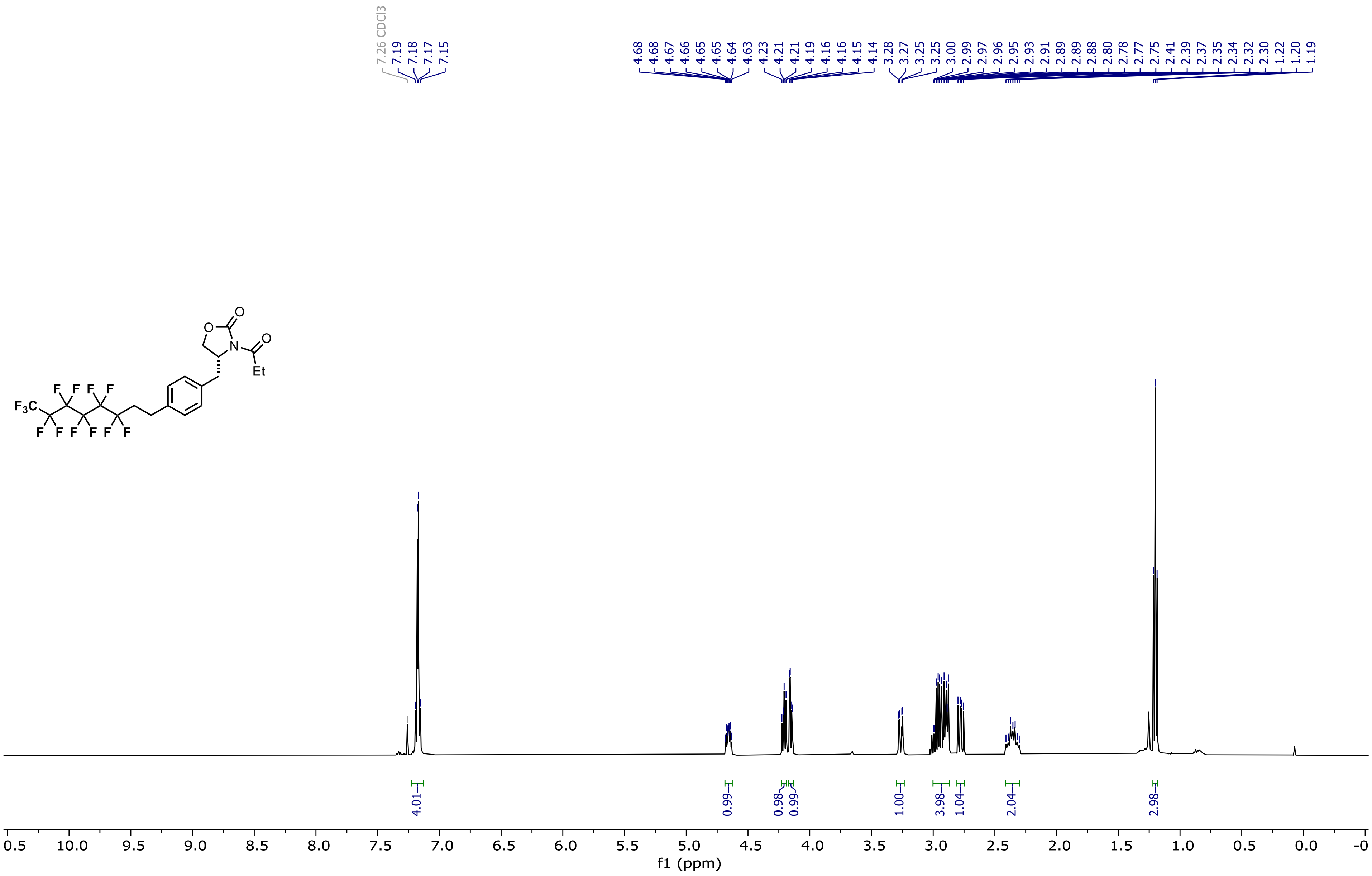

^{13}C NMR of tridecafluorooctyl benzyloxazolidinone derivative 6

$CDCl_3$, 23 °C

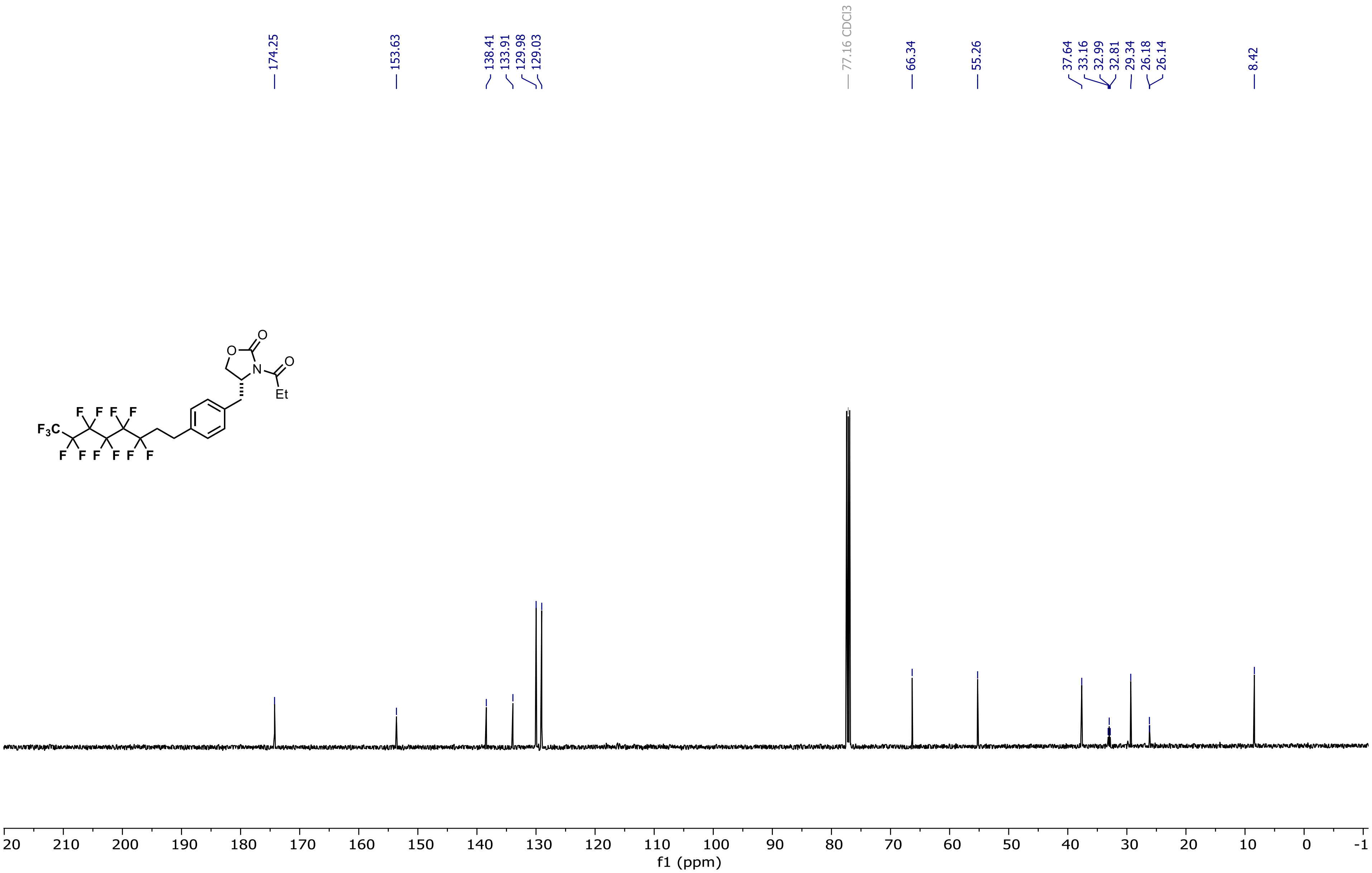

^{19}F NMR of tridecafluorooctyl benzyloxazolidinone derivative 6

CDCl$_3$, 23 °C

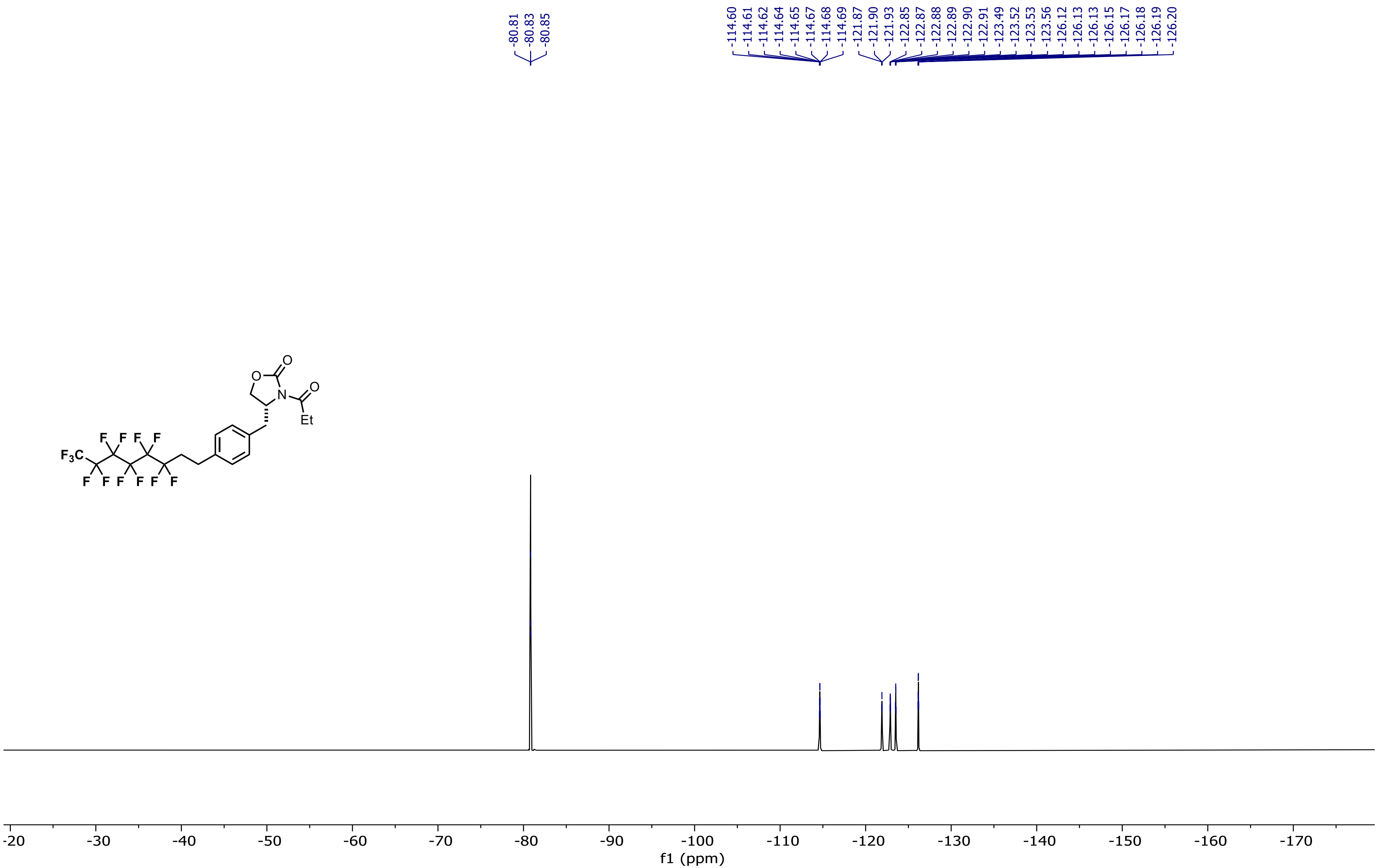

^{1}H NMR of (methyl)trimethylsilyl pyriproxyfen derivative 7

CDCl$_3$, 23 °C

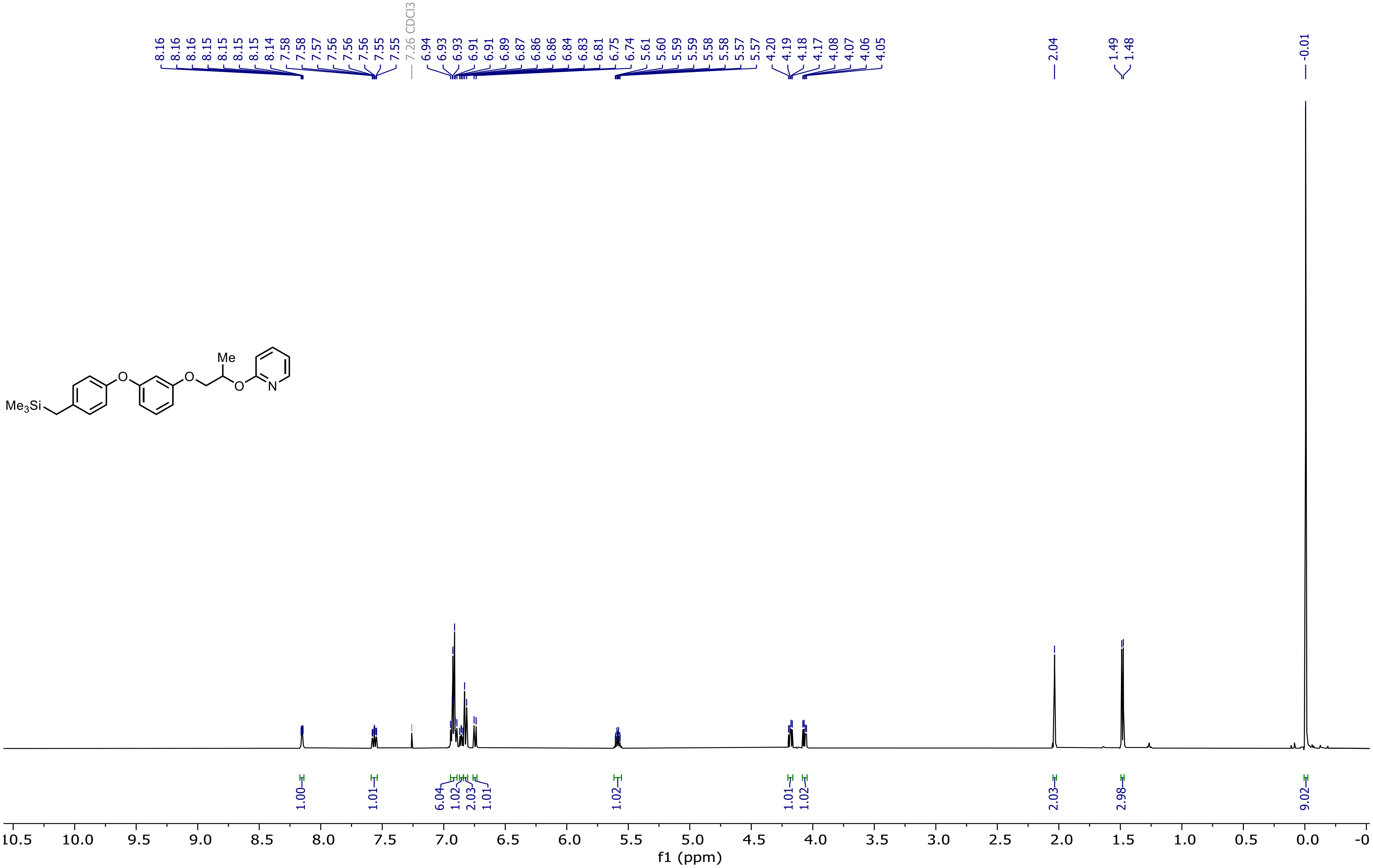

^{13}C NMR of (methyl)trimethylsilyl pyriproxyfen derivative 7

CDCl$_3$, 23 °C

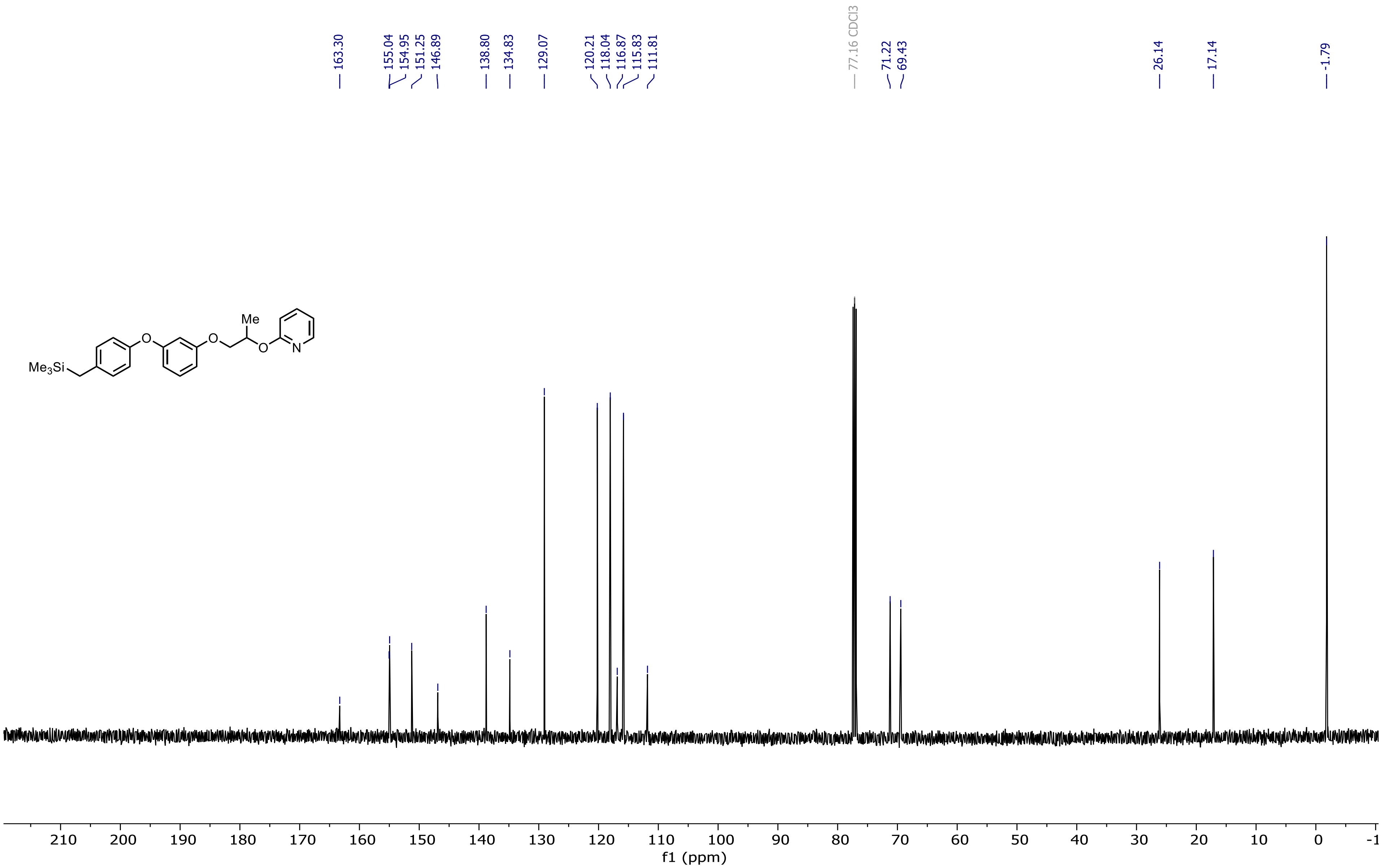

^{1}H NMR of methyl bis(pinacolato)diboron derivative 8

CDCl₃, 23 °C

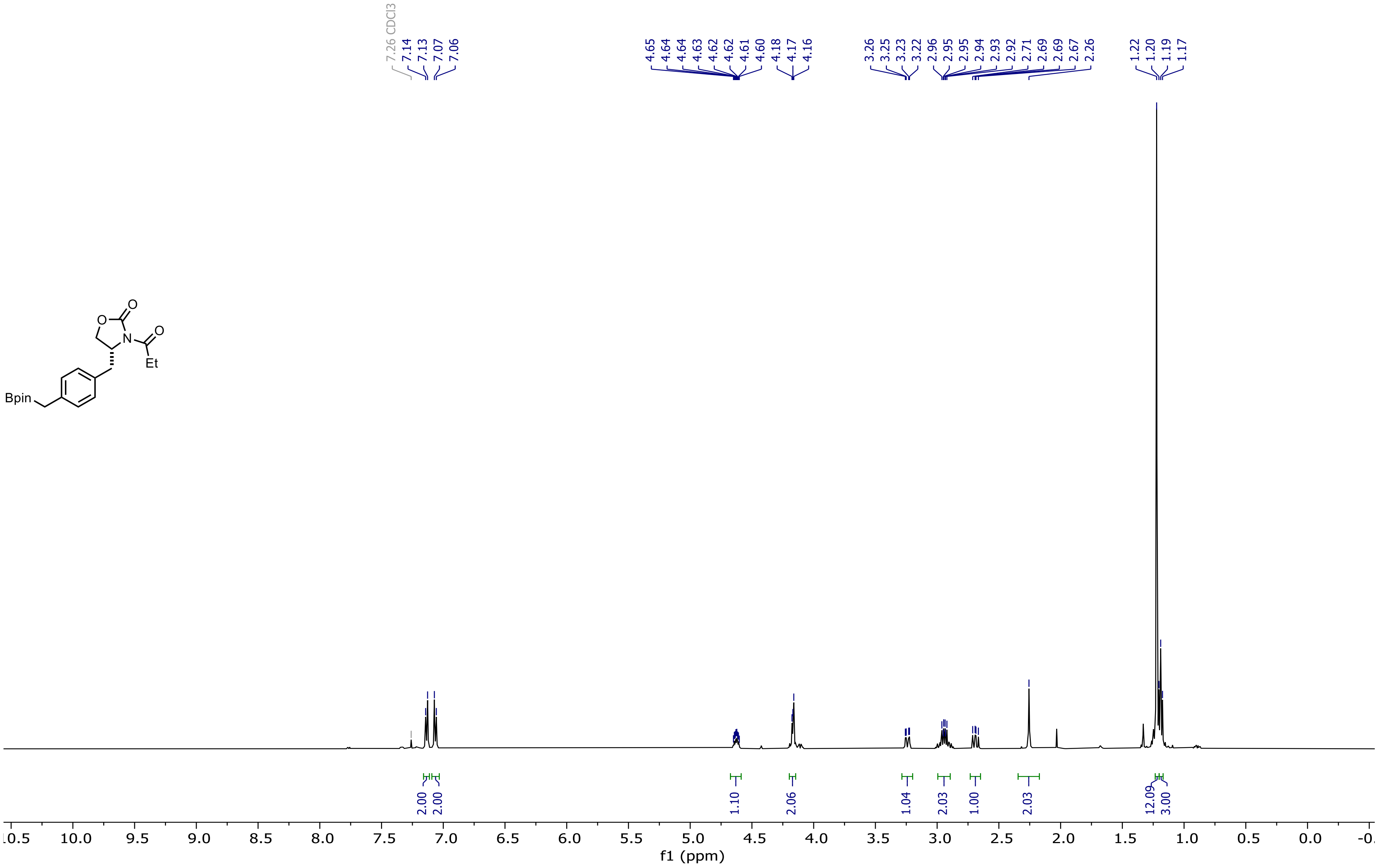

^{13}C NMR of methyl bis(pinacolato)diboron derivative 8

CDCl$_3$, 23 °C

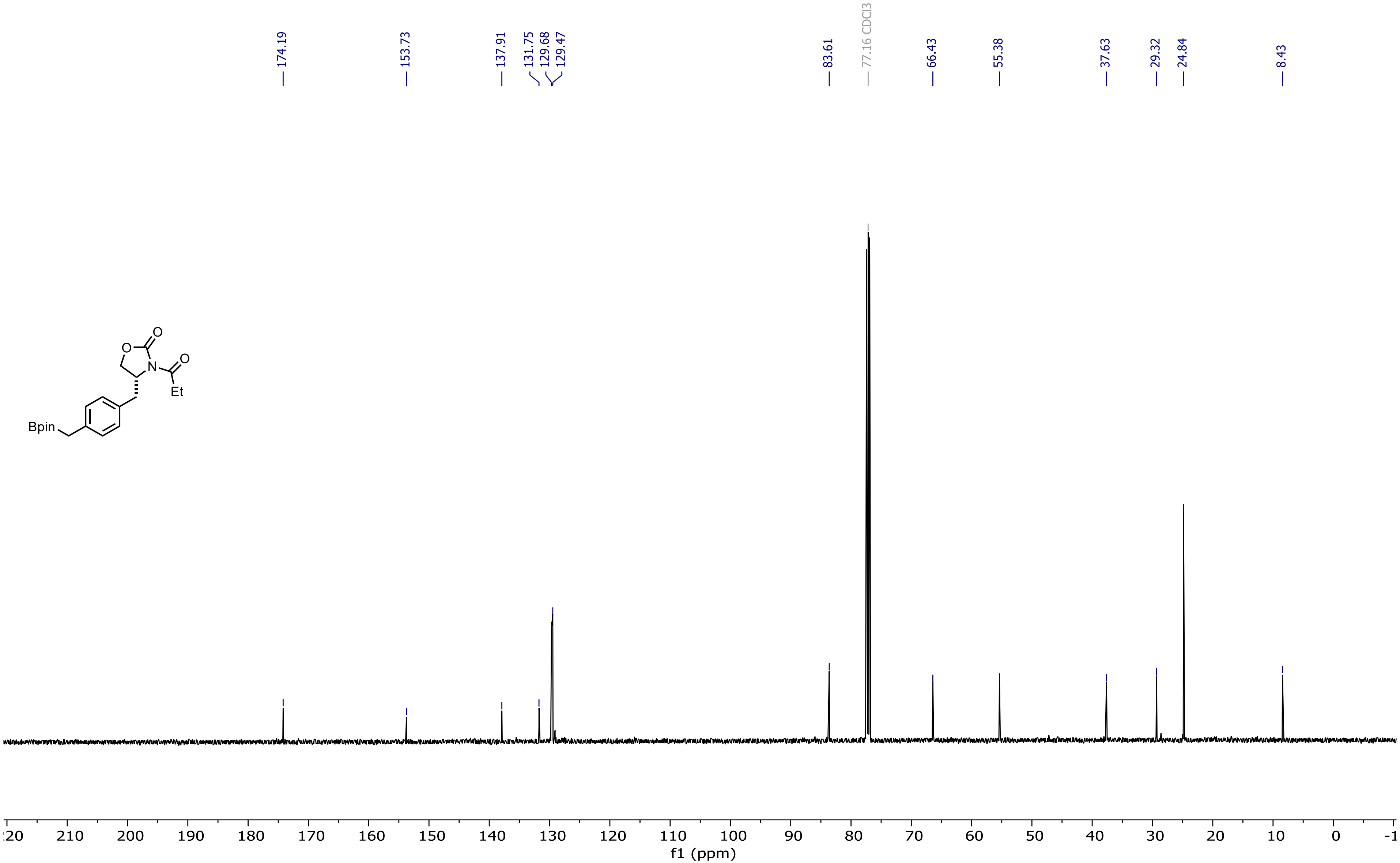

^{1}H NMR of alanine-*N*-boc-methyl ester pyriproxyfen derivative 9

CDCl3, 23 °C

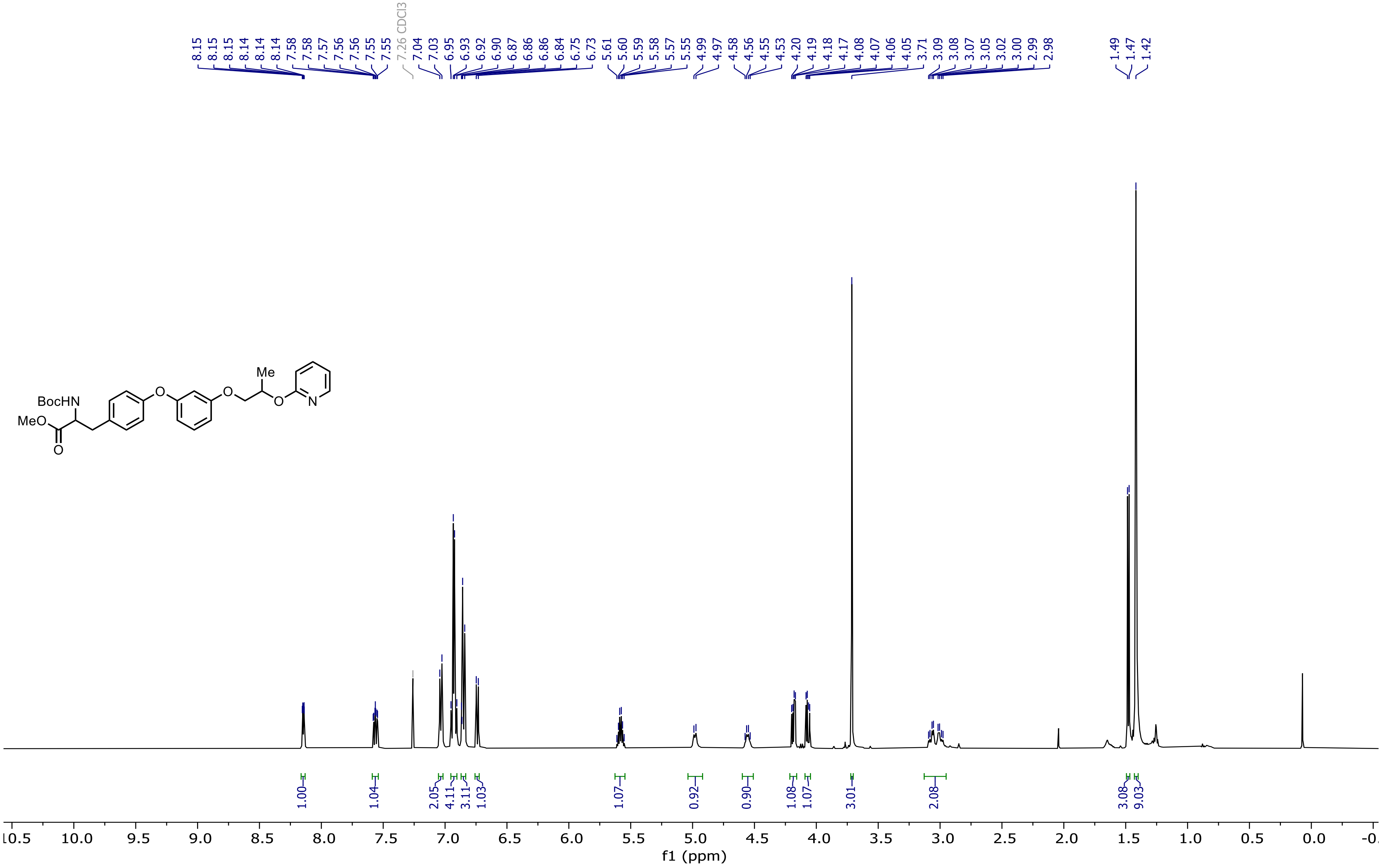

^{13}C NMR of alanine-*N*-boc-methyl ester pyriproxyfen derivative 9

CDCl$_3$, 23 °C

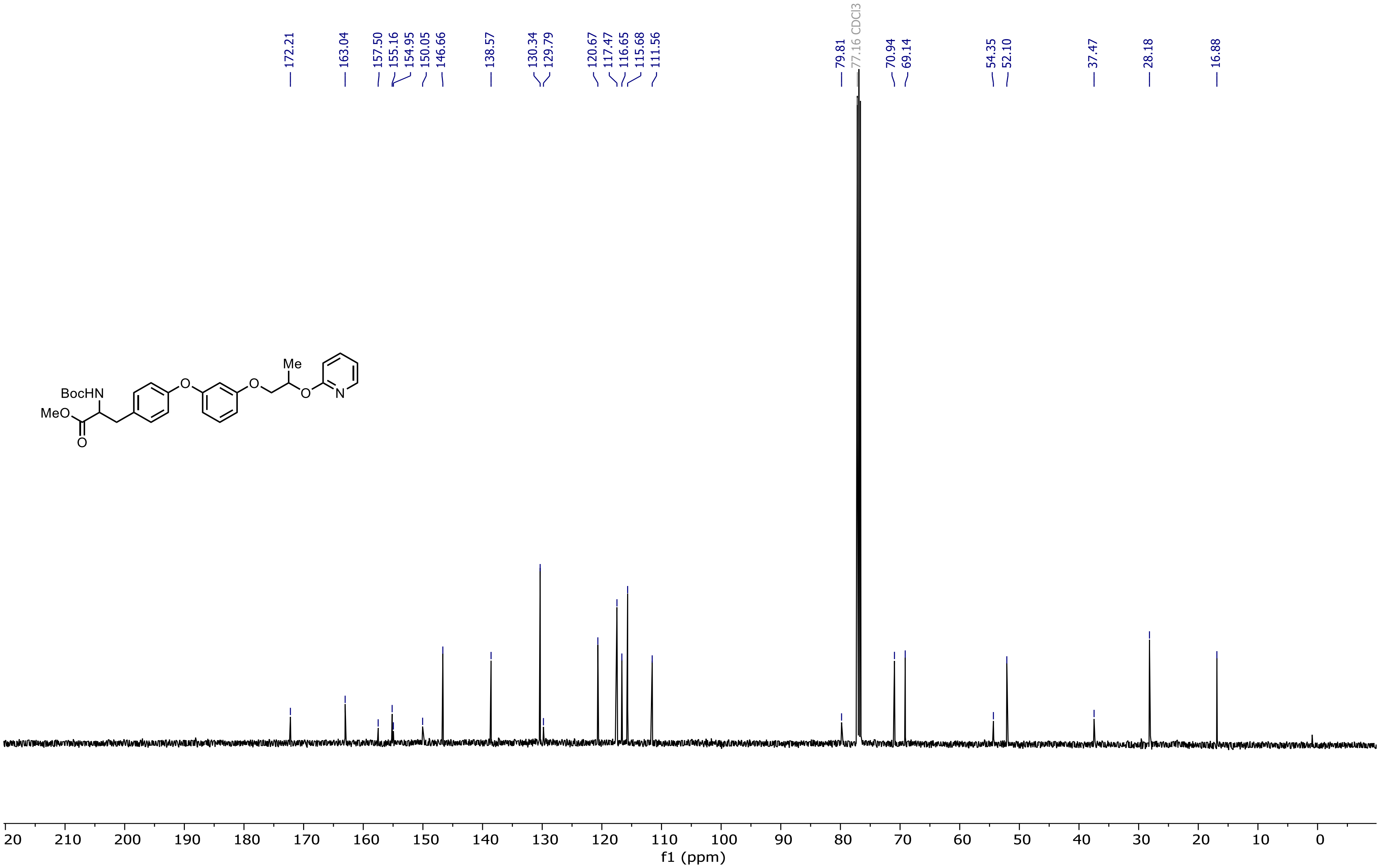

^{1}H NMR of oxetanyl pyriproxyfen derivative 10

CDCl$_3$, 23 °C

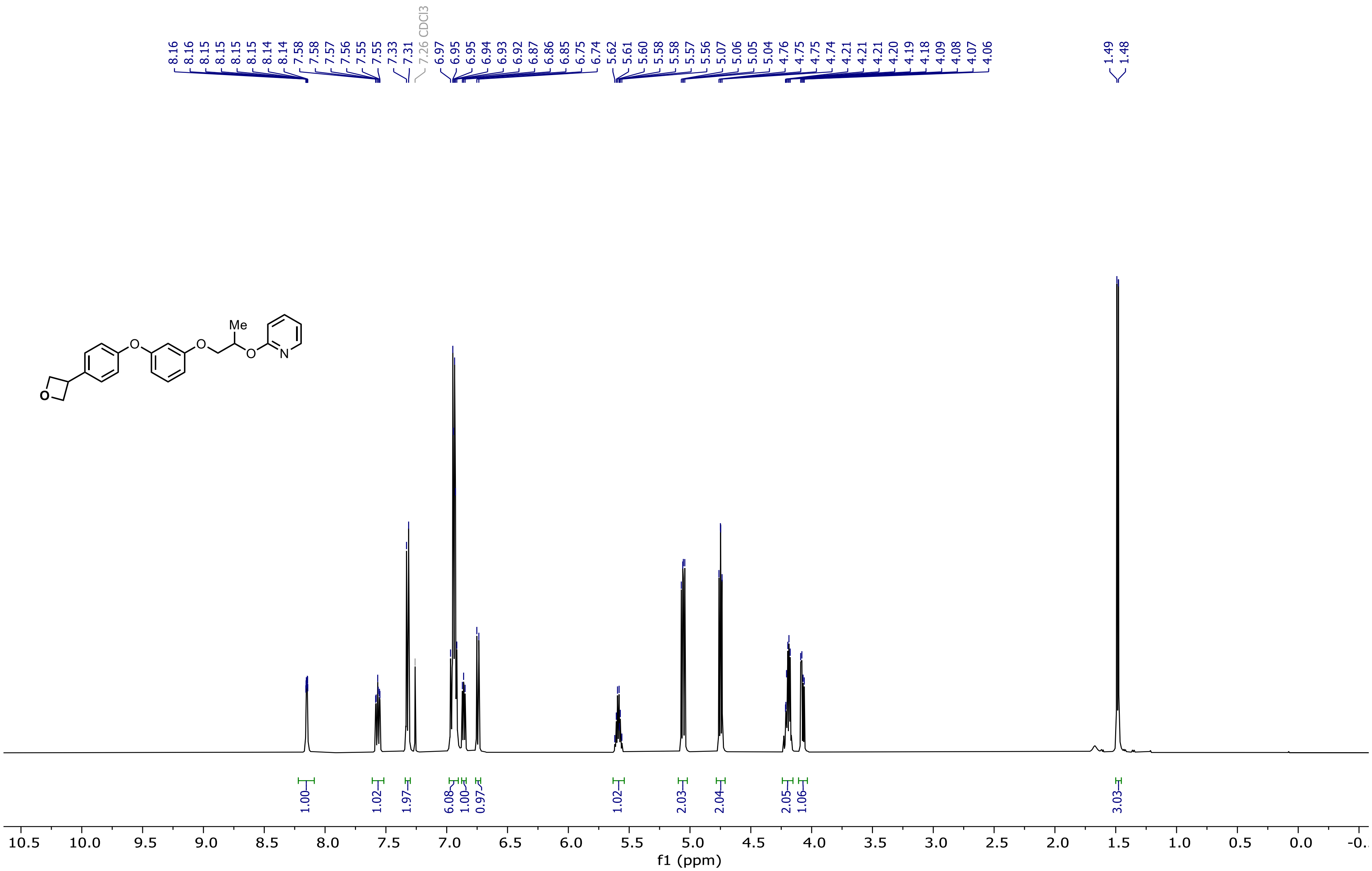

^{13}C NMR of oxetanyl pyriproxyfen derivative 10

CDCl$_3$, 23 °C

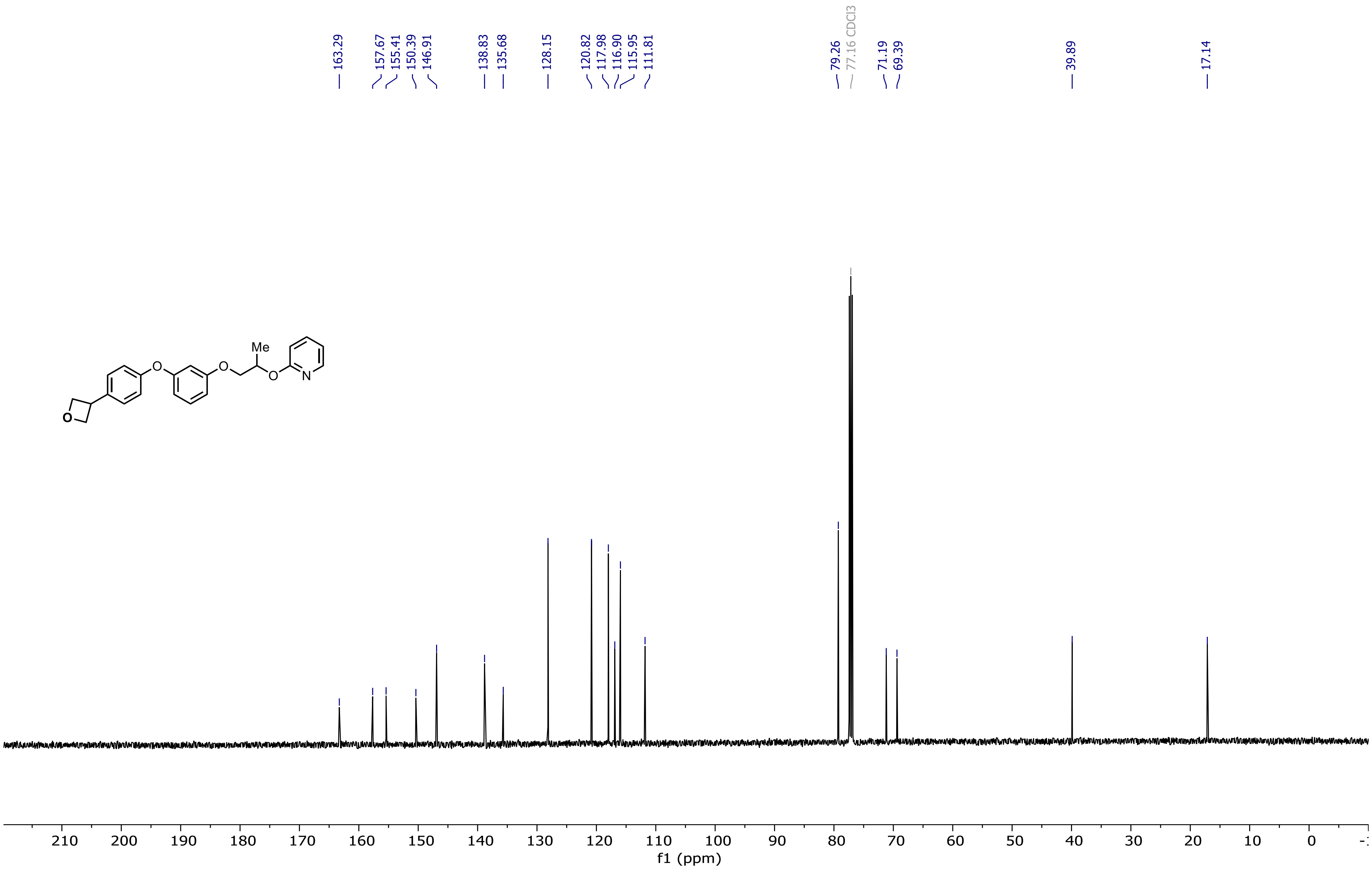

^{1}H NMR of boc-azetidinyl salicin pentaacetate derivative 11

CDCl$_3$, 23 °C

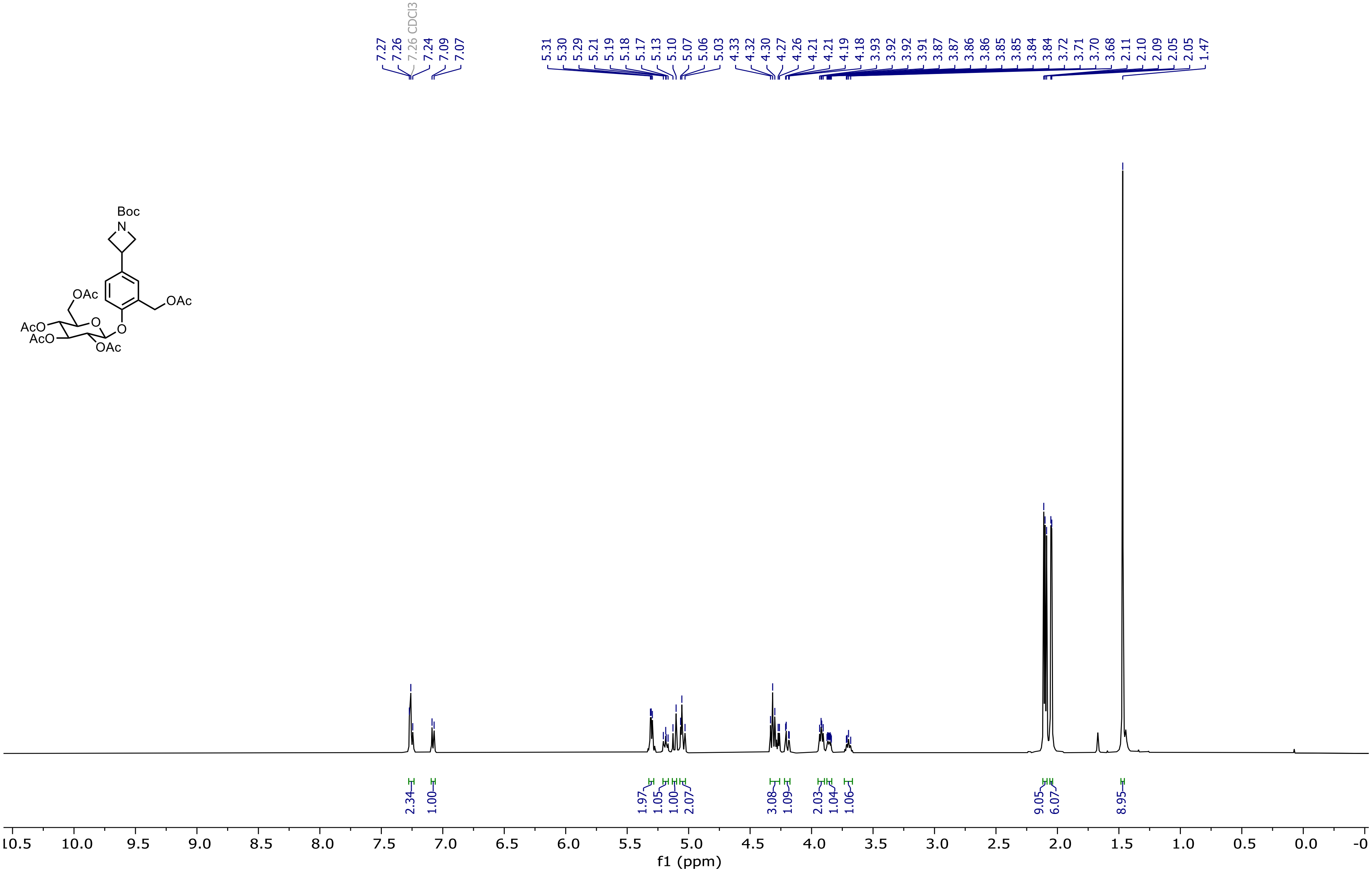

^{13}C NMR of boc-azetidinyl salicin pentaacetate derivative 11

CDCl$_3$, 23 °C

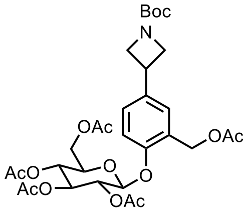

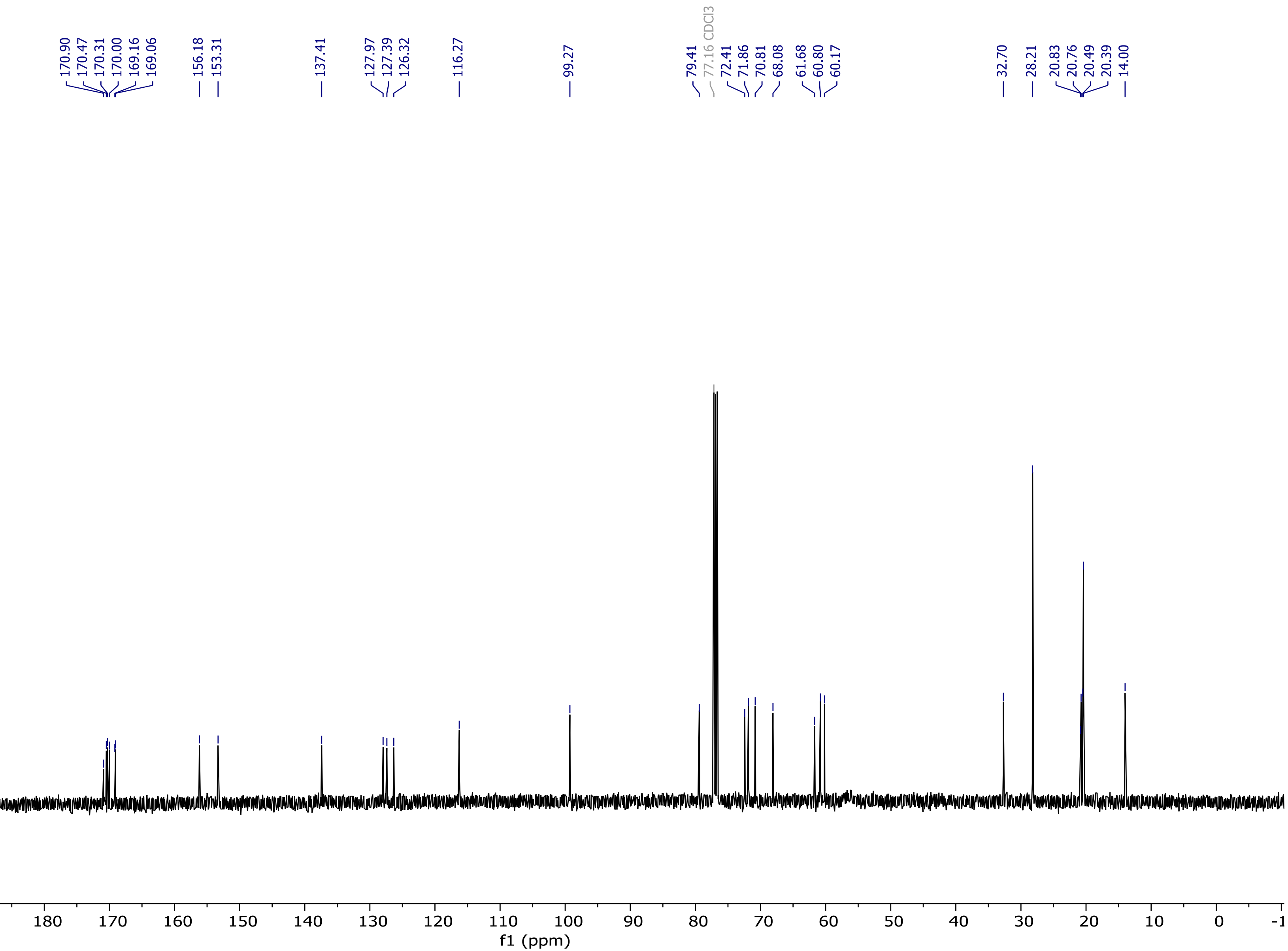

^{1}H NMR of boc-azetidinyl strychnine derivative 12

$CDCl_3$, 23 ºC

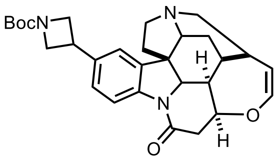

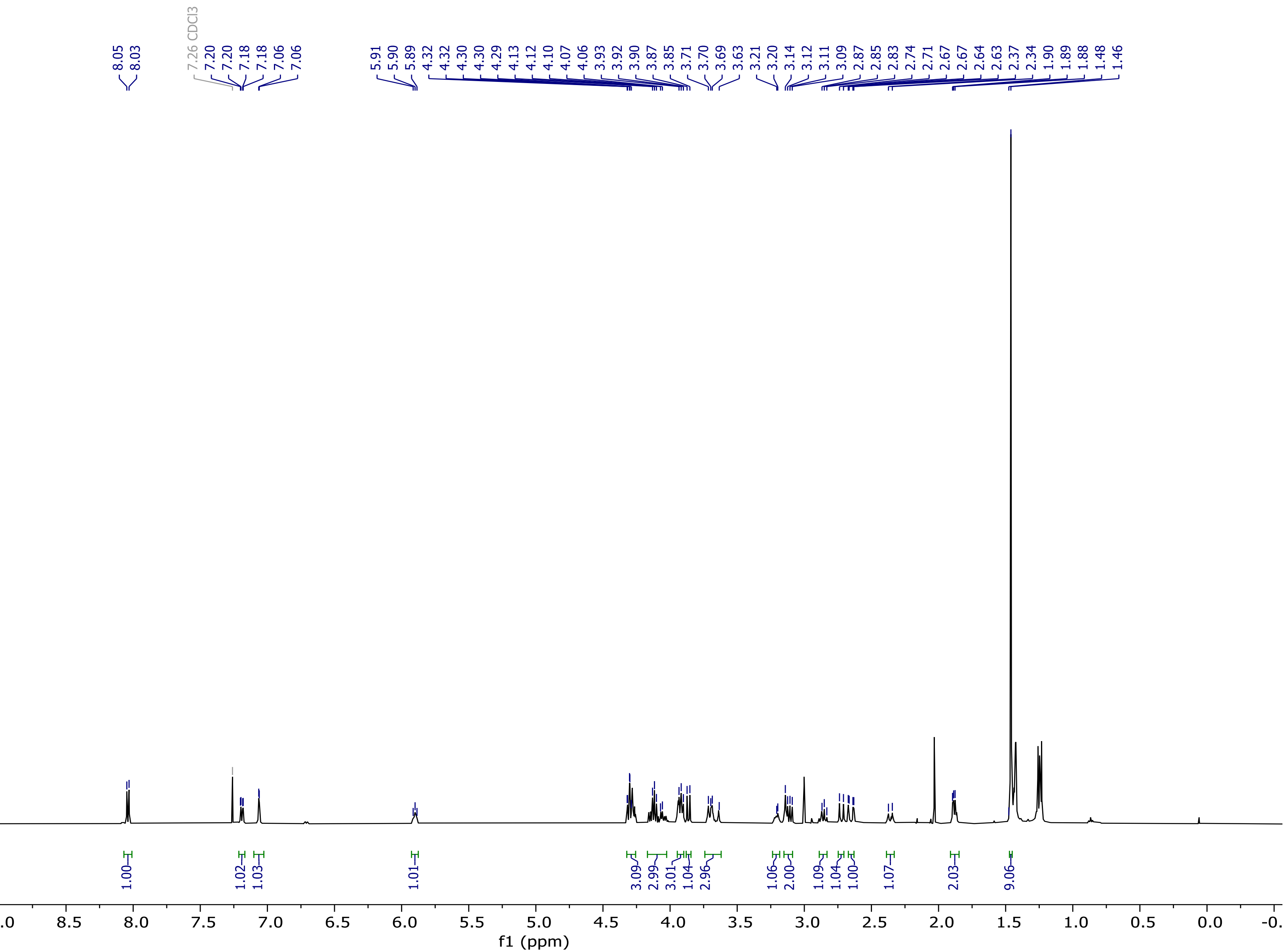

^{13}C NMR of boc-azetidinyl strychnine derivative 12

CDCl$_3$, 23 ºC

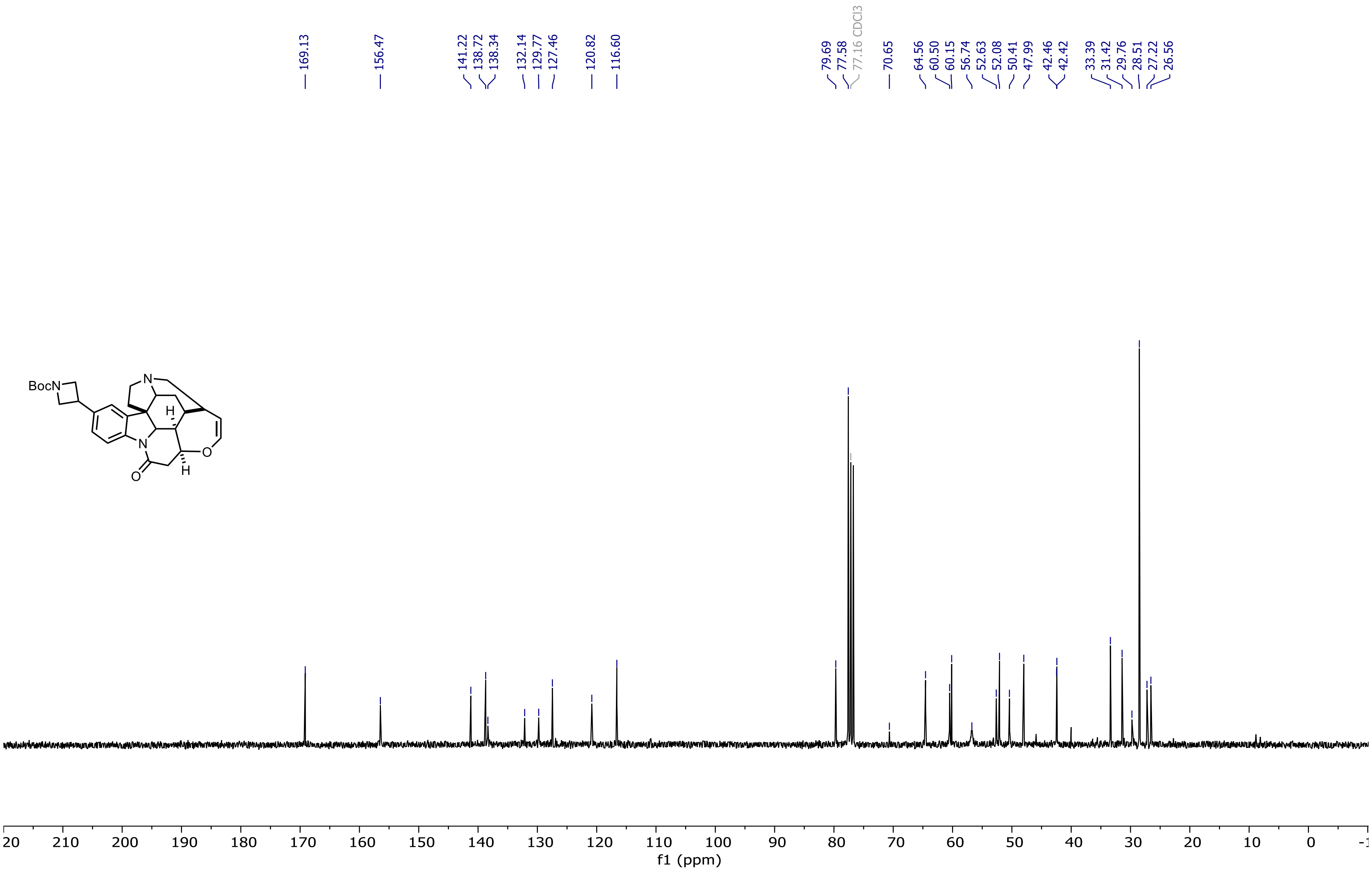

^{1}H NMR of methylene cyclobutane carboxylate pyriproxyfen derivative 13

CDCl$_3$, 23 °C

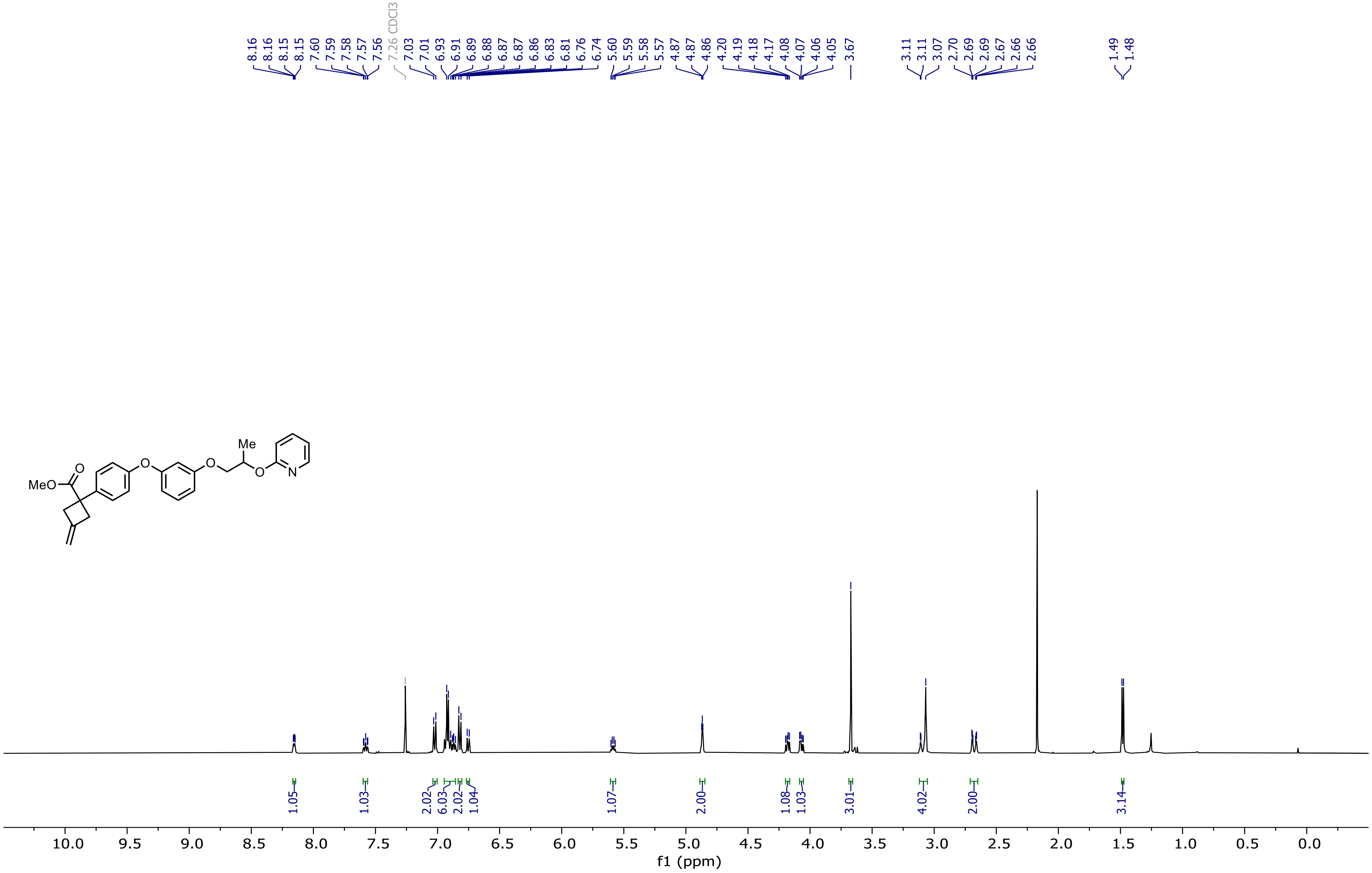

^{13}C NMR of methylene cyclobutane carboxylate pyriproxyfen derivative 13

CDCl$_3$, 23 °C

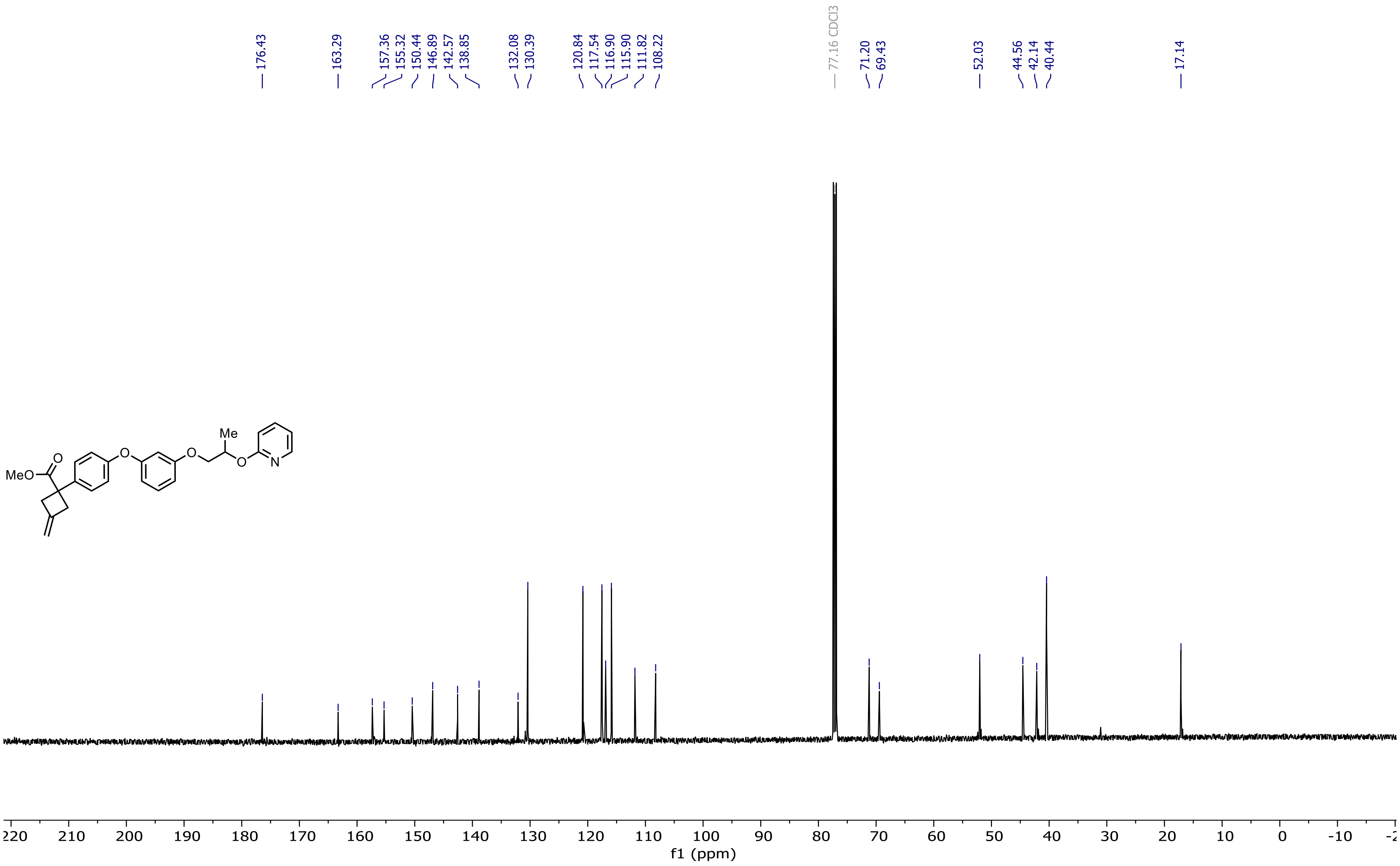

HSQC of methylene cyclobutane carboxylate pyriproxyfen derivative 13

$CDCl_3$, 23 °C

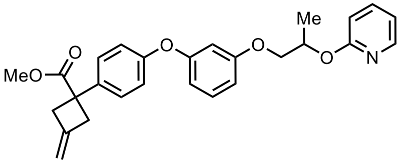

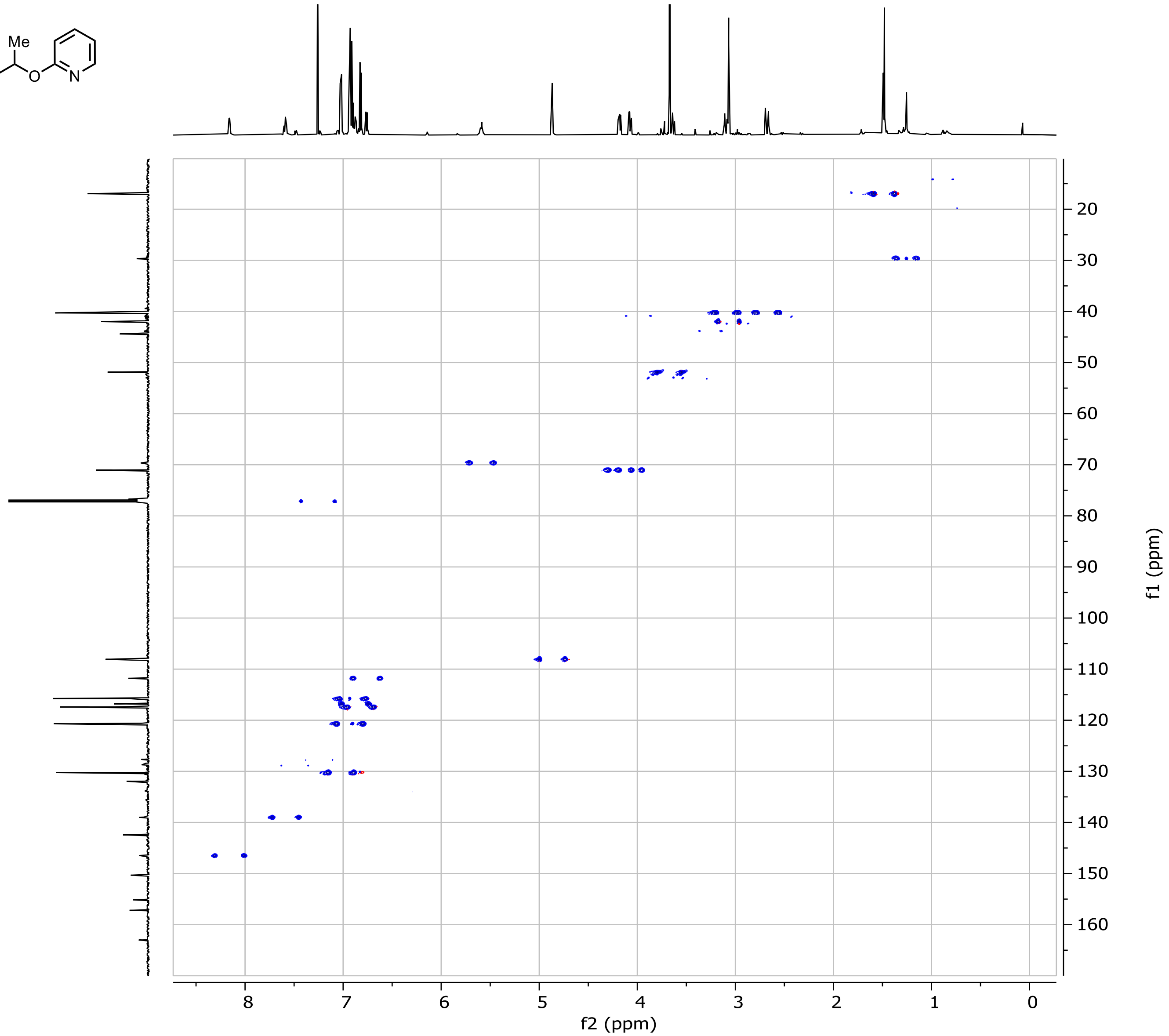

HMBC of methylene cyclobutane carboxylate pyriproxyfen derivative 13

CDCl3, 23 °C

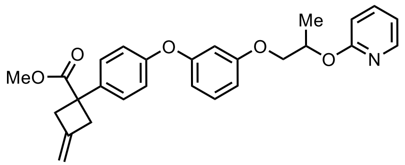

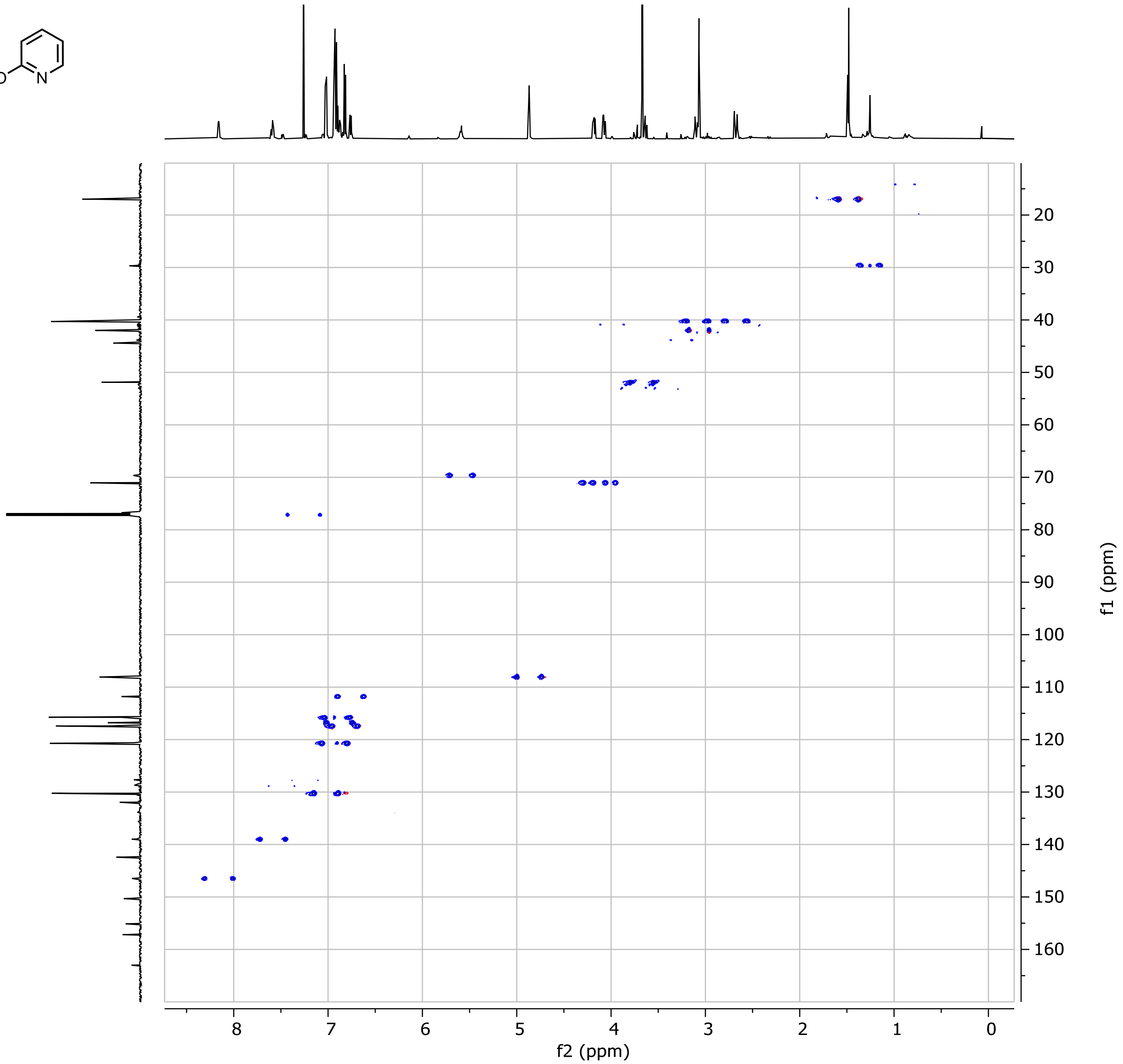

COSY of methylene cyclobutane carboxylate pyriproxyfen derivative 13

$CDCl_3$, 23 °C

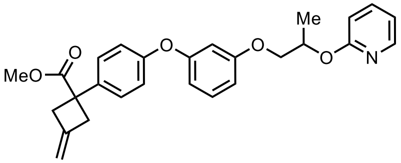

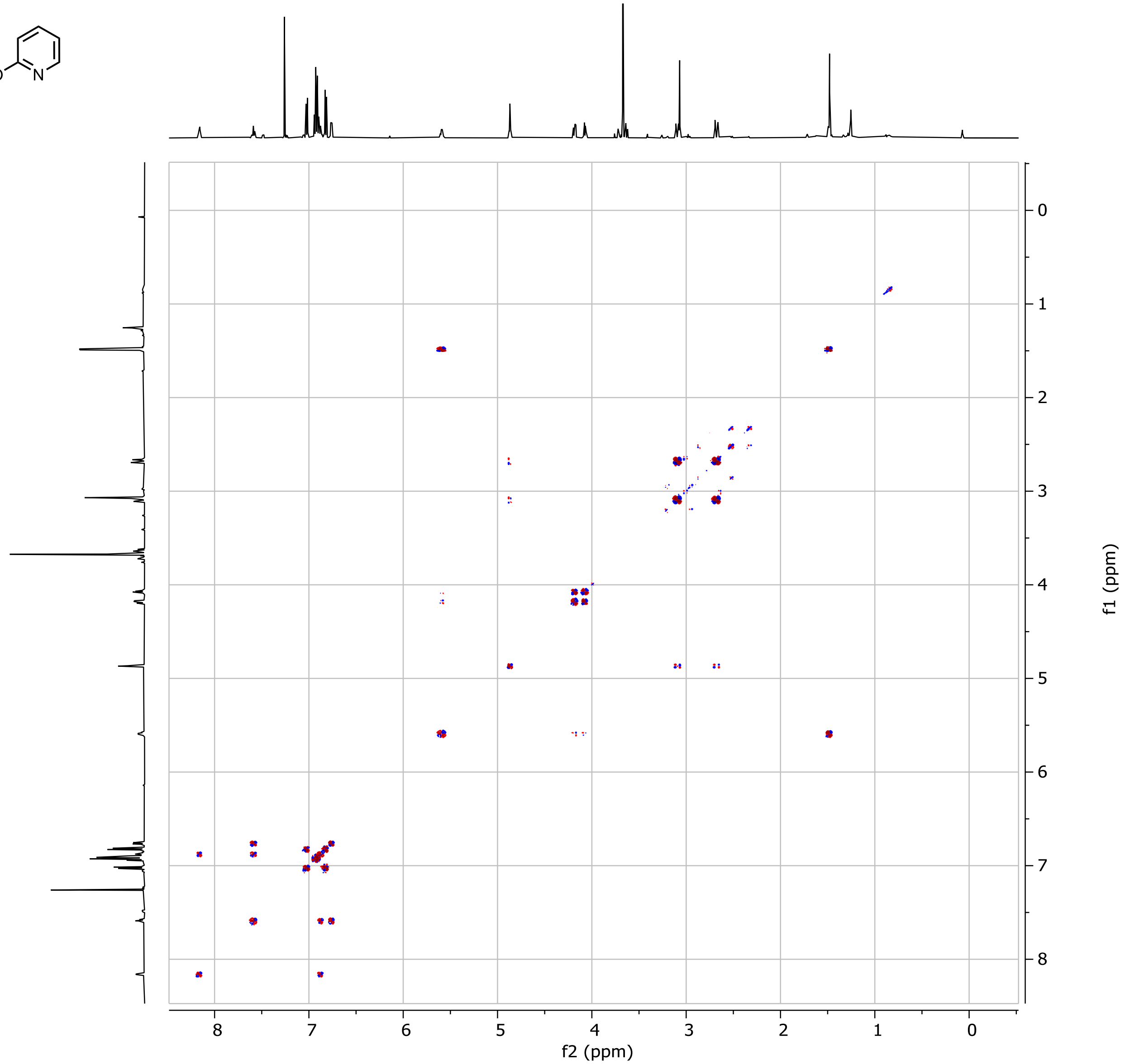

^{1}H NMR of oxaspiro[3.3]heptanyl pyriproxyfen derivative 14

CDCl$_3$, 23 °C

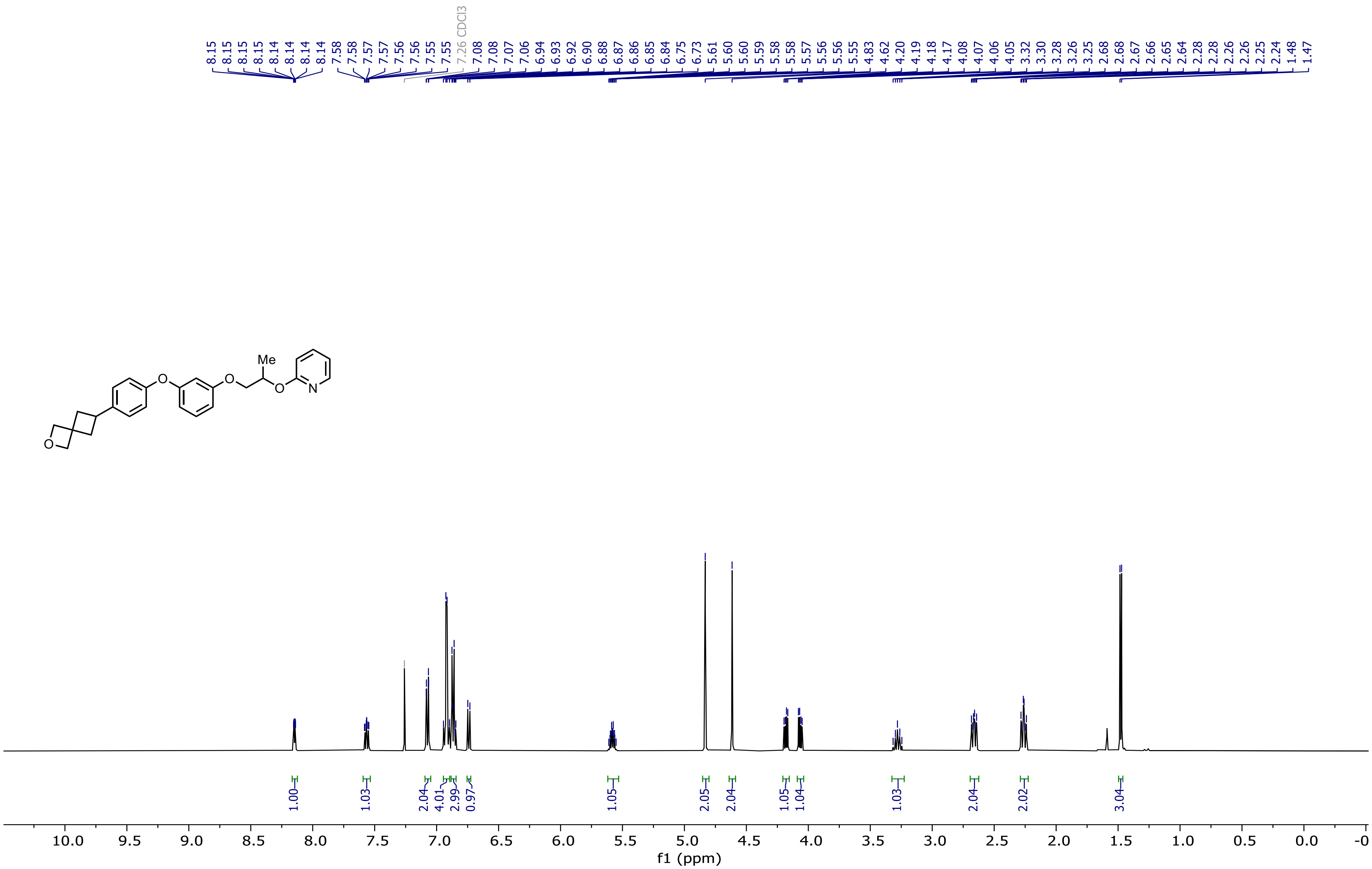

^{13}C NMR of oxaspiro[3.3]heptanyl pyriproxyfen derivative 14

CDCl$_3$, 23 °C

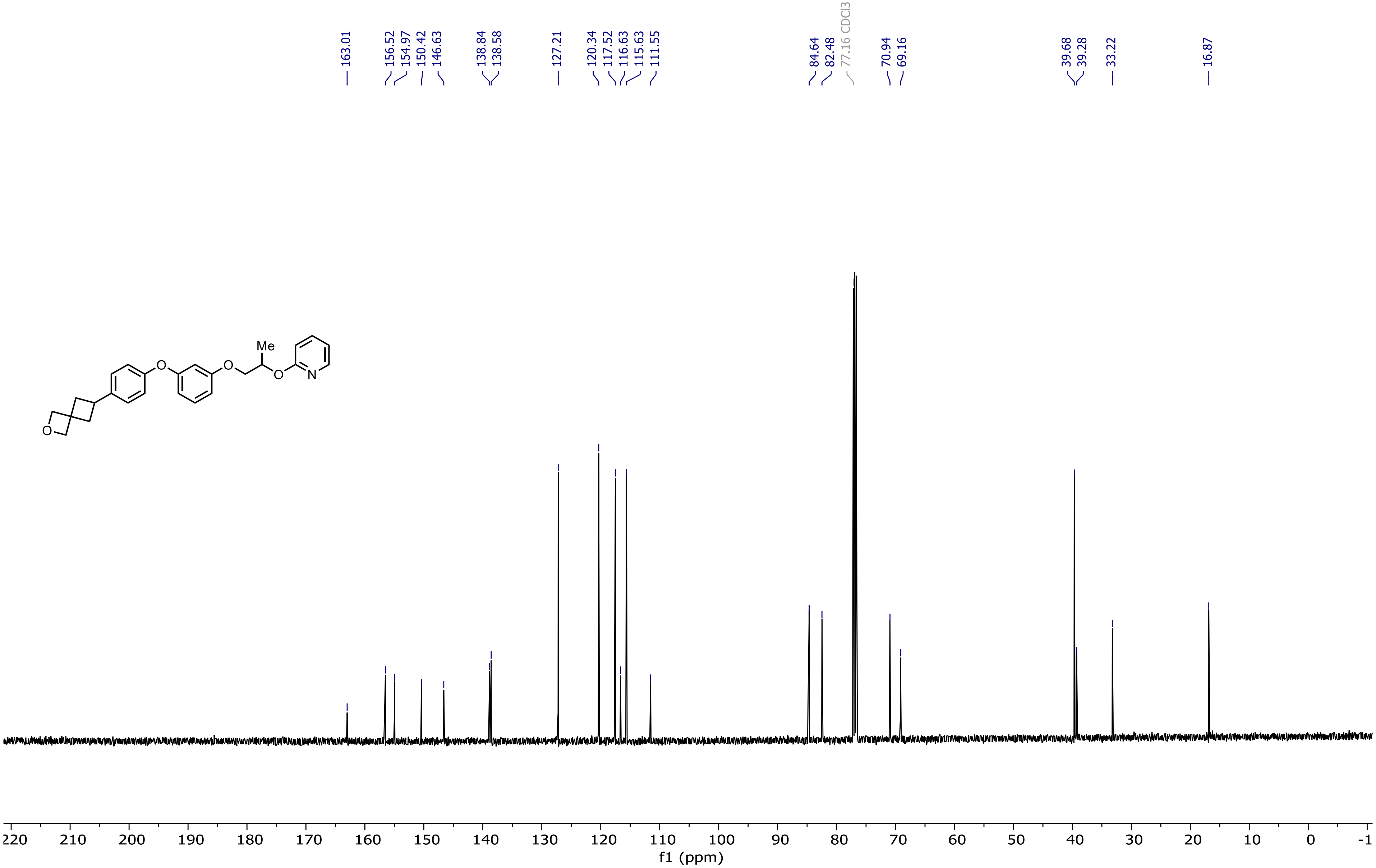

^{1}H NMR of methyl fenofibrate derivative 15

$CDCl_3$, 23 ºC

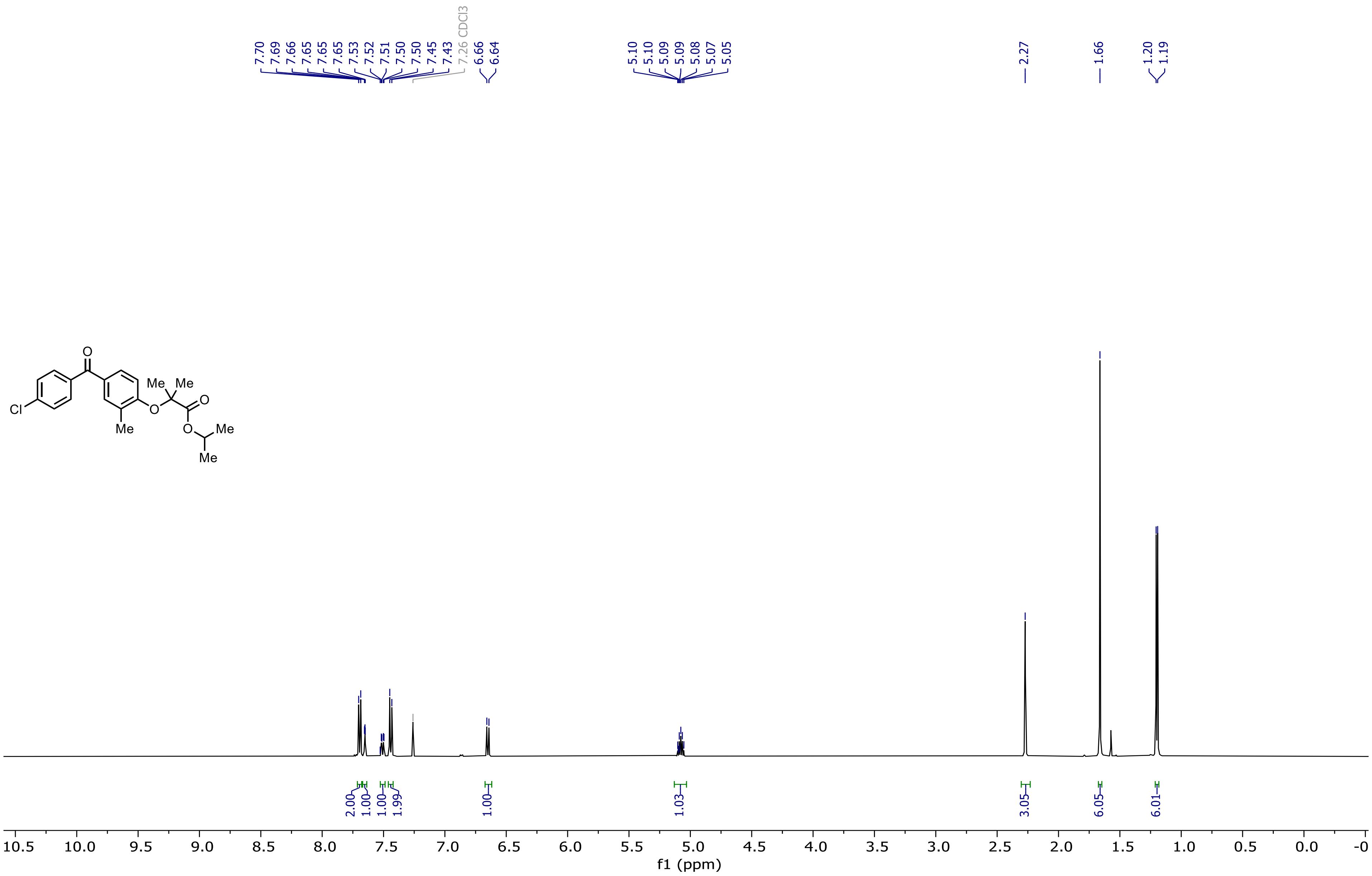

^{13}C NMR of methyl fenofibrate derivative 15

CDCl$_3$, 23 ºC

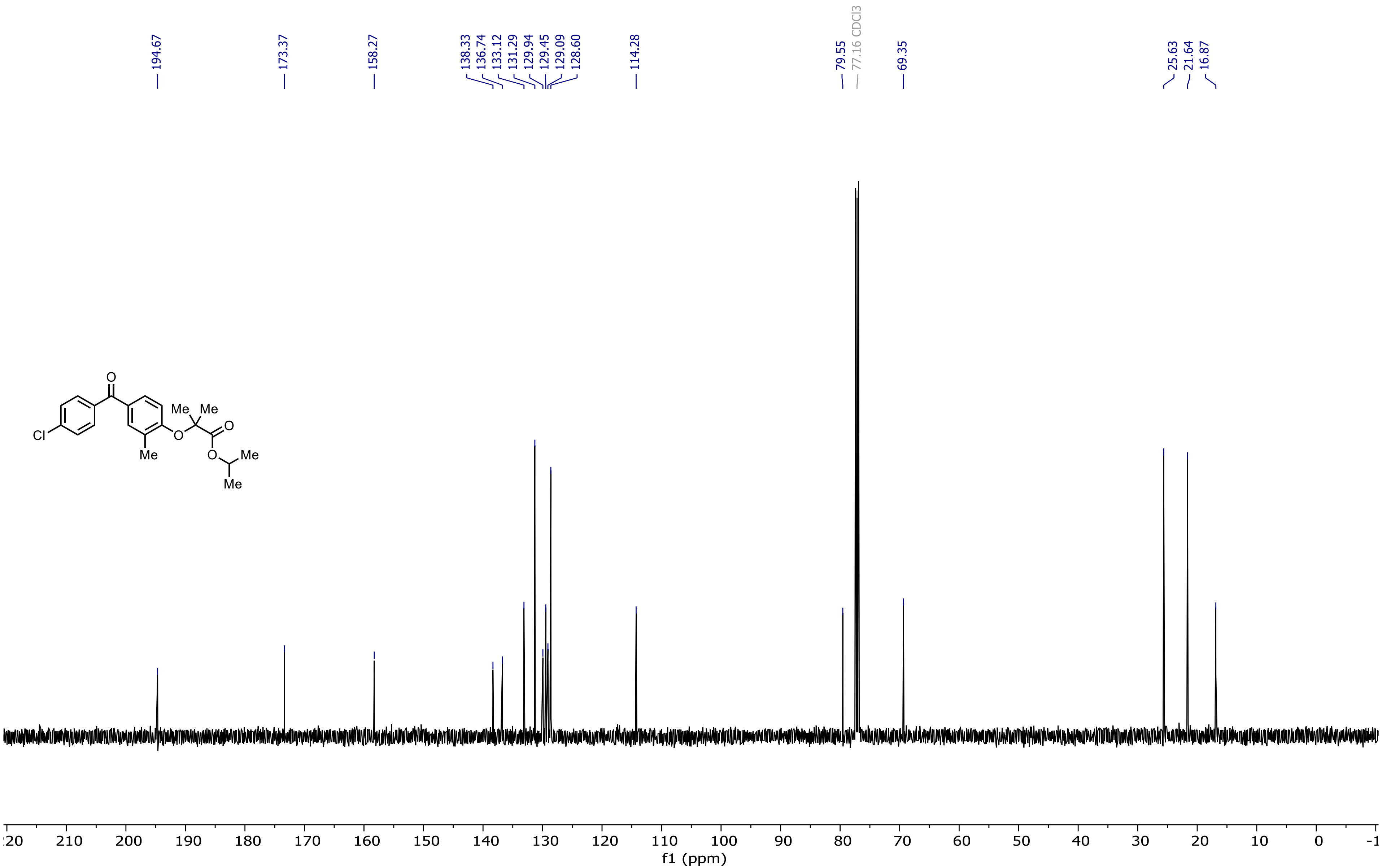

^{1}H NMR of 2-methylpropanyl indomethacin methyl ester derivative 16

CDCl$_3$, 23 °C

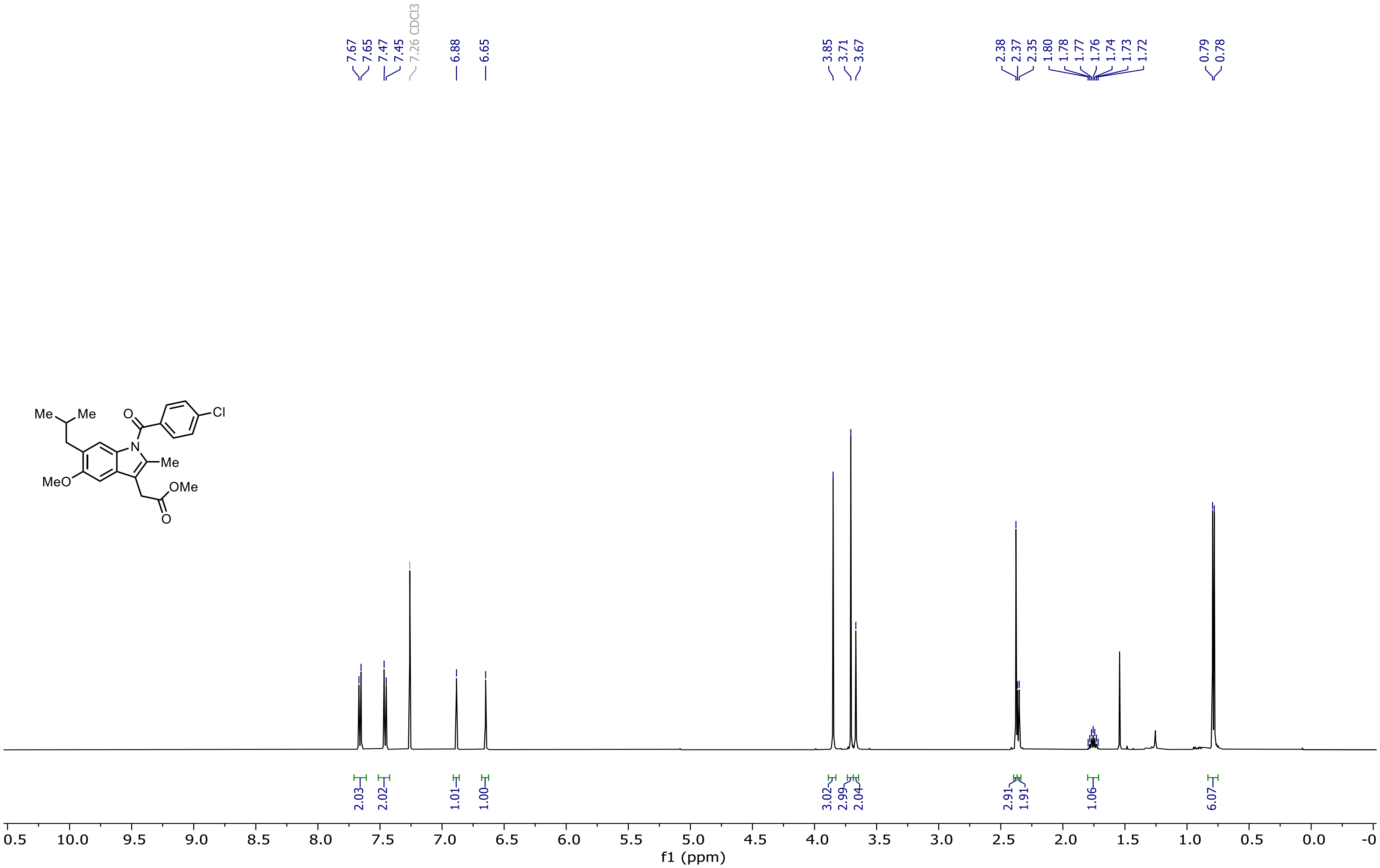

^{13}C NMR of 2-methylpropanyl indomethacin methyl ester derivative 16

$CDCl_3$, 23 °C

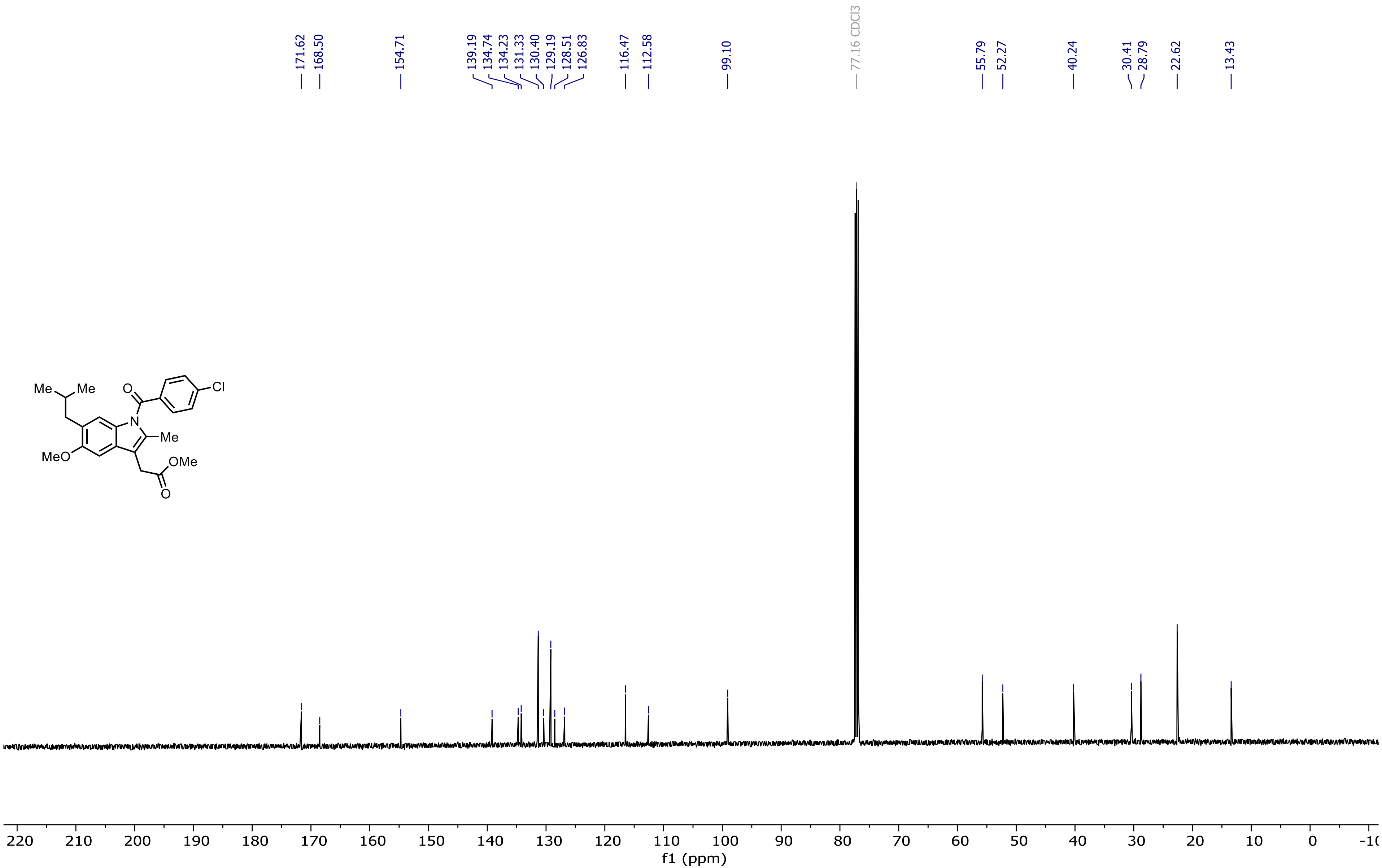

^{1}H NMR of boc-piperidinyl pyriproxyfen derivative 17

$CDCl_3$, 23 °C

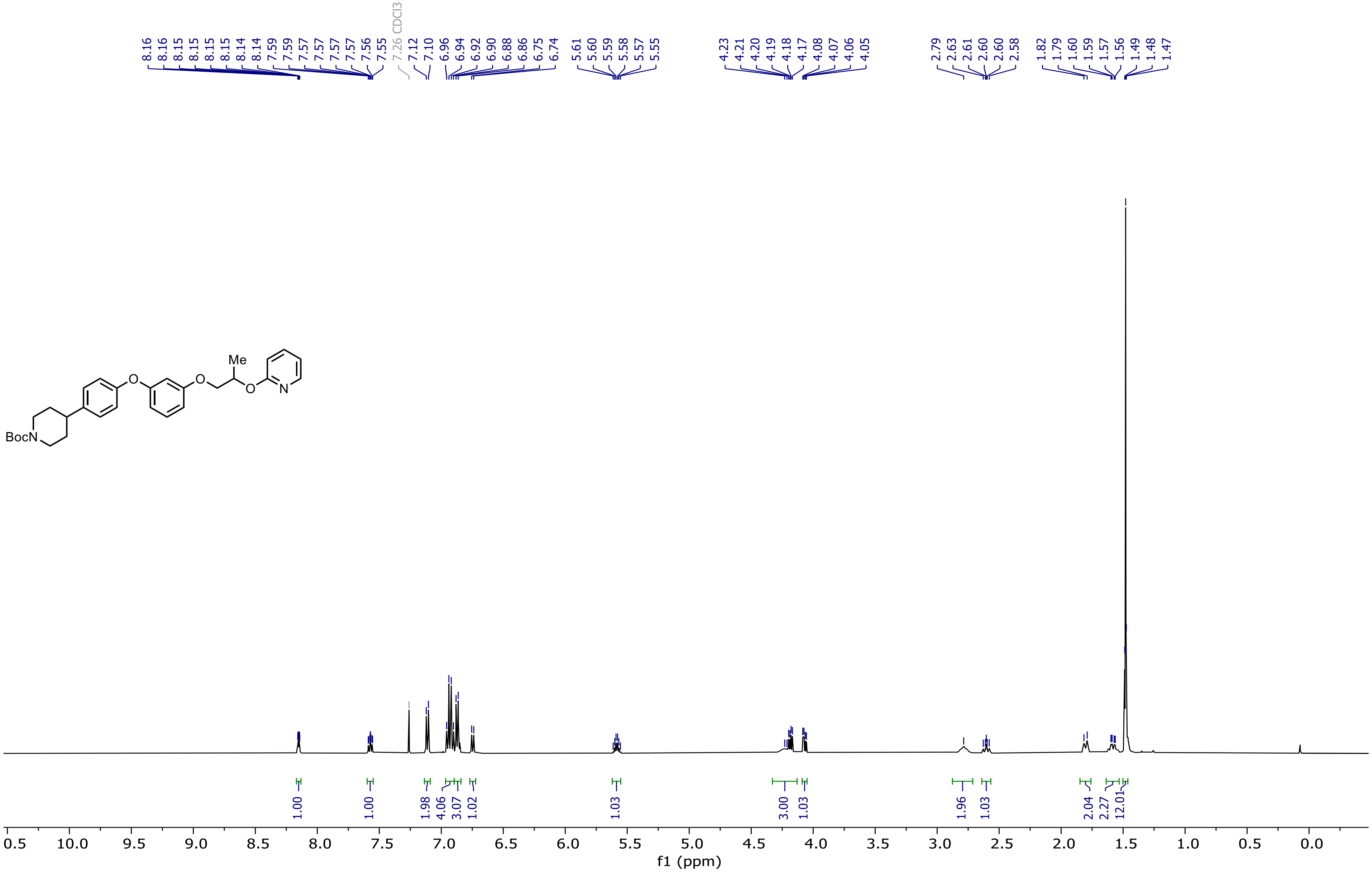

^{13}C NMR of boc-piperidinyl pyriproxyfen derivative 17

$CDCl_3$, 23 °C

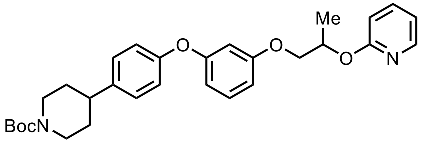

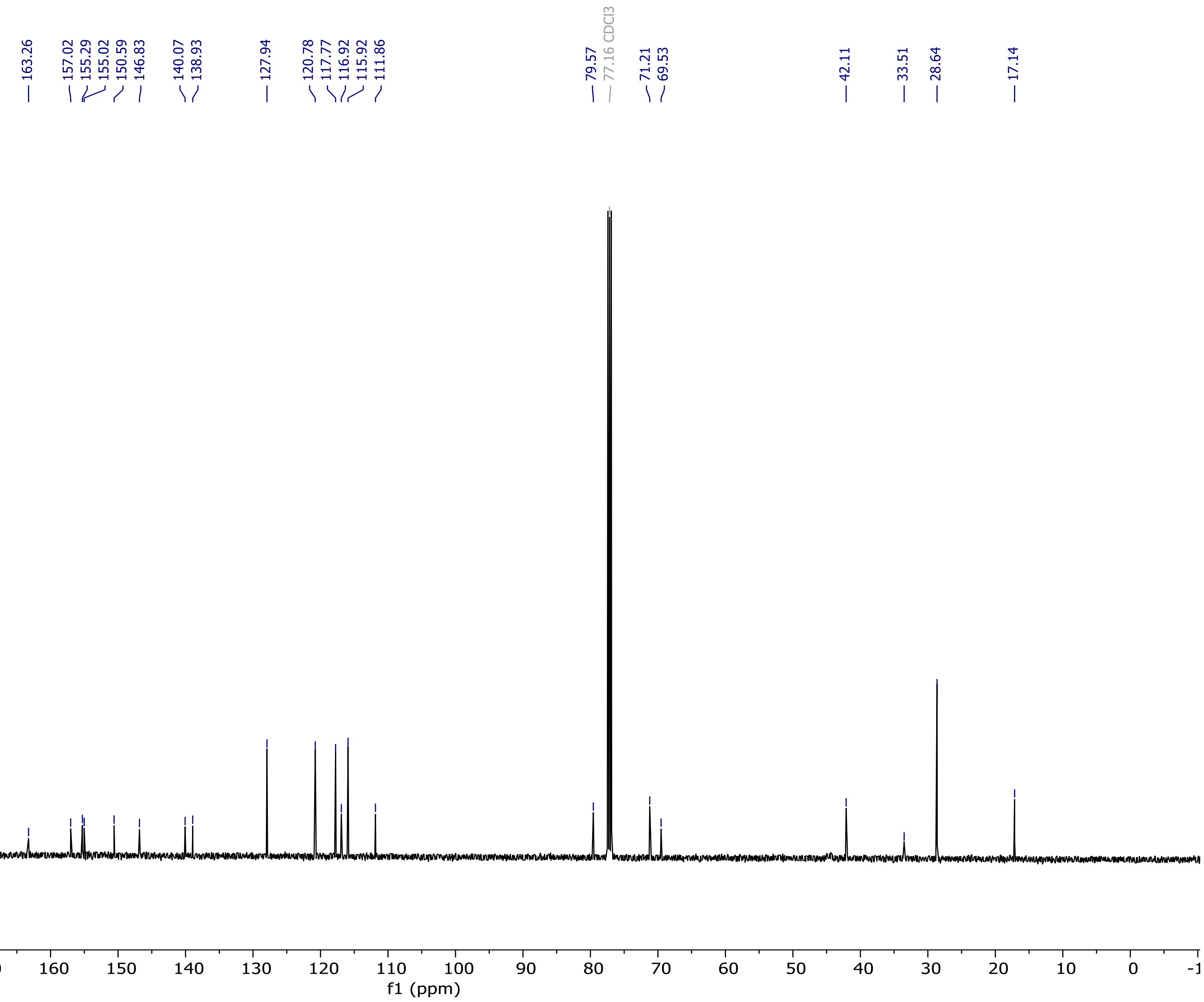

^{1}H NMR of Fmoc-Arg(Pbf)-OH pyriproxyfen derivative 18

CDCl$_3$, 23 °C

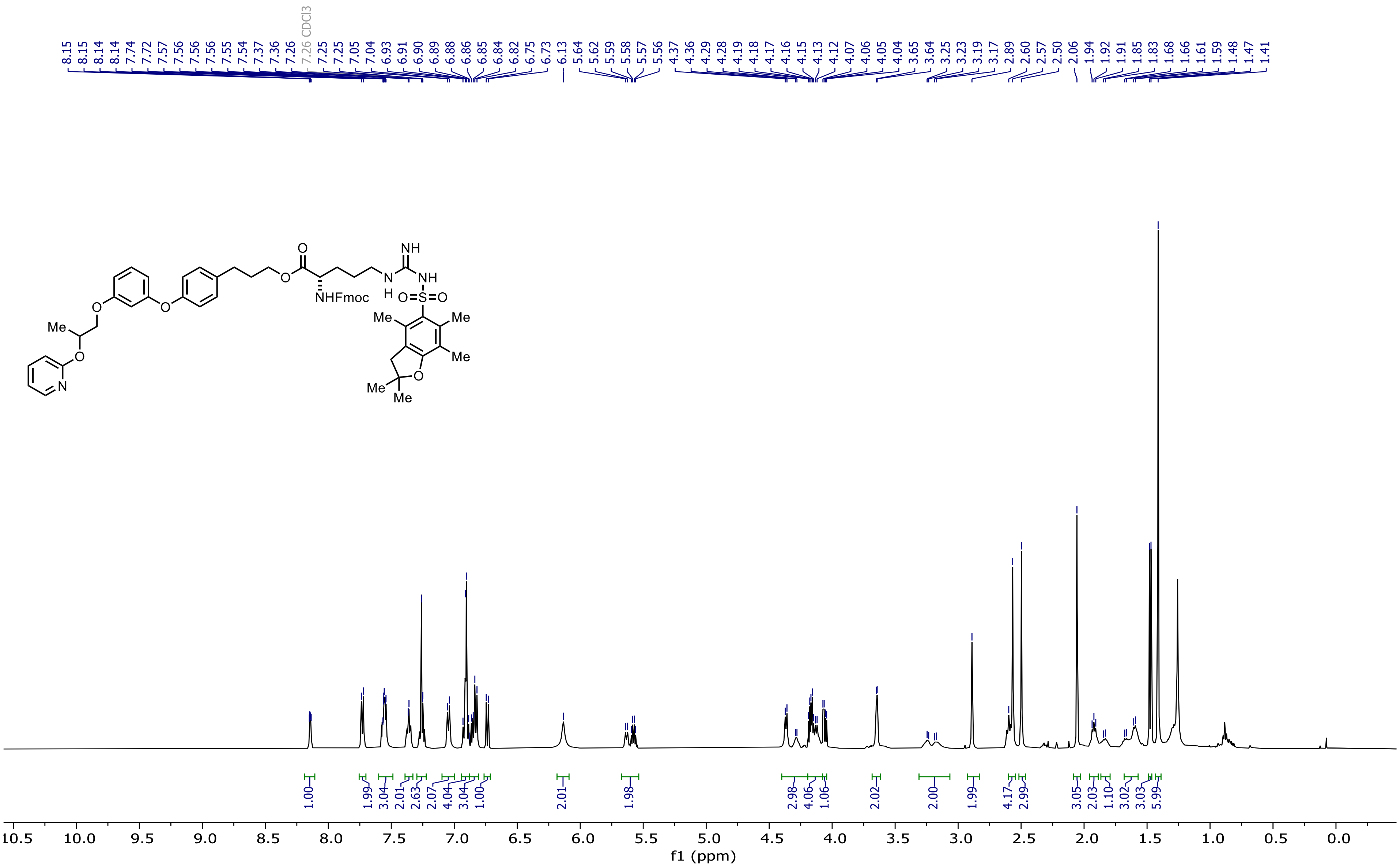

^{13}C NMR of Fmoc-Arg(Pbf)-OH pyriproxyfen derivative 18

CDCl$_3$, 23 °C

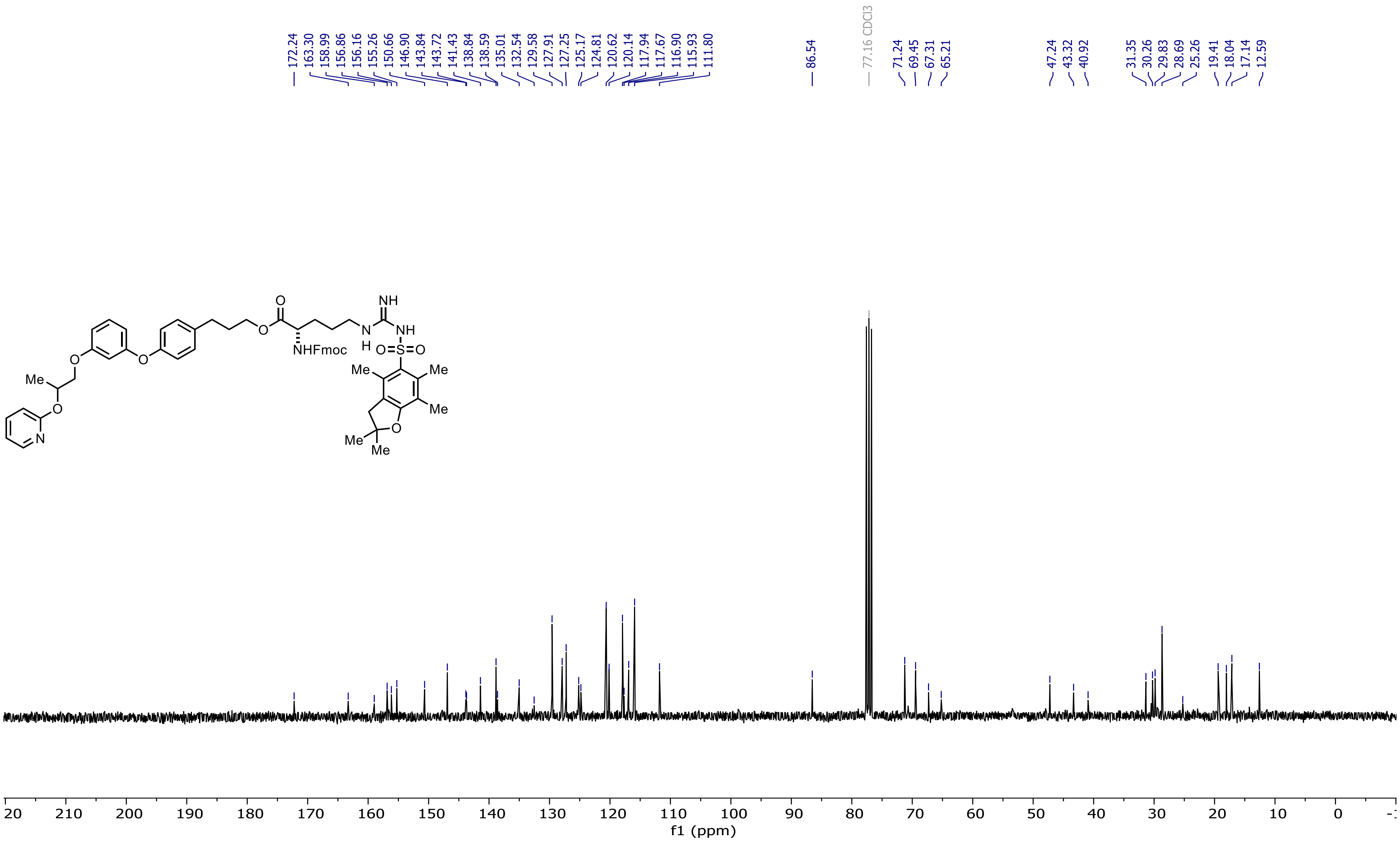

^{1}H NMR of nefiractam sulbactam derivative 19

CDCl₃, 23 °C

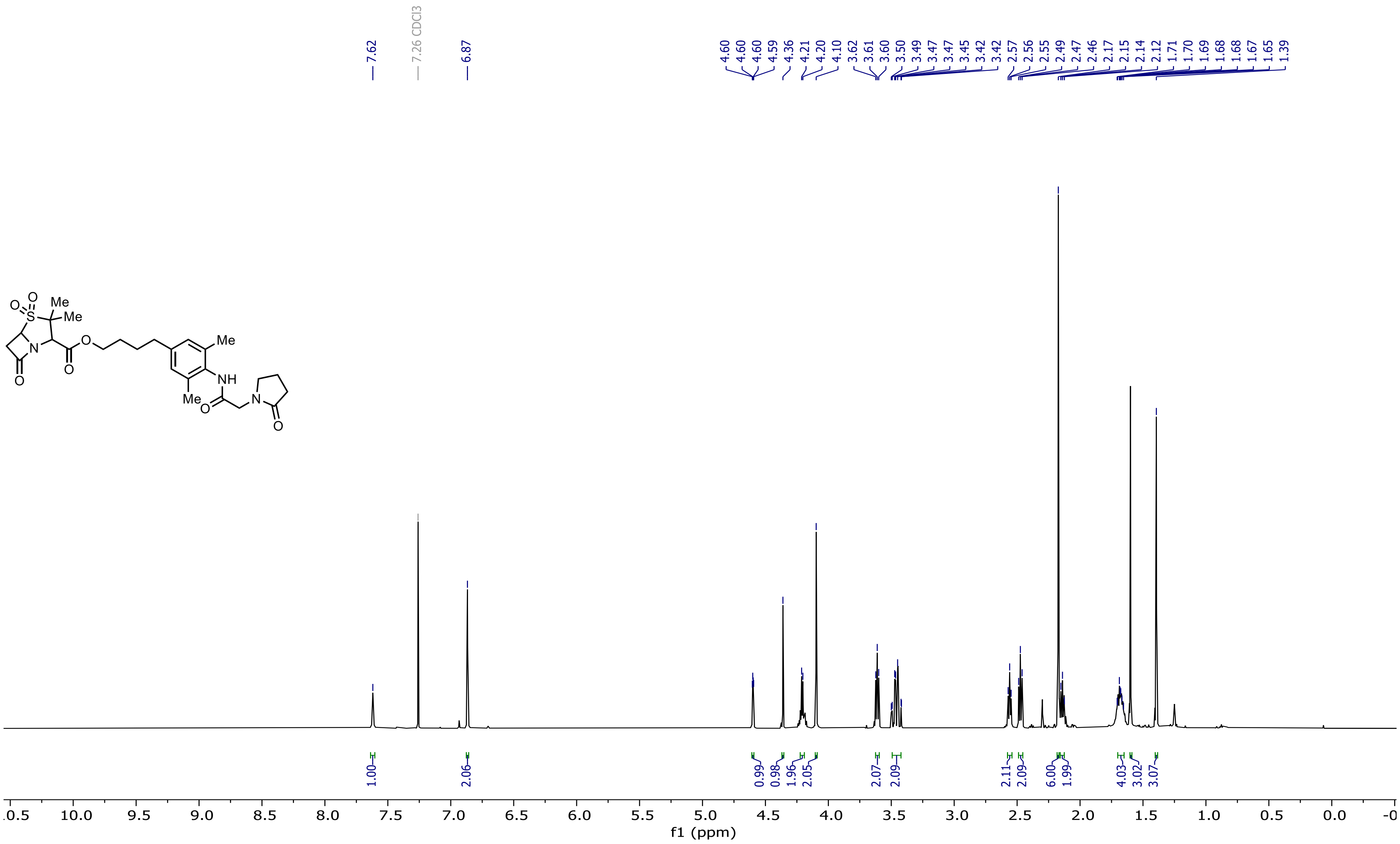

^{13}C NMR of nefiractam sulbactam derivative 19

CDCl3, 23 °C

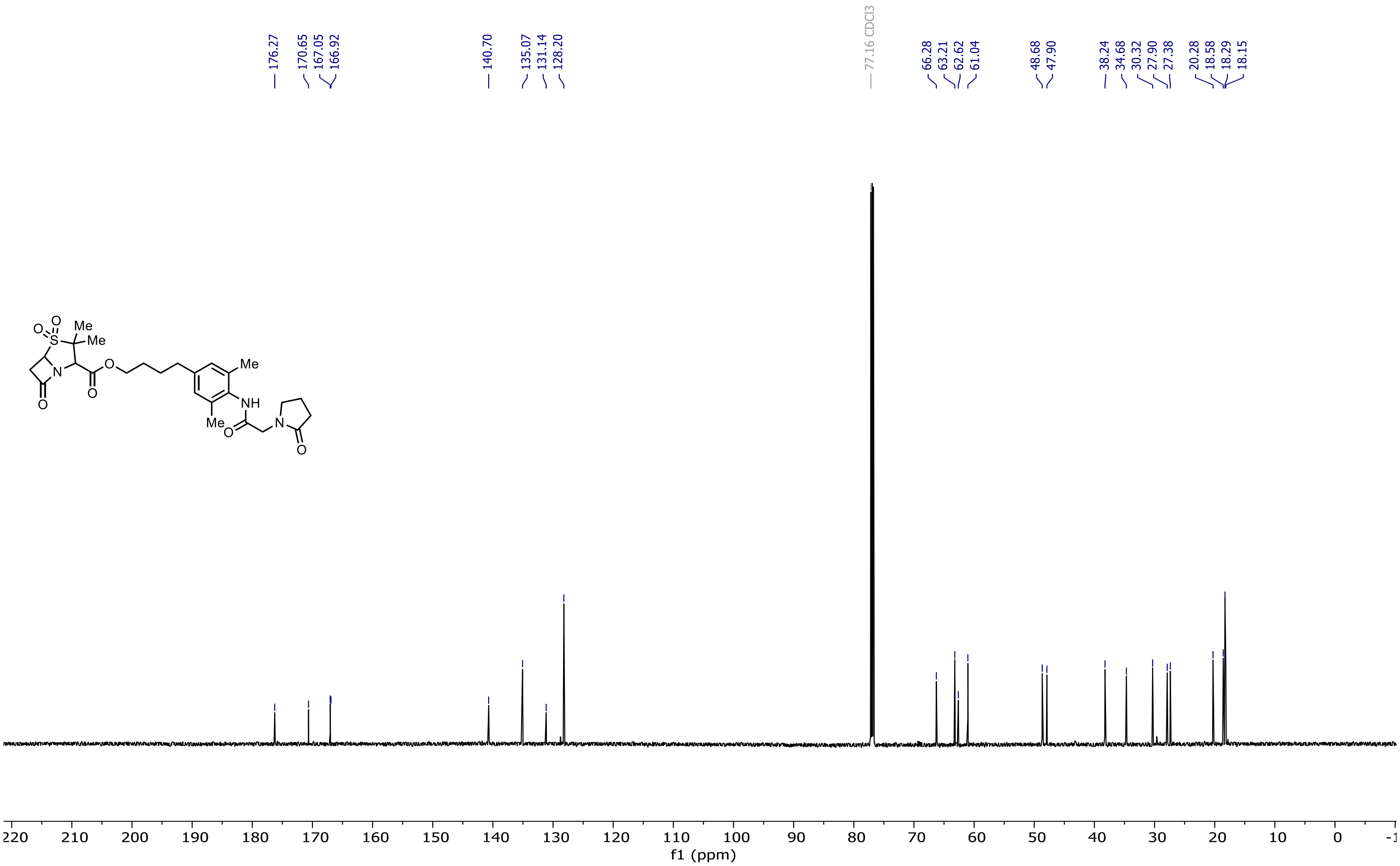

^{1}H NMR of sulbactam estrone methyl ether derivative 20

CDCl$_3$, 23 °C

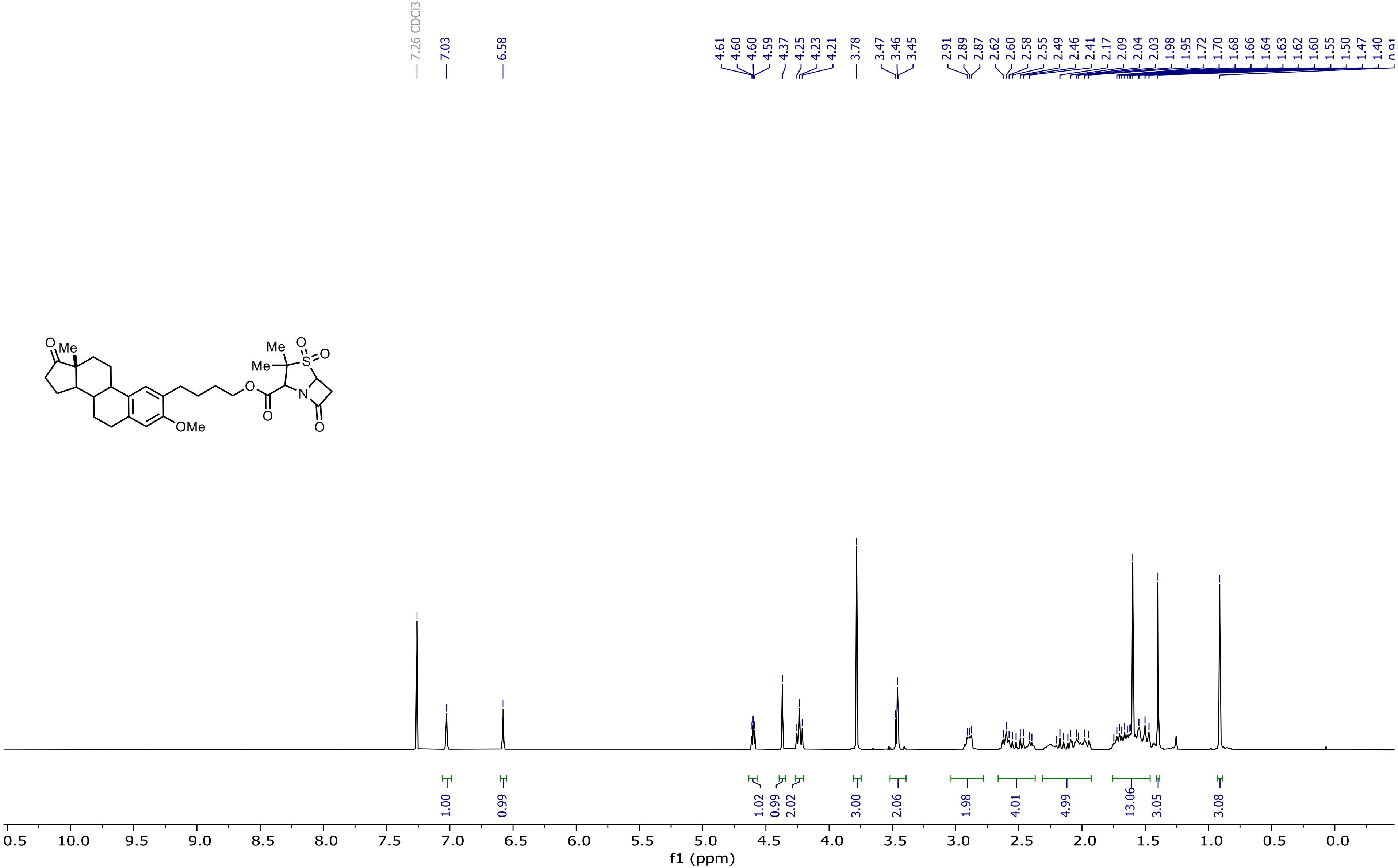

^{13}C NMR of sulbactam estrone methyl ether derivative 20

CDCl3, 23 °C

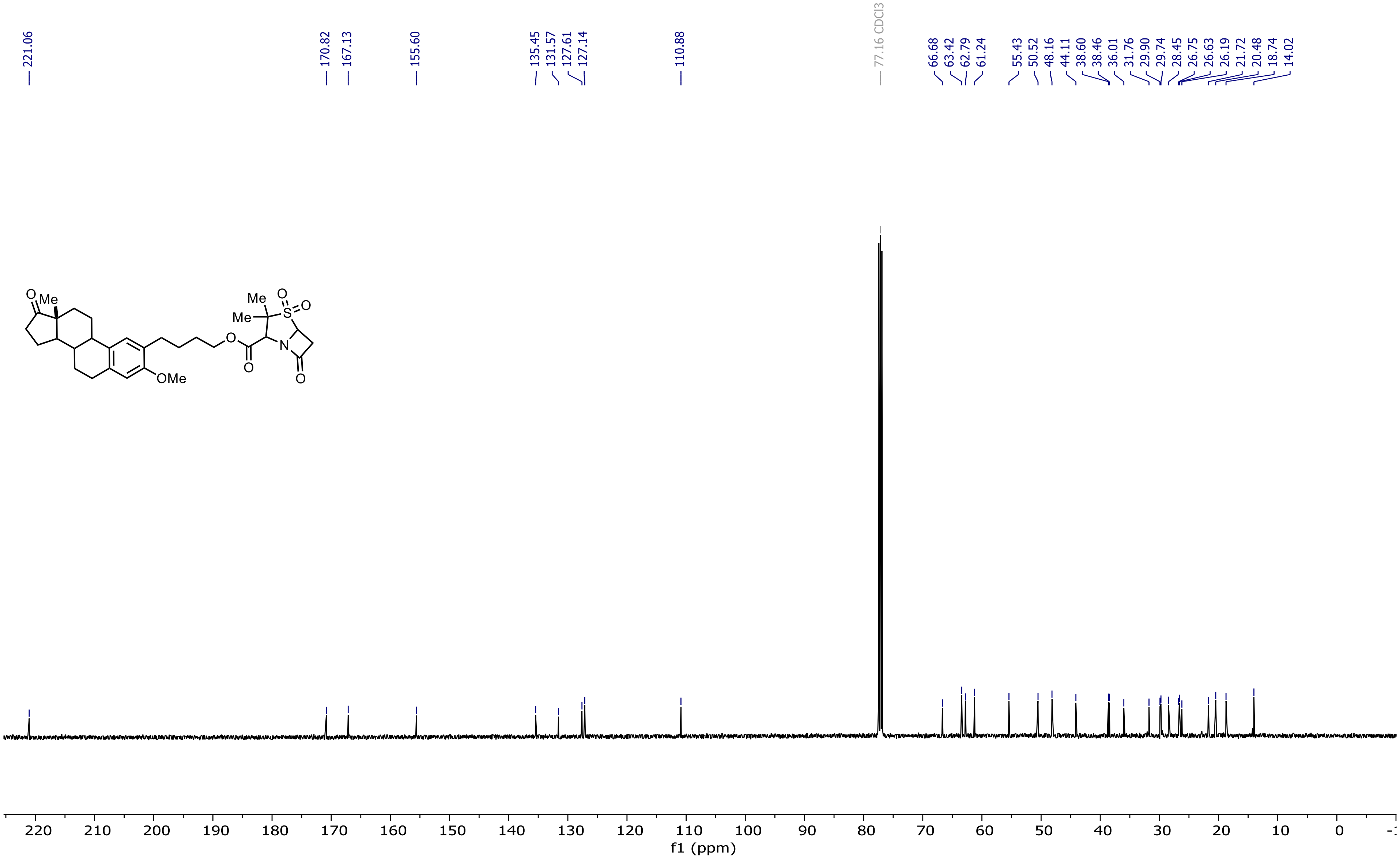

^{1}H NMR epiandrosterone flurbiprofen methyl ester derivative 21

CDCl$_3$, 23 ºC

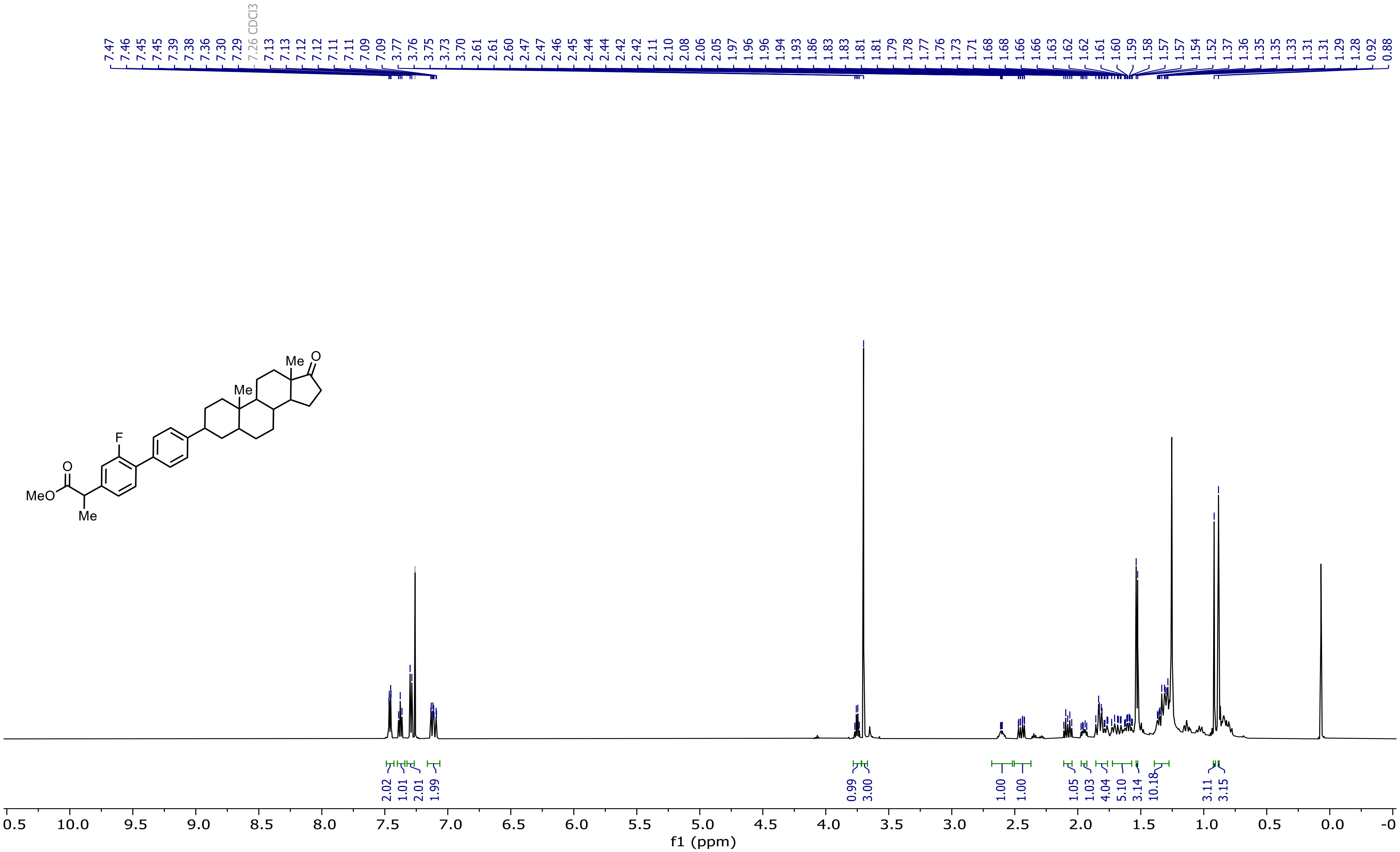

^{13}C NMR epiandrosterone flurbiprofen methyl ester derivative 21

CDCl$_3$, 23 ºC

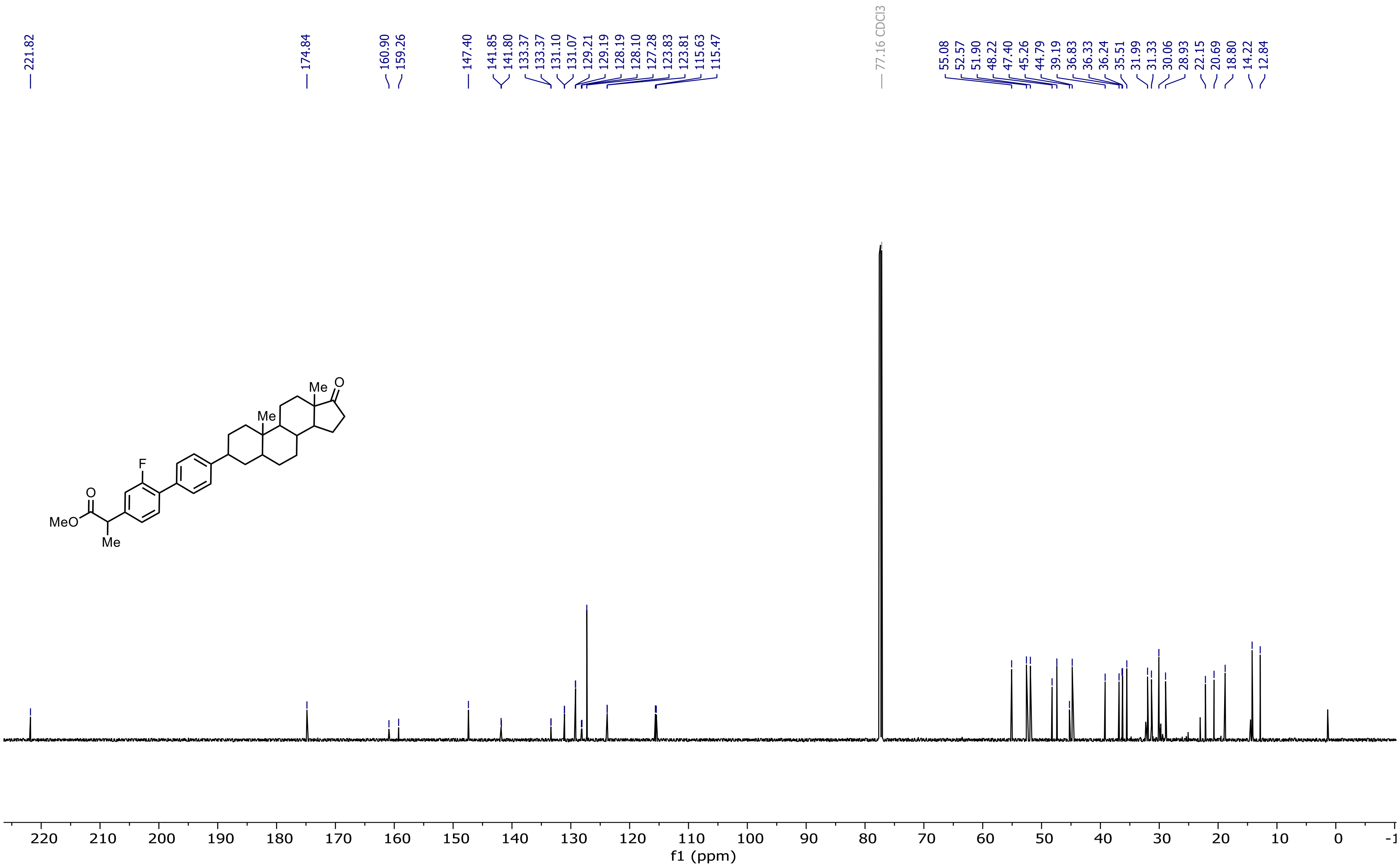

^{19}F NMR epiandrosterone flurbiprofen methyl ester derivative 21

$CDCl_3$, 23 ºC

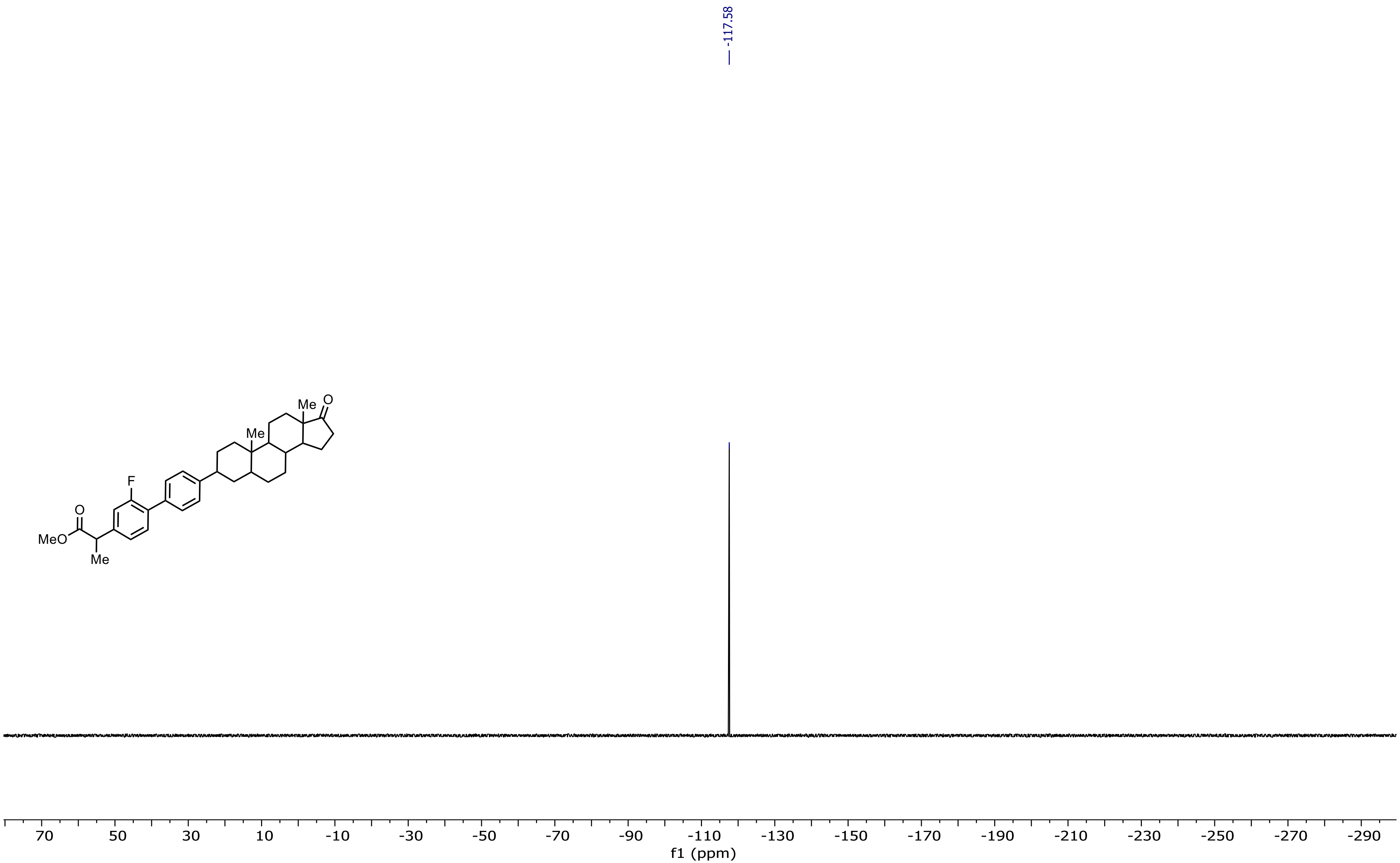

^{1}H NMR methyl 4-hydroxy-3-isopropylbenzoate 22, radical clock cyclization experiment

$CDCl_3$, 23 ºC

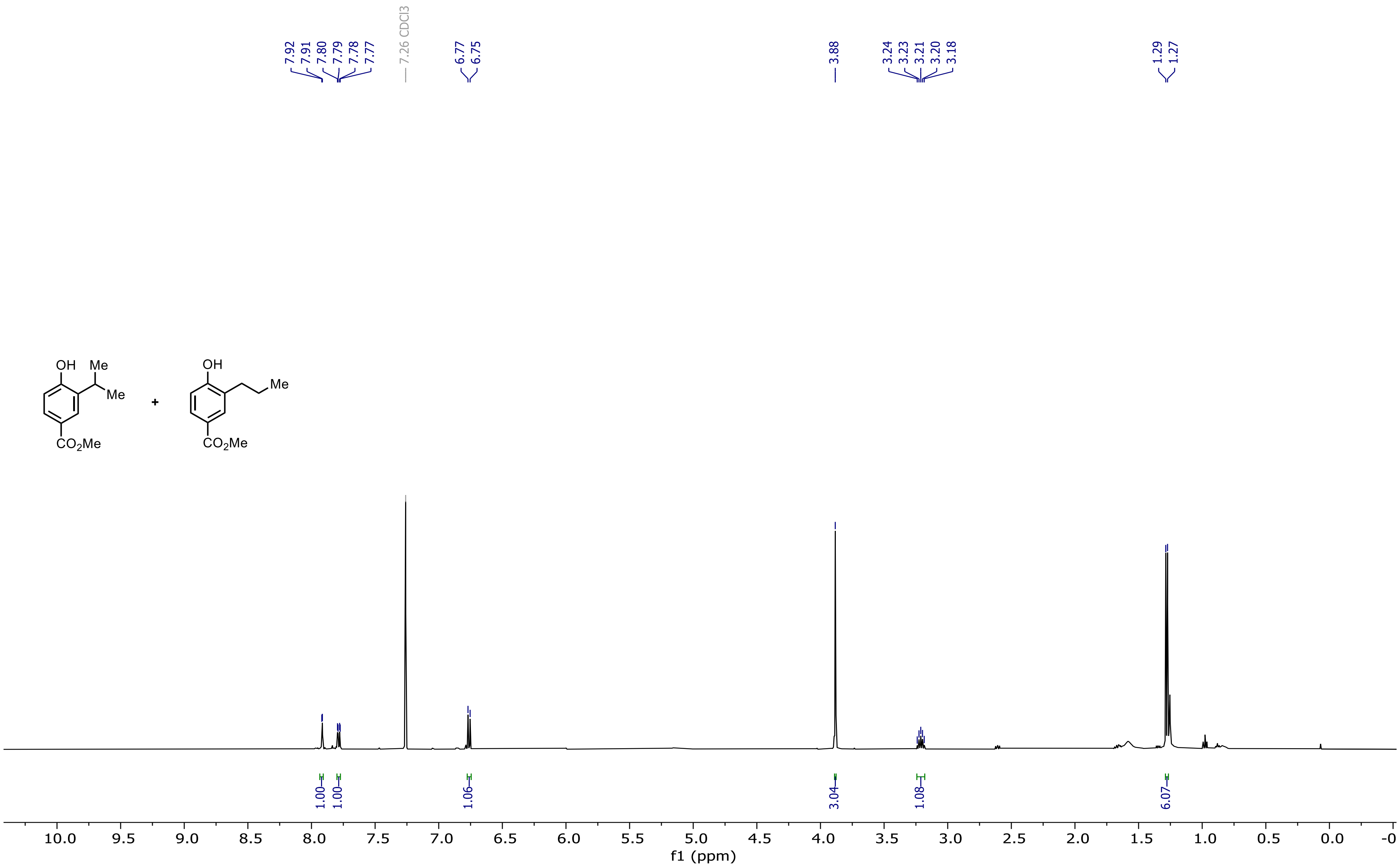

^{13}C NMR methyl 4-hydroxy-3-isopropylbenzoate 22, radical clock cyclization experiment

$CDCl_3$, 23 ºC

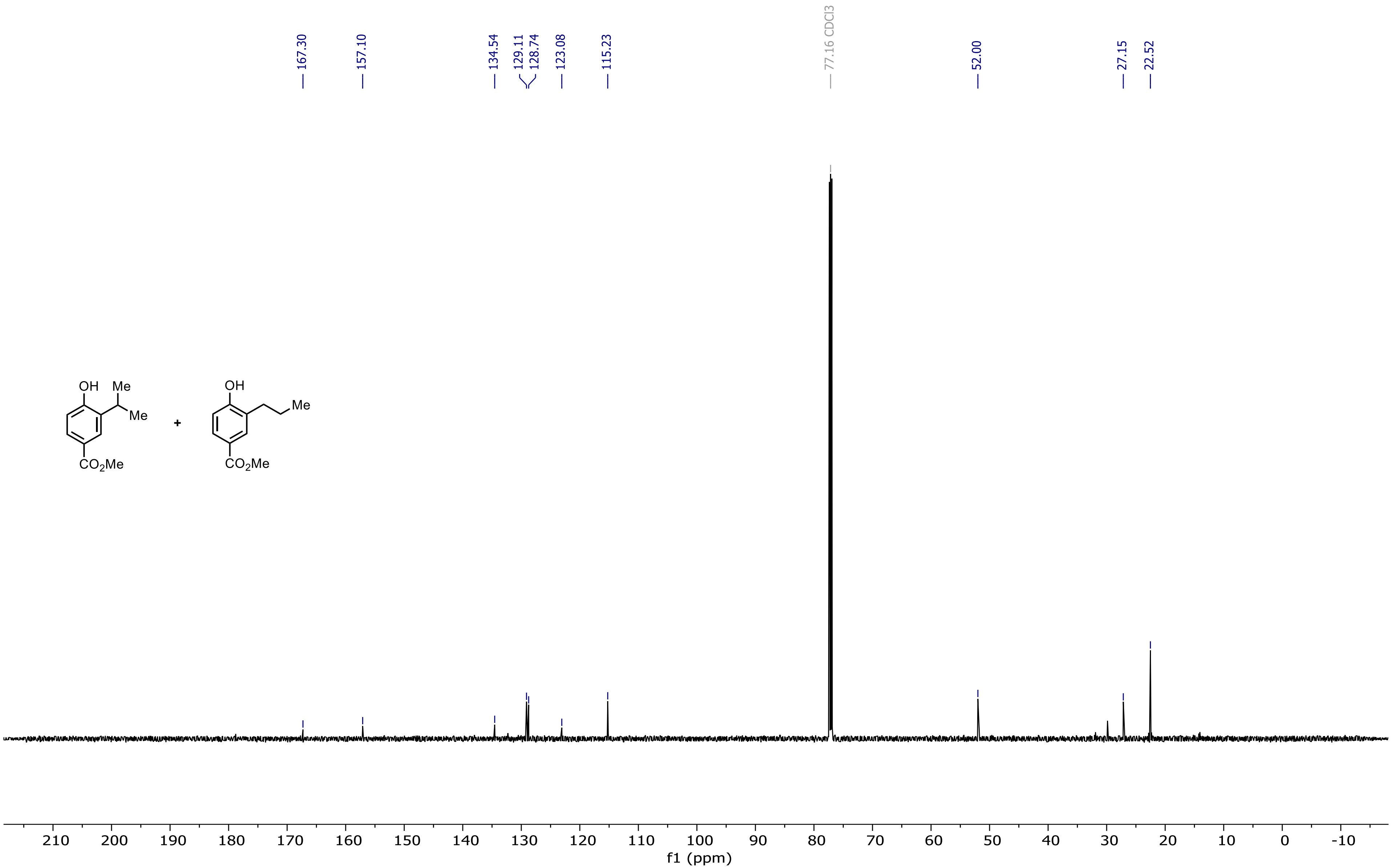

^{1}H NMR methyl 4-hydroxybenzoate 23, radical clock cyclization experiment

$CDCl_3$, 23 ºC

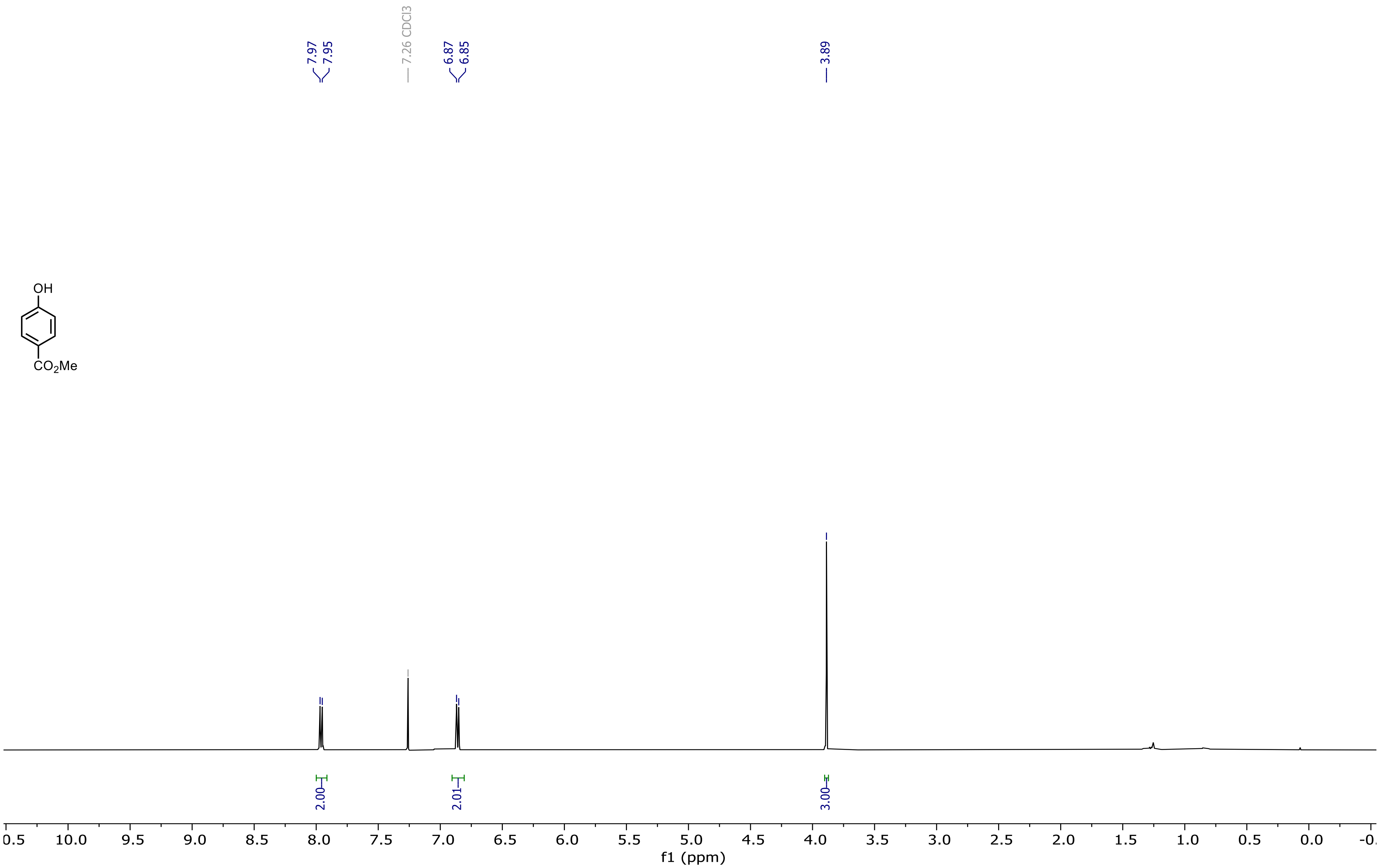

^{13}C NMR methyl 4-hydroxybenzoate 23, radical clock cyclization experiment

$CDCl_3$, 23 ºC

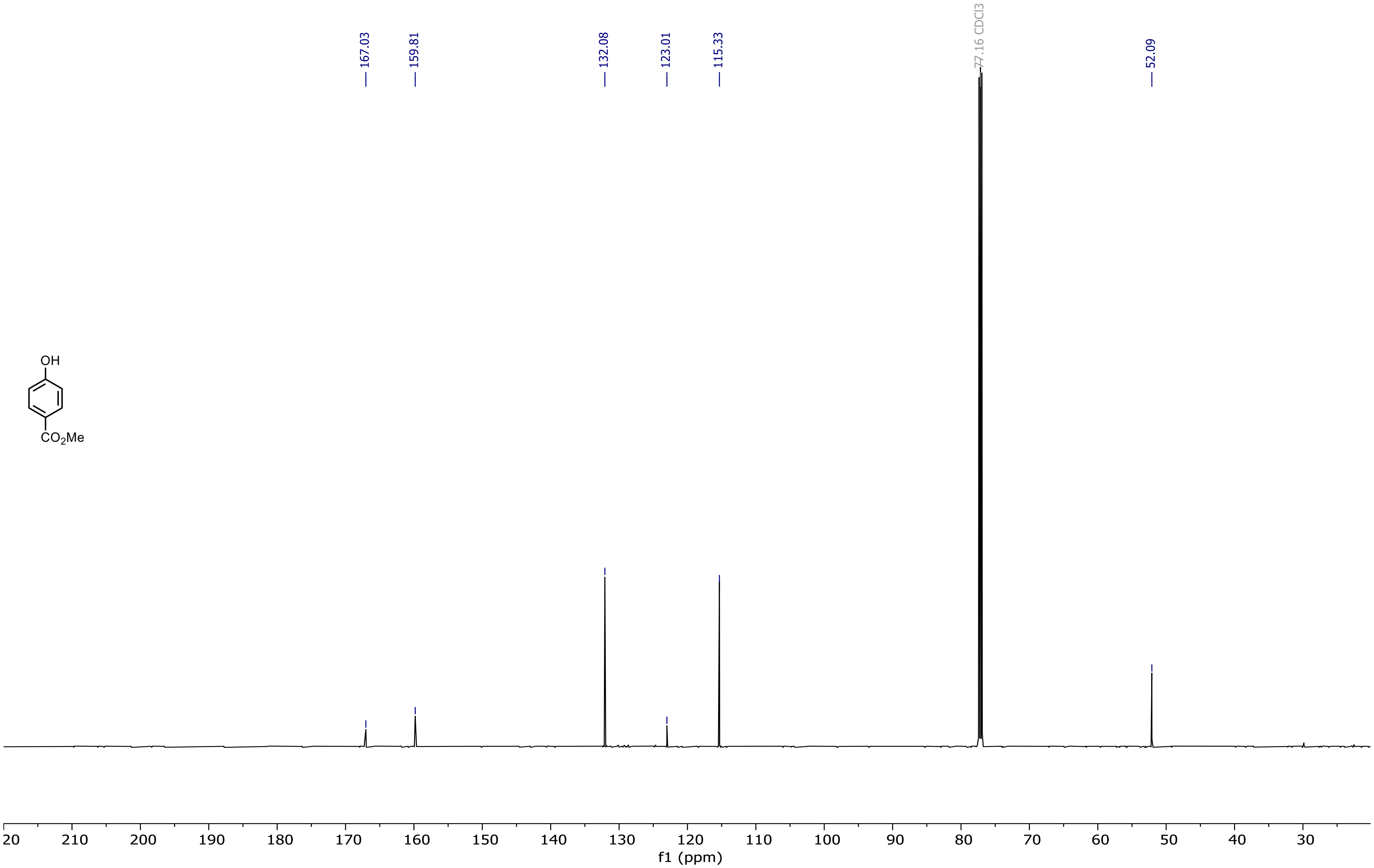

^{1}H NMR of boc-azetidinyl chlorobenzene derivative S-5

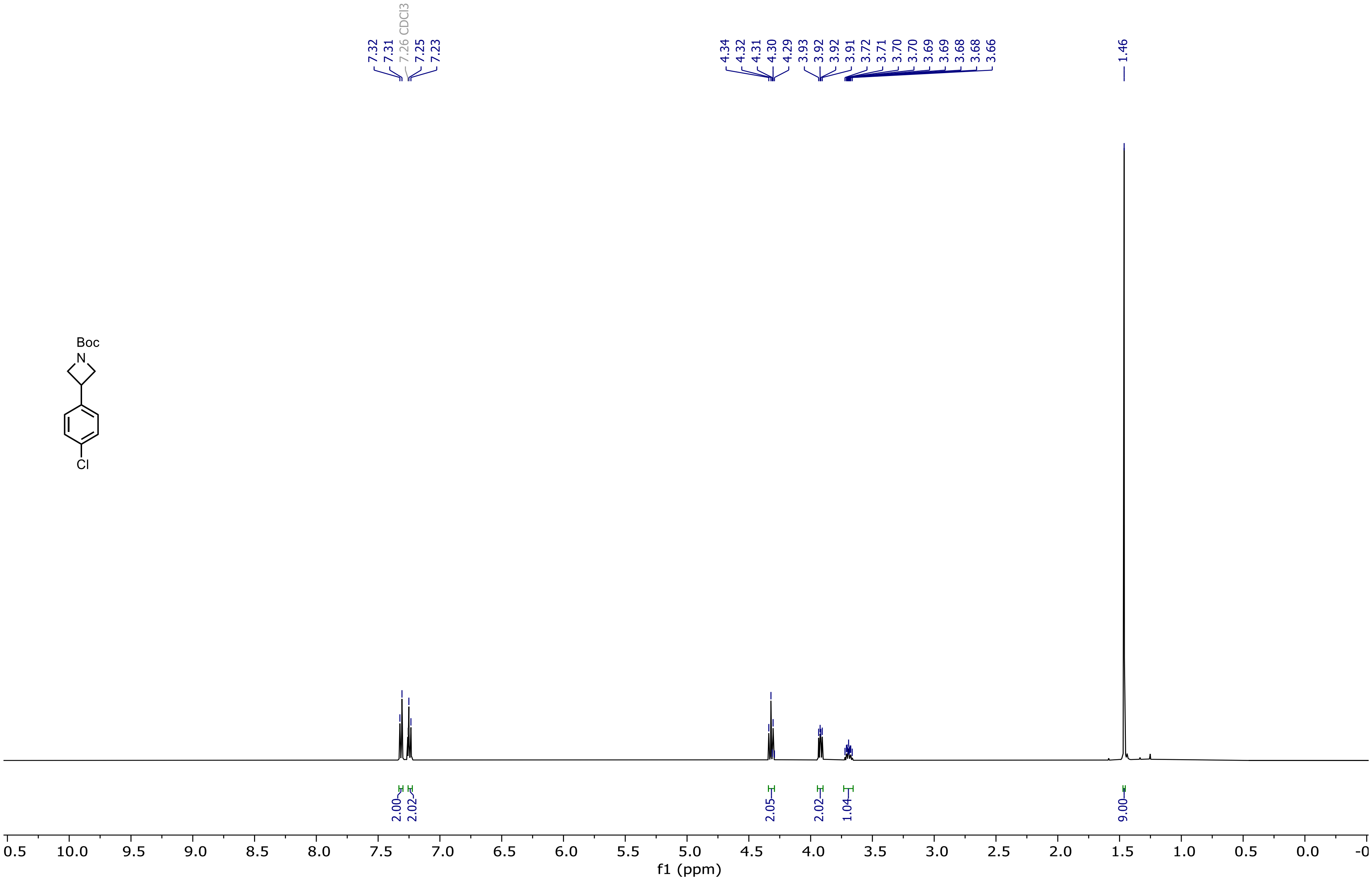

^{13}C NMR of boc-azetidinyl chlorobenzene derivative S-5

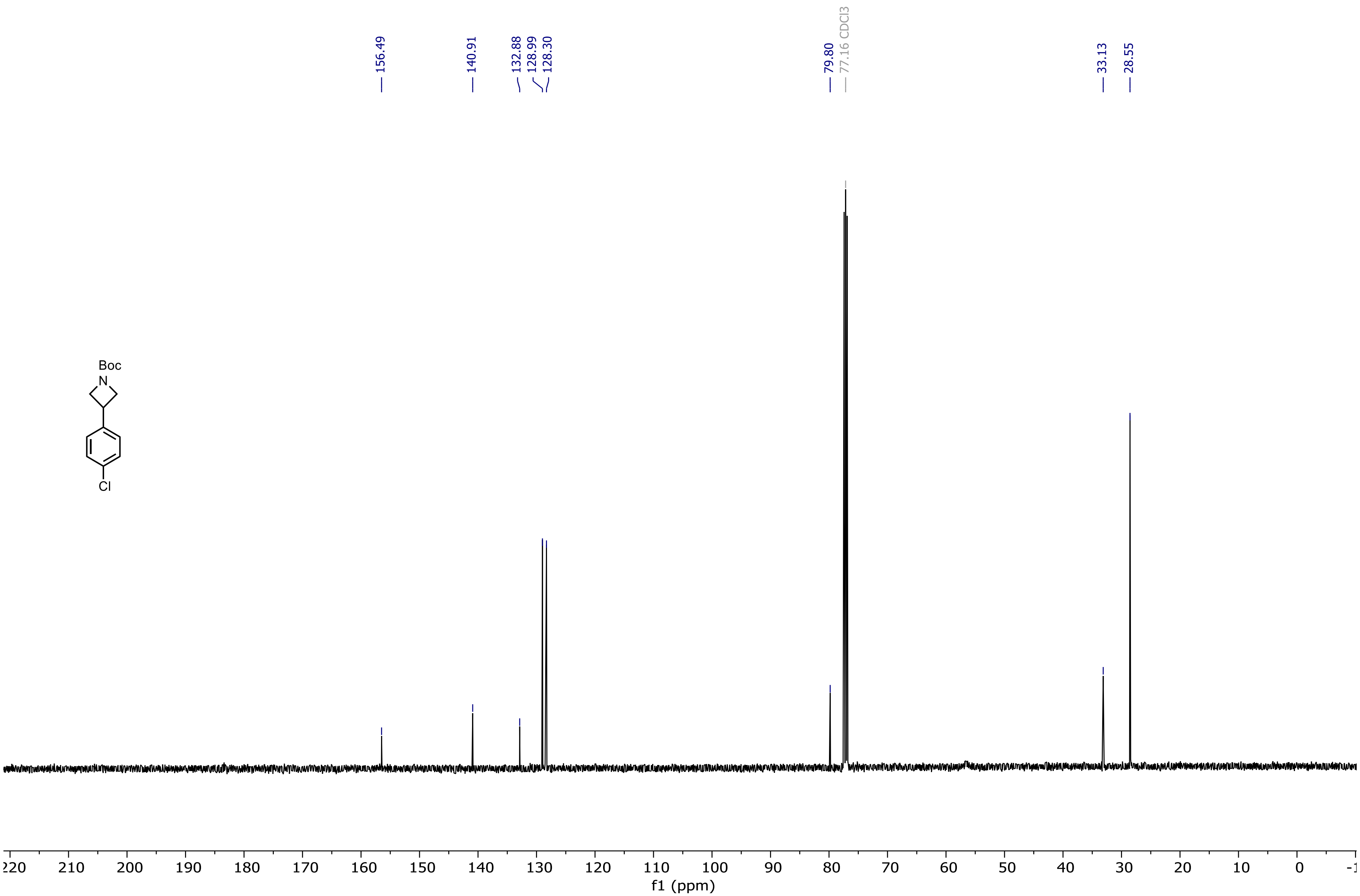

^{1}H NMR of methyl pyriproxyfen derivative S-6

CDCl$_3$, 23 °C

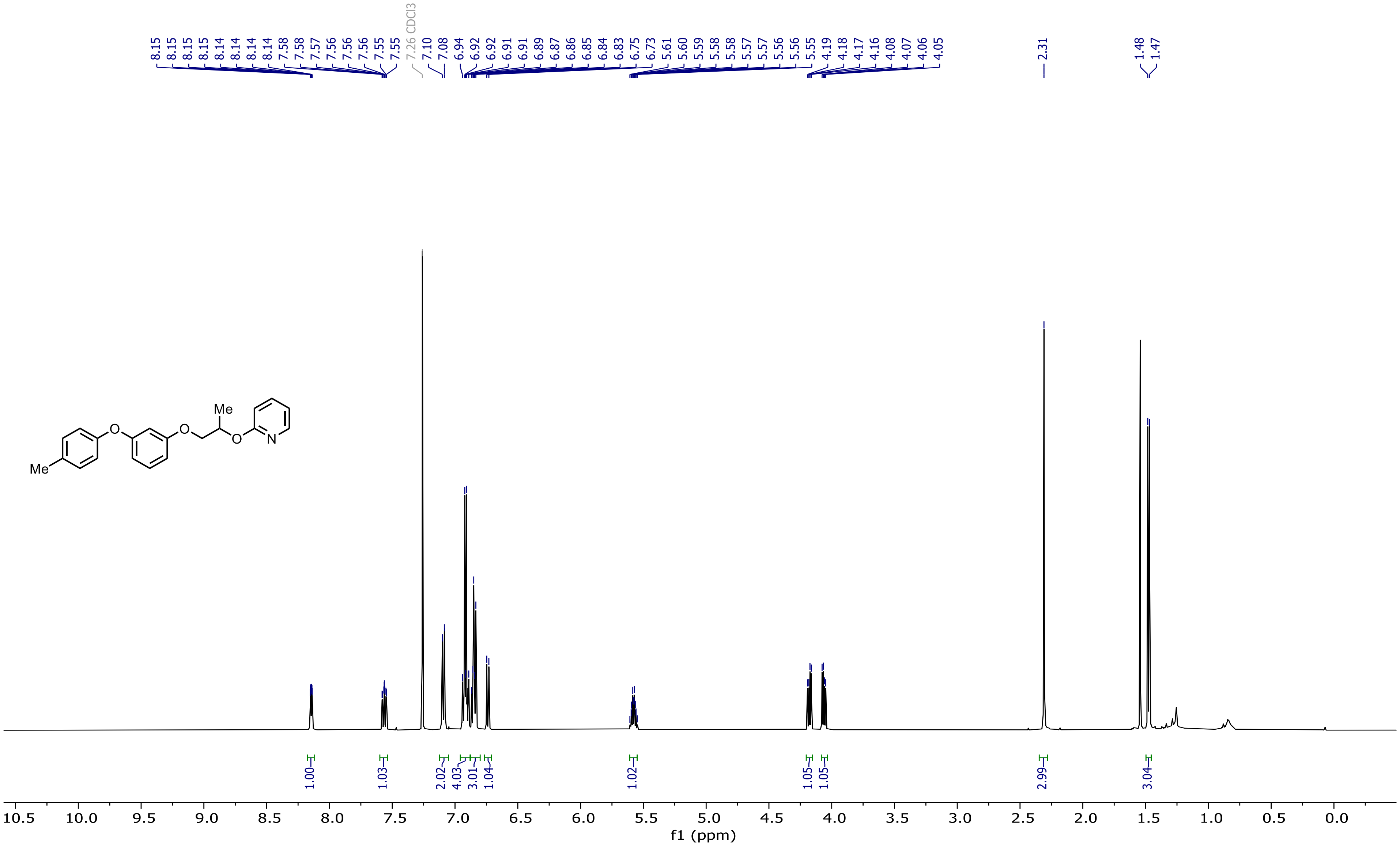

^{13}C NMR of methyl pyriproxyfen derivative S-6

$CDCl_3$, 23 °C

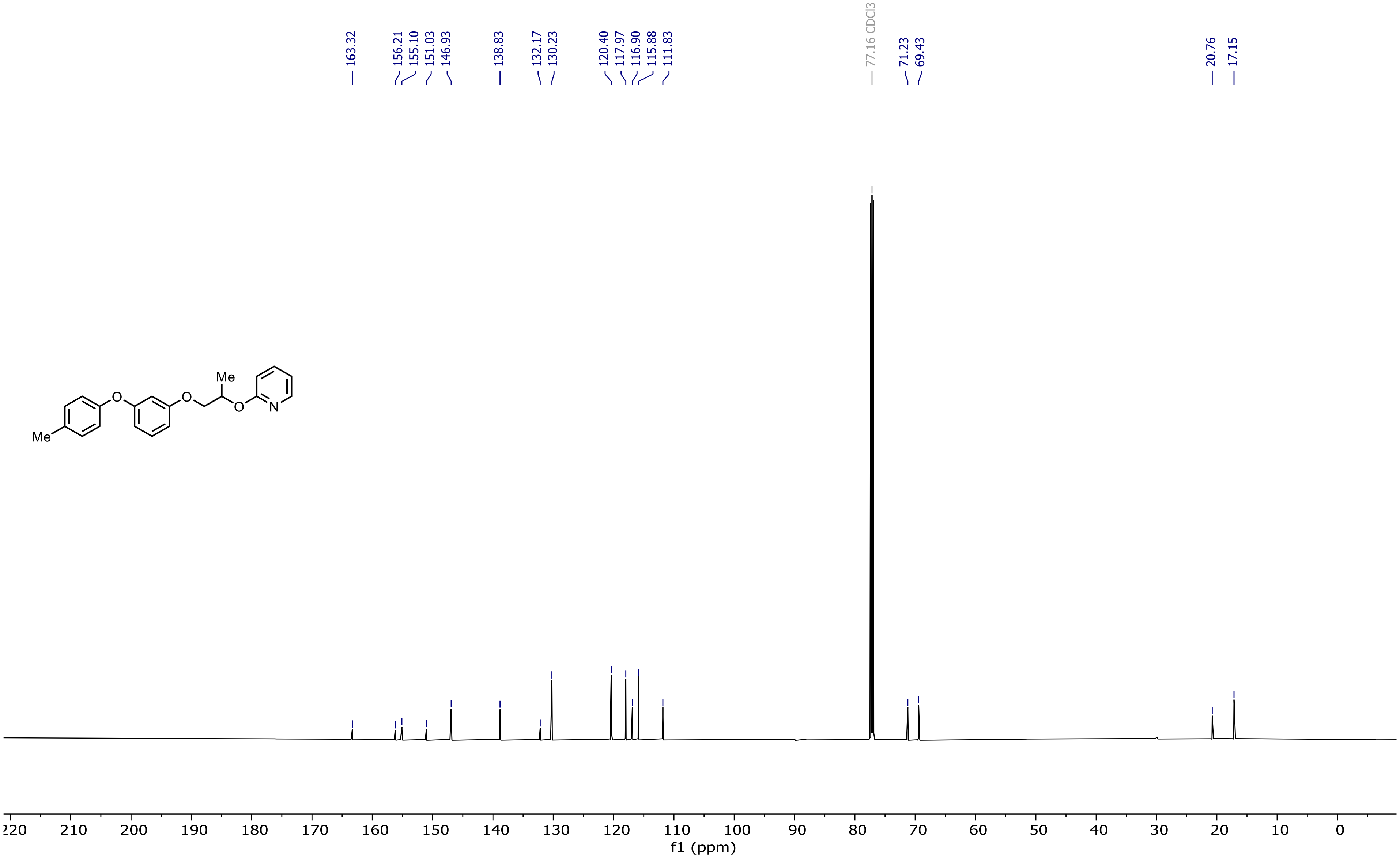

^{1}H NMR of fluoro butyl pyriproxyfen derivative S-7

$CDCl_3$, 23 °C

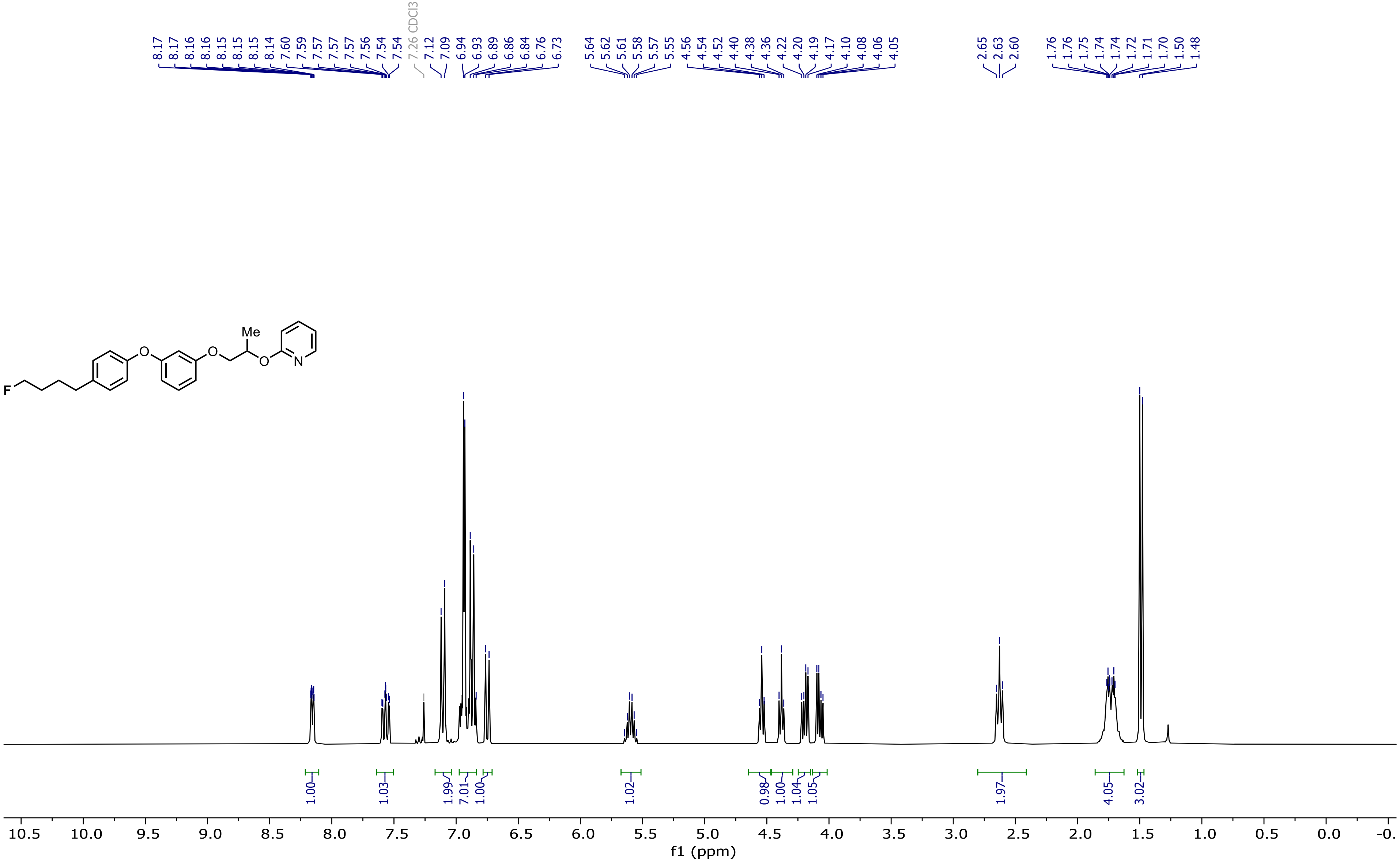

^{13}C NMR of fluoro butyl pyriproxyfen derivative S-7

CDCl$_3$, 23 °C

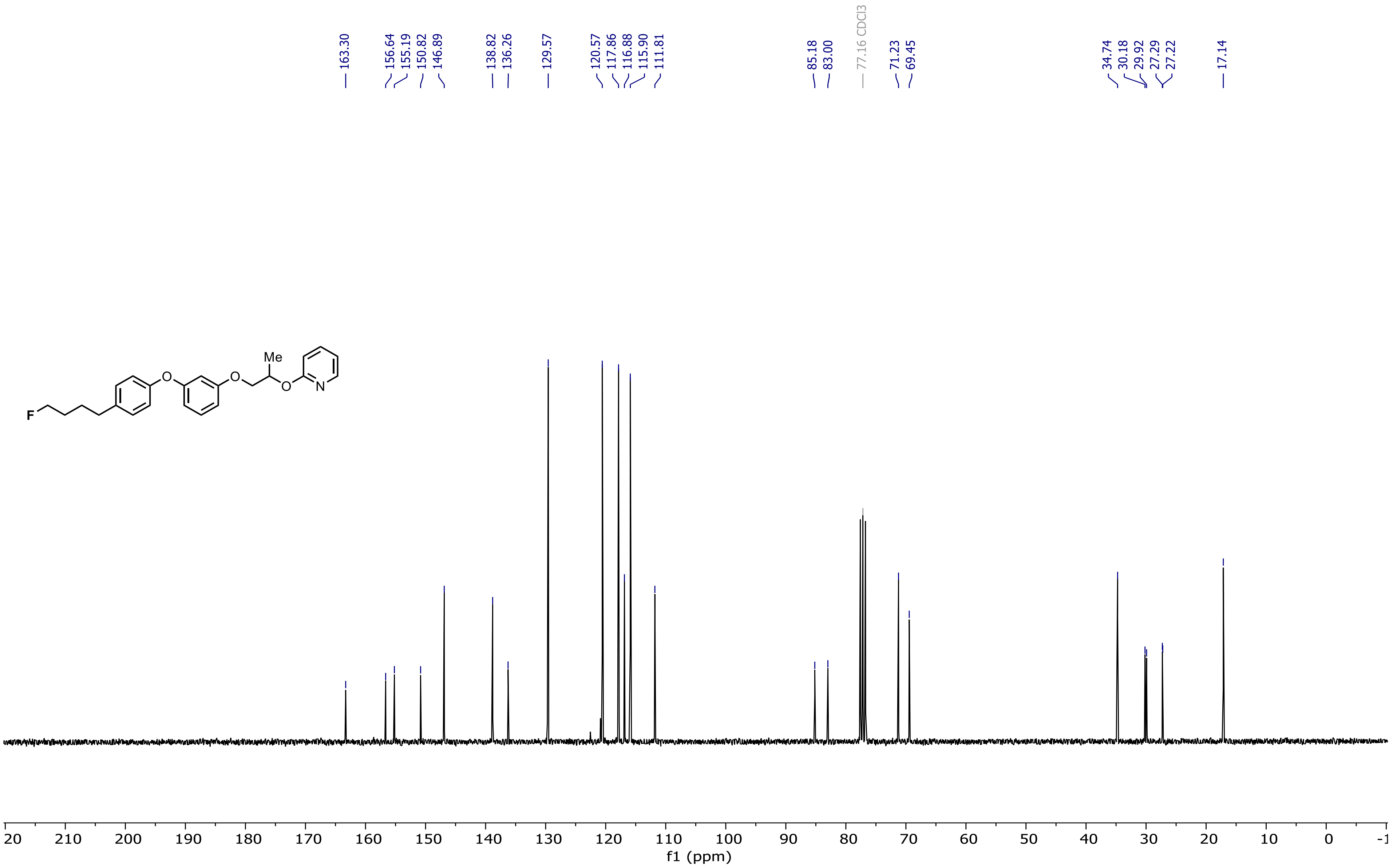

^{19}F NMR of fluoro butyl pyriproxyfen derivative S-7

$CDCl_3$, 23 °C

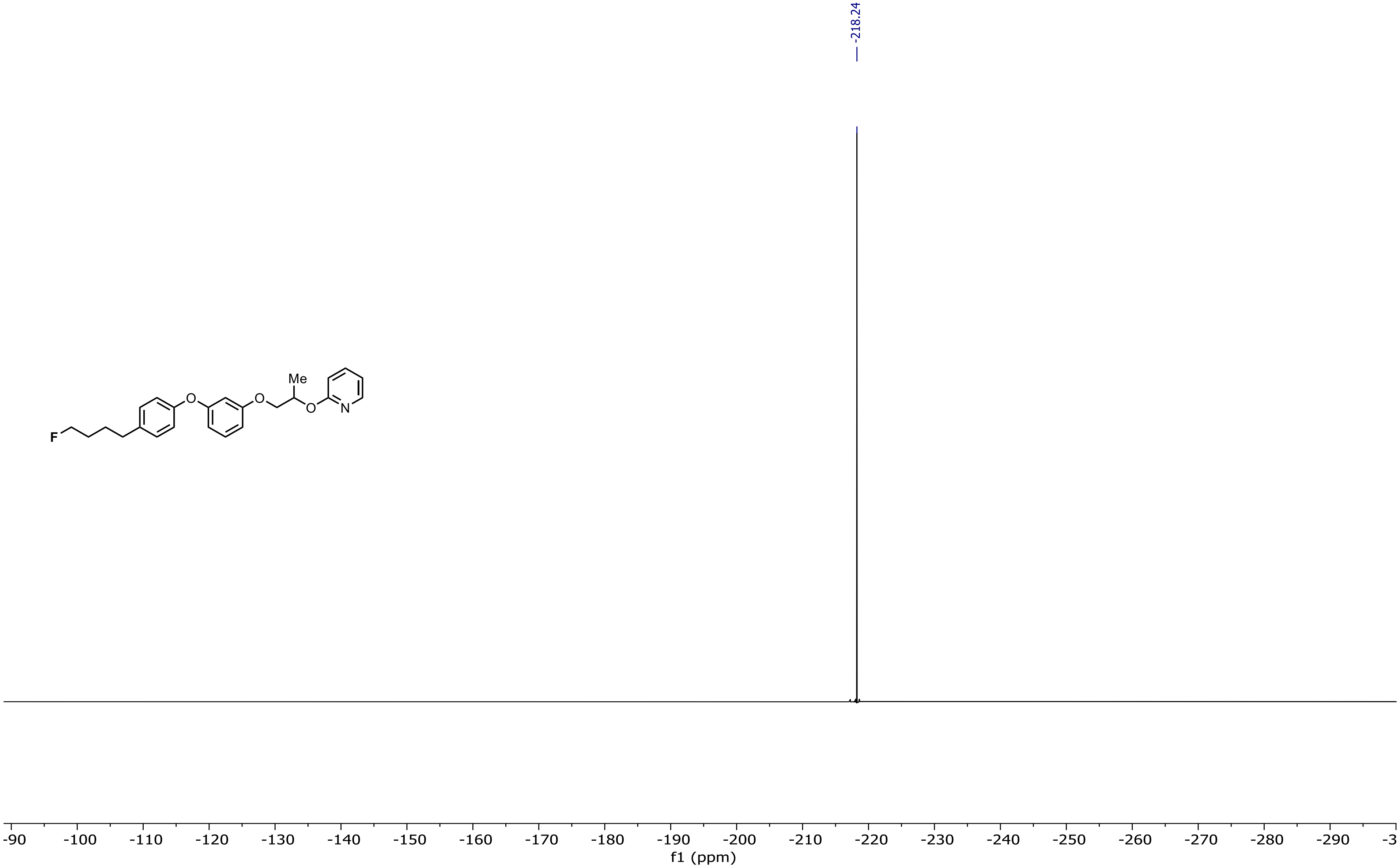

^{1}H NMR of butenyl pyriproxyfen derivative S-8 + S-9

CDCl3, 23 °C

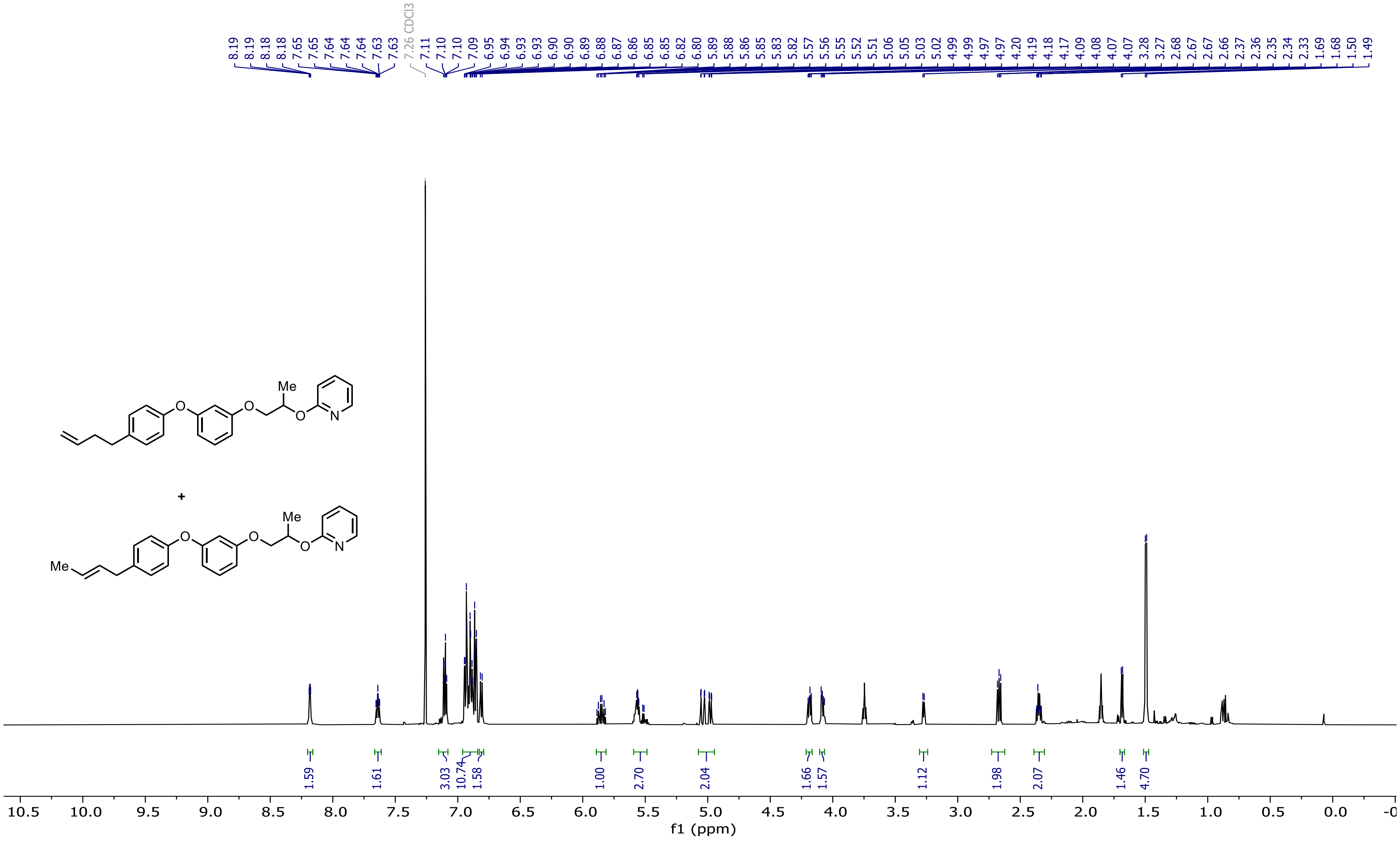

^{13}C NMR of butenyl pyriproxyfen derivative S-8 + S-9

CDCl$_3$, 23 °C

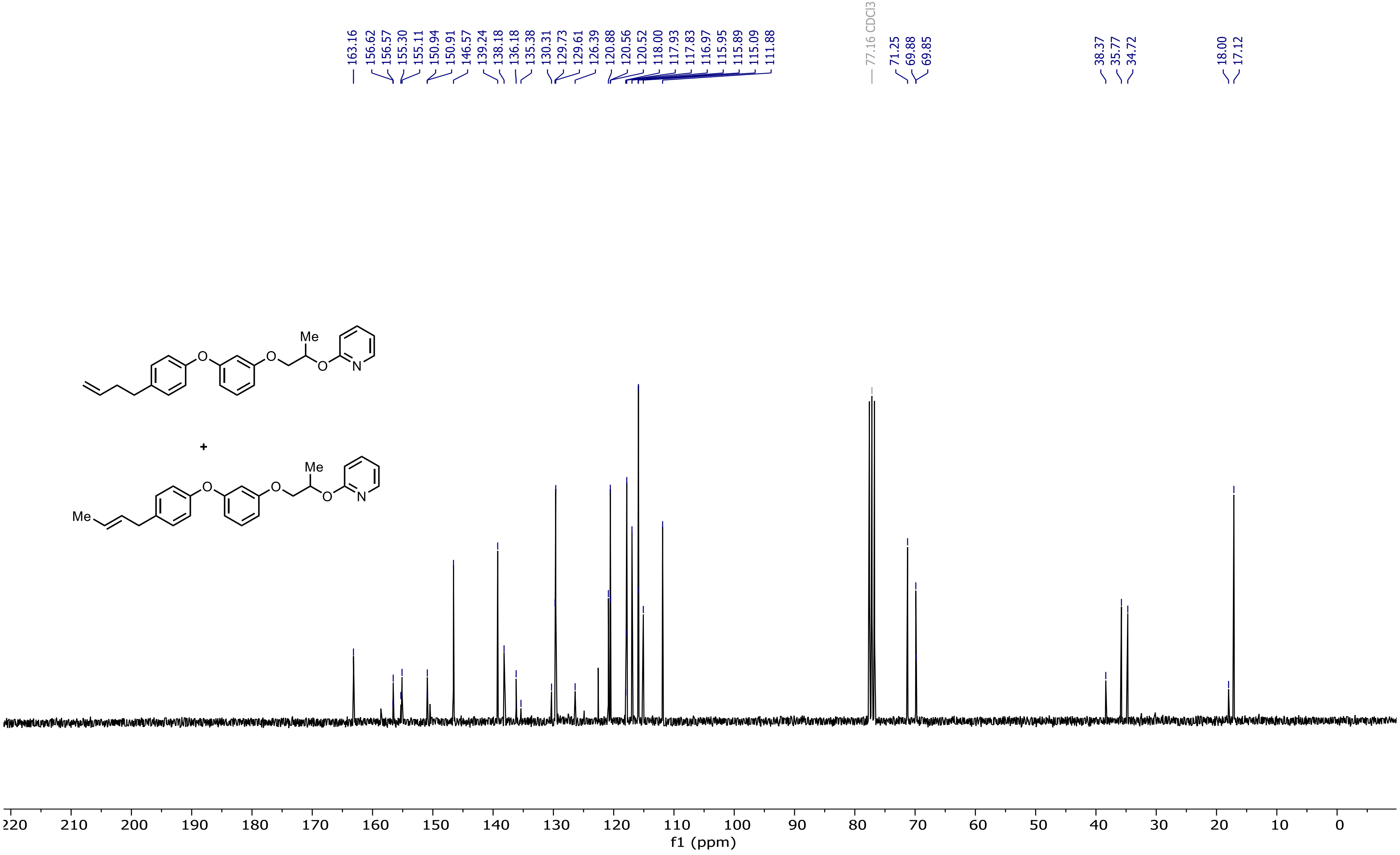

^{1}H NMR of boc-piperidinyl diphenyl ether derivative 3

$CDCl_3$, 23 °C

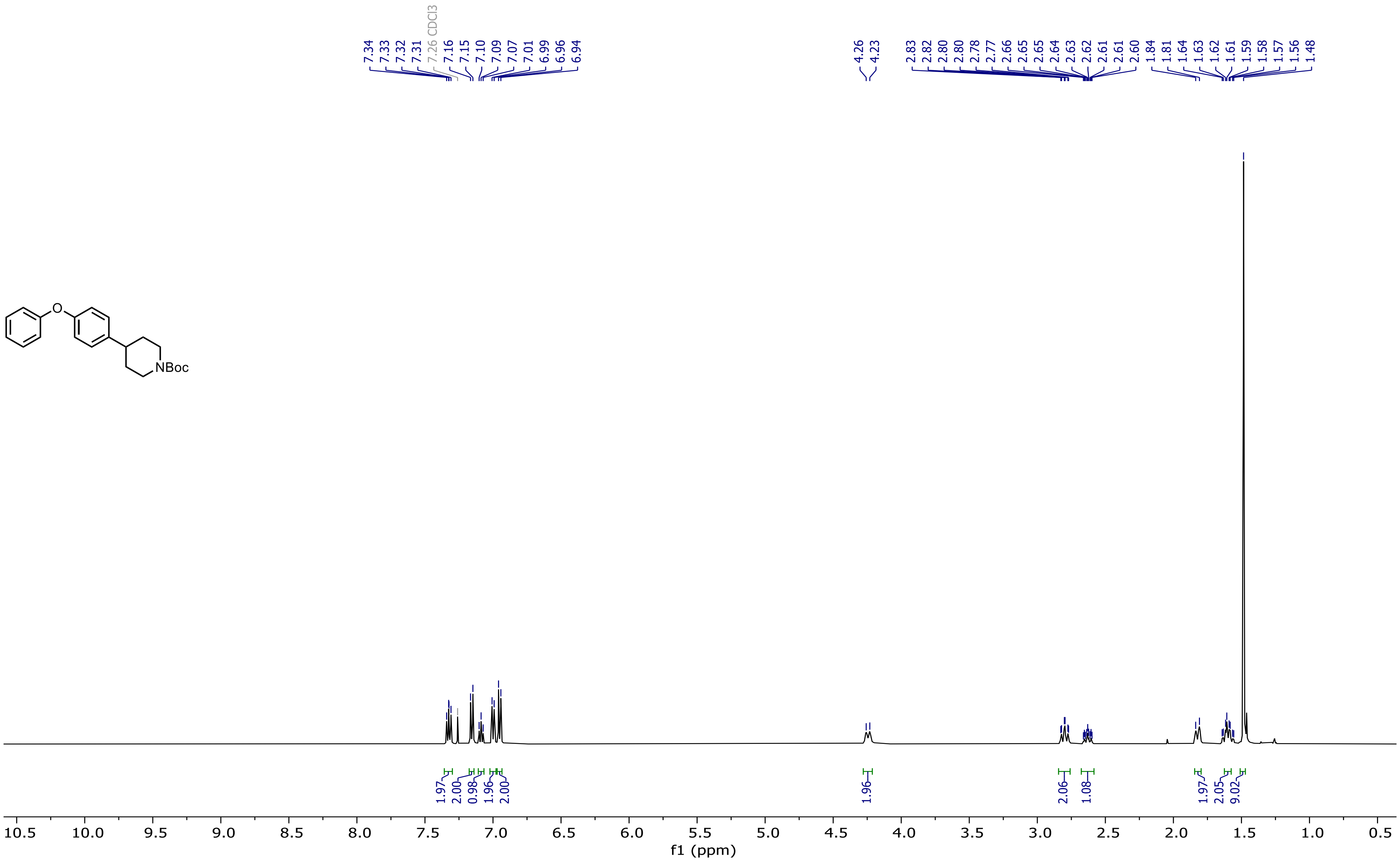

^{13}C NMR of boc-piperidinyl diphenyl ether derivative 3

$CDCl_3$, 23 °C

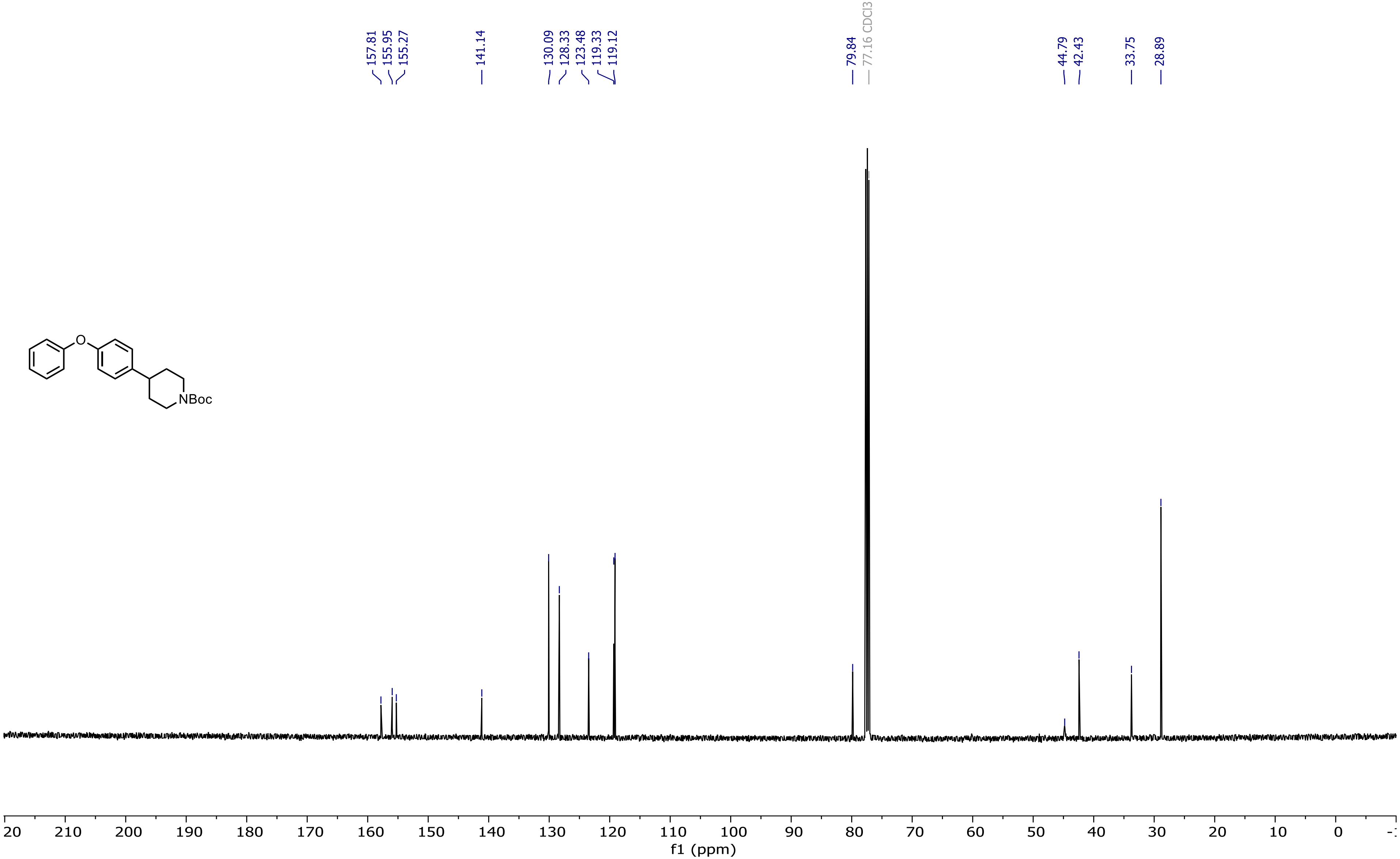

^{1}H NMR of radical clock starting material allyl ether thianthrenium salt TT-2

CD_2Cl_2, 23 °C

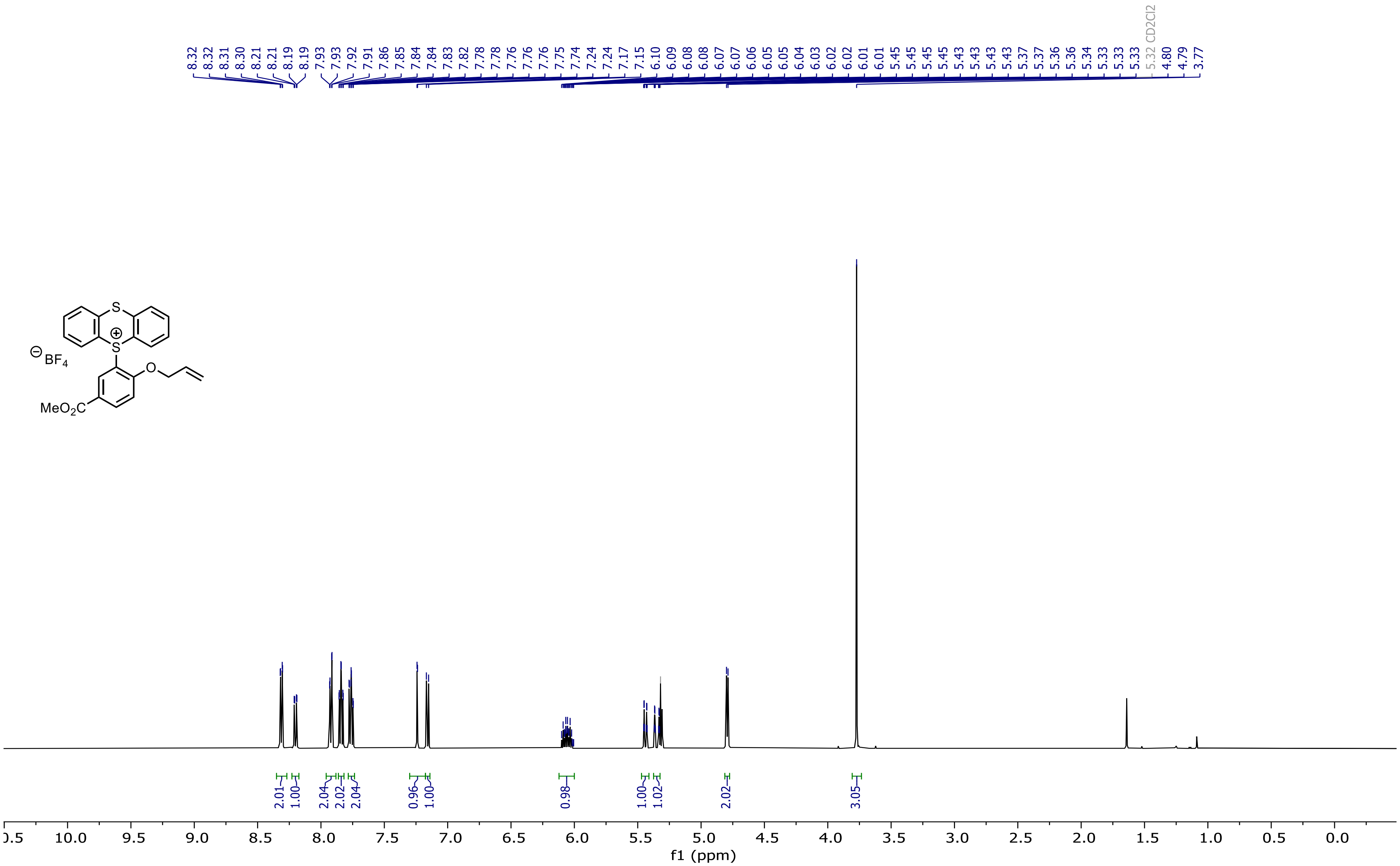

^{13}C NMR of radical clock starting material allyl ether thianthrenium salt TT-2

CD_2Cl_2, 23 °C

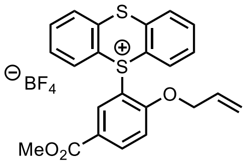

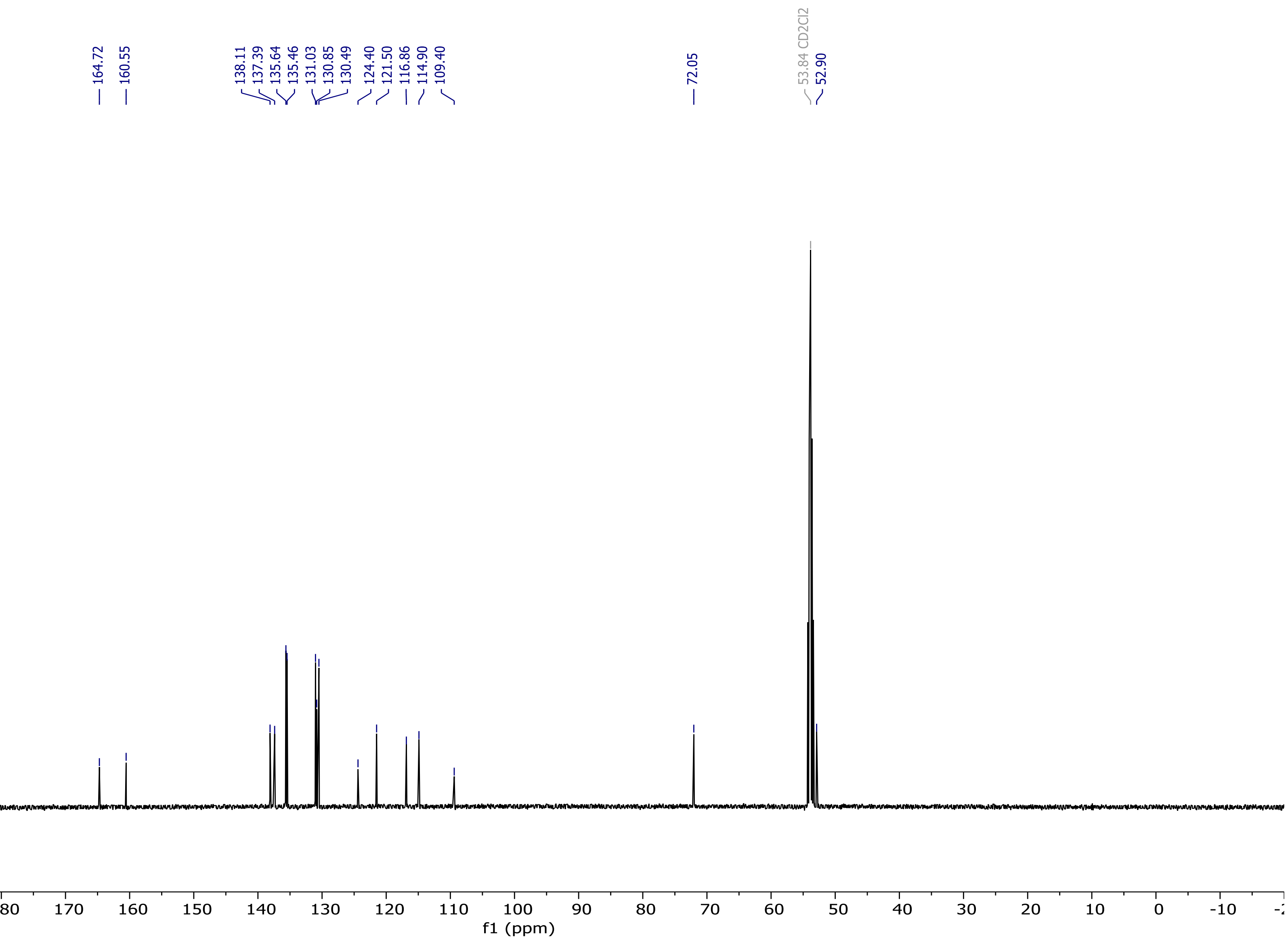

^{19}F NMR of radical clock starting material allyl ether thianthrenium salt TT-2

CD_2Cl_2, 23 °C

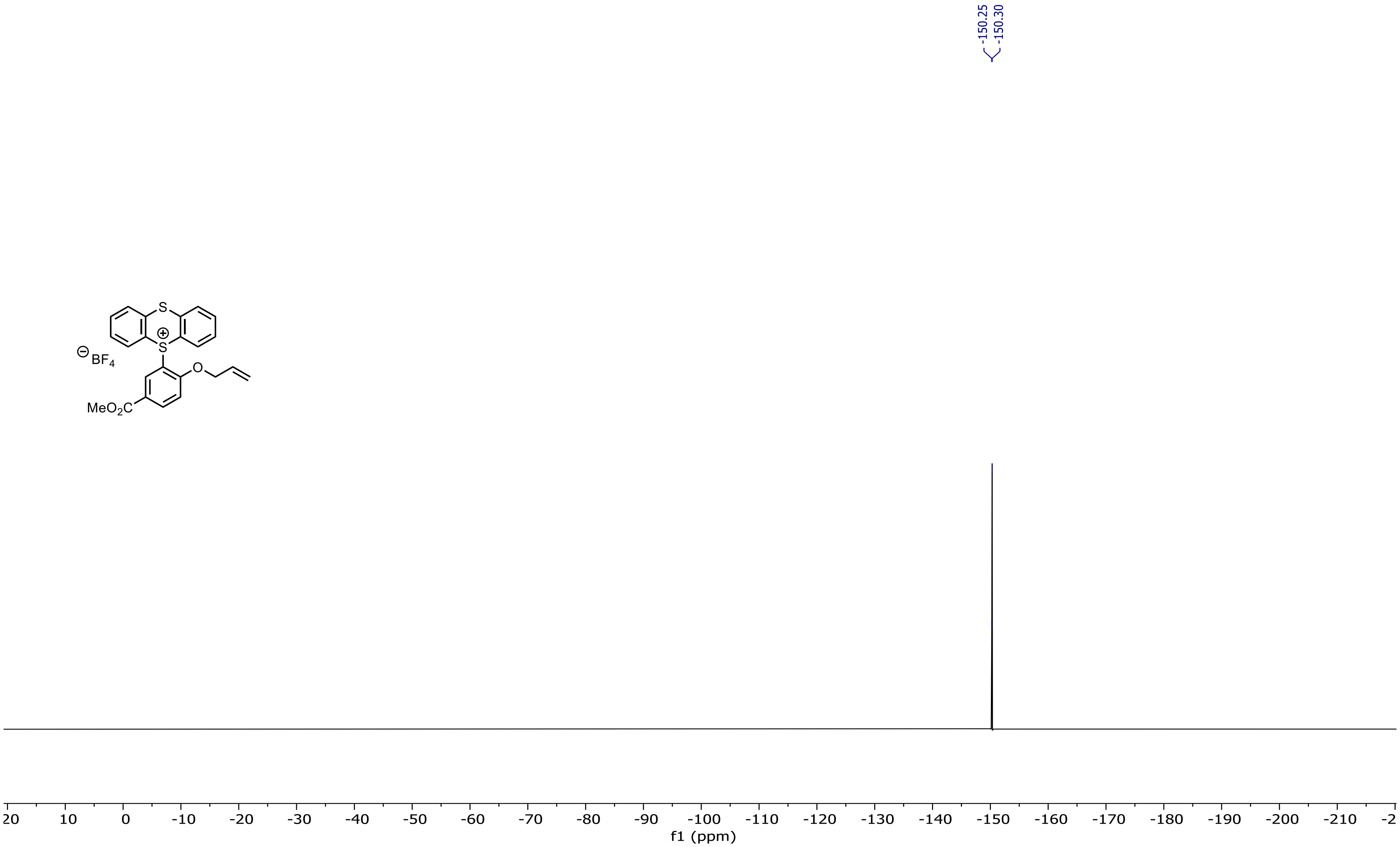

REFERENCES

1. Fulmer, G. R.; Miller, A. J.; Sherden, N. H.; Gottlieb, H. E.; Nudelman, A.; Stoltz, B. M.; Bercaw, J. E.; Goldberg, K. I., NMR chemical shifts of trace impurities: common laboratory solvents, organics, and gases in deuterated solvents relevant to the organometallic chemist. *Organometallics* **2010**, *29* (9), 2176-2179.
2. Berger, F.; Plutschack, M. B.; Riegger, J.; Yu, W.; Speicher, S.; Ho, M.; Frank, N.; Ritter, T., Site-selective and versatile aromatic C− H functionalization by thianthrenation. *Nature* **2019**, *567* (7747), 223-228.
3. Alvarez, E. M.; Plutschack, M. B.; Berger, F.; Ritter, T., Site-Selective C–H Functionalization–Sulfination Sequence to Access Aryl Sulfonamides. *Org. Lett.* **2020**, *22* (12), 4593-4596.
4. Engl, P. S.; Häring, A. P.; Berger, F.; Berger, G.; Pérez-Bitrián, A.; Ritter, T., C–N cross-couplings for site-selective late-stage diversification via aryl sulfonium salts. *J. Am. Chem. Soc.* **2019**, *141* (34), 13346-13351.
5. Li, J.; Chen, J.; Sang, R.; Ham, W.-S.; Plutschack, M. B.; Berger, F.; Chabbra, S.; Schnegg, A.; Genicot, C.; Ritter, T., Photoredox catalysis with aryl sulfonium salts enables site-selective late-stage fluorination. *Nat. Chem.* **2020**, *12* (1), 56-62.
6. Sang, R.; Korkis, S. E.; Su, W.; Ye, F.; Engl, P. S.; Berger, F.; Ritter, T., Site‐Selective C− H Oxygenation via Aryl Sulfonium Salts. *Angew. Chem. Int. Ed.* **2019**, *131* (45), 16307-16312.
7. Ye, F.; Berger, F.; Jia, H.; Ford, J.; Wortman, A.; Börgel, J.; Genicot, C.; Ritter, T., Aryl sulfonium salts for site‐selective late‐stage trifluoromethylation. *Angew. Chem. Int. Ed.* **2019**, *131* (41), 14757-14761.
8. Thomas, L.; Lutter, F. H.; Hofmayer, M. S.; Karaghiosoff, K.; Knochel, P., Cobalt-Catalyzed Diastereoselective Cross-Couplings between Alkynylzinc Pivalates and Functionalized Cyclic Iodides or Bromides. *Org. Lett.* **2018**, *20* (8), 2441-2444.
9. Mo, F.; Yan, J. M.; Qiu, D.; Li, F.; Zhang, Y.; Wang, J. J. A. C. I. E., Gold‐Catalyzed Halogenation of Aromatics by N‐Halosuccinimides. **2010**, *49* (11), 2028-2032.
10. Tang, R.-J.; Milcent, T.; Crousse, B., Regioselective halogenation of arenes and heterocycles in hexafluoroisopropanol. *J. Org. Chem.* **2018**, *83* (2), 930-938.
